# Evolution of the mammalian lysozyme gene family

**DOI:** 10.1186/1471-2148-11-166

**Published:** 2011-06-15

**Authors:** David M Irwin, Jason M Biegel, Caro-Beth Stewart

**Affiliations:** 1 Department of Laboratory Medicine and Pathobiology, University of Toronto, Toronto, Canada; 2 Banting and Best Diabetes Centre, University of Toronto, Toronto, Canada; 3 Department of Biological Sciences, University at Albany, State University of New York, Albany, New York 12222, USA

## Abstract

**Background:**

Lysozyme *c *(chicken-type lysozyme) has an important role in host defense, and has been extensively studied as a model in molecular biology, enzymology, protein chemistry, and crystallography. Traditionally, lysozyme *c *has been considered to be part of a small family that includes genes for two other proteins, lactalbumin, which is found only in mammals, and calcium-binding lysozyme, which is found in only a few species of birds and mammals. More recently, additional testes-expressed members of this family have been identified in human and mouse, suggesting that the mammalian lysozyme gene family is larger than previously known.

**Results:**

Here we characterize the extent and diversity of the lysozyme gene family in the genomes of phylogenetically diverse mammals, and show that this family contains at least eight different genes that likely duplicated prior to the diversification of extant mammals. These duplicated genes have largely been maintained, both in intron-exon structure and in genomic context, throughout mammalian evolution.

**Conclusions:**

The mammalian lysozyme gene family is much larger than previously appreciated and consists of at least eight distinct genes scattered around the genome. Since the lysozyme *c *and lactalbumin proteins have acquired very different functions during evolution, it is likely that many of the other members of the lysozyme-like family will also have diverse and unexpected biological properties.

## Background

The vertebrate lysozyme gene family has traditionally been considered to be composed of three genes: lysozyme *c*, lactalbumin, and calcium-binding lysozyme [1-4]. Lysozyme *c*, chicken-type (or conventional) lysozyme, is a bacteriolytic enzyme that is secreted into many body fluids of mammals (*e.g., *blood, tears, and milk) and is found at a high concentration in the eggs of many bird species [1,2,5]. Lysozyme *c *is widespread in nature; its protein and gene sequences have been characterized from numerous diverse vertebrate and non-vertebrate species [3,5,6]. Lactalbumin is related to lysozyme, with around 40% amino acid identity and nearly identical three-dimensional structure, but lacks its bacteriolytic activity [1,2,4,7]. Lactalbumin is expressed in lactating mammary glands, where it binds a calcium ion and modifies the activity of β-galactosyltransferase-1, such that the complex catalyzes the synthesis of lactose [2,4,7]. Lactalbumin has recently been shown to have a second activity in the gut, where it loses the calcium ion and binds a fatty acid; this new form of lactalbumin appears to promote apoptosis of tumor cells, and thus has been renamed HAMLET (human lactalbumin made lethal to tumors) [8]. Lactalbumin appears to be found only in mammals, and is widely distributed in this group. Calcium-binding lysozyme has bacteriolytic activity like lysozyme *c*, but also shares with lactalbumin the ability to bind a calcium ion. Calcium-binding lysozymes appear to be relatively rare; they have been found in the milk of only a few mammalian species (*e.g*., horse, dog, cat, seal, and echidna), as well as in the eggs (*e.g., *pigeon) and stomachs (*e.g*., hoatzin) of some bird species [3,9]. Indeed, calcium-binding lysozyme genes have not been reported for the human or rodent genomes.

Previous phylogenetic analyses of lysozyme *c*, lactalbumin, and calcium-binding lysozyme sequences had suggested that the earliest divergences within this gene family occurred between lysozyme *c *and the ancestor of the genes for lactalbumin and calcium-binding lysozyme, and that this initial gene duplication may have preceded the divergence of the lineages leading to fish and mammals [10,11]. The separation of the lactalbumin and calcium-binding lysozyme genes was proposed to be more recent, with some studies [9,12] suggesting a divergence on the early mammalian lineage, which would be consistent with the restriction of the lactalbumin gene to mammals. In contrast, another study [11] suggested that the duplication generating the lactalbumin and calcium-binding lysozyme genes predated the bird-mammal divergence. Moreover, the orthology of the mammalian and avian calcium-binding lysozymes has even been questioned [3,11]. Thus, the origin of these mammalian lysozyme-like genes remains an open question.

Recently, cDNAs for several additional lysozyme-like sequences have been identified from human testis cDNA libraries [13-15]. These cDNAs were found to be encoded by genes that are now annotated by *Ensembl *[16] as *LYZL *(lysozyme-like): *LYZL2, LYZL4, LYZL6 *and *LYZL3 *(Synonym *SPACA3; SPACA*, Sperm acrosome associated [15]. *SPACA3 *is also known as *SPRSA *[14] and *SLLP1 *[13]). The predicted protein sequences of some of these lysozyme-like sequences have amino acid substitutions at sites important for the catalytic activity of lysozyme, suggesting that these proteins would not be able to hydrolyze the glycosidic bonds of bacterial peptidoglycan [13,15]. Since these four new lysozyme-like genes (*LYZL2, LYZL4, LYZL6*, and *SPACA3*) are expressed predominantly in the testes, it has been suggested that they might have a role in reproduction [13-15,17]. Such a role has been shown for *Lyzl4 *and *Spaca3 *in mice [18,19].

The identification of these *LYZL *genes in the human genome suggests that the mammalian lysozyme-like gene family is larger than previously appreciated, and raises the possibility that the lysozyme-like proteins encoded by these genes may have novel biological functions. Here we have used extensive similarity searches of the human and other vertebrate genomes. We thereby identified three additional intact lysozyme-like genes in the human genome; these have been annotated in the databases, but not reported in the literature. We have also identified multiple lysozyme-like genes in the genomes of diverse vertebrates. Using a combination of phylogenetic and genomic neighborhood (or synteny) analyses, wherein the relationships of the genes that flank the lysozyme-like genes in diverse species were examined, we demonstrate that orthologs of the human lysozyme-like genes are found in the genomes of diverse mammalian species. Our analyses suggest that there were at least six, and perhaps as many as nine, diverse types (or subfamilies) of lysozyme-like genes in the genome of the common ancestor of all extant mammals, and that these diverse genes have been maintained on most mammalian lineages. This suggests that their protein products probably have essential biological functions that are yet to be identified.

## Results and Discussion

### Number of Lysozyme Genes in the Human Genome

**Figure 1:**
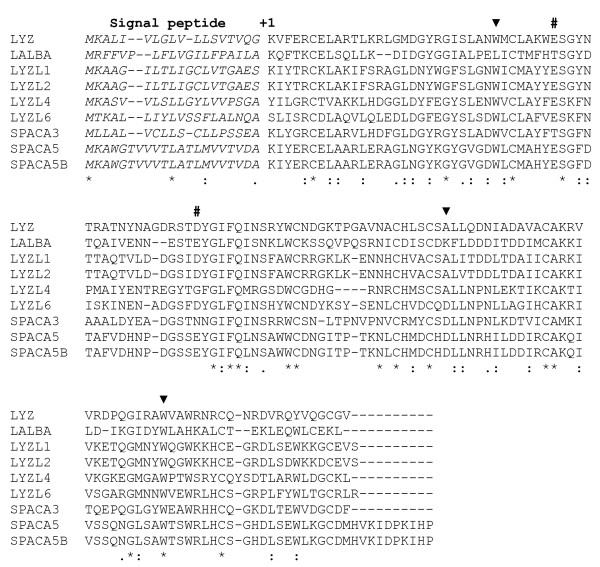
**Alignment of human lysozyme-like proteins**. Amino acid sequences of predicted human lysozyme-like proteins are aligned. The number +1 identifies the N-terminal amino acid of the mature protein and the signal peptides are shown in italics, based upon homology with lysozyme and lactalbumin. The number symbols (#) identify the active site residues (positions Glu-35 and Asp-52 of the mature protein sequence) of lysozyme *c*. The solid black triangles above the sequences indicate the positions in the coding sequence that are interrupted by introns in the genes. Asterisks below the sequences identify residues that are perfectly conserved among the sequences, while the symbols ":" and "." indicate conserved and semi-conserved residues, respectively.

To determine the size of the lysozyme-like gene family, we performed *BLAST *[20] similarity searches of the human genome for sequences that predict potential protein sequences similar to lysozyme *c*, and thereby identified a total of nine annotated genes (Table 1, and Additional file 1: Table S1). Of these nine annotated genes, six had previously been characterized: lysozyme *c *(*LYZ*) [21], lactalbumin (*LALBA*, Synonym: *LYZL7*) [22], *LYZL2, LYZL4, LYZL6 *[15], and *SPACA3 *(Synonyms: *LYZL3, SPRSA, SLLP1*) [13,14]. The three remaining genes identified in our *BLAST *searches -- the *LYZL1, SPACA5 *(Synonym: *LYZL5*), and *SPACA5B *genes -- had been annotated as lysozyme-like in *Ensembl *[16], but have not been discussed in the literature. A tenth lysozyme-like sequence was later identified using our genomic neighborhood analysis (see below), but appears to be a pseudogene (*ψLYSC1*). These ten lysozyme-like sequences are distributed over five chromosomes in humans, with two genes each on chromosomes 10, 17, and X, three genes on chromosome 12, and one gene on chromosome 3 (Table 1). Each of the potentially functional genes predicts a protein sequence about 140-150 amino acids long, similar to lysozyme *c *and lactalbumin. The mature regions of these proteins are readily aligned due to the presence of many highly conserved residues, including the eight cysteines known to be involved in disulfide bonds in lysozyme and lactalbumin (Figure 1). Each of the lysozyme-like genes has been annotated as being composed of four or five exons. The 140-150 amino acid coding regions are spread over four exons in each of the genes with the introns in exactly the same locations, including phases, as found in the lysozyme *c *and lactalbumin genes [5,6] (Figure 1). Moreover, the *LYZL1, LYZL2, LYZL4, LYZL6*, and *SPACA3 *genes are annotated as having an additional 5' exon, which in some cases might be translated to produce proteins that have longer N-terminal regions (especially in the case of *SPACA3*; not shown in Figure 1).

**Table 1:** Chromosomal location of human lysozyme-like genes

Gene	Chromosome^a^	Strand^a^	Position^a^	Intact^b^	Protein ID^c^
*LYZ*	12	+	69,742,134 -69,748,013	Y	ENSP00000261267
*LALBA*	12	-	48,961,468 -48,963,829	Y	ENSP00000301046
*ψLYSC1*	12	+	49,024,938 - 49,026,186	N	None (pseudogene)
*LYZL1*	10	+	29,577,990 - 29,607,257	Y	ENSP00000364650
*LYZL2*	10	-	30,895,152 - 30,918,691	Y	ENSP00000364467
*LYZL4*	3	-	42,438,570 - 42,452,092	Y	ENSP00000287748
*LYZL6*	17	-	34,261,548 - 34,270,674	Y	ENSP00000293274
*SPACA3*	17	+	31,318,887 - 31,324,895	Y	ENSP00000269053
*SPACA5*	X	+	47,863,734 - 47,869,126	Y	ENSP00000366139
*SPACA5B*	X	+	47,986,603 - 47,991,995	Y	ENSP00000304762

^a ^- Chromosomal localization, strand, and coordinates from release 57 of the human genome available in the *Ensembl *database (http://www.ensembl.org[16])

^b ^- Y, full-length protein sequence; N, no intact open reading frame

^c ^- *Ensembl *release 57 protein ID, None means no *Ensembl *Protein ID is available

Most of the predicted human proteins (Figure 1) show between 30% and 53% amino acid sequence identity in pairwise comparisons (Table 2), suggesting that the gene duplications that gave rise to them are fairly ancient (or, alternatively, that these proteins have evolved at extremely rapid rates). If these gene duplications were indeed ancient, then we would expect to find these genes in diverse mammalian species (as we did; see below). In contrast, the LYZL1/LYZL2 and SPACA5/SPACA5B protein pairs are 97% and 100% identical, respectively (Table 2); this suggests that their genes likely duplicated fairly recently, and thus these duplicates are predicted to be more limited phylogenetically. The *LYZL1 *and *LYZL2 *genes are both located on human chromosome 10, but are separated by about 1 Mb; moreover, these genes are embedded within 60 kb long repeated sequences that are greater than 95% identical (not shown). This suggests that the *LYZL1 *and *LYZL2 *gene duplicates were generated as part of a recent segmental duplication on chromosome 10. Likewise, the *SPACA5 *and *SPACA5B *genes are both on the X chromosome, separated by about 120 kb, and are within long (~100 kb) repeated DNA sequences that have high sequence identity (not shown). Thus, the *LYZL1/LYZL2 *and *SPACA5/SPACA5B *gene pairs both appear to have originated from relatively recent and large genomic segmental duplications, a common form of gene duplication in mammals [23]. The high sequence identity of these gene pairs also could be due, at least in part, to concerted evolution [24], as discussed below.

**Table 2:** Pairwise identity, in percent, of human lysozyme-like protein sequences

	LALBA	LYZL1	LYZL2	LYZL4	LYZL6	SPACA3	SPACA5	SPACA5B
LYZ	37	49	49	41	45	53	44	44
LALBA		36	36	30	32	33	32	32
LYZL1			97	43	43	47	47	47
LYZL2				43	43	46	47	47
LYZL4					46	47	40	40
LYZL6						45	47	47
SPACA3							49	49
SPACA5								100

### Lysozyme Genes in Other Vertebrate Genomes

**Figure 2:**
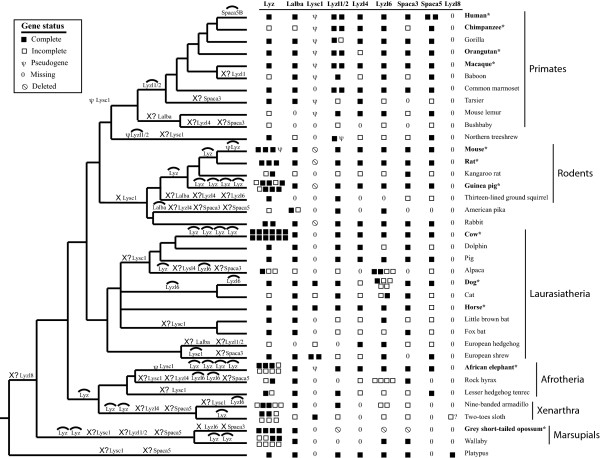
**Diversity and evolution of mammalian lysozyme-like genes**. A phylogeny, adapted from recent phylogenomic analyses [71-73], of the mammalian species examined in this study is shown; branch lengths are not proportional to evolutionary time. The numbers of each type of lysozyme-like gene identified in the mammalian genomes are shown by symbols on the right of the Figure. Species with genomes of higher quality (better coverage and assembly) are labeled in bold and with asterisks. The status of the genes in each genome are indicated by symbols, listed in the key box in the figure, where solid boxes indicate genes that are predicted to encode full-length sequences, open boxes indicate incomplete genes, which may be due to either incomplete genome sequences or may be pseudogenes, the Greek letter psi identifies well-characterized pseudogenes, zero indicates that no gene was identified in our searches, and the 'not' sign indicates genes that appear to have been deleted from the genomes. The question mark beside the sloth *Lyzl8 *gene indicates that this is a tentative assignment. Inferred evolutionary events are mapped onto their most likely lineage, based upon the data presented in this figure, coupled with our various evolutionary analyses. Duplication of genes is indicated by a gene name with an arc over it. Gene deletion is indicated by an X preceding the name of the deleted gene. X? means that the deletion is uncertain, as the missing gene may actually exist in a gap in the descendant genome(s). Pseudogene generation is indicated by the psi (ψ) that precedes the gene name; if the pseudogene is associated with a gene duplication event it also has an arc. If multiple events are shown on a lineage, the order shown does not imply historical order of occurrence.

To determine whether an expanded lysozyme-like gene family is a general feature of mammalian (and other vertebrate) genomes, we conducted intensive homology searches for lysozyme-like genes in all vertebrate genomes available in the *Ensembl *and *Pre!Ensembl *databases (http://www.ensembl.org, http://pre.ensembl.org/index.html) [16,25]. These results are listed in Supplementary Table 1 (Additional file 1: Table S1), and summarized for mammals in Figure 2. At least one lysozyme-like gene was found in each vertebrate genome, with the exception of the lamprey. Multiple lysozyme-like genes were identified in all of the mammalian species ranging from 5 in opossum to 18 in cow. The fewest number of genes were found in bony fish, where only one gene was identified per genome, and in birds, where at most two genes were identified per genome. The anole lizard and *Xenopus tropicalis*, the lone representatives of reptiles and amphibians with sequenced genomes, had 8 and 16 lysozyme-like genes, respectively.

Importantly, the *BLAST *searches identified potential orthologs in most mammalian genomes (Figure 2) of all of the divergent lysozyme-like genes found in the human genome (*LYZ, LALB, LYZL1*/*2, LYZL4, LYZL6, SPACA3, *and *SPACA5*). Initial assignments of orthology of these mammalian genes were based upon sequence similarity, but were subsequently confirmed by performing genomic neighborhood and phylogenetic analyses (see below). Our combined findings about gene number and orthologous relationships of the mammalian lysozyme-like gene family members are outlined in Figure 2. In contrast to the mammalian genes, the lysozyme-like genes found in the other vertebrate genomes could not be readily classified into the above subfamilies based upon sequence similarities; this is because these genes and their encoded proteins displayed similar levels of sequence identity to all of the different mammalian paralogs. Furthermore, no evidence of synteny with the mammalian genes was found for any of the non-mammalian vertebrate genes, except for the lysozyme *c *gene. Thus, none of the non-mammalian vertebrate lysozyme-like genes could be definitively classified as orthologs of any of the lysozyme-like mammalian genes, other than *Lyz *itself.

Many of the genes that were identified in our searches were only partial sequences, most likely due to the incomplete nature of the genomes in question. However, all but one of these genes were consistent with a structure similar to that of the mammalian lysozyme and lactalbumin genes -- that is, their coding regions appeared to be composed of four exons having similar intron-exon structures [5,6]. The lone exception was a *Lyzl1/2*-like gene found in the treeshrew (from Genescaffold_6044), which had a nearly full-length coding sequence that contained stop codons and frameshifts, but no introns; thus, this gene appears to be a processed pseudogene. Taken together, these observations suggest that essentially all of the vertebrate lysozyme-like genes have been generated by duplications of genomic DNA, rather than by reverse-transcription and insertion into genome.

### Phylogeny of Vertebrate Lysozymes

**Figure 3:**
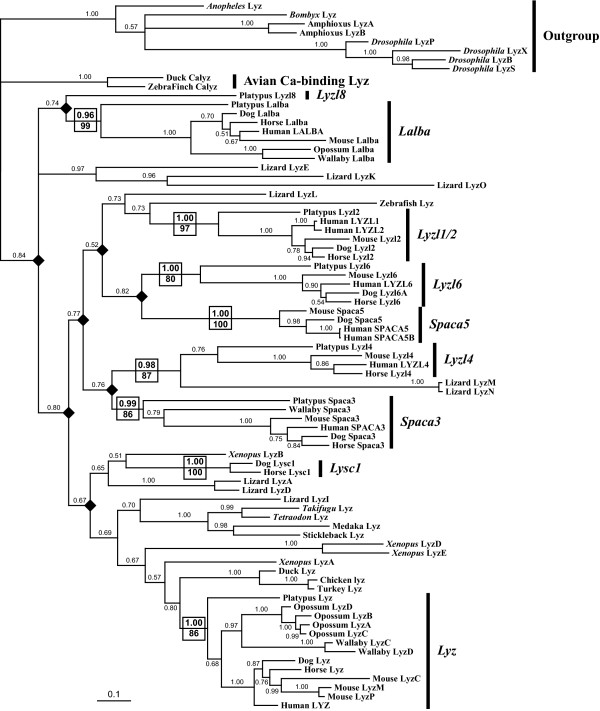
**Phylogeny of vertebrate lysozyme-like genes**. Bayesian phylogenetic tree of vertebrate lysozyme genes. The tree shown was generated with *MrBayes *[60,61] using DNA sequences of diverse vertebrate lysozyme-like sequences. (For sequences see Additional files 1 and 18: Tables S1 and S2 for sequences.) The alignment used to generate the tree is shown in Additional file 19: Figure S17. The trees were built after 2,000,000 generations with nst = 6 and rates = gamma (the TrNef+I+G model was selected as the best model by ModelTest [66-68]). Insect and amphioxus sequences were used to root the tree. Solid diamonds indicate nodes that represent the gene duplication events that generated the different types of lysozyme-like genes, or subfamilies, found in mammals. The support for each clade containing each subfamily of mammalian lysozyme-like gene is boxed, with posterior probabilities above the lineage and bootstrap support from maximum likelihood analysis below (from Additional file 3: Figure S2). The remaining lineages only display the posterior probabilities from the Bayesian analysis.

The presence of multiple lysozyme-like genes in all mammalian genomes, as well as in the genomes of several other vertebrate species, raises the possibility that the lysozyme-like gene family may have amplified early in vertebrate evolution. To examine this issue, and to further establish the orthology-paralogy relationships of lysozyme-like genes, we conducted a series of phylogenetic analyses (Figure 3, and Additional files 2-4: Figure S1-S3).

Importantly, as illustrated by the Bayesian analysis shown in Figure 3, all of these phylogenetic analyses suggested that most lineages of mammals have eight different types of lysozyme-like genes (or pseudogenes): *Lyz, Lalba, Lysc1, Lyzl1/2, Lyzl4, Lyzl6, Spaca3*, and *Spaca5*. Regardless of type of phylogenetic analysis, these mammalian genes always clustered together as monophyletic groups, or clades, supporting their orthologous relationships. These mammalian gene clades routinely had high statistical support, again regardless of method used. These results are consistent with the orthologous relationships suggested by the original *BLAST *searches, as well as with our genomic neighborhood analyses (described in more detail in the sections below). However, the genes from the other vertebrates did not consistently group with any of these mammalian orthologs, with the exception of some of the lysozyme *c *sequences.

In addition, it is clear that most, if not all, of the eight mammalian lysozyme-like genes duplicated and diverged from each other prior to the divergence of the earliest mammalian lineages. This is clear for at least two reasons. First, both the platypus and the eutherian genomes contain copies of most of these gene duplicates; therefore, these genes must have diverged earlier than did the species lineages. Second, some of the non-mammalian vertebrate genes appear to have phylogenetic affinity for some of the mammalian gene lineages, although few have much statistical support. This is particularly evident for many of the lizard genes which, as illustrated in Figure 3, tend to branch with various mammalian orthologs. If this result is not an artifact, then many of the lysozyme-like genes must have duplicated prior to the mammal-reptile (or even mammal-amphibian) divergence. If this were the case, however, then these gene duplicates must have been deleted from the genomes of birds.

Whereas our phylogenetic analyses supported the monophyly of each of the mammalian lysozyme-like gene duplicates, the relationships between the paralogs were not resolved well (see Figure 3, and Additional files 2-4: Figures S1-S3). While many of our phylogenetic analyses, including the one shown in Figure 3 (and Additional file 3: Figure S2), suggested that the *Lalba *clade was the earliest diverging lineage and that most of the *Lyzl *and *Spaca *genes (*Lyzl1/2, Lyzl4, Lyzl6, Spaca3*, and *Spaca5*, but not *Lyzl8*) were most closely related to each other, these relationships were not consistently found (*e.g*., see Additional file 2: Figure S1). Therefore, the phylogenetic analyses are inconclusive concerning the relationships of the different subfamilies of mammalian lysozyme-like genes.

The phylogenetic trees also suggested the possibility that at least some of the gene divergences occurred very early in vertebrate evolution, *i.e*., prior to the mammal-fish divergence. For example, the mammalian *Lyz *gene sequences were found to branch with *Lyz *genes from fish, rather than with the other mammalian lysozyme-like genes (Figure 3); if this branching order reflects the actual evolutionary history of the genes (rather than phylogenetic affinity based upon conserved lysozyme protein structure and function), then the *Lyz *gene lineage must have diverged from the other lysozyme-like genes prior to the mammal-fish divergence. Again, if this were true, then many species lineages must have deleted the duplicates from their genomes. Below, we discuss each subfamily of lysozyme-like gene in the mammals, and consider their potential non-mammalian orthologs.

### Lysozyme *c *(*Lyz*) Genes

**Figure 4:**
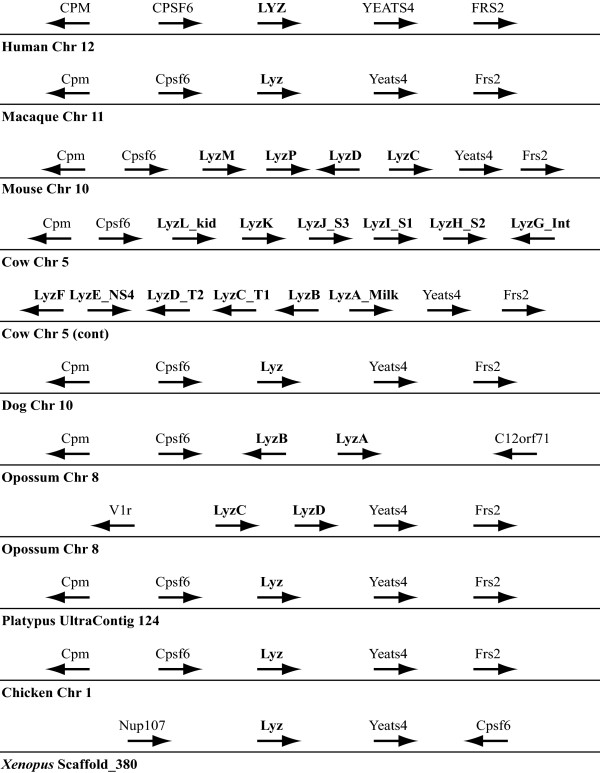
**Genomic neighborhood surrounding the lysozyme (*Lyz*) gene**. The relative organization and orientation (with arrowheads indicating the direction of transcription) of genes near the *Lyz *genes in representative diverse vertebrate genomes. Species and chromosomes (or contigs or scaffolds) are from *Ensembl *[16], and are shown under each gene array. Gene sizes and distances between genes are not to scale. The distance between the human *CFPS6 *and *YEATS4 *genes is about 90 kb. Gene symbols are: *CPM*, Carboxypeptidase M; *CPSF6*, Cleavage and polyadenylation specificity factor subunit 6; *YEATS4*, YEATS domain-containing protein 4 (Synonym: *Gas41*, Glioma-amplified sequence 41); *FRS2*, Fibroblast growth factor receptor substrate 2 (FGFR substrate 2); *C12orf71*, Chromosome 12 open reading frame 71; *V1r*, a member of the family of vomeronasal receptor gene family; *Nup107*, nucleoprotein 107 kDa. By necessity, the cow *Lyz *genes are, shown on two lines, but are actually contiguous in the genome. The opossum *Lyz *genes are at two non-adjacent locations on chromosome 8.

The *Lyz *gene is the best-studied lysozyme-like gene, and has been extensively characterized in many species [1-6]. Our genome searches and phylogenetic analyses identified many genes that appear to be orthologous to *Lyz *in diverse vertebrates (Figure 3, and Additional files 1-3: Table S1, and Figures S1 and S2). To confirm the orthology of the *Lyz *genes, we used a genomic neighborhood analysis, wherein we examined the orthology of the genes that flank the *Lyz *gene in diverse species. The avian and mammalian *Lyz *genes are flanked by the *Cpsf6 *and *Yeats4 *genes (Figure 4). This organization is maintained in species with tandemly duplicated *Lyz *genes, such as rodents and cow, where the *Cpsf6 *and *Yeats4 *genes are found flanking a cluster of *Lyz *genes (Figure 4). A slightly different organization is seen in the opossum, which has four *Lyz *genes; in this case the *Cpsf6 *gene is upstream of two of the *Lyz *genes and the *Yeats4 *gene is downstream of the other two, but these two clusters are separated by about 12 Mb of DNA that potentially was inserted into the opossum genome (Figure 4). Of the numerous lysozyme genes found in *Xenopus tropicalis*, the *LyzA *gene was found to be most closely related to the avian and mammalian *Lyz *genes in the phylogenetic trees (Figure 3); consistent with this, the *LyzA *gene is located adjacent to a *Yeats4 *ortholog (Figure 4), suggesting it is a true ortholog of the mammalian *Lyz *gene. Most fish *Lyz *sequences branch in a clade with mammalian *Lyz *genes (Figure 3). However, although the *Cpsf6 *and *Yeats4 *genes are neighbors in the fish genomes, the fish lysozyme genes are not adjacent to either of these genes; thus the genomic neighborhood analysis does not provide support for the orthology of fish and mammalian *Lyz *genes. Intriguingly, the zebrafish *Lyz *gene was found to be in a different genomic context from other fish *Lyz *genes (results not shown), which would agree with the hypothesis generated by the phylogenetic analyses (see Figure 3, and Additional files 2 and 3: Figures S1 and S2) that the zebrafish *Lyz *gene is not truly orthologous to the other fish *Lyz *genes.

**Figure 5:**
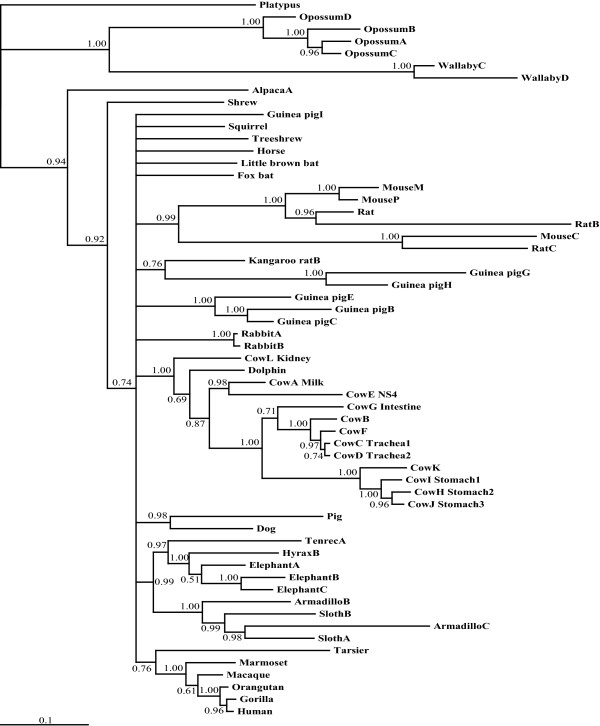
**Phylogeny of mammalian lysozyme *c *(*Lyz*) genes**. A Bayesian phylogenetic tree of mammalian lysozyme *c *genes was generated by *MrBayes *[60,61] using the DNA coding sequences of mammalian *Lyz *sequences. This tree was built with nst = 2 and rates = gamma as selected by *ModelTest *[66-68]. The tree was rooted with the platypus *Lyz *sequence. The posterior probability support for each node is shown.

The number of *Lyz *genes found in mammalian genomes varied from 1, in most species, to 12 (Figure 2, and Additional file 1: Table S1). Multiple *Lyz *genes had previously been identified in the genomes of mice and rats [26-29], rabbits [30,31], and artiodactyls [32-36]. In addition to these species, multiple *Lyz *genes were found in many other species, including guinea pig (9 genes), elephant (8 genes), armadillo (5 genes), sloth (6 genes), opossum (4 genes), and wallaby (8 genes) (Figure 2, and Additional file 1: Table S1). Phylogenetic analysis of the *Lyz *sequences, suggested that the multiple genes in diverse species are due to independent gene duplications or amplification events (Figure 5). However, previous work has shown that *Lyz *genes have been subjected to concerted evolution on the ruminant and rodent lineages [28,29,32-37]. Since the inference of independent gene duplication on sister lineages, instead of on their common ancestral lineage, is a pattern generated by concerted evolution, the distributions of duplicated *Lyz *genes (Figure 2, and Additional file 1: Table S1) combined with the phylogenetic analysis (Figure 5) suggests that concerted evolution might also have occurred on lineages such as Afrotheria (*e.g*., elephant and hyrax) and marsupials (opossum and wallaby).

### Lysozyme-like 1/2 (*Lyzl1/2*) Genes

**Figure 6:**
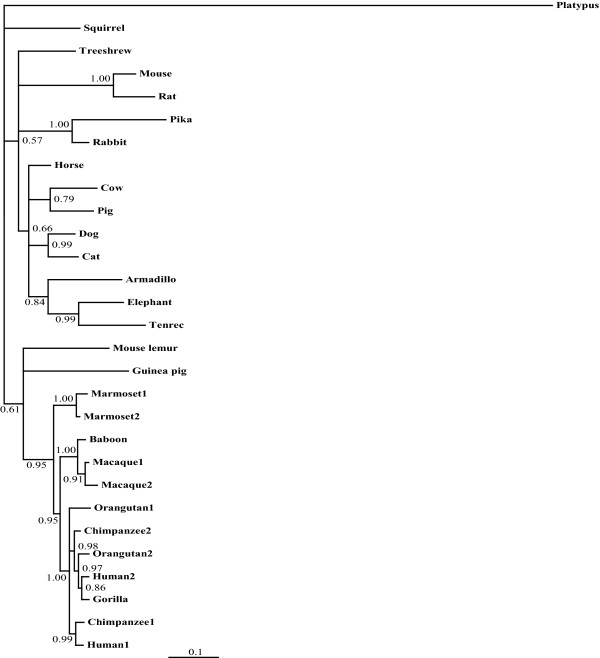
**Phylogeny of mammalian lysozyme-like 1/2 (*Lyz1/2*) genes**. A Bayesian phylogenetic tree of mammalian lysozyme-like 1 and 2 genes was generated by *MrBayes *[60,61] using the DNA coding sequences of mammalian *Lyzl1/2 *sequences. This tree was built with nst = 6 and rates = gamma as selected by *ModelTest *[66-68]. The tree was rooted with the platypus *Lyzl2 *sequence. The posterior probability support for each node is shown.

As mentioned above, the *Lyzl1 *and *Lyzl2 *genes in the human genome appear to have been generated recently via a genomic segmental duplication. Indeed, with the exception of those primate species that are close relatives of human, most other mammals have only a single gene sequence similar to *Lyzl1 *or *Lyzl2 *(Figure 2, and Additional file 1: Table S1). Comparison of the genomic neighborhoods surrounding the *Lyzl1/2 *genes in diverse mammals demonstrates orthology of these genes, as the genes adjacent to them are either *Bambi *or *Dnm1p17*, or both (Additional file 5: Figure S4). Interestingly, although an ortholog of *Lyzl1/2 *was found in the platypus genome, one could not be found in the opossum, even though the *Bambi *and *Dnm1p17 *genes are adjacent in this species; this suggests that the *Lyzl1/2 *gene was deleted from the opossum genome (Figure 2, and Additional file 5: Figure S4). Both human and macaque have duplicated *Lyzl1/2 *genes that reside in similar genomic neighborhoods (Additional file 5: Figure S4), which should imply that the *Lyzl1/2 *gene duplication occurred prior to the human-macaque divergence. Phylogenetic analysis of the *Lyzl1/2 *sequences, however, implies that independent gene duplication events occurred on the macaque, human, and marmoset lineages (Figure 6). A more likely scenario than multiple independent gene duplications in these closely-related primate species is that the original *Lyzl1/2 *gene duplication event occurred in their common ancestor (as diagrammed in Figure 2), and concerted evolution between the *Lyzl1 *and *Lyzl2 *genes has obscured this original event. Although a *Lyzl1/2 *pseudogene was found in the treeshrew (Figure 2, and Additional file 1: Table S1), it was generated independently, as discussed above. Potential *Lyzl1/2 *orthologs in zebrafish and lizard were suggested by phylogenetic analysis (Figure 3); however, these genes were not in genomic neighborhoods similar to those of the mammalian genes (results not shown), so their evolutionary relationships remain ambiguous.

### Lysozyme-like 4 (*Lyzl4*) Genes

The *Lyzl4 *gene was either found as a single copy, or was missing, in all of the mammalian genomes examined (Figure 2, and Additional file 1: Table S1). Many of the missing genes likely reflect incomplete genomes rather than deletions. Genomic neighborhood analysis confirmed the orthology of the *Lyzl4 *genes across placental mammals. However, the flanking genes are on different chromosomes in opossum, suggesting that chromosomal recombination had occurred (Additional file 6: Figure S5). Phylogenetic analyses of *Lyzl4 *sequences were consistent with it being a single copy gene (Additional file 7: Figure S6). The only potential non-mammalian orthologs of *Lyzl4 *identified by the phylogenetic analyses were from the lizard (*LyzM *and *LyzN *in Figure 3, but no evidence for orthology in Additional files 2-4: Figures S1-S3); however, the lizard and mammalian genes were not in similar genomic neighborhoods (data not shown).

### Lysozyme-like 6 (*Lyzl6*) Genes

Most mammals exhibited only one *Lyzl6 *gene, although the opossum had none. Yet, in contrast to most of the other paralogs, great variation in the number of *Lyzl6 *genes was observed across mammals, with five genes identified in the dog and four genes identified in both the alpaca and the hyrax (Figure 2, and Additional file 1: Table S1). Given the distant relationships of these three species, these gene duplication events must have occurred independently. The placental and wallaby *Lyzl6 *genes reside in a conserved genomic neighborhood, which again suggests that this gene was deleted on the opossum lineage (Additional file 8: Figure S7). The presence of multiple *Lyzl6 *genes in a genome raises the possibility of concerted evolution; however, sufficient data were not available to allow examination of this possibility for the *Lyzl6 *genes (Figure 2, and Additional files 1 and 9: Table S1 and Figure S8). The phylogenetic analyses did not suggest any candidates for *Lyzl6 *orthologs in non-mammalian species (Figure 3, and Additional files 2-4: Figures S1-S3).

### Sperm acrosomal protein 3 (*Spaca3*) Genes

*Spaca3*, like *Lyzl4*, was not found to be duplicated in any of the mammalian genomes examined (Figure 2, and Additional file 1: Table S1). *Spaca3 *resides in a conserved genomic neighborhood in placental mammals; however, a *Spaca3 *gene is absent from this genomic neighborhood in the opossum (Additional file 10: Figure S9). While the wallaby and platypus *Spaca3 *genes could not be placed in a genomic context due to the short lengths of their genomic contigs (Additional file 10: Figure S9), phylogenetic analysis of these sequences (Additional file 11: Figure S110 was consistent with them being orthologs. These results suggest that the *Spaca3 *gene was deleted on the opossum lineage. Again, no non-mammalian orthologs were suggested by phylogenetic analysis (Figure 3, and Additional files 2-4: Figures S1-S3).

### Sperm acrosomal protein 5 (*Spaca5*) Genes

The *Spaca5 *gene was found only within placental mammals, with no orthologs suggested by phylogenetic analysis or similarity searches in marsupials, platypus, or other vertebrates (Figures 2 and 3, and Additional files 1-4: Table S1 and Figures S1-S3). Thus, it is possible that this gene duplication happened in the ancestor of placental mammals. Genomic neighborhood analysis showed that the *Spaca5 *gene was in a similar neighborhood on the human, macaque, mouse, and dog X chromosomes (Additional file 12: Figure S11); this genomic region was not found in marsupials, platypus, or other vertebrates (results not shown). The *SPACA5 *gene was found to be uniquely duplicated in the human genome (Figure 2, Additional files 1 and 13: Table S1 and Figure S12). A very recent duplication of *SPACA5*, since human-chimpanzee divergence, could account for the perfect identity of the protein sequences (Table 2) without requiring concerted evolution; however, concerted evolution between the human *SPACA5 *and *SPACA5B *genes cannot be excluded.

### Lysozyme-like 8 (*Lyzl8*) Gene

The platypus genome contained one lysozyme-like gene, named *Lyzl8*, which did not group with any of the other mammalian genes (Figure 3, and Additional files 2-4: Figures S1-S3). All of our phylogenetic analyses supported the designation of *Lyzl8 *as a unique lysozyme-like gene duplicate, as the platypus gene did not fall within any of the other monophyletic gene groups. The relationship of the platypus *Lyzl8 *gene to the other lysozyme-like genes was highly labile in the phylogenetic analyses (Figure 3, and Additional files 2 and 3: Figures S1 and S2). This result is in accord with the fact that the platypus *Lyzl8 *gene (or protein) showed little similarity to any of the other lysozyme-like genes (or proteins) in our *BLAST *searches. When the platypus *Lyzl8 *gene was used as a query to search mammalian genomes, only one genomic sequence -- from the sloth (Figure 2, and Additional file 1: Table S1) -- was found to have greater similarity to *Lyzl8 *than to any other lysozyme-like gene. When the short sloth sequence was used as a query against the platypus genome, its best match was the *Lyzl8 *gene. However, the sloth genomic contig was short, containing only a single exon, and therefore could not be used for phylogenetic or genomic neighborhood analysis; thus, the evidence supporting orthology of the sloth sequence to the platypus *Lyzl8 *gene is very weak. Thus, at present, it is not clear whether this gene duplication happened on the ancestral mammal lineage, with subsequent losses on most descendant lineages, or on the monotreme lineage.

### Lactalbumin (*Lalba*) and Calcium-binding Lysozyme (*Lysc1*) Genes

**Figure 7:**
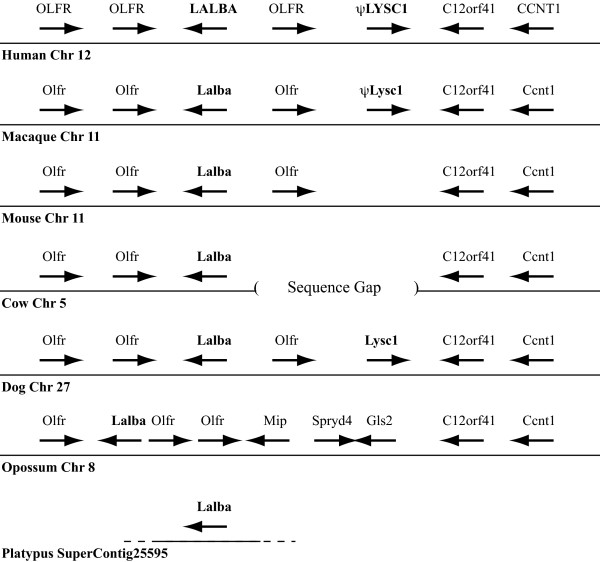
**Genomic neighborhood surrounding the lactalbumin (*Lalba*) and calcium-binding lysozyme (*Lysc1*) genes**. The relative organization and orientation (arrowheads indicated direction of transcription) of genes near the *Lalba *and *Lysc1 *genes in representative genomes from *Ensembl *[16]. Species and chromosome (or SuperContig for platypus) are indicated below each gene array. Gene sizes and distances are not to scale. The distance between the human *LALBA *and *CCNT1 *genes is about 150 kb. Gene symbols are: *OLFR*, a member of the Olfactory receptor gene family; *C12orf41*, chromosome 12 open reading frame 41; *CCNT1*, Cyclin-T1 (CycT1, Cyclin-T); *Mip*, major intrinsic protein of lens fiber; *Spryd4*, SPRY domain containing 4; *Gls2*, glutaminase 2. A large sequence gap exists in the cow genome, indicated by the parentheses, near the expected location of the *Lysc1 *gene. The platypus *Lalba *gene is on a small contig, as indicated by the shorter line flanked by dotted lines, that has not been annotated to contain (nor do we find) any other genes. Genes in the horse genome (not shown) are organized similar to those shown for the dog. Genes in the chimpanzee, gorilla, orangutan, baboon, and elephant genomes (not shown) are similar to those of the human and macaque. Genes in the rat and guinea pig genomes (not shown) are similar to those of the mouse.

Mammalian *Lalba *genes have been well characterized, and are typically single copy in mammals [1,2,4,6,7] (Additional file 14: Figure S13). Curiously, it was previously reported that multiple *Lalba *genes exist in the bovine and ovine genomes [38,39], but here we found only a single copy of the *Lalba *gene in the cow genome (Figure 2, and Additional file 1: Table S1). Whether this reflects differences in the sources of DNA in the different studies or is due to incomplete genome assembly is unknown. The only genome that revealed a duplicate *Lalba *gene was the pika (Figure 2, and Additional file 1: Table S1). Despite hypotheses about an early origin of the *Lalba *gene [10-12], no good candidates for non-mammalian orthologs were identified by our phylogenetic (Figure 3, and Additional file 2-4: Figures S1-S3) or genomic neighborhood (Figure 7, and results not shown) analyses.

An intriguing observation from our genomic neighborhood analysis of was that the mammalian calcium-binding lysozyme gene (*Lysc1*) is located adjacent to the *Lalba *gene in the dog (Figure 7) and horse (not shown) genomes. Both previous phylogenetic analyses [9-12] and our new phylogenetic analyses (Figure 2, and Additional files 2-4: Figures S1-S3) suggested that the *Lysc1 *gene originated prior to the radiation of mammals. However, our *tBLASTn *searches using either dog or horse *Lysc1 *identified similar sequences in the genomes of only a few diverse mammals -- dog, cat, horse, shrew, sloth, and mouse lemur (Figure 2, and Additional file 1: Table S1). It is also noteworthy that the mammalian (*Lysc1*) and avian calcium-binding lysozyme genes are not closely related in our phylogenies, a finding in agreement with some earlier analyses [3,11]. Thus, it is reasonable to speculate that calcium binding evolved independently in these bird and mammal lysozymes. The newly identified *Lysc1*-like genomic sequences all were found on short genomic contigs (Additional file 1: Table S1); nonetheless, both the cat and mouse lemur genomic contigs also encode part of the *c12orf41 *gene (Additional file 15: Figure S14A), which is adjacent to the *Lysc1 *gene in both the dog and horse genomes (Figure 7). This suggests that the *Lysc1 *gene may be near the *c12orf41 *gene in other mammalian genomes. Using a strategy that has previously worked to identify genes that could not be found through typical *BLAST *searches [40,41], we focused carefully on the sequences between the *Lalba *and *c12orf41 *genes. In 17 of the 37 mammalian genomes available from *Ensembl *[16,25], the *Lalba *and *c12orf41 *genes were contained in contiguous genomic sequences. In 18 of the 20 species this genomic region was fragmented into several small genomic contigs; thus, we cannot exclude the possibility that in these genomes the two genes are contiguous. In the pig and the little brown bat this genomic region was not fragmented. In the pig, the current genome assembly does not encode the *Lalba *gene and the *c12orf41 *gene is embedded within a very large genomic fragment, suggesting that the *Lalba *- *c12orf41 *genomic region has been reorganized in the pig genome (or that this region has been incorrectly assembled). In the little brown bat, the *Lalba *gene is embedded in a large genomic fragment that was not annotated to include *c12orf41 *(although our *BLAST *searches did identify a very small fragment with strong similarity). A more careful examination of the little brown bat genomic contig revealed that most of the genomic region is composed of unsequenced gaps.

For the 17 genomes that did have linked *Lalba *and *c12orf41 *genes, the distance between these two genes ranged from ~50 kb (mouse, rat, and rabbit) to ~250 kb (cow and opossum). For all of these genomes, except the opossum (see below), the only genes (or pseudogenes) annotated as existing between *Lalba *and *c12orf41 *were olfactory receptor-like genes, which are not very useful for identifying orthologous and conserved genomic neighborhoods due to their abundance. In the opossum, in addition to the olfactory receptor-like genes, three additional genes were annotated between *Lalba *and *c12orf41*: the genes *Mip, Spryd4*, and *Gls2*. Unfortunately, the wallaby genome is poorly assembled near the *Lalba *and *c12orf41 *genes, and thus the neighboring genes could not be identified. Although the *Mip, Spryd4*, and *Gls2 *genes reside on the same chromosome as *Lalba *and *c12orf41 *in many mammals (*e.g., *human, rat, guinea pig, cow, horse, and elephant), they are found greater than 8 Mb away; furthermore, in some species (*e.g*., mouse and dog) they are on different chromosomes. These observations suggest that the organization of the *Lalba, c12orf41, Mip, Spryd4*, and *Gls2 *genes, and potentially a *Lysc1 *gene, has changed between the opossum (and possibly other marsupials) and placental mammals.

The genomic sequence between the *Lalba *and *c12orf41 *genes for the 17 genomes where these two genes were linked was aligned with *MultiPipMaker *[42,43]. Sequences with similarity to the *Lysc1 *gene were not observed in 9 of the genomic sequences -- those from marmoset, mouse, rat, guinea pig, rabbit, treeshrew, cow, little brown bat, and opossum (Additional file 15: Figure S14B). It should be noted, however, that for 3 of these species (cow, little brown bat, and marmoset) these genomic sequences contain large amounts of unknown sequence (*i.e*., sequence gaps). Thus, there are only 6 species with nearly complete genomic sequences spanning the *Lalba *and *c12orf41 *genes for which we have good evidence for the actual absence of a *Lysc1 *gene or pseudogene -- mouse, rat, guinea pig, rabbit, treeshrew, and opossum. Pairwise sequence alignments between the mouse, rat, or guinea pig genomic sequences with those from dog or horse (or primates) using *PipMaker *[42] revealed that a large genomic region, which could potentially encode a *Lysc1 *gene, is missing from these rodent genomes (results not shown). This suggests that this genomic region, including the *Lysc1 *gene, was deleted either early on the rodent lineage or in the common ancestor of rodents and close relatives (*e.g., *rabbit), but after the divergence of the rodent lineage from the primate lineage (see Figure 2).

**Figure 8:**
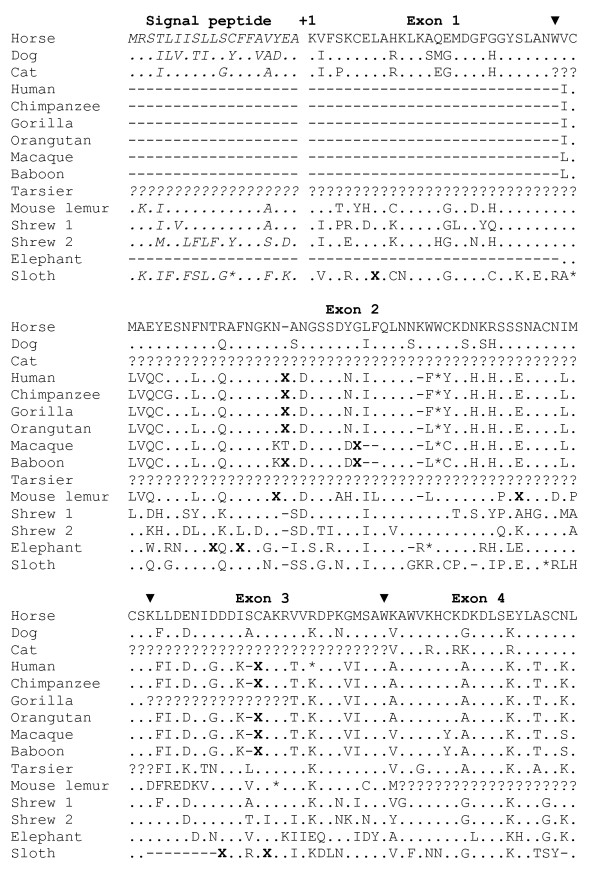
**Alignment of predicted calcium-binding lysozymes (*Lysc1*)**. Inferred amino acid sequences of predicted *Lysc1 *genes for diverse mammals are shown in the single-letter amino acid code. The DNA sequences are shown in Additional file 16: Figure S15. The number +1 identifies the N-terminal residue of the mature protein, and the signal peptides are shown in italics. The solid black triangles above the sequence indicate locations of the introns in the gene, with the exon number shown above the protein sequence. Dashes (-) identify gaps introduced to maximize alignment and refer to the absence of homologous sequence. Questions marks (?) indicate gaps introduced to maximize alignment, but are also potential sequence that may exist in sequence gaps in the genome assemblies (= missing data). Codons that have one or two base deletions, and thus would have a frame shift, are marked by an **X**. Asterisks identify in-frame stop codons in the sequences.

Interestingly, some of the genomes -- including those from certain haplorrhine primate species (human, chimpanzee, gorilla, orangutan, macaque, baboon, and tarsier) and the elephant -- do possess sequences between the *Lalba *and *c12orf41 *genes that aligned with three of the four exons (exons 2 through 4) of the horse and dog *Lysc1 *genes (Figures 7 and 8, and Additional files 15 and 16: Figures S14C and S15). All of these genomic sequences, except for that of the tarsier, are on large genomic segments that have only a few short unsequenced gaps. It is unlikely that all of these genomic sequences have been similarly misassembled, thus we conclude that exon 1 was deleted from all of these genes, and therefore the *Lysc1 *gene is a pseudogene in all of these species (Figure 2). In addition to missing exon 1, all of these *Lysc1*-like gene sequences have both frameshift insertions and/or deletions and in-frame stop codons, strengthening the conclusion that they are pseudogenes (Figure 8, and Additional file 16: Figure S15). The loss of exon 1 from the *Lysc1 *gene of haplorrhine primates and elephant must have been independent events (Figure 2) as the mouse lemur, a strepsirrhine primate, has a *Lysc1 *gene that has an intact exon 1 (Figure 8, and Additional file 16: Figure S15), plus the primates and elephants are quite distant relatives.

Intact *Lysc1 *genes that predict potentially functional calcium-binding lysozymes were found in only a few species (dog, horse, and shrew), whereas pseudogenes were found on several lineages (primates, elephant, and sloth). Phylogenetic and genomic analyses suggested that the pair of *Lysc1 *genes found in the shrew resulted from a tandem gene duplication event on the lineage leading to this species (Additional files 1 and 17: Table S1 and Figure S16); the divergence of the predicted protein sequences of the two genes suggests that they are not undergoing concerted evolution, however. The *Lysc1 *gene was deleted from the genome on the lineages leading to rodents (mouse, rat, guinea pig, and squirrel) and treeshrews. Taken together, the above observations suggest that the *Lysc1 *gene likely arose from a duplication of the lactalbumin gene early in mammalian evolution, and was inactivated several times independently, as summarized in Figure 2.

## Conclusions

Here we have shown that the mammalian lysozyme gene family is much larger than previously anticipated, and is composed of at least eight distantly-related members (*Lyz, Lalba, Lysc1, Lyzl1/2, Lyzl4, Lyzl6, Spaca3*, and *Spaca5*) in most mammalian species. These observations suggest that this family experienced several duplication events prior to the origin of mammals. Several other gene families also experienced such amplifications near the origin of mammals, such as those generating the gene families for keratin-associated proteins [44], kallikriens [45,46], and bitter taste receptors [47]. Amplification of these latter genes has been suggested to be associated with development of new mammal-specific features -- *e.g*., hair (keratin-associated proteins), skin (kallikriens), and diet (bitter taste receptors) [44-47]. Intriguingly, lactalbumin is essential for lactose synthesis in mammary glands, a mammal-specific trait [2,4,7]. These observations raise the possibility that other members of the lysozyme-like family have also evolved mammal-specific roles. The new lysozyme-like genes have been largely conserved within mammals, suggesting that they provide important biological functions. The products of the *Spaca3 *and *Lyzl4 *genes have recently been shown to be involved in fertilization in mice [18,19]. Much further study is needed to identify the enzymatic activities (if any) and biological functions of these newly identified lysozyme-like proteins.

Similar to the keratin-associated protein [44] and bitter taste receptor [47] gene families, genes for the lysozyme-like proteins are dispersed over several chromosomes (Table 1). The mechanisms by which these original gene duplications occurred are unclear, as the genes that flank the dispersed lysozyme-like genes show no homology to each other, implying that they were not generated by large segmental duplication events (as we observed for the duplications of *LYZL1/LYZL2 *and *SPACA5/SPACA5B *in the human genome). The lysozyme-like gene family also shares with the keratin associated protein [44], kallikrein [45], and bitter taste receptor [47] gene families the propensity for lineage-specific gene duplications (see Figures 2 and 3). The lineage-specific expansions, in contrast to the initial duplications, have frequently been tandem in nature. Such tandem organization increases the likelihood that the duplicated genes could be involved in concerted evolution [22,23], which our phylogenetic analyses suggest have occurred in the *Lyz *and *Lyzl1/2 *subfamilies. The *Lyz *subfamily showed the greatest tendency to tandemly duplicate and evolve in concert, whereas the other lysozyme-like genes typically showed conservation in copy number. Tandem duplication or amplification of the *Lyz *gene has previously been observed in certain mammals, including the ruminants and rodents, where lysozyme appears to function as a digestive enzyme in the gut [3,25-34]. It is of interest to note that many of the species that we found to possess multiple *Lyz *genes -- *e.g., *elephant and wallaby -- are also herbivorous species, and thus may use lysozyme as a digestive enzyme upon gut bacteria. The need for higher levels of digestive lysozymes in the guts of fermenting herbivores could have driven the fixation of the tandem duplications in these lineages. Gene conversion between the tandem duplicates might then provide a mechanism whereby favorable mutations in one gene copy could spread to the other copies in the cluster [33,36], as well as a mechanism for retention of sequence similarity [24] in well-adapted proteins.

## Methods

### Database Searches

All vertebrate genomes maintained in the *Ensembl *[16] and *Pre!Ensembl *[25] databases (release 57, see Additional file 1: Table S1 for a full list) were searched in April 2010 for lysozyme-like sequences. We initially searched the genomes using the *tBLASTn *algorithm [20,48] using previously-characterized human and rodent lysozyme *c *and lactalbumin sequences. Subsequent *tBLASTn *searches used all of the identified putative lysozyme-like protein sequences. Similar searches were conducted using additional databases (*e.g*., genome assemblies and ESTs) available at the NCBI website [49]. After identification of the dog and horse calcium-binding lysozyme gene, the other mammalian genome assemblies on the *Ensembl *database were searched using these sequences using *tBLASTn *for similar sequences. All sequences that had E-scores below 0.01 were examined. Sequences identified by *BLAST *searches were used in reciprocal *BLASTx *searches of the human, mouse and dog proteomes to ensure that their best matches were lysozyme-like sequences. Sequences that were unannotated to encode lysozyme-like sequences (see Additional file 1: Table S1) were examined to identify potential coding sequences using published methods [50-52]. Insect and amphioxus lysozyme sequences, used as outgroups for the phylogenetic analysis (see below), were identified by searches of the NCBI ENTREZ protein database [49] for *Drosophila *[53] and amphioxus [54] lysozymes; these protein sequences were then used in *tBLASTn *[20] searches of the *Ensembl *[16] and NCBI databases [49] for related sequences. Several insect sequences were downloaded to represent the diversity of insect lysozyme sequences.

Genomic comparisons of DNA sequences near the lysozyme-like genes were conducted using *PipMaker *and *MultiPipMaker *[42,43,55]. Genes neighboring the lysozyme-like genes were identified from the genome assemblies at *Ensembl *[16] and *Pre!Ensembl *[25]. The organization of genes adjacent to the lysozyme-like genes was used to determine whether the genes of interest reside in conserved genomic neighborhoods.

### Phylogenetic Analysis

Phylogenies of vertebrate lysozyme-like gene coding sequences were generated with sequences from human, mouse, dog, horse, opossum, wallaby, and platypus, representing the diversity of mammals, as well as those from other vertebrate species (see Additional file 1: Table S1) and outgroups (Additional file 18: Table S2). Lysozyme-like coding sequences were aligned using *MAFFT *[56] and *Clustal *[57], as implemented at the *Guidance *web site [58,59], using default parameters. (A *MAFFT *alignment of all the full-length sequences is provided in Additional file 19: Figure S17). Protein sequences were used as guides to generate the DNA sequence alignments. The reliability of the alignments was examined using *Guidance *[58,59] and trimmed alignments using sites that had values above the default cut-off of 0.93 were generated. Insect and/or amphioxus lysozyme sequences were used to root the trees of vertebrate lysozyme-like sequences.

Phylogenetic trees of the sequences were generated by a variety of methods including *MrBayes *3.1.2 [60,61], *PhyloBayes *3.2f [62], and *PhyML *[63], *MEGA*4.0.2 [64], and PAUP* 4beta10 [65]. Bayesian trees were generated from coding sequences with *MrBayes *3.1.2 using parameters selected by hierarchical likelihood ratio tests with *ModelTest *version 3.8, as implemented on the ModelTest server [66-68]. *MrBayes *was run for 2,000,000 generations with four simultaneous Metropolis-coupled Monte Carlo Markov chains sampled every 100 generations. The average standard deviation of split frequencies dropped to less than 0.02 for all analyses. The first 25% of the trees were discarded as burn-in with the remaining samples used to generate the consensus trees. Trace files generated by *MrBayes *were examined by *Tracer *[69] to verify if they had converged. Bayesian phylogenies were also generated from protein sequences using *PhyloBayes*, with two chains being used with the automatic stopping rule set to terminate the analysis when *bpcomp *and *tracecomp *indicated that discrepancies between the chains was equal to or below 0.2 and all effective sizes were greater than 100. The first 10% of the trees were discarded as burnin. PAUP* was used to construct parsimony trees. Bootstrapped maximum likelihood trees, 100 replications, were generated by *PhyML *[63] on the *PhyML *webserver [70] using parameters for the substitution model suggested by *ModelTest*. The maximum likelihood search was initiated from a tree generated by *BIONJ *and the best tree was identified after heuristic searches using the nearest neighbor interchange (NNI) algorithm. *MEGA4 *[64] was used to construct bootstrapped (1000 replications) neighbor-joining distance trees, using either Maximum Composite Likelihood distances for the DNA sequences or JTT distances for the proteins sequences. Bootstrapped parsimony trees were also generated by *PAUP *[65], with 1000 replications and the same search method used for maximum likelihood.

With respect to orthology-paralogy issues, the choice of outgroup, the alignment method (*MAFFT *[56] or *Clustal *[57]), and the use of full-length or trimmed (based on *Guidance *scores [58,59]) alignments had little influence on the key findings of these analyses. Methods that relied on shorter sequences (*i.e*., trimmed alignments or protein sequences) or simpler models of sequence evolution (*i.e*., neighbor-joining or parsimony) tended to yield weaker support for the earlier diverging lineages, but none of our analyses were in significant conflict with the key inferences of the phylogeny presented in Figure 3.

For phylogenies that contained just mammalian lysozyme-like sequences, *Lalba *sequences were arbitrarily used to root the trees. When only mammalian lysozyme-like gene sequences were used for the phylogenetic analyses, then stronger support for each of the orthologous groups was found with all of the phylogenetic methods used including Bayesian inference, maximum likelihood, distance, and parsimony (see Additional file 4: Figure S3). To generate gene-specific phylogenies, the platypus sequence was used as a root, except for *Lysc1 *and *Spaca5 *where the platypus does not have these sequences. For *Lysc1*, the sloth sequence was used to root the tree, whereas for *Spaca5 *the elephant and tenrec sequences provided the root.

## Authors' contributions

DMI and CBS together designed the research and outlined the manuscript. DMI, JMB, and CBS obtained and analyzed the data. DMI drafted the manuscript. All of the authors have read, edited, and approved the final manuscript.

## Supplementary Material

Additional file 1 **Supplementary Table 1**. This file is in PDF format. Location of lysozyme genes in vertebrate genomes. Click here for file

Additional file 2 **Supplementary Figure 1**. This file is in PDF format. Phylogeny of vertebrate lysozyme-like sequences generated by *PhyloBayes*. Click here for file

Additional file 3 **Supplementary Figure 2**. This file is in PDF format. Phylogeny of vertebrate lysozyme-like sequences generated by *PhyML*. Click here for file

Additional file 4 **Supplementary Figure 3**. This file is in PDF format. Phylogeny of only mammalian lysozyme-like sequences generated by *MrBayes *with support for the orthologous genes by different phylogenetic methods. Click here for file

Additional file 5 **Supplementary Figure 4**. This file is in PDF format. Conservation of genomic organization near *Lyzl1/2 *genes. Click here for file

Additional file 6 **Supplementary Figure 5**. This file is in PDF format. Conservation of genomic organization near *Lyzl4 *genes. Click here for file

Additional file 7 **Supplementary Figure 6**. This file is in PDF format. Phylogeny of *Lyzl4 *genes. Click here for file

Additional file 8 **Supplementary Figure 7**. This file is in PDF format. Conservation of genomic organization near *Lyzl6 *genes. Click here for file

Additional file 9 **Supplementary Figure 8**. This file is in PDF format. Phylogeny of *Lyzl6 *genes. Click here for file

Additional file 10 **Supplementary Figure 9**. This file is in PDF format. Conservation of genomic organization near *Spaca3 *genes. Click here for file

Additional file 11 **Supplementary Figure 10**. This file is in PDF format. Phylogeny of *Spaca3 *genes. Click here for file

Additional file 12 **Supplementary Figure 11**. This file is in PDF format. Conservation of genomic organization near *Spaca5 *genes. Click here for file

Additional file 13 **Supplementary Figure 12**. This file is in PDF format. Phylogeny of *Spaca5 *genes. Click here for file

Additional file 14 **Supplementary Figure 13**. This file is in PDF format. Phylogeny of *Lalba *genes. Click here for file

Additional file 15 **Supplementary Figure 14**. This file is in PDF format. Conservation of genomic sequences between *Lalba *and *c12orf41 *genes. Click here for file

Additional file 16 **Supplementary Figure 15**. This file is in PDF format. DNA sequences of *Lysc1 *genes. Click here for file

Additional file 17 **Supplementary Figure 16**. This file is in PDF format. Phylogeny of *Lysc1 *genes. Click here for file

Additional file 18 **Supplementary Table 2**. This file is in PDF format. Outgroup lysozyme sequences used for phylogenetic analysis. Click here for file

Additional file 19 **Supplementary Figure 17**. This file is in Word format. FASTA formatted *MAFFT *alignment of lysozyme DNA sequences Click here for file
