# Supplementary material for: Evolution of the mammalian lysozyme gene family
Source: BMC Evol Biol. 2011 Jun 15;11:166. doi: 10.1186/1471-2148-11-166 (PMC3141428; doi:10.1186/1471-2148-11-166)
Supplement: Additional file 1 — Supplementary Table 1. This file is in PDF format. Location of lysozyme genes in vertebrate genomes. [file 1471-2148-11-166-S1.PDF]

Supplementary Table 1. Location of lysozyme-like genes in mammalian genomes.

The genomic locations (chromosome/contig/scaffold, strand, and coordinates) of lysozyme-like genes that were identified by *Blast* searches of mammalian genomes available in *Ensembl* release 57 or *Pre.Ensembl* [16,25]. Whether the predicted sequences are full length and Protein ID (from *Ensembl*), or NCBI identifier (if an incomplete or no *Ensemble* ID is available), are indicated.

	Chromosome/contig[a]	strand[a]	position[a]	full-length[b]	Protein ID[c]
**Human** (*Homo sapiens*)					
LYZ	Chr 12	+	69,742,134-69,748,013	Y	ENSP00000261267
LALBA	Chr 12	-	48,961,468-48,963,829	Y	ENSP00000301046
LYSC1	Chr 12	+	49,024,938-49,026,186	Pseudo	-
LYZL1	Chr 10	+	29,577,990-29,607,257	Y	ENSP00000364650
LYZL2	Chr 10	-	30,895,152-30,918,691	Y	ENSP00000364467
LYZL4	Chr 3	-	42,438,570-42,452,092	Y	ENSP00000287748
LYZL6	Chr 17	-	34,261,548-34,270,674	Y	ENSP00000293274
SPACA3	Chr 17	+	31,318,887-31,324,895	Y	ENSP00000269053
SPACA5	Chr X	+	47,863,734-47,869,126	Y	ENSP00000366139
SPACA5B	Chr X	+	47,986,603-47,991,995	Y	ENSP00000304762
LYZL8	NO HIT[d]				-
**Chimpanzee** (*Pan troglodytes*)					
Lyz	Chr 12	+	69,669,876-69,673,755	Incomplete	ENSPTRP00000050073
Lalba	Chr 12	+	41,183,822-41,185,328	Incomplete	ENSPTRP00000009035
Lysc1	Chr 12	-	41,117,131-41,118,358	Pseudo	-
Lyzl1	Chr 10	+	29,984,329-30,007,563	Y	ENSPTRP00000050711
Lyzl2	Chr 10	-	31,341,939-31,359,879	Y	ENSPTRP00000004072
Lyzl4	Chr 3	-	43,366,037-43,379,513	Y	ENSPTRP00000025500
Lyzl6	Chr 17	+	21,220,666-21,225,648	Y	ENSPTRP00000015409
Spaca3	Chr 17	-	24,229,998-24,235,488	Incomplete	ENSPTRP00000015349
Spaca5	Chr X	+	48,325,010-48,329,894	Y	ENSPTRP00000037522
Lyzl8	NO HIT				-
**Gorilla** (*Gorilla gorilla*)					
Lyz	chr12	+	67,915,290-67,921,167	Y	ENSGGOP00000010835
Lalba	chr12	-	46,612,732-46,615,107	Y	ENSGGOP00000025397
Lysc1	chr12_47119396_665003	+	46,675,468-46,676,658	Incomplete	-
Lyzl1	chr10	-	33,961,416-33,982,440	Y	ENSGGOP00000025764
Lyzl2	chr10	+	33,978,384-33,979,216	Incomplete	ENSGGOP00000000745
Lyzl4	chr3	-	43,493,355-43,506,871	Y	ENSGGOP00000015037
Lyzl6	chr5	-	48,199,157-48,204,140	Y	ENSGGOP00000010201
Spaca3	chr5	+	51,245,333-51,251,344	Y	ENSGGOP00000001109
Spaca5	chrX	+	49,043,631-49,045,644	Y	ENSGGOP00000025571
Lyzl8	NO HIT				-

**<u>Orangutan</u>** (*Pongo pygmaeus*)					
Lyz	Chr 12	+	69,610,466-69,615,240	Y	ENSPPYP00000005419
Lalba	Chr 12	-	48,221,846-48,223,930	Y	ENSPPYP00000005095
Lysc1	Chr 12	+	48,283,291-48,284,522	Pseudo	-
Lyzl1	Chr 10	+	30,470,390-30,491,388	Y	ENSPPYP00000002539
Lyzl2	Chr 10	-	31,603,439-31,621,815	Y	ENSPPYP00000002546
Lyzl4	Chr 3	+	104,256,284-104,259,609	Incomplete	ENSPPYP00000015639
Lyzl6	Chr 17	-	30,720,560-30,725,061	Y	ENSPPYP00000009216
Spaca3	Chr 17	+	10,341,667-10,344,300	Y	ENSPPYP00000009945
Spaca5	Chr X	+	48,817,704-48,819,545	Y	ENSPPYP00000022725
Lyzl8	NO HIT				-
**<u>Macaque</u>** (*Macaca mulatta*)					
Lyz	Chr 11	+	66,342,375-66,347,986	Y	ENSMMUP00000011770
Lalba	Chr 11	-	45,692,915-45,695,265	Y	ENSMMUP00000000385
Lysc1	Chr 11	+	45,753,683-45,754,901	Pseudo	-
Lyzl1	Chr 9	+	29,429,118-29,450,851	Y	ENSMMUP00000036072
Lyzl2	Chr 9	-	30,788,164-30,808,180	Y	ENSMMUP00000036053
Lyzl4	Chr 2	+	94,182,315-94,191,138	Y	ENSMMUP00000023139
Lyzl6	Chr 16	-	31,215,047-31,219,931	Y	ENSMMUP00000009005
Spaca3	Chr 16	+	28,309,200-28,315,336	Y	ENSMMUP00000014973
Spaca5	Chr X	+	45,824,139-45,829,262	Y	ENSMMUP00000014266
Lyzl8	NO HIT				-
**<u>Baboon</u>** (*Papio hamadryas*)					
Lyz	Contig570200_Contig299388	-	272,406-272,693	Incomplete	-
Lalba	Contig453675_Contig296390	+	20,897-22,942	Y	ENSP00000301046_1
Lysc1	Contig313296_Contig312315	+	41,139-42,356	Pseudo	-
Lyzl1/2	Contig559024_Contig443093	-	41,669-63,587	Y	ENSP00000364650_1
Lyzl4	Contig391086_Contig615552	-	215,380-218,706	Incomplete	-
Lyzl6	Contig2096_Contig696651	-	11,317-15,994	Y	ENSP00000378031_1
Spaca3	Contig330087_Contig330086	+	23-2,152	Incomplete	-
Spaca5	Contig359723_Contig766466	-	16,334-18,121	Y	ENSP00000305847_1
Lyzl8	NO HIT				-
**<u>Marmoset</u>** (*Callithrix jacchus*)					
Lyz	SuperContig96	-	7,376,942-7,382,056	Y	ENSCJAP00000003201
Lalba	SuperContig268	-	294,587-296,965	Y	ENSCJAP00000038560
Lysc1	NO HIT				-
Lyzl1	SuperContig99	+	1,804,869-1,826,854	Y	ENSCJAP00000024228
Lyzl2	SuperContig99	-	2,890,304-2,899,786	Y	ENSCJAP00000024432
Lyzl4	SuperContig273	-	2,084,032-2,094,826	Y	ENSCJAP00000033280

Lyzl6	SuperContig57	-	10,370,917-10,380,987	Y	ENSCJAP00000027724
Spaca3	SuperContig57	+	7,390,332-7,396,896	Y	ENSCJAP00000026803
Spaca5	SuperContig793	-	82,860-88,074	Y	ENSCJAP00000028737
Lyzl8	NO HIT				-
**<u>Tarsier</u>** (*Tarsius syrichta*)					
Lyz	GeneScaffold_1103	+	13,960-17,194	Y	ENSTSYP00000011435
Lalba	Scaffold_37335	+	1,749-3,886	Y[d]	ENSTSYP00000006674
Lysc1	Scaffold_232175	-	416-885	Pseudo	-
Lyzl1/2	GeneScaffold_2783	+	202-8,343	Incomplete	ENSTSYP00000002381
Lyzl4	GeneScaffold_5308	-	713-12,962	Y	ENSTSYP00000012479
Lyzl6	GeneScaffold_7654	-	12,541-15,037	Incomplete	ENSTSYP00000012944
Spaca3	NO HIT				-
Spaca5	Scaffold_59756	+	259-605	Incomplete	-
Lyzl8	NO HIT				-
**<u>Mouse lemur</u>** (*Microcebus murinus*)					
Lyz	Scaffold_5043	+	11,336-15,291	Incomplete	ENSMICP00000002246
Lalba	NO HIT				-
Lysc1	GeneScaffold_2182	+	32,175-35,039	Incomplete	-
Lyzl1/2	GeneScaffold_1461	+	28,171-51,571	Y	ENSMICP00000010674
Lyzl4	GeneScaffold_657	-	526,838-536,724	Y	ENSMICP00000000285
Lyzl6	Scaffold_27961	-	2,273-5,786	Y	ENSMICP00000006251
Spaca3	GeneScaffold_1063	+	540,966-545,612	Incomplete	ENSMICP00000010833
Spaca5	Scaffold_10046	+	30,958-33,322	Y	ENSMICP00000004140
Lyzl8	NO HIT				-
**<u>Bushbaby</u>** (*Otolemur garnettii*)					
Lyz	Scaffold_86558	+	44,394-48,340	Incomplete	ENSOGAP00000004704
Lalba	NO HIT				-
Lysc1	NO HIT				-
Lyzl1/2	GeneScaffold_3643	+	4,572-5477	Incomplete	-
Lyzl4	NO HIT				-
Lyzl6	Scaffold_16374	-	680-2,885	Incomplete	ENSOGAP00000007622
Spaca3	NO HIT				-
Spaca5	Scaffold_17127	+	3,410-3,556	Incomplete	-
Lyzl8	NO HIT				-
**<u>Treeshrew</u>** (*Tupaia belangeri*)					
Lyz	Scaffold_116195	+	26,997-32,126	Y	ENSTBEP00000000453
Lalba	GeneScaffold_5557	-	57,447-58,177	Incomplete	ENSTBEP00000014313
Lysc1	NO HIT				-

Lyzl1/2	GeneScaffold_1790	+	275,843-322,708	Y	ENSTBEP00000003616
Lyzl1/2	Genescaffold_6044	-	117,775-118,127	Incomplete	processed Pseudogene
Lyzl4	GeneScaffold_4950	-	586,086-589,163	Incomplete	ENSTBEP00000005592
Lyzl6	Scaffold_50624	-	1-1,156	Incomplete	-
Spaca3	GeneScaffold_2806	+	34,451-62,252	Incomplete	ENSTBEP00000009723
Spaca5	Scaffold_148165	+	4,024-7,332	Y	ENSTBEP00000002460
Lyzl8	NO HIT				-
**<u>Mouse</u>** (*Mus musculus*)					
LyzM	Chr10	-	116,724,853-116,729,939	Y	ENSMUSP00000089800
LyzP	Chr10	-	116,714,390-116,719,343	Y	ENSMUSP00000089801
LyzC	Chr10	-	116,670,815-116,675,662	Y	ENSMUSP00000020392
LyzD	Chr10	+	116,685,029-116,687,277	Pseudo	-
Lalba	Chr15	-	98,310,831-98,313,114	Y	ENSMUSP00000023726
Lysc1	NO HIT				-
Lyzl1/2	Chr18	+	4,165,830-4,182,230	Y	ENSMUSP00000025076
Lyzl4	Chr9	-	121,486,961-121,551,214	Y	ENSMUSP00000076887
Lyzl6	Chr11	-	103,492,384-103,500,204	Y	ENSMUSP00000021328
Spaca3	Chr11	+	80,671,867-80,681,316	Y	ENSMUSP00000069612
Spaca5	ChrX	+	20,645,532-20,655,089	Y	ENSMUSP00000048721
Lyzl8	NO HIT				-
**<u>Rat</u>** (*Rattus norvegicus*)					
Lyz1	Chr7	-	56,607,709-56,613,053	Y	ENSRNOP00000007747
Lyz2	Chr7	+	56,587,878-56,594,629	Y	ENSRNOP00000047172
LyzC	Chr7	-	56,576,837-56,581,709	Y	ENSRNOP00000007610
Lalba	Chr7	-	137,220,699-137,223,614	Y	ENSRNOP00000014457
Lysc1	NO HIT				
Lyzl1/2	Chr17	-	62,058,344-62,071,768	Y	ENSRNOP00000021901
Lyzl4	Chr8	-	126,588,208-126,592,666	Y	ENSRNOP00000026173
Lyzl6	Chr10	-	92,787,372-92,792,857	Y	ENSRNOP00000004650
Spaca3	Chr10	+	69,087,498-69,090,699	Y	ENSRNOP00000009171
Spaca5	NO HIT			Y	NP_001101528
Lyzl8	NO HIT				-
**<u>Kangaroo rat</u>** (*Dipodomys ordii*)					
LyzA	Scaffold_17077	+	4,875-9,083	Incomplete	-
LyzB	Scaffold_17077	+	17,482-21,837	Y	ENSDORP00000004234
Lalba	Scaffold_34623	+	5,904-7,444	Incomplete	ENSDORP00000009249
Lysc1	NO HIT				-
Lyzl1/2	GeneScaffold_2235	+	4,539-24,563	Incomplete	ENSDORP00000015408
Lyzl4	Scaffold_166223	+	260-325	Incomplete	-
Lyzl6	Scaffold_25282	-	2,388-5,981	Incomplete	ENSDORP00000003574

Spaca3	GeneScaffold_3417	+	285-4,782	Y	ENSDORP00000014957
Spaca5	Scaffold_48457	+	3,897-4170	Incomplete	-
Lyzl8	NO HIT				-
**Guinea pig** (*Cavia porcellus*)					
LyzA	Scaffold_9	-	29,020,611-29,028,668	Incomplete	-
LyzB	Scaffold_9	-	29,045,453-29,050,823	Y	-
LyzC	Scaffold_9	-	29,092,689- 29,097,700	Y	-
LyzD	Scaffold_9	-	29,121,945-29,125,002	Incomplete	-
LyzE	Scaffold_9	-	29,141,105-29,146,834	Y	ENSCPOP00000001464
LyzF	Scaffold_9	-	29,162,335-29,165,255	Incomplete	-
LyzG	Scaffold_9	+	29,220,088-29,224,656	Y	ENSCPOP00000000667
LyzH	Scaffold_9	-	29,273,580-29,278,396	Y	-
LyzI	Scaffold_9	-	29,318,131-29,322,329	Y	-
Lalba	Scaffold_9	+	50,981,327-50,983,368	Y	ENSCPOP00000019835
Lysc1	NO HIT				-
Lyzl1/2	Scaffold_39	-	16,999,347-17,010,174	Y	ENSCPOP00000000236
Lyzl4	Scaffold_7	-	44,755,235-44,762,587	Y	ENSCPOP00000018998
Lyzl6	Scaffold_32	-	19,218,323-19,221,780	Y	ENSCPOP00000004538
Spaca3	Scaffold_32	-	13,840,901-13,846,589	Y	ENSCPOP00000016556
Spaca5	Scaffold_132	+	1,579,881-1,582,144	Y[e]	ENSCPOP00000010522
Lyzl8	NO HIT				-
**Squirrel** (*Spermophilus tridecemlineatus*)					
Lyz	GeneScaffold_1472	+	201,668-211,074	Y	ENSSTOP00000012663
Lalba	NO HIT				-
Lysc1	NO HIT				-
Lyzl1/2	GeneScaffold_1894	+	69,687-84,959	Y	ENSSTOP00000006136
Lyzl4	NO HIT				-
Lyzl6	NO HIT				-
Spaca3	GeneScaffold_45	+	91,432-95,598	Incomplete	ENSSTOP00000010720
Spaca5	Scaffold_16373	+	46,564-48,908	Incomplete	ENSSTOP00000005580
Lyzl8	NO HIT				
**Pika** (*Ochotona princeps*)					
Lyz	GeneScaffold_623	+	490-3,944	Incomplete	ENSOPRP00000003488
LalbaA	GeneScaffold_3794	-	14,175-17,516	Y	ENSOPRP00000011798
LalbaB	Scaffold_66832	-	3,735-5,393	Incomplete	ENSOPRP00000006887
Lysc1	NO HIT				-
Lyzl1/2	GeneScaffold_1570	+	58,076-78,553	Y	ENSOPRP00000007956
Lyzl4	NO HIT				-
Lyzl6	GeneScaffold_4410	+	4,821-28,684	Y	ENSOPRP00000010098
Spaca3	NO HIT				-

Spaca5	NO HIT				-
Lyzl8	NO HIT				-
**Rabbit** (*Oryctolagus cuniculus*)					
LyzA	Scaffold_18	-	35,620,599-35,626,277	Y	ENSOCUP00000009926
LyzB	Scaffold_18	-	35,578,828-35,585,187	Y	ENSOCUP00000011947
Lalba	Scaffold_145664	-	1,438-5,488	Y	ENSOCUP00000013596
Lysc1	NO HIT				-
Lyzl1/2	Scaffold_62	-	5,772,049-5,781,414	Y	ENSOCUP00000015233
Lyzl4	Scaffold_51	+	1,821,409-1,827,724	Y	ENSOCUP00000011274
Lyzl6	Scaffold_23	-	23,182,069-23,185,501	Y	ENSOCUP00000020841
Spaca3	Scaffold_23	+	10,738-14,550	Y	ENSOCUP00000009286
Spaca5	Scaffold_24	-	1,334,795-1,337,921	Y	ENSOCUP00000002597
Lyzl8	NO HIT				-
**Cow** (*Bos taurus*)					
LyzA_Milk	Chr5	-	47,609,298-47,615,293	Y	ENSBTAP00000015846
LyzB	Chr5	+	47,678,791-47,683,754	Y	ENSBTAP00000050453
LyzC_Tra1	Chr5	+	47,766,066-47,770,500	Y	ENSBTAP00000000231
LyzD_Tra2	Chr5	+	47,786,448-47,791,028	Y	ENSBTAP00000048332
LyzE_NS4X	Chr5	-	47,819,300-47,824,676	Y	ENSBTAP00000049668
LyzF	Chr5	+	47,838,680-47,843,614	Y	ENSBTAP00000049136
LyzG _Int	Chr5	+	47,887,127-47,892,714	Y	ENSBTAP00000027401
LyzH_S2	Chr5	-	47,905,648-47,911,397	Y	ENSBTAP00000007924
LyzI_S1	Chr5	-	47,932,130-47,939,885	Y	ENSBTAP00000004235
LyzJ_S3	Chr5	-	47,973,394-47,980,390	Y	ENSBTAP00000027399
LyzK	Chr5	-	47,994,902-48,018,682	Y[e]	ENSBTAP00000017351
LyzL_Kid	Chr5	-	48,056,542-48,065,378	Y	ENSBTAP00000038081
Lalba	Chr5	+	34,386,886-34,388,564	Y	ENSBTAP00000007701
Lysc1	NO HIT				-
Lyzl1/2	Chr13	-	35,402,323-35,418,340	Y	ENSBTAP00000013640
Lyzl4	Chr22	-	4,981,785-14,986,671	Y	ENSBTAP00000024756
Lyzl6	Chr19	-	46,690,507-46,698,057	Y	ENSBTAP00000000032
Spaca3	Chr19	-	17,016,505-17,020,569	Y[e]	ENSBTAP00000000449/DT839200.1
Spaca5	NO HIT			Y	DT847078.1
Lyzl8	NO HIT				-
**Dolphin** (*Tursiops truncatus*)					
Lyz	Scaffold_114746	+	182,136-187,936	Y	ENSTTRP00000013219
Lalba	GeneScaffold_2519	-	29,946-33,578	Y	ENSTTRP00000016184
Lysc1	NO HIT				-
Lyzl1/2	Scaffold_93758	+	41,190-52,144	Y[d]	ENSTTRP00000014089

Lyzl4	Scaffold_107303	-	4,332-10,089	Y	ENSTTRP00000005817
Lyzl6	Scaffold_84769	-	8,500-12,072	Incomplete	ENSTTRP00000005866
Spaca3	GeneScaffold_742	+	466,055-473,589	Y	ENSTTRP00000010623
Spaca5	GeneScaffold_2689	+	18,640-21,956	Incomplete	ENSTTRP00000005316
Lyzl8	NO HIT				-
**Aplaca** (*Vicugna pacos*)					
LyzA	GeneScaffold_447	+	17,798-53,056	Y	ENSVPAP00000008610
LyzB	Scaffold_11030	+	8,431-13,764	Incomplete	ENSVPAP00000004983
LyzC	Scaffold_3658	+	255-3,648	Incomplete	ENSVPAP00000010159
Lalba	Scaffold_7356	+	29,781-30,385	Incomplete	-
Lysc1	NO HIT				-
Lyzl1/2	GeneScaffold_483	+	483,304-490,932	Incomplete	ENSVPAP00000006029
Lyzl4	NO HIT				-
Lyzl6A	Scaffold_21099	-	1,215-4,770	Y	ENSVPAP00000003843
Lyzl6B	Scaffold_25066	-	1,732-5,307	Y	ENSVPAP00000002334
Lyzl6C	Scaffold_158358	-	75-1,186	Incomplete	ENSVPAP00000008182
Lyzl6D	Scaffold_8592	-	43,780-44,890	Incomplete	ENSVPAP00000007592
Spaca3	NO HIT				-
Spaca5	GeneScaffold_2767	+	45,086-46,668	Incomplete	ENSVPAP00000011076
Lyzl8	NO HIT				-
**Pig** (*Sus scrofa*)					
Lyz	Chr5	-	32,494,728-32,501,113	Y	ENSSSCP00000000522
Lalba	NO HIT			Y	NP_999525
Lysc1	NO HIT				-
Lyzl1/2	Chr10	+	38,800,719-38,816,348	Y	ENSSSCP00000011751
Lyzl4	Chr13	-	21,825,227-21,832,323	Y	ENSSSCP00000012025
Lyzl6	Chr12	+	15,719,424-15,726,694	Y	ENSSSCP00000018354
Spaca3	Chr12	-	39,640,598-39,648,712	Y	ENSSSCP00000018781
Spaca5	ChrX	+	42,444,219-42,446,313	Y	ENSSSCP00000013074
Lyzl8	NO HIT				-
**Dog** (*Canis familiaris*)					
Lyz	Chr10	+	14,330,496-14,334,946	Y	ENSCAFP00000000619
Lalba	Chr27	+	8,908,493-8,910,505	Y	ENSCAFP00000013149
Lysc1	Chr27	-	8,871,076-8,873,413	Y	ENSCAFP00000013141
Lyzl1/2	Chr2	-	19,805,709-19,819,124	Y	ENSCAFP00000005897
Lyzl4	Chr23	-	14,568,389-14,575,403	Incomplete	ENSCAFP00000035021
Lyzl6A	Chr9	+	13,349,476-13,353,820	Y	ENSCAFP00000019754/CX991013.1
Lyzl6B	Chr9	-	12,049,540-12,052,412	Incomplete	-
Lyzl6C	Chr9	-	21,328,591-21,331,407	Incomplete	-
Lyzl6D	Unknown	+	81,335,870-81,339,050	Incomplete	ENSCAFP00000035795

Lyzl6E	Unknown	-	56,808,101-56,809,225	Incomplete	-
Spaca3	Chr9	-	43,417,559-43,425,263	Y	ENSCAFP00000027123
Spaca5	ChrX	+	41,540,634-41,543,344	Y	ENSCAFP00000022555
Lyzl8	NO HIT				-
**Cat** (*Felis catus*)					
Lyz	GeneScaffold_630	+	86,315-126,378	Incomplete	ENSFCAP00000005029
Lalba	Scaffold_138738	-	1,490-3,488	Y	ENSFCAP00000010481
Lysc1	GeneScaffold_2359	+	37,417-39,195	Incomplete	-
Lyzl1/2	GeneScaffold_1561	-	108,904-123,524	Y	ENSFCAP00000004315
Lyzl4	Scaffold_167763	-	1,455-1,854	Incomplete	ENSFCAP00000008279
Lyzl6A	Scaffold_151594	-	1,065-3,557	Incomplete	ENSFCAP00000000035
Lyzl6B	Scaffold_3692	-	1,953-5,535	Y	ENSFCAP00000014610
Spaca3	GeneScaffold_46	+	95,012-99,101	Y	ENSFCAP00000000848
Spaca5	GeneScaffold_354	+	38,509-38,788	Incomplete	-
Lyzl8	NO HIT				-
**Horse** (*Equus caballus*)					
Lyz	Chr6	+	84,276,135-84,280,482	Y	ENSECAP00000015931
Lalba	Chr6	-	66,475,022-66,477,097	Y	ENSECAP00000001776
Lysc1	Chr6	+	66,522,809-66,525,494	Y	ENSECAP00000008172
Lyzl1/2	Chr29	-	7,935,911-7,948,922	Y	ENSECAP00000000207
Lyzl4	Chr16	+	43,681,812-43,691,829	Y	ENSECAP00000011383
Lyzl6	Chr11	+	18,250,920-18,254,412	Y	ENSECAP00000014352
Spaca3	Chr11	-	39,667,637-39,671,818	Y	ENSECAP00000004434
Spaca5	ChrX	+	38,988,302-38,990,682	Incomplete	ENSECAP00000019693
Lyzl8	NO HIT				-
**Microbat** (Little brown bat, *Myotis lucifugus*)					
Lyz	GeneScaffold_1421	+	169,750-173,322	Y	ENSMLUP00000003804
Lalba	GeneScaffold_5829	-	16,524-18,242	Y	ENSMLUP00000009932
Lysc1	NO HIT				-
Lyzl1/2	GeneScaffold_1855	+	174,251-194,627	Incomplete	ENSMLUP00000010372
Lyzl4	Scaffold_137119	+	3,564-3,971	Incomplete	ENSMLUP00000009358
Lyzl6	GeneScaffold_5946	+	3,415-6,796	Y	-
Spaca3	GeneScaffold_37	+	99,053-103,286	Incomplete	ENSMLUP00000001099
Spaca5	Scaffold_108973	-	3,595-2,778	Incomplete	-
Lyzl8	NO HIT				-
**Megabat** (Fox bat, *Pteropus vampyrus*)					
Lyz	GeneScaffold_521	+	30,537-34,396	Y	ENSPVAP00000000877
Lalba	GeneScaffold_3836	-	12,922-14,675	Y	ENSPVAP00000016929
Lysc1	NO HIT				-

Lyzl1/2	GeneScaffold_1191	+	23,062-34,164	Incomplete	ENSPVAP00000007292
Lyzl4	Scaffold_8866	-	24,676-32,556	Incomplete	-
Lyzl6	GeneScaffold_341	+	531,987-534,890	Incomplete	-
Spaca3	GeneScaffold_3231	+	304,021-307,739	Y	ENSPVAP00000014828
Spaca5	GeneScaffold_2091	+	42,696-45,081	Incomplete	ENSPVAP00000001668
Lyzl8	NO HIT				-
**<u>Hedgehog</u>** (*Erinaceus europaeus*)					
Lyz	Scaffold_278844	+	4,895-4,996	Incomplete	-
Lalba	NO HIT				-
Lysc1	Scaffold_224095	-	4,192-4,887	Incomplete	-
Lyzl1/2	NO HIT				-
Lyzl4	Scaffold_332621	+	6,634-13,823	Y[d]	ENSEEUP00000002515
Lyzl6	GeneScaffold_7555	-	755-47,672	Y	ENSEEUP00000010257
Spaca3	GeneScaffold_2043	+	37,766-52,621	Incomplete	ENSEEUP00000010696
Spaca5	Scaffold_218555	-	212-666	Incomplete	ENSEEUP00000005570
Lyzl8	NO HIT				-
**<u>Shrew</u>** (*Sorex araneus*)					
Lyz	Scaffold_230272	-	3,709-8,166	Y	ENSSARP00000005328
Lalba	Scaffold_221830	-	32,631-34,955	Y	ENSSARP00000010825
Lysc1A	Scaffold_236349	-	46,719-49,946	Y	-
Lysc1B	Scaffold_236349	-	1,936-5,695	Y	-
Lyzl1/2	GeneScaffold_2371	-	288,547-300,367	Incomplete	ENSSARP00000001339
Lyzl4	Scaffold_254066	-	38,939-43,040	Incomplete	-
Lyzl6	GeneScaffold_6567	-	483,674-675,701	Y	ENSSARP00000008115
Spaca3	NO HIT				-
Spaca5	Scaffold_230559	-	43,524-45,684	Y	ENSSARP00000007987
Lyzl8	NO HIT				-
**<u>Elephant</u>** (*Loxodonta africana*)					
LyzA	scaffold_2	+	59,614,117-59,618,033	Y	ENSLAFG00000000591
LyzB	scaffold_2	+	59,633,793-59,639,459	Y	ENSLAFG00000026760
LyzC	scaffold_2	+	59,660,084-59,664,714	Y	ENSLAFG00000006201
LyzD	scaffold_2	+	59,688,921–59,690,133	Incomplete	-
LyzE	scaffold_386	-	12,127-13,325	Incomplete	-
LyzF	scaffold_386	-	37,341-41,765	Incomplete	-
LyzG	scaffold_386	-	72,482-76,945	Incomplete	-
LyzH	scaffold_3	-	1,681,161-1,681,619	Incomplete	-
Lalba	scaffold_2	+	34,979,610-34,980,810	Y[f]	ENSLAFG00000014199
Lysc1	scaffold_2	+	35,030,039-35,031,483	Peudo	-
Lyzl1/2	scaffold_15	-	13,759,019-13,768,961	Y	ENSLAFG00000014906

Lyzl4	scaffold_12	+	24,998,877-25,002,582	Y[f]	ENSLAFG00000000649
Lyzl6	scaffold_31	-	26,646,888-26,650,310	Y	ENSLAFG00000029877
Spaca3	scaffold_31	+	2,417,144-2,422,135	Y	ENSLAFG00000028131
Spaca5	scaffold_56	-	2,140,113-2,142,602	Y	ENSLAFG00000009001
Lyzl8	NO HIT				-
**Hyrax** (*Procavia capensis*)					
LyzA	GeneScaffold_1012	+	12,774-15,642	Incomplete	ENSPCAP00000010775
LyzB	Scaffold_63832	+	2,457-6,655	Y	ENSPCAP00000003920
Lalba	Scaffold_35551	+	8,032-9,767	Y	ENSPCAP00000010016
Lysc1	NO HIT				-
Lyzl1/2	NO HIT				-
Lyzl4	GeneScaffold_4763	-	22,202-33,037	Incomplete	ENSPCAP00000006600
Lyzl6A	Scaffold_117632	-	5,411-8,454	Incomplete	ENSPCAP00000007508
Lyzl6B	Scaffold_46853	-	10,959-13,701	Incomplete	ENSPCAP00000013285
Lyzl6C	Scaffold_216402	-	684-851	Incomplete	-
Lyzl6D	Scaffold_265523	-	292-471	Incomplete	-
Spaca3	GeneScaffold_3760	+	22,384-27,864	Y	ENSPCAP00000015514
Spaca5	NO HIT				-
Lyzl8	NO HIT				-
**Lesser hedgehog tenrec** (*Echinops telfairi*)					
LyzA	GeneScaffold_1096 5' end	+	17,292-21,931	Y	ENSETEP00000012524
LyzB	GeneScaffold_1096 3' end	+	50,184-54,393	Incomplete	ENSETEP00000012525
Lalba	Scaffold_304215	+	1,016-4,532	Y	ENSETEP00000002456
Lysc1	NO HIT				-
Lyzl1/2	Scaffold_306400	-	207,370-217,648	Y	ENSETEP00000005802
Lyzl4	GeneScaffold_5432	-	586-5,721	Incomplete	ENSETEP00000001859
Lyzl6	GeneScaffold_5695	-	130-2,716	Incomplete	ENSETEP00000004328
Spaca3	GeneScaffold_4368	+	942-9,689	Incomplete	ENSETEP00000011348
Spaca5	Scaffold_244132	+	5,602-7,532	Y	ENSETEP00000014842
Lyzl8	NO HIT				-
**Armadillo** (*Dasypus novemcinctus*)					
LyzA	Scaffold_38871	+	10,296-13,915	Incomplete	ENSDNOP00000006895
LyzB	GeneScaffold_3972	+	5,056-17,916	Y	ENSDNOP00000000659
LyzC	Scaffold_93846	+	298-3,750	Y	ENSDNOP00000008463
LyzD	Scaffold_48888	-	5,10-5,258	Incomplete	-
LyzE	Scaffold_74767	-	8,139-8,359	Incomplete	-
Lalba	Scaffold_34167	+	38,320-40,217	Y	ENSDNOP00000014634
Lysc1	NO HIT				-
Lyzl1/2	GeneScaffold_2379	+	55,068-72,723	Y[d]	ENSDNOP00000012525
Lyzl4	NO HIT				-

Lyzl6A	GeneScaffold_6655	-	23,995-26,693	Incomplete	ENSDNOP00000004596
Lyzl6B	Scaffold_53955	-	6,486-8,852	Incomplete	-
Spaca3	GeneScaffold_3674	+	37,948-89,324	Incomplete	ENSDNOP00000004174
Spaca5	NO HIT				-
Lyzl8	NO HIT				-
**<u>Sloth</u>** (*Choloepus hoffmanni*)					
LyzA	GeneScaffold_1075	+	4,873-9,554	Y	ENSCHOP00000001798
LyzB	Scaffold_114763	+	1,082-4,103	Y	ENSCHOP00000004357
LyzC	Scaffold_247746	+	3,305-3,466	Incomplete	-
LyzD	Scaffold_269748	+	1,052-1,213	Incomplete	-
LyzE	Scaffold_389743	+	19-198	Incomplete	-
LyzF	Scaffold_361510	+	1,103-1,231	Incomplete	-
Lalba	Scaffold_99624	+	5,429-6,831	Incomplete	ENSCHOP00000000336
Lysc1	Scaffold_11723	-	14,308-16,545	Y	-
Lyzl1/2	GeneScaffold_2648	+	4,023-17,614	Incomplete	ENSCHOP00000006091
Lyzl4	NO HIT				-
Lyzl6	GeneScaffold_5313	-	5,006-7,254	Incomplete	ENSCHOP00000011584
Spaca3	Scaffold_10256	-	8,614-12,379	Incomplete	ENSCHOP00000011090
Spaca5	NO HIT				-
Lyzl8	Scaffold_55261	-	1,747-1,911	Incomplete	-
**<u>Opossum</u>** (*Monodelphis domestica*)					
LyzA	Chr8	-	26,289,116-26,297,126	Y	ENSMODP00000037594
LyzB	Chr8	+	26,424,282-26,440,587	Y	ENSMODP00000008733
LyzC	Chr8	+	36,555,471-36,563,676	Y	ENSMODP00000025482
LyzD	Chr8	+	36,620,811-36,626,060	Y	ENSMODP00000025484
Lalba	Chr8	-	31,135,425-31,137,796	Y	ENSMODP00000026437
Lysc1	NO HIT				-
Lyzl1/2	NO HIT				-
Lyzl4	NO HIT				-
Lyzl6	NO HIT				-
Spaca3	NO HIT				-
Spaca5	NO HIT				-
Lyzl8	NO HIT				-
**<u>Wallaby</u>** (*Macropus eugenii*)					
LyzA	Scaffold339490	-	1,333-2,104	Incomplete	ENSMEUP00000006159
LyzB	Scaffold23673	-	19,736-22,246	Incomplete	ENSMEUP00000014520
LyzC	Scaffold31416	-	8,887-14,579	Y	ENSMEUP00000010780
LyzD	Scaffold8611	-	22,422-28,826	Y[e]	ENSMEUP00000011237
LyzE	Scaffold8611	-	6,385-12,992	Incomplete	-
LyzF	Scaffold217224	-	477-1,258	Incomplete	ENSMEUP00000011660

LyzG	Scaffold269826	-	876-1.049	Incomplete	-
LyzH	Scaffold153024	+	8,572-9,297	Incomplete	-
Lalba	Scaffold140	-	5,758-8,126	Y	ENSMEUP00000005076 /AAA31602
Lysc1	NO HIT				-
Lyzl1/2	NO HIT				-
Lyzl4	NO HIT				-
Lyzl6	GeneScaffold9201	-	52,514-53,803	Y[e]	ENSMEUP00000014464
Spaca3	Scaffold5145	+	6,783-10,781	Y[e]	ENSMEUP00000013735
Spaca5	NO HIT				-
Lyzl8	NO HIT				-
**Platypus** (*Ornithorhynchus anatinus*)					
Lyz	Ultracontig124	+	1,819,564-1,826,204	Y	ENSOANP00000009465
Lalba	SuperContig25595	-	8,125-8,732	Y[f]	ENSOANP00000020722/ P30805.1
Lysc1	NO HIT				-
Lyzl1/2	UltraContig274	+	2,804,545-2,815,508	Y	ENSOANP00000009625
Lyzl4	UltraContig117	-	210,620-213,969	Y	ENSOANP00000005681
Lyzl6	Contig32352	+	4,491-10,705	Y	ENSOANP00000027472
Spaca3	SuperContig47148	-	991-3,970	Y	ENSOANP00000007614
Spaca5	NO HIT				-
Lyzl8	SuperContig2498	-	55,151-59,094	Y	ENSOANP00000023594
**Anole lizard** (*Anolis carolinensis*)					
LyzA	Scaffold_158	+	1,438,241-1,442,659	Y	ENSACAP00000006668
LyzB	Scaffold_158	-	841,352-846,778	Incomplete	ENSACAP00000006012
LyzC	Scaffold_245	+	417,275-425,365	Incomplete	ENSACAP00000013796
LyzD	Scaffold_553	-	416,342-425,116	Y	ENSACAP00000016383
LyzE	Scaffold_158	-	874,499-877203	Y	-
LyzF	Scaffold_158	-	934,359-937,158	Incomplete	-
LyzG	Scaffold_158	+	862,866-869,973	Incomplete	ENSACAP00000006070
LyzH	Scaffold_3299	-	4,906-7949	Incomplete	-
LyzI	Scaffold_245	-	404,750-408,994	Y	ENSACAP00000013790
LyzJ	Scaffold_158	-	888,734-892,451	Incomplete	-
LyzK	Scaffold_469	-	355,445-360,329	Y	-
LyzL	Scaffold_245	+	384,244-393,323	Y	ENSACAP00000013648
LyzM	Scaffold_469	+	367,650-375,866	Y	ENSACAP00000010877
LyzN	Scaffold_469	+	388,061-395,410	Y	ENSACAP00000010879
LyzO	Scaffold_469	+	175,926-193,750	Y	ENSACAP00000010862
LyzP	Scaffold_469	+	212,149-212,604	Incomplete	-
**Chicken** (*Gallus gallus*)					
Lyz	Chr1	+	37,298,007-37,301,735	Y	ENSGALP00000016177

Ca lzm	NO HIT				-
**Turkey** (*Meleagris gallopavo*)					
Lyz	Chr1	+	36,969,201-36,972,964	Y	ENSMGAP00000010985
Ca lzm	NO HIT				-
**Duck** (*Anas platyrhynchos*)					
Lyz	Scaffold14	-	265,585-269,139	Y	ENSAPLT00000013816
Ca lzm	Scaffold355	+	836-56,747	Y	ENSAPLP00000001891
**Zebra finch** (*Taeniopygia guttata*)					
Lyz	NO HIT				-
Ca lzm	NO HIT			Y	NCBI: ACH46437.1
Xenopus (*Xenopus tropicalis*)					
XlyzA	Scaffold_380	-	865,758-869,726	Y	ENSXETP00000056246
XlyzB	Scaffold_6903	-	106-2,553	Y	ENSXETP00000028567
XlyzC	Scaffold_165	-	1,317,614-1,323,021	Incomplete	-
XlyzD	Scaffold_165	-	1,327,495-1,331,781	Y	-
XlyzE	Scaffold_165	-	1,254,137-1,257,836	Y	ENSXETP00000040356
XlyzF	Scaffold_13869	-	2,741-5,449	Incomplete	-
XlyzG	Scaffold_165	+	1,293,660-1,295,475	Incomplete	-
XlysH	Scaffold_165	-	1,299,595-1,301,691	Incomplete	-
**Zebrafish** (*Danio rerio*)					
Lyz	Chr24	-	23,512,905-23,515,840	Y	ENSDARP00000074998
**Medaka** (*Oryzias latipes*)					
Lyz	Chr12	+	30,016,849-30,020,507	Y	ENSORLP00000019211
**Stickleback** (*Gasterosteus aculeatus*)					
Lyz	GroupXIV	-	13,700,784-13,704,111	Y	ENSGACP00000024176
Takifugu (*Takifugu rubripes*)					
Lyz	Scaffold_106	-	514,080-517,847	Y	ENSTRUP00000015311
Tetraodon (*Tetraodon nigroviridis*)					
Lyz	Un_random	+	33,003,553-33,006,230	Y	ENSTNIP00000017205
**Lamprey** (*Petromyzon marinus*)					
Lyz	NO HIT				-

[a] – Chromosome/contig/superContig, strand and base coordinates from Genomic data in *Ensembl* release 57. NO HIT means no best match was found by *Blast*.
[b] – Full length: Y, a full length protein coding sequence can be predicted; Incomplete, sequence does not contain all four coding exons; Pseudo, sequence predicts non-functional gene sequence (pseudogene) due to deletions, frameshifts or non-sense substitutions.
[c] – Protein ID from *Ensembl*, or NCBI identifier if no *Ensembl* ID available or is incomplete; -, indicates no *Ensembl* ID or NCBI identifier was available.
[d] – Otherwise full length sequence has a short internal unknown sequence.
[e] – Sequence edited to extend to full-length (i.e., *Ensembl* predicted sequence is not full-length).
[f] – Sequences were full-length in a previous draft assembly (release 53).
