# Supplementary material for: Evolution of the mammalian lysozyme gene family
Source: BMC Evol Biol. 2011 Jun 15;11:166. doi: 10.1186/1471-2148-11-166 (PMC3141428; doi:10.1186/1471-2148-11-166)
Supplement: Additional file 2 — Supplementary Figure 1. This file is in PDF format. Phylogeny of vertebrate lysozyme-like sequences generated by PhyloBayes. [file 1471-2148-11-166-S2.PDF]

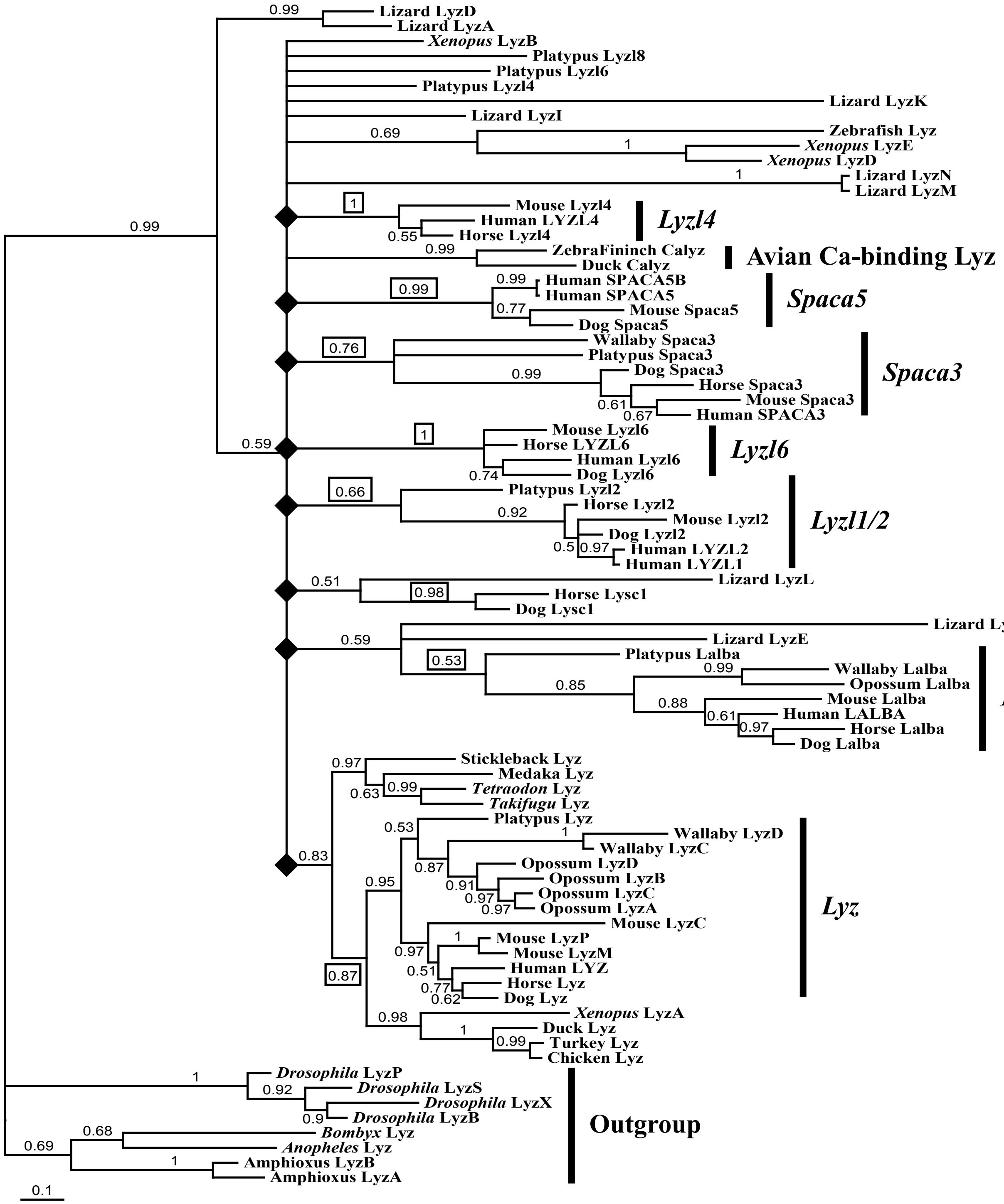

Supplementary Figure 1. Bayesian inference phylogenetic tree of vertebrate lysozyme proteins. A Bayesian phylogenetic tree was generated by *PhyloBayes* [62] from the amino acid sequences of lysozyme-like sequences from diverse vertebrates. The phylogeny was rooted with sequences from Amphioxus and insects. The sequences were aligned using the MAFFT algorithm [56]. The tree was generated when the analysis was terminated by the autostopping rule (using bpcomp and tracecomp) when the discrepancies between the two runs was below 0.2 and all effective sizes were greater than 100. This tree was generated after 15,000 cycles. Nodes which represent gene duplications that generated the mammalian lysozyme-like genes are indicated as diamonds. The posterior probabilities of each node are shown. Support values for each of the mammalian lysozyme-like genes are boxed. Monophyletic Lyzl4 and Lyzl6 gene clusters were not seen in this majority rule tree but did receive posterior probabilities of 0.32 (and 0.43 with Lizard LyzN, LyzM, and LyzK) and 0.47, respectively.
