# Supplementary material for: Evolution of the mammalian lysozyme gene family
Source: BMC Evol Biol. 2011 Jun 15;11:166. doi: 10.1186/1471-2148-11-166 (PMC3141428; doi:10.1186/1471-2148-11-166)
Supplement: Additional file 3 — Supplementary Figure 2. This file is in PDF format. Phylogeny of vertebrate lysozyme-like sequences generated by PhyML. [file 1471-2148-11-166-S3.PDF]

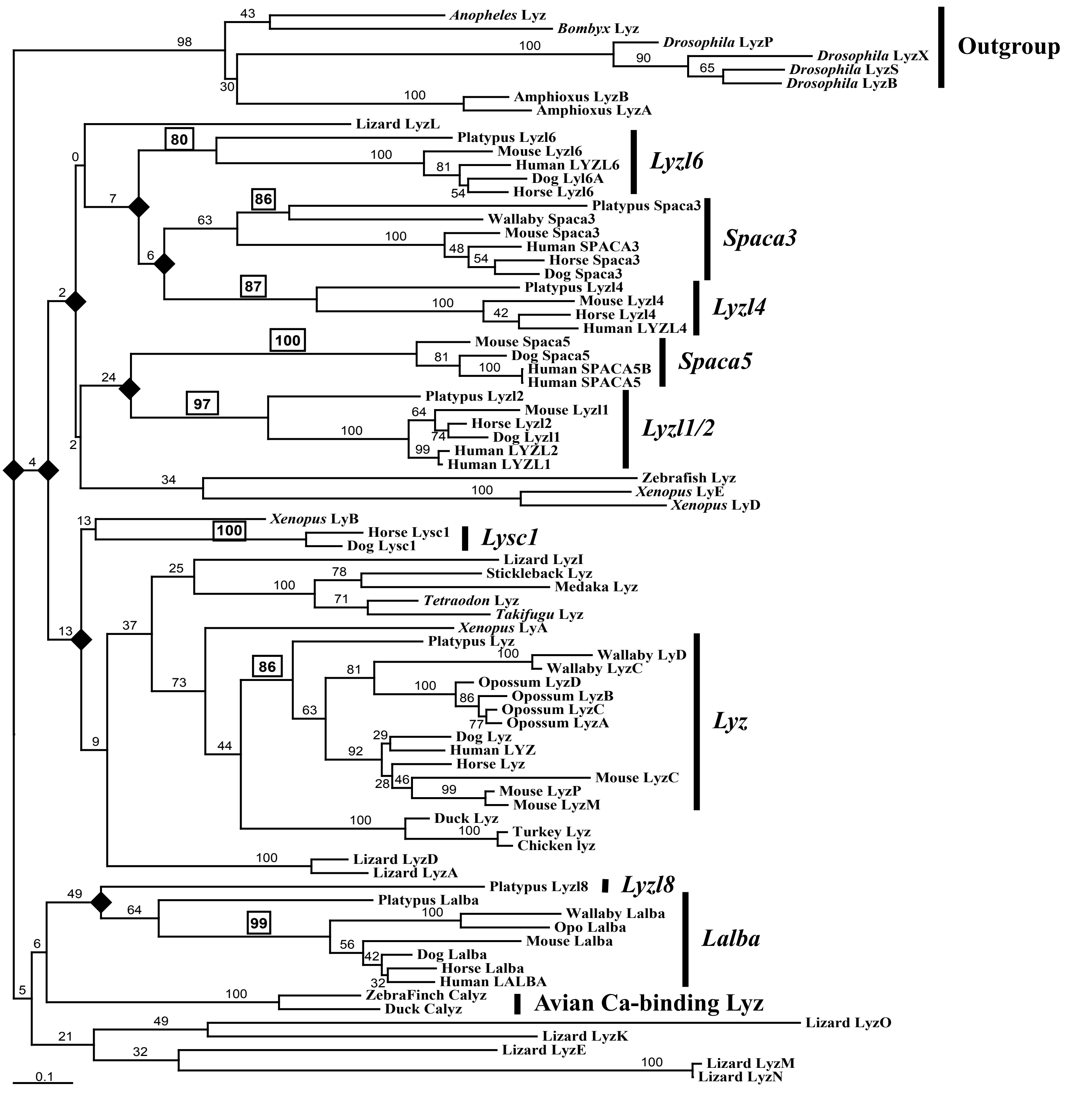

Supplementary Figure 2. Maximum likelihood phylogenetic tree of vertebrate lysozyme genes. A bootstrapped maximum likelihood phylogenetic tree was generated by *PhyML* [63] using DNA coding sequences of lysozyme-like sequences from diverse vertebrates. The phylogeny was rooted with sequences from Amphioxus and insects. The sequences were aligned using the MAFFT algorithm [56]. The tree is a majority rule tree from 100 bootstrap replicates. Parameters for the analysis included the use of the TN93 substitution model, which was the best available model selected by *ModelTest* [66-68], with the transition/transversion ratio, proportion of invariant sites, and gamma shape parameters estimated by *PhyML*. Nodes that represent duplications generating the different types of mammalian lysozyme-like genes are indicated by diamonds. The proportions of bootstrap replications that support each node are shown. The support for the monophyly of each mammalian lysozyme-like gene is boxed.
