# Supplementary material for: Evolution of the mammalian lysozyme gene family
Source: BMC Evol Biol. 2011 Jun 15;11:166. doi: 10.1186/1471-2148-11-166 (PMC3141428; doi:10.1186/1471-2148-11-166)
Supplement: Additional file 4 — Supplementary Figure 3. This file is in PDF format. Phylogeny of only mammalian lysozyme-like sequences generated by MrBayes with support for the orthologous genes by different phylogenetic methods. [file 1471-2148-11-166-S4.PDF]

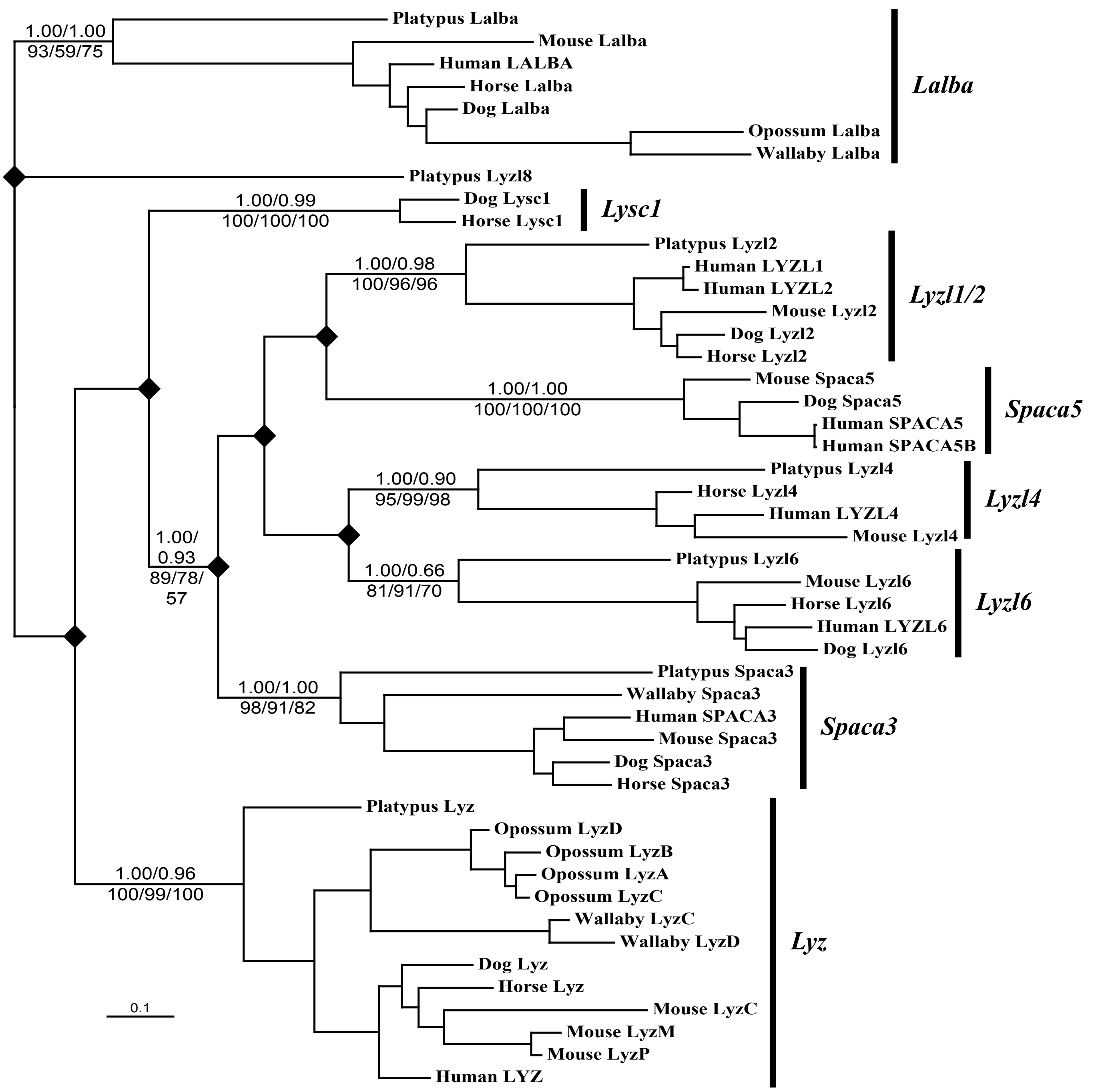

Supplementary Figure 3. Support for the monophyly of the different types of mammalian lysozyme-like sequences. A Bayesian phylogeny of mammalian lysozyme-like sequences generated by *MrBayes* [60,61] is presented. Lactalbumin (*Lalba*) sequences were arbitrarily chosen as the root. The levels of support for the monophyly of each type of lysozyme-like gene, as well as for the clustering of *Lyzl1/2*, *Lyzl4*, *Lyzl6*, *Spaca3*, and *Spaca5* are shown on the ancestral lineages with posterior probabilities (from *MrBayes* [60,61] and *PhyloBayes* [62], respectively) above the lineage and the bootstrap support (from *PhyML* [63], neighbour-joining (by *MEGA* [64]), and parsimony (by *PAUP* [65]), respectively) shown below. *MrBayes* was run with nst=2 and rates=gamma. *PhyloBayes* ran for 5800 cycles. *PhyML* used the TN93 substitution model and bootstrapped 100 times. *MEGA* used Maximum Composite Likelihood nucleotide distances and bootstrapped 1000 times. *PAUP* was bootstrapped 1000 times. Nodes that represent gene duplications that generated the different mammalian lysozyme-like genes are indicated by diamonds.
