# Supplementary material for: Evolution of the mammalian lysozyme gene family
Source: BMC Evol Biol. 2011 Jun 15;11:166. doi: 10.1186/1471-2148-11-166 (PMC3141428; doi:10.1186/1471-2148-11-166)
Supplement: Additional file 5 — Supplementary Figure 4. This file is in PDF format. Conservation of genomic organization near Lyzl1/2 genes. [file 1471-2148-11-166-S5.PDF]

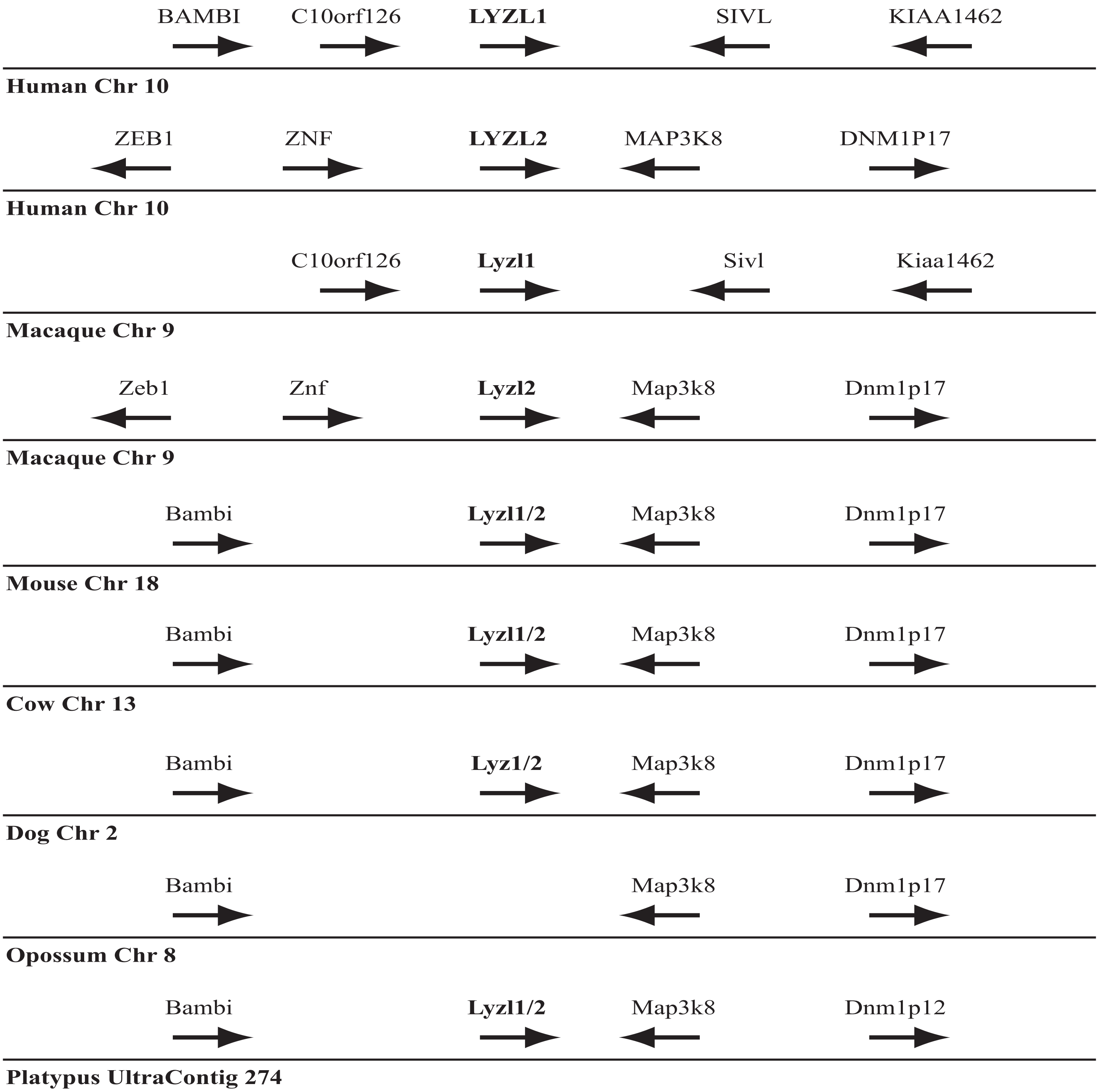

Supplementary Figure 4. Conservation of genomic organization near Lysozyme-like 1/2 genes (*Lyzl1/2*) in diverse vertebrates. Species and chromosomes (or contigs or scaffolds) are from *Ensembl* [16] and are shown on the left. Direction of transcription of each gene is indicated by the arrowheads. Gene sizes and distances between genes are not to scale. *Lyzl1* and *Lyzl2* are duplicated genes that are found only in some primate species, and are located about 1.3 Mb apart in the human genome. The distance between the human *BAMBI* and *SVIL* genes is about 770 kb, and between *ZNF438* and *MAP3K8* is about 390 kb. Gene symbols are: *BAMBI*, BMP and activin membrane-bound inhibitor; *C10orf126*, Chromosome 10 open reading frame 126; *SVIL*, Supervillin; *KIAA1462*, Uncharacterized protein KIAA1462; *DNM1P17*, DNM1 pseudogene; *MAP3K8*, Mitogen-activated protein kinase kinase kinase 8; *ZNF*, member of the Zinc finger protein family; *ZEB1*, Zinc finger E-box-binding homeobox 1.
