# Supplementary material for: Evolution of the mammalian lysozyme gene family
Source: BMC Evol Biol. 2011 Jun 15;11:166. doi: 10.1186/1471-2148-11-166 (PMC3141428; doi:10.1186/1471-2148-11-166)
Supplement: Additional file 6 — Supplementary Figure 5. This file is in PDF format. Conservation of genomic organization near Lyzl4 genes. [file 1471-2148-11-166-S6.PDF]

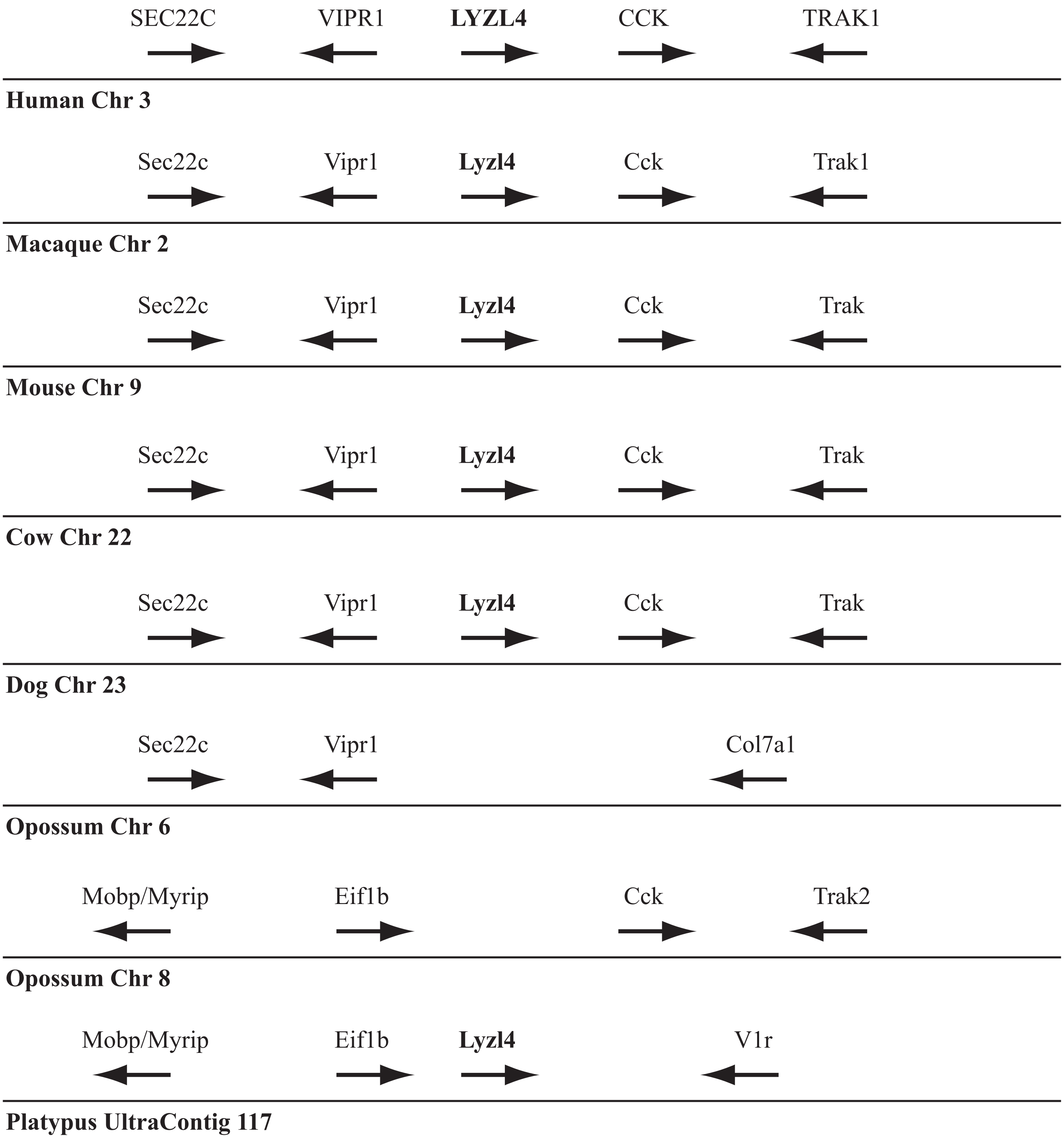

Supplementary Figure 5. Conservation of genomic organization near Lysozyme-like 4 genes (*Lyzl4*) in diverse vertebrates. Species and chromosomes (or contigs or scaffolds) are from *Ensembl* [16] and are shown on the left. Direction of transcription of each gene is indicated by the arrowheads. Gene sizes and distances between genes are not to scale. The distance between the human *VIPR* and *CCK* genes is about 240 kb. Gene symbols are: *TRAK1*, Trafficking kinesin-binding protein 1; *CCK*, Cholecystokinin precursor; *VIPR1*, Vasoactive intestinal polypeptide receptor 1 precursor; *SEC22c*, Vesicle-trafficking protein SEC22c; *COL7A1*, Collagen alpha-1 (VII) chain precursor; *EIF1B*, Eukaryotic translation initiation factor 1b (eIF1b); *Mobp*, Myelin-associated oligodendrocyte basic protein; *Myrip*, myosin VIIA and Rab interacting protein; *V1r*, a member of the family of vomernasal receptor gene family.
