# Supplementary material for: Evolution of the mammalian lysozyme gene family
Source: BMC Evol Biol. 2011 Jun 15;11:166. doi: 10.1186/1471-2148-11-166 (PMC3141428; doi:10.1186/1471-2148-11-166)
Supplement: Additional file 7 — Supplementary Figure 6. This file is in PDF format. Phylogeny of Lyzl4 genes. [file 1471-2148-11-166-S7.PDF]

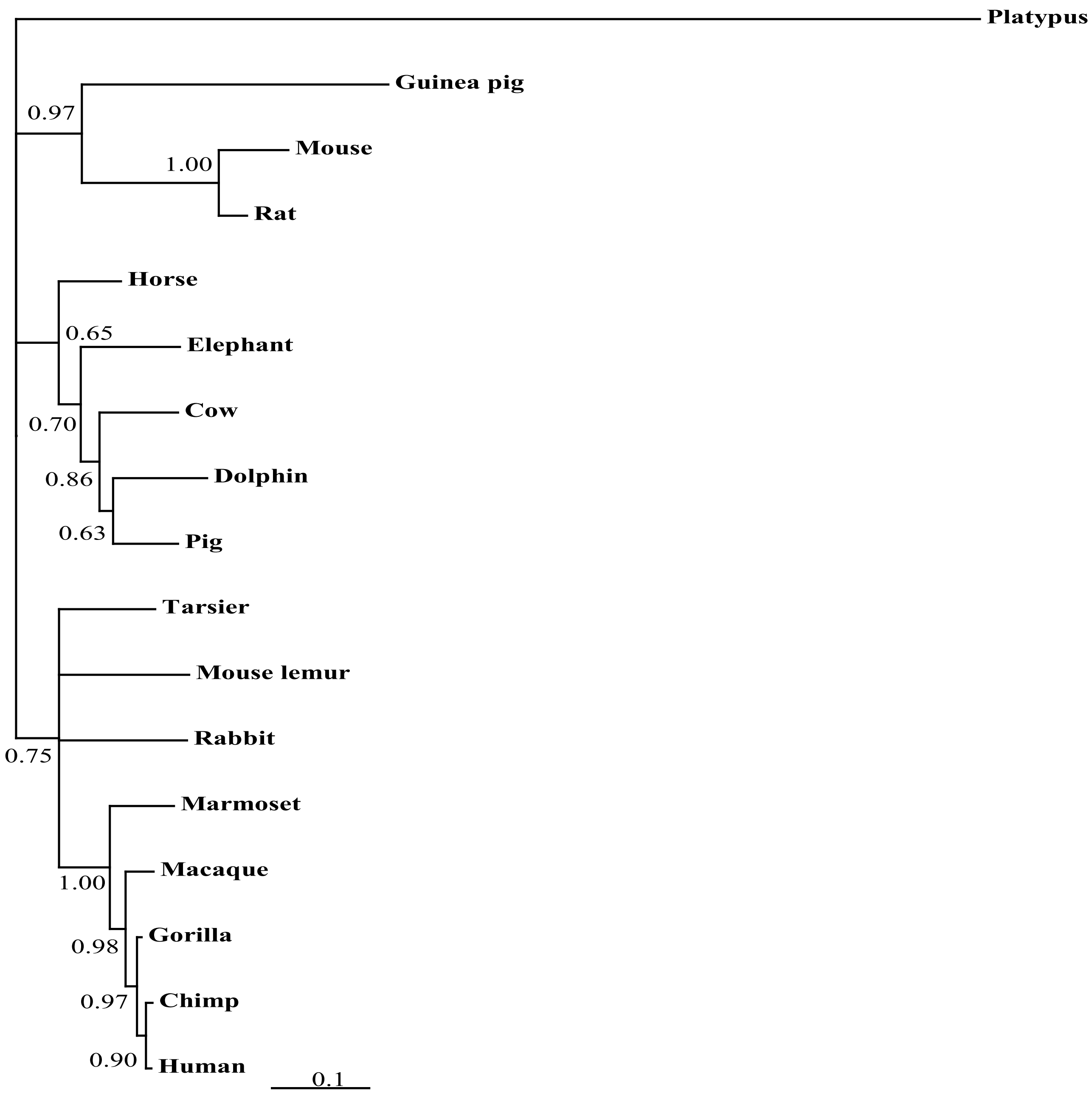

Supplementary Figure 6. Phylogeny of mammalian Lysozyme-like 4 (*Lyzl4*) genes. A Bayesian phylogenetic tree of mammalian lysozyme-like 4 genes was generated by *MrBayes* [60,61] using the DNA coding sequences of mammalian *Lyzl4* sequences. This tree was built with nst=2 and rates=gamma as selected by *ModelTest* [66-68]. The tree was rooted with the platypus *Lyzl4* sequence.
