# Supplementary material for: Evolution of the mammalian lysozyme gene family
Source: BMC Evol Biol. 2011 Jun 15;11:166. doi: 10.1186/1471-2148-11-166 (PMC3141428; doi:10.1186/1471-2148-11-166)
Supplement: Additional file 8 — Supplementary Figure 7. This file is in PDF format. Conservation of genomic organization near Lyzl6 genes. [file 1471-2148-11-166-S8.PDF]

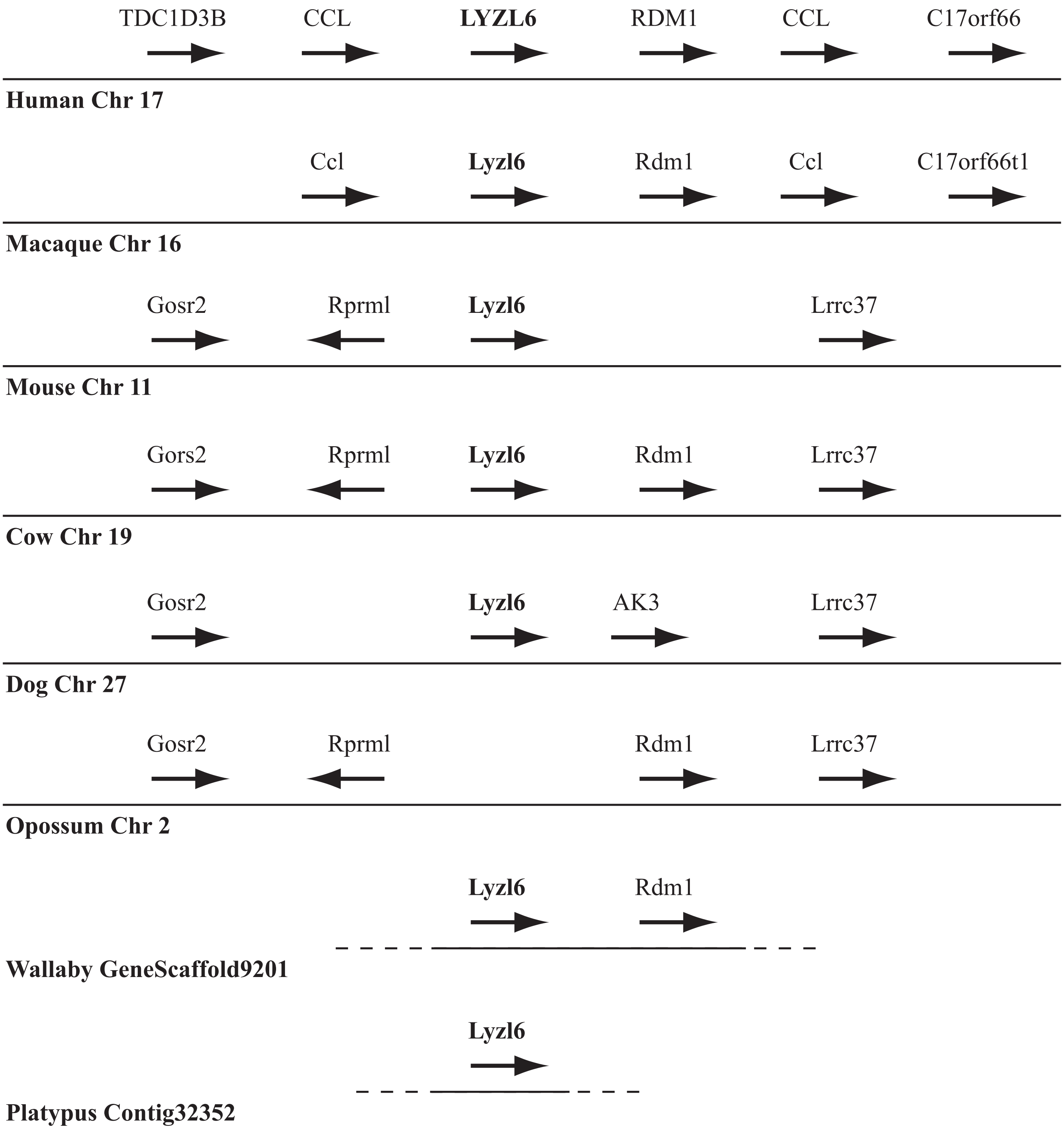

Supplementary Figure 7. Conservation of genomic organization near Lysozyme-like 6 genes (*Lyzl6*) in diverse vertebrates. Species and chromosomes (or contigs or scaffolds) are from *Ensembl* [16] and are shown on the left. Direction of transcription of each gene is indicated by the arrowheads. Gene sizes and distances between genes are not to scale. Short genomic contigs are indicated by the shorter solid lines flanked by dashed lines. The distance between the human *TBC1D3F* and *RDM1* genes is about 240 kb. Gene symbols are: *TBC1D3F*, TBC1 domain family member 3B/I; *CCL*, a member of the chemokine ligand family; *RDM1*, RAD52 motif-containing protein 1; *C17orf66*, chromosome 17 open reading frame 66; *Gosr2*, Golgi SNAP receptor complex member 2; *Rprml*, Reprimo-like protein; *Lrrc37*, a member of the leucine-rich repeat containing 37A family.
