# Supplementary material for: Evolution of the mammalian lysozyme gene family
Source: BMC Evol Biol. 2011 Jun 15;11:166. doi: 10.1186/1471-2148-11-166 (PMC3141428; doi:10.1186/1471-2148-11-166)
Supplement: Additional file 10 — Supplementary Figure 9. This file is in PDF format. Conservation of genomic organization near Spaca3 genes. [file 1471-2148-11-166-S10.PDF]

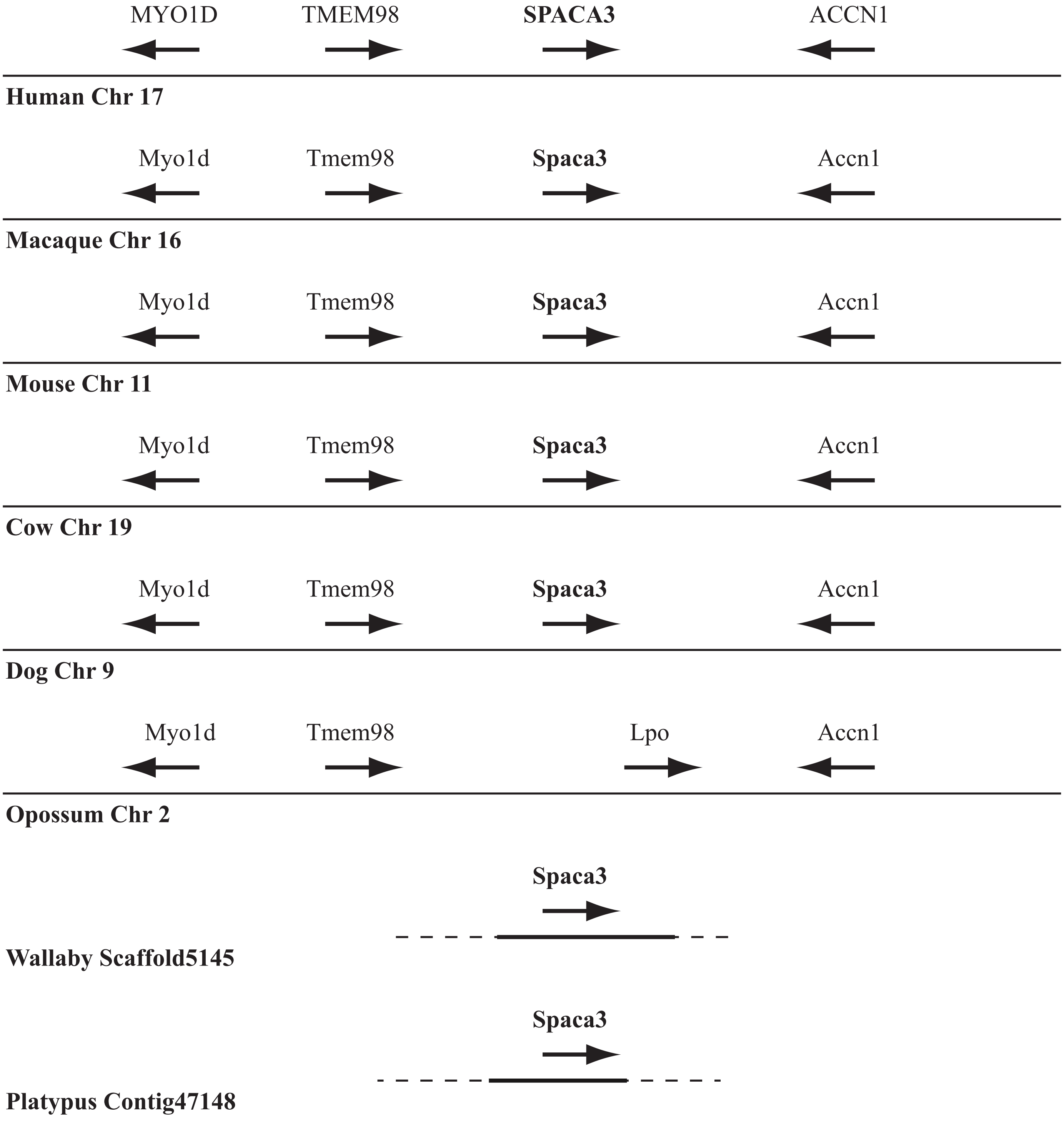

Supplementary Figure 9. Conservation of genomic organization near Sperm acrosomal protein 3 genes (*Spaca3*) in diverse vertebrates. Species and chromosomes (or contigs or scaffolds) are from *Ensembl* [16] and are shown on the left. Direction of transcription of each gene is indicated by the arrowheads. Gene sizes and distances between genes are not to scale. Short genomic contigs are indicated by the shorter solid lines flanked by dashed lines. The distance between the human *TMEM98* and *ACCN1* genes is about 75 kb. Gene symbols are: *TMEM98*, Transmembrane protein 98; *SPACA3*, Sperm acrosome membrane-associated protein 3 Precursor; *ACCN1*, Amiloride-sensitive cation channel 1, neuronal; *MYO1D*, Myosin-Id; *RPL23*, 60S ribosomal protein L23; *Lpo*, lactoperoxidase.
