# Supplementary material for: Evolution of the mammalian lysozyme gene family
Source: BMC Evol Biol. 2011 Jun 15;11:166. doi: 10.1186/1471-2148-11-166 (PMC3141428; doi:10.1186/1471-2148-11-166)
Supplement: Additional file 11 — Supplementary Figure 10. This file is in PDF format. Phylogeny of Spaca3 genes. [file 1471-2148-11-166-S11.PDF]

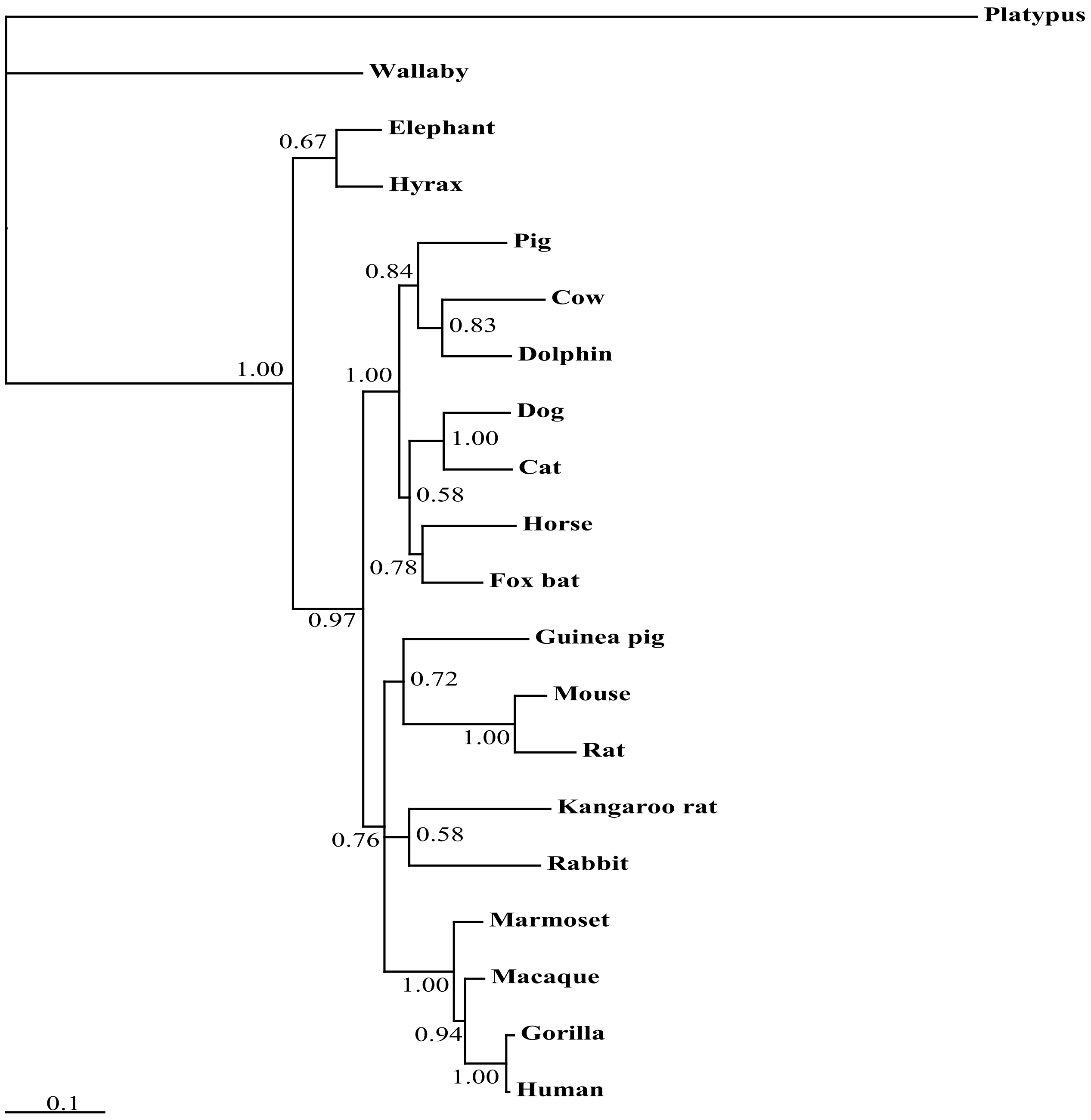

Supplementary Figure 10. Phylogeny of mammalian Sperm acrosomal protein (*Spaca3*) genes. A Bayesian phylogenetic tree of mammalian sperm acrosomal protein 3 genes was generated by *MrBayes* [60,61] using the DNA coding sequences of mammalian *Spaca3* sequences. This tree was built with nst=6 and rates=gamma as selected by *ModelTest* [66-68]. The tree was rooted with the platypus *Spaca3* sequence.
