# Supplementary material for: Evolution of the mammalian lysozyme gene family
Source: BMC Evol Biol. 2011 Jun 15;11:166. doi: 10.1186/1471-2148-11-166 (PMC3141428; doi:10.1186/1471-2148-11-166)
Supplement: Additional file 12 — Supplementary Figure 11. This file is in PDF format. Conservation of genomic organization near Spaca5 genes. [file 1471-2148-11-166-S12.PDF]

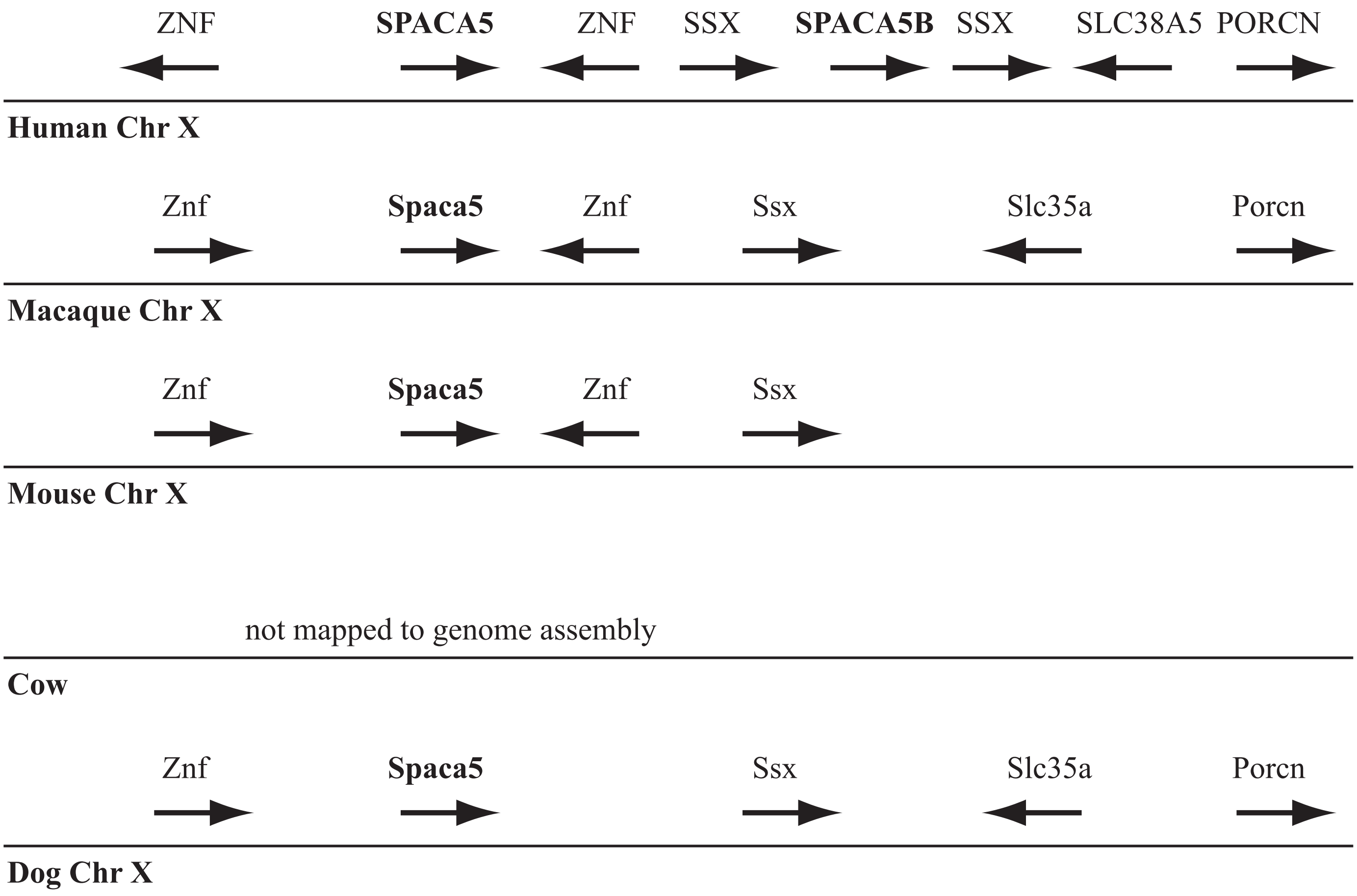

Supplementary Figure 11. Conservation of genomic organization near Sperm acrosomal protein 5 genes (*Spaca5*) in diverse vertebrates. Species and chromosomes (or contigs or scaffolds) are from *Ensembl* [16] and are shown on the left. Direction of transcription of each gene is indicated by the arrowheads. Gene sizes and distances between genes are not to scale. The distance between the human *ZNF182* and *ZNF630* genes (*ZNF* genes flanking *SPACA5*) is about 60 kb, and between *ZNF630* and *PORCN* (Flanking *SPACA5B*) is about 450 kb. Gene symbols are: *ZNF*, a member of the Zinc finger gene family; *SSX*, a member of the Synovial sarcoma, X breakpoint family; *PORCN*, Probable protein-cysteine N-palmitoyltransferase porcupine; *SLC38A5*, Solute carrier family 38 member 5.
