# Supplementary material for: Evolution of the mammalian lysozyme gene family
Source: BMC Evol Biol. 2011 Jun 15;11:166. doi: 10.1186/1471-2148-11-166 (PMC3141428; doi:10.1186/1471-2148-11-166)
Supplement: Additional file 15 — Supplementary Figure 14. This file is in PDF format. Conservation of genomic sequences between Lalba and c12orf41 genes. [file 1471-2148-11-166-S15.PDF]

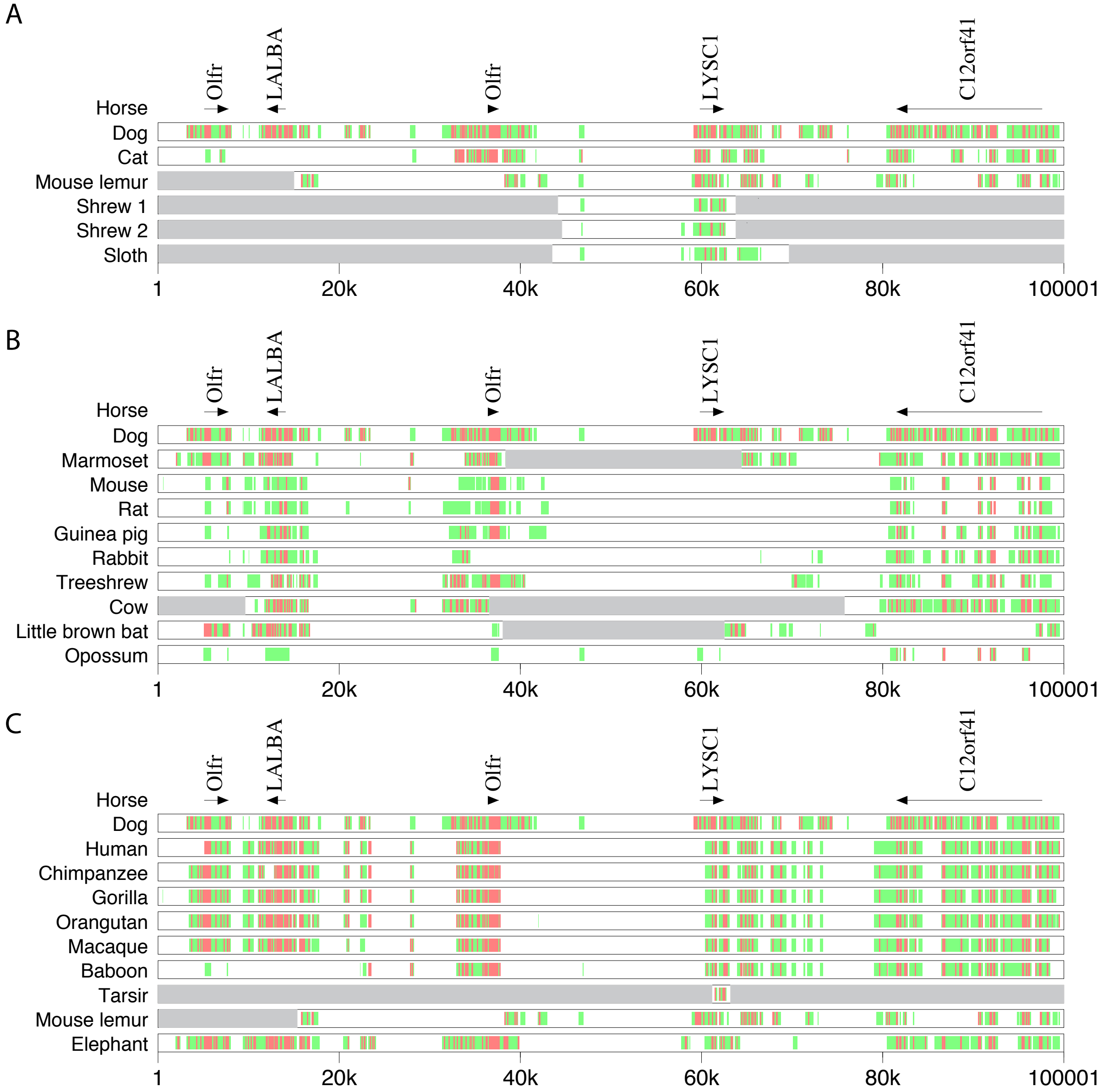

Supplementary Figure 14. Conservation of genomic sequences between the lactalbumin (*Lalba*) and *C12orf14* genes in diverse mammals. Percent identify plots (PIPs) were generated by *MultiPipMaker* [42,43] for genomic sequences spanning the *Lalba* and *C12orf41* genes. The summary figure is shown, wherein sequences with greater similarity to the horse sequence (top line in each figure) are indicated in red, lower similarity in green, and no similarity (due to absence of similarity between the sequences) in white, with the approximate locations of large sequence gaps (i.e., incomplete genome sequence) shown in grey. The relative position and orientation of the gene near the calcium-binding lysozyme (*Lysc1*) gene in the horse genome is shown at the top of each plot, with the similarity of each species to the horse sequence (indicated on the left). The shrew has two *Lysc1* genes, which were analyzed separately. *Olfr* indicate genes (or pseudogenes) with similarity to olfactory receptors. A, Percent identity plot (PIP) of mammalian species that were identified as having calcium-binding lysozyme genes based on our tBlastn results. The shrew and sloth genes are on short genomic contigs, and thus do not show similarity beyond the *Lysc1* gene. B, Percent identity plot (PIP) of mammalian species that do not have a calcium-bind lysozyme gene are compared to that of the horse and dog. The sequence with high sequence similarity in the little brown bat genome near the *Lysc1* gene maps just downstream of the gene and is at the boundary of a sequence gap in the little brown bat genomic sequence, raising the possibility that a *Lysc1* gene may exist in a gap in this genome sequence. C, Percent identity plot (PIP) of primate and elephant genomic sequences compared to the horse and dog sequences. These sequences did not show similarity to lysozyme-like sequences based on our tBlastn analysis, but MultiPipMaker alignments indicate that they do have *Lysc1*-like sequences. The predicted amino acid and DNA coding sequences are shown in Figure 8, and Additional file 16: Figure S15, respectively. The tarsier and mouse lemur sequences are on shorter genomic contigs, and thus have limited similarity to other genomes.
