# Supplementary material for: Evolution of the mammalian lysozyme gene family
Source: BMC Evol Biol. 2011 Jun 15;11:166. doi: 10.1186/1471-2148-11-166 (PMC3141428; doi:10.1186/1471-2148-11-166)
Supplement: Additional file 16 — Supplementary Figure 15. This file is in PDF format. DNA sequences of Lysc1 genes. [file 1471-2148-11-166-S16.PDF]

```
                                       Signal Peptide                                 +1
             M   R   S   T   L   I   I   S   L   L   S   C   F   F   A   V   Y   E   A   K   V   F   S   K   C
Horse       ATG AAG TCC ACT CTC ATC ATC TCC CTC CTC AGC TGC TTC TTT GCA GTT TAC GAG GCC AAA GTC TTC TCC AAG TGT
Dog         ... ... ..T .T. ..G G.. ... A.. A.. ... ... .A. ... ... .T. .CA G.T ... ... ... A.. ... ... ... ...
Cat         ... ... ... .T. ..G ... ... ... ... ... G.. .A. ... ... ... .CA ..T ... ... ... A.. ... C.. ... ...
Human       --- --- --- --- --- --- --- --- --- --- --- --- --- --- --- --- --- --- --- --- --- --- --- --- ---
Chimpanzee  --- --- --- --- --- --- --- --- --- --- --- --- --- --- --- --- --- --- --- --- --- --- --- --- ---
Gorilla     --- --- --- --- --- --- --- --- --- --- --- --- --- --- --- --- --- --- --- --- --- --- --- --- ---
Orangutan   --- --- --- --- --- --- --- --- --- --- --- --- --- --- --- --- --- --- --- --- --- --- --- --- ---
Macaque     --- --- --- --- --- --- --- --- --- --- --- --- --- --- --- --- --- --- --- --- --- --- --- --- ---
Baboon      --- --- --- --- --- --- --- --- --- --- --- --- --- --- --- --- --- --- --- --- --- --- --- --- ---
Tarsier     ??? ??? ??? ??? ??? ??? ??? ??? ??? ??? ??? ??? ??? ??? ??? ??? ??? ??? ??? ??? ??? ??? ??? ??? ???
Mouse lemur ... .A. ... .T. ..G ... ... ... ... ... ... ... ... ... ... .C. ..T ... ..G ... ... ... A.. ... .AC
Shrew 1     ... ... ... .TG ..G ..T C.. .TT ... T.. ... ... ... ... ... AG. ..T ..T ... ... A.. ... ..T G.A ...
Shrew 2     ... ... ... .TC ..G G.. ... ..T ... ... ..T ... ... ... ..G .CC ... ... ... ... A.. ... C.. .GA ...
Elephant    --- --- --- --- --- --- --- --- --- --- --- --- --- --- --- --- --- --- --- --- --- --- --- --- ---
Sloth       ... .A. ... .T. T.T ... T.. ... ... ... G.. ..A ... ... ... T.. ... A.. ... ... ... ... ... .G. ...
            <<<     Exon 1


             E   L   A   H   K   L   K   A   Q   E   M   D   G   F   G   G   Y   S   L   A   N   W   V   C   M
Horse       GAG CTG GCC CAC AAG CTA AAG GCC CAG GAA ATG GAT GGT TCC GGT GGC TAC AGC CTG GCA AAC TGG GTC TGC ATG
Dog         ... ... ... .G. ... ..G ... AG. AT. .G. ... ... ..C ... CA. ... ... ... ... ... ... ... ... ... ...
Cat         ... ... ... .G. ... ..G ... ..A G.. .G. ... ... ..C ... CAC ... ... ... ... ... ... .?? ??? ??? ???
Human       --- --- --- --- --- --- --- --- --- --- --- --- --- --- --- --- --- --- --- --- --- -.. A.. ... T..
Chimpanzee  --- --- --- --- --- --- --- --- --- --- --- --- --- --- --- --- --- --- --- --- --- -.. A.. ... T..
Gorilla     --- --- --- --- --- --- --- --- --- --- --- --- --- --- --- --- --- --- --- --- --- -.. A.. ... T..
Orangutan   --- --- --- --- --- --- --- --- --- --- --- --- --- --- --- --- --- --- --- --- --- -.. A.. ... T..
Macaque     --- --- --- --- --- --- --- --- --- --- --- --- --- --- --- --- --- --- --- --- --- -.. C.. ... T..
Baboon      --- --- --- --- --- --- --- --- --- --- --- --- --- --- --- --- --- --- --- --- --- -.. C.. ... T..
Tarsier     ??? ??? ??? ??? ??? ??? ??? ??? ??? ??? ??? ??? ??? ??? ??? ??? ??? ??? ??? ??? ??? ??? ??? ??? ???
Mouse lemur C.T ... ..T TG. ... ..G ... ... ..A .G. ... ..C .AC ... CA. ... ... ... ... ... ... ... ... ... ...
Shrew 1     ... ..A ... A.A ... ..G ... ... ..T .G. ... ... AAC ... CA. ..T ... ... ... ... ... ... ... ... ...
Shrew 2     ... ..A ... A.G ... ..G ... ... ... .GG C.. ... ..C .A. CAG ... ... ... ... ... ... ... ... ... T..
Elephant    --- --- --- --- --- --- --- --- --- --- --- --- --- --- --- --- --- --- --- --- --- -.. ... ... ...
Sloth       ... G.- ... TG. ..C ... ... ... ... .G. ... ... ..C ... T.. ... ... .A. ... .A. ... C.. .C. ..A ...
                                                                                  Exon 1    >>><<<    Exon 2
```

	A	E	Y	E	S	N	F	N	T	R	A	F	N	G	K	N		A	N	G	S	S	D	Y	G
Horse	GCT	GAG	TAT	GAG	AGT	AAC	TTC	AAC	ACC	CGG	GCC	TTT	AAT	GGA	AAA	AA-	--T	GCC	AAT	GGC	AGT	AGT	GAC	TAT	GGG
Dog	...	...	...	...	...	...	...	...	...	.A.	...	...	...	..G	.G.	...	...	T..	...	...	...	...	...	...	..A
Cat	???	???	???	???	???	???	???	???	???	???	???	???	???	???	???	???	???	???	???	???	???	???	???	???	???
Human	.T.	C..	.G.	...	...	..T	..G	...	...	.A.	...	...	...	...	...	...	.C.	..T	G..	...	...	...	...	A.C	...
Chimpanzee	.T.	C..	.G.	.G.	...	...	..G	...	...	.A.	...	...	...	...	...	...	.C.	..T	G..	...	...	...	...	A.C	...
Gorilla	.T.	C..	.G.	...	...	...	..G	...	...	.A.	...	...	..C	...	...	...	.C.	..T	G..	...	...	...	...	A.C	...
Orangutan	.T.	C..	.G.	..A	...	...	..G	...	...	.A.	...	...	...	...	...	...	.C.	..T	G..	...	...	...	...	A.C	...
Macaque	.T.	C..	.G.	...	...	...	..G	...	...	.A.	...	...	...	...	...	..A	AC.	..T	G..	...	...	...	...	G.C	..-
Baboon	.T.	C..	.G.	...	...	...	..G	...	...	.A.	...	...	...	...	...	...	AC.	..T	G..	...	...	...	...	G.C	..-
Tarsier	???	???	???	???	???	???	???	???	???	???	???	???	???	???	???	???	???	???	???	???	???	???	???	???	???
Mouse lemur	.T.	C..	...	...	...	...	..G	...	...	.A.	...	...	...	...	...	.-.	...	...	...	...	...	...	.C.	C..	...
Shrew 1	...	A..	C.C	..A	...	G.T	..A	..T	...	AA.	...	C.C	...	.AC	...	...	...	T.T	G..	...	.CC	.T.	...	...	...
Shrew 2	...	..T	C..	...	...	.G.	.A.	..T	..T	AAA	...	..C	...	..G	...	...	...	T.T	G..	...	..C	...	...	...	...
Elephant	...	TG.	...	AG.	.A.	...	...	...	.-A	.A.	..T	..-	...	...	GG.	...	...	T..	...	A..	...	..A	...	...	...
Sloth	..C	C..	...	.G.	..C	..T	...	...	...	.A.	..T	...	...	...	..T	...	...	T..	.G.	..T	G..	...	A.T	...	...

	L	F	Q	L	N	N	K	W	W	C	K	D	N	K	R	S	S	S	N	A	C	N	I	M	C
Horse	CTC	TTC	CAG	CTG	AAC	AAC	AAG	TGG	TGG	TGC	AAA	GAT	AAC	AAG	CGT	TCT	TCA	TCA	AAT	GCC	TGC	AAC	ATA	ATG	TGC
Dog	A..	...	...	...	...	.G.	...	...	...	...	...	AGC	...	TCT	.AC	..C	..G	G..	...	...	...	...	...	...	...
Cat	???	???	???	???	???	???	???	???	???	???	???	???	???	???	???	???	???	???	???	???	???	???	???	???	???
Human	A.T	...	...	...	...	...	---	.TC	..A	.A.	...	..C	C..	...	.A.	..C	...	GA.	...	...	...	...	C..	...	...
Chimpanzee	A.T	...	...	...	...	...	---	.TC	..A	.A.	...	..C	C..	...	.A.	..C	...	GA.	...	...	...	...	C..	...	...
Gorilla	A.T	...	...	...	...	...	---	.TC	..A	.A.	...	..C	C..	...	.A.	..C	...	GA.	...	...	...	...	C..	...	...
Orangutan	A.T	...	...	...	...	...	---	.T.	..A	.A.	...	..C	C..	...	.A.	..C	...	GA.	...	...	...	...	G..	...	...
Macaque	---	---	...	...	...	...	---	.T.	..A	...	...	..C	C..	...	.A.	..G	...	GA.	...	...	...	...	G..	...	...
Baboon	---	---	...	...	...	...	---	.T.	..A	...	...	..C	C..	...	.A.	..G	...	GA.	...	...	...	...	G..	...	...
Tarsier	???	???	???	???	???	???	???	???	???	???	???	???	???	???	???	???	???	???	???	???	???	???	???	???	???
Mouse lemur	A..	..A	...	...	...	...	---	.TA	...	...	...	...	...	...	..C	C.C	...	G-.	...	...	...	G.T	...	CCA	...
Shrew 1	A.A	...	...	G..	...	...	..A	...	...	...	..G	..C	...	..A	...	CAG	...	AA.	...	...	...	...	...	GCA	...
Shrew 2	A..	...	...	...	...	...	.G.	...	...	...	.C.	..C	.G.	...	TA.	C.C	..T	G.G	C.C	.G.	...	...	..G	GCA	...
Elephant	A.A	...	...	...	...	---	.G.	..A	...	...	...	..C	...	.GA	.A.	..C	.T.	GA.	...	...	..T	...	...	...	...
Sloth	A..	...	...	...	..T	GG.	...	C..	...	...	CC.	..C	---	...	AT.	C.C	..G	GA.	..C	...	..A	.GA	C..	CAC	...

Exon 2

	S	K	L	L	D	E	N	I	D	D	D	I	S	C	A	K	R	V	V	R	D	P	K	G	M
Horse	AGC	AAA	CTT	TTG	GAT	GAG	AAC	ATC	GAT	GAT	GAC	ATC	AGC	TGT	GCC	AAG	AGG	GTT	GTG	AGA	GAT	CCT	AAA	GGG	ATG
Dog	...	..G	T.C	C..	...	..C	...	...	...	...	..T	...	GC.	...	...	...	...	...	...	.A.	...	...	..T	...	...
Cat	???	???	???	???	???	???	???	???	???	???	???	???	???	???	???	???	???	???	???	???	???	???	???	???	???
Human	...	..G	T..	A.T	...	..T	...	..T	G..	...	...	.AA	---	-C.	...	...	...	AC.	...	TA.	...	...	...	.T.	..A
Chimpanzee	...	..G	T..	A.T	...	..T	...	..T	G..	...	...	.AA	---	-C.	...	...	...	AC.	...	.A.	...	...	...	.T.	..A
Gorilla	...	.??	???	???	???	???	???	???	???	???	???	???	???	???	???	???	?..	AC.	...	.A.	...	...	...	.T.	..A
Orangutan	...	..G	T..	A.T	...	..T	...	..T	G..	...	...	.AA	---	-..	...	...	...	AC.	...	.A.	...	...	...	.T.	..A
Macaque	...	..G	T..	A.T	...	..T	...	..T	G..	...	...	.AA	---	-..	...	...	...	AC.	...	.A.	...	...	...	.T.	G.A
Baboon	...	..G	T..	A.T	...	..T	...	..T	G..	...	...	.AA	---	-..	...	...	...	AC.	...	.A.	...	...	...	.T.	G.A
Tarsier	???	?..	T..	A.T	...	A.A	...	.C.	A..	...	...	...	CT.	...	...	..A	...	...	...	.A.	...	...	...	...	...
Mouse lemur	...	G..	T..	AG.	..G	..T	..G	G..	...	...	...	...	GT.	...	...	T.A	...	...	...	.A.	...	...	...	...	...
Shrew 1	...	...	..C	...	...	..C	...	..T	...	...	...	...	.CT	...	AT.	..A	..A	A.A	...	.A.	...	...	..C	AA.	...
Shrew 2	..T	...	T..	...	...	..C	...	..T	...	...	...	...	GC.	...	...	...	..A	...	...	.A.	...	..C	..T	...	..A
Elephant	.A.	...	T..	A..	...	..T	GG.	.CT	...	A..	.G.	...	T..	...	...	...	..T	...	...	.AC	...	...	..T	...	...
Sloth	...	.--	---	---	---	---	---	---	---	--G	...	...	..G	...	.-.	...	...	S..	...	.A.	...	.T.	..T	..A	...

>>><<< **Exon 3**

	S	A	W	K	A	W	V	K	H	C	K	D	K	D	L	S	E	Y	L	A	S	C	N	L	*
Horse	TCT	GCC	TGG	AAG	GCC	TGG	GTA	AAA	CAC	TGC	AAG	GAC	AAG	GAT	TTG	TCC	GAA	TAC	CTG	GCT	AGC	TGT	AAC	CTG	TGA
Dog	...	...	...	GT.	...	...	..T	...	...	...	..A	.G.	..A	...	...	...	A..	...	...	..C	...	...	...	...	...
Cat	...	...	...	GT.	...	...	...	.G.	...	...	.GA	A..	...	...	...	...	AG.	..T	..A	..C	..T	...	..T	...	...
Human	...	...	...	GCA	...	...	...	...	...	...	..A	.C.	..A	...	...	..T	A..	...	...	A.C	...	...	..G	...	...
Chimpanzee	...	...	...	GCA	...	...	...	...	...	...	..A	.C.	..A	...	...	..T	A..	...	...	A.C	...	...	..G	...	...
Gorilla	...	...	...	GCA	...	...	...	...	...	...	..A	.C.	..A	...	...	..T	A..	...	...	A.C	...	...	..G	...	...
Orangutan	...	...	...	GCA	...	...	...	...	...	...	..A	.C.	..A	...	...	..T	A..	...	...	A.C	...	...	..G	...	...
Macaque	...	...	...	GT.	...	...	...	...	...	.A.	..A	.C.	..A	...	...	..T	A..	...	...	A.C	...	...	.CG	...	...
Baboon	...	...	...	GT.	...	...	...	...	...	.A.	..A	.C.	..A	...	...	..T	A..	...	...	A.C	...	...	.CG	...	...
Tarsier	...	...	...	GT.	...	...	.G.	...	...	...	..A	.G.	...	...	...	..T	A..	...	...	..G	..T	...	..G	...	...
Mouse lemur	.G.	...	...	.T.	.??	???	???	???	???	???	???	???	???	???	???	???	???	???	???	???	???	???	???	???	???
Shrew 1	AA.	...	...	T.T	...	...	..C	...	...	...	..A	.G.	...	...	..A	...	A.G	...	T..	...	G..	...	..G	..A	...
Shrew 2	AG.	...	...	GTT	.G.	...	..T	...	...	...	..A	.GA	..A	...	...	..T	A.G	...	T..	...	G..	..C	..T	...	...
Elephant	CT.	...	...	GCA	...	...	...	...	...	...	..A	..T	TT.	...	...	...	A..	C.T	T..	...	G.T	...	..G	...	...
Sloth	...	...	...	GTA	..A	.TT	...	..T	A.T	...	..A	.G.	...	...	...	...	A..	...	...	A.C	...	.A.	---	T.A	.A.

Exon 3 >>><<< **Exon 4** **Exon 4** >>>

Supplementary Figure 15. DNA sequences of calcium-binding lysozyme (*Lysc1*) genes from mammalian genomes. Alignment of the coding sequences of *Lysc1* gene sequences generated from an alignment generated by *MultiPipMaker* [42,43], see Additional file 15: Fig. S14. Sequences are compared to the horse *Lysc1* gene sequence, with identical bases indicated by dots. The predicted protein sequence of horse Lysc1 is shown above the sequence, with the signal peptide shown in italics and the N-terminal residue of the mature protein indicated as +1. The exon structure of the gene is shown below, with the arrow-heads (<<< or >>>) indicating the extent of each exon. Gaps that were introduced to maximize identity are shown as dashes (-). If a gap that was introduced to maximize identity was potentially due to missing sequence (*i.e.,* sequence assembly gap), then the missing bases are indicated as question marks (?).
