# Supplementary material for: Evolution of the mammalian lysozyme gene family
Source: BMC Evol Biol. 2011 Jun 15;11:166. doi: 10.1186/1471-2148-11-166 (PMC3141428; doi:10.1186/1471-2148-11-166)
Supplement: Additional file 17 — Supplementary Figure 16. This file is in PDF format. Phylogeny of Lysc1 genes. [file 1471-2148-11-166-S17.PDF]

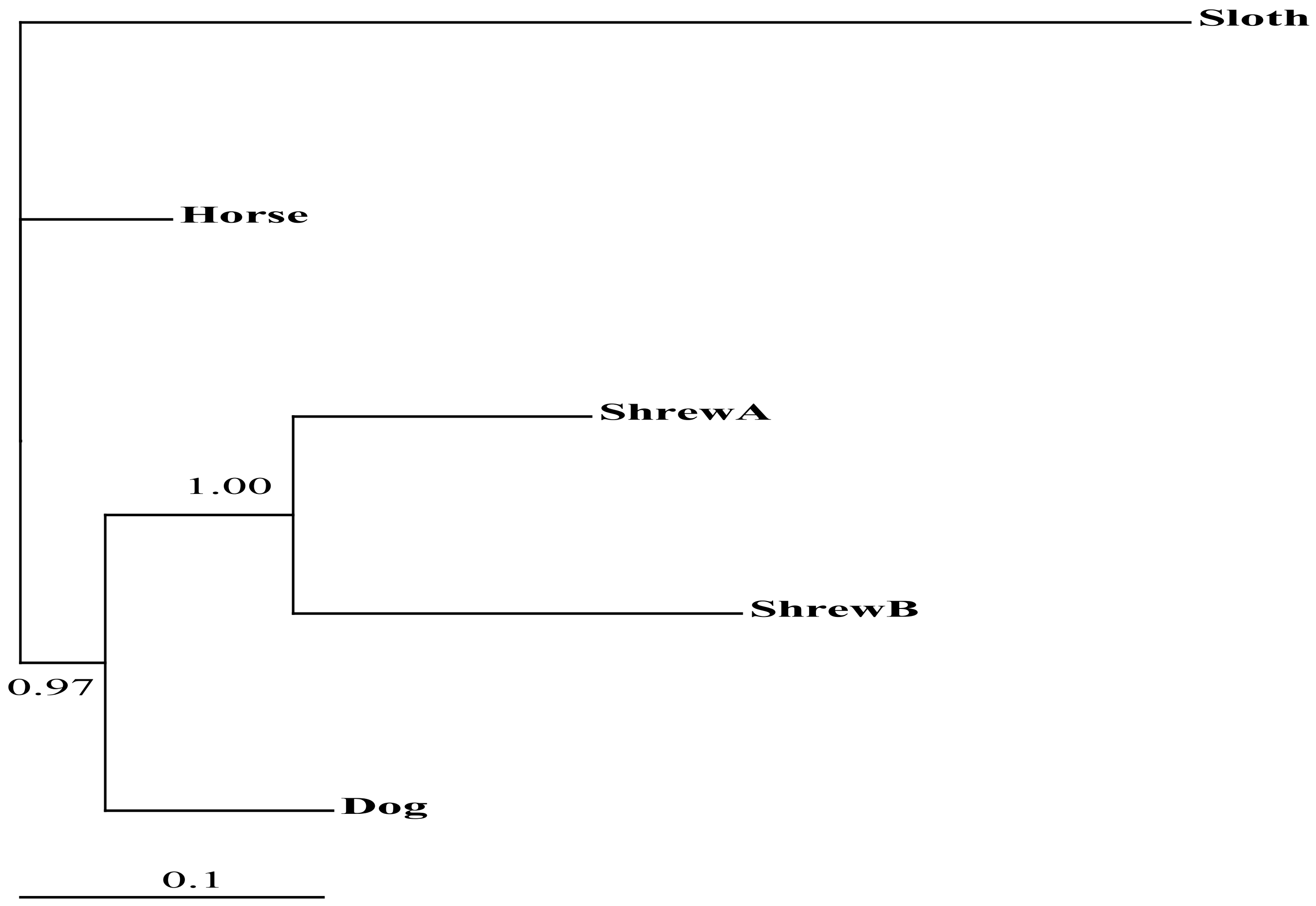

Supplementary Figure 16. Phylogeny of mammalian Calcium-binding Lysozyme (*Lysc1*) genes. A Bayesian phylogenetic tree of mammalian calcium-binding lysozyme genes was generated by *MrBayes* [60,61] using the DNA coding sequences of mammalian *Lysc1* sequences. This tree was built with nst=2 and rates=gamma as selected by *ModelTest* [66-68]. The tree was rooted with the sloth *Lysc1* sequence.
