# Supplementary material for: Evolution of the mammalian lysozyme gene family
Source: BMC Evol Biol. 2011 Jun 15;11:166. doi: 10.1186/1471-2148-11-166 (PMC3141428; doi:10.1186/1471-2148-11-166)
Supplement: Additional file 18 — Supplementary Table 2. This file is in PDF format. Outgroup lysozyme sequences used for phylogenetic analysis. [file 1471-2148-11-166-S18.PDF]

Supplementary Table 2. Outgroup lysozyme-like gene sequences.
Accession numbers, from *Ensembl* [16] or NCBI [49], of lysozyme sequences that were used to root the phylogeny of vertebrate lysozyme-like sequences (Figure 3, and Additional files 2 and 3: Figures S1 and S2).

**Species/Gene**	**Accession number**
Branchiostoma floridae	
Amphioxus_LyzA	AY175372.1
Amphioxus_LyzB	XM_002586710.1
Drosophila melanogaster	
*Drosophila*_LyzB	FBgn0004425
*Drosophila*_LyzP	FBgn0004429
*Drosophila*_Lyz S	FBgn0004430
*Drosophila*_Lyz X	FBgn0004431
Anopheles gambia	
*Anopheles*_Lyz	DQ007317
Bombix mori	
*Bombix*_Lyz	NM_001043983
