# Supplementary material for: Evolution of the mammalian lysozyme gene family
Source: BMC Evol Biol. 2011 Jun 15;11:166. doi: 10.1186/1471-2148-11-166 (PMC3141428; doi:10.1186/1471-2148-11-166)
Supplement: Additional file 19 — Supplementary Figure 17. This file is in Word format. FASTA formatted MAFFT alignment of lysozyme DNA sequences [file 1471-2148-11-166-S19.DOC]

>Human_Lyz

------------------------------------------------------------------------------------------ATGAAGGCT---------------------------------------------------CTCATTGTTCTGGGGCTT---GTC---CTC---CTTTCTGTTACG---------GTCCAGGGCAAGGTCTTTGAAAGGTGTGAGTTGGCCAGAACTCTGAAAAGATTGGGAATGGATGGCTACAGGGGAATCAGCCTAGCAAACTGGATGTGTTTGGCCAAATGGGAGAGTGGTTACAACACACGAGCTACAAACTACAATGCTGGAGACAGAAGCACTGATTATGGGATATTTCAGATCAATAGCCGCTACTGGTGTAATGAT------GGCAAA---ACCCCAGGAGCAGTTAATGCCTGTCATTTATCC------------TGCAGTGCTTTGCTGCAAGATAACATCGCTGATGCTGTAGCTTGTGCAAAG---------------

---AGGGTT---------GTCCGTGATCCA---CAAGGCATTAGAGCATGGGTGGCATGGAGAAATCGTTGTCAAAACAGAGAT---GTCCGTCAGTATGTTCAA------GGTTGTGGAGTG---------------------------------------------------------

---

>Gorilla_Lyz

------------------------------------------------------------------------------------------ATGAAGGCT---------------------------------------------------CTCATCGTTCTGGGGCTT---GTC---CTC

---CTTTCTGTTATG---------GTCCAGGGCAAGGTCTTTGAAAGGTGTGAGTTGGCCAGAACTCTGAAAAGATTGGGAATGGATGGCTACAGGGGAATCAGCCTAGCAAACTGGATGTGTTTGGCCAAATGGGAGAGTGGTTATAACACACGAGCTACAAACTACAATGCTGGAGACAGAAGCACTGATTATGGGATATTTCAGATCAATAGCCGCTACTGGTGTAATGAT------GGCAAA---ACCCCAGGAGCAGTTAATGCCTGTCATTTATCC------------TGCAGTGCTTTGCTGCAAGATAACATCGCTGATGCTGTAGCTTGTGCAAAG---------------

---AGGGTT---------GTCCGTGATCCA---CAAGGCATTAGAGCATGGGTGGCATGGAGAAATCATTGTCAAAACAGAGAT---GTCCGTCAGTATGTTCAA------GGTTGTGGAGTA---------------------------------------------------------

---

>Orangutan_Lyz

------------------------------------------------------------------------------------------ATGAAGGCT---------------------------------------------------CTCATTATTCTGGGGCTT---GTC---CTC

---CTTTCTGTTACA---------GTCCAGAGCAAGGTCTTTGAAAGGTGTGAGTTGGCCAGAACTCTGAAAAGATTGGGAATGGATGGCTACAGGGGAATCAGCCTAGCAAACTGGATGTGTTTGGCCAAATGGGAGAGTGGTTATAACACACGAGCTACAAACTACAATCCTGGAGACAGAAGCACTGATTATGGGATATTTCAGATCAATAGCCGCTACTGGTGTAATGAT------GGCAAA---ACCCCAGGAGCAGTTAATGCCTGTCATTTATCC------------TGCAGTGCTTTGCTGCAAGATAACATCGCTGATGCTGTAGCTTGTGCAAAG---------------

---AGGGTT---------GTCCGTGATCCA---CAAGGCATTAGAGCATGGGTGGCATGGAGAAATCGTTGTCAAAACAGAGAT---GTCCGTCAGTATGTTCAA------GGTTGTGGAGTA---------------------------------------------------------

---

>Macaque_Lyz

------------------------------------------------------------------------------------------ATGAAGGCT---------------------------------------------------GTCATTATTCTGGGGCTT---GTC---CTC

---CTTTCTGTCACG---------GTCCAGGGCAAGATCTTTGAAAGGTGTGAGTTGGCCAGAACTCTGAAAAGATTGGGACTGGATGGCTACAGGGGAATCAGCCTAGCAAACTGGGTGTGTTTGGCCAAATGGGAGAGTAATTATAACACACAAGCTACAAACTACAATCCTGGAGACCAAAGCACTGATTATGGGATATTTCAGATCAATAGCCACTACTGGTGTAATAAT------GGCAAA---ACCCCAGGAGCAGTTAATGCCTGTCATATATCC------------TGCAATGCTTTGCTGCAAGATAACATCGCTGATGCTGTAACTTGTGCAAAG---------------

---AGGGTT---------GTCAGTGATCCA---CAAGGCATTAGAGCATGGGTGGCATGGAGAAATCACTGTCAAAACAGAGAT---GTCAGTCAGTATGTTCAA------GGTTGTGGAGTG---------------------------------------------------------

---

>Marmoset_Lyz

------------------------------------------------------------------------------------------ATGAAGGTT---------------------------------------------------CTCATTATTCTGGGGCTG---GTC---CTC

---CTTTCTGTCATG---------GTCCAGGGCAAGGTCTTTGAAAGGTGTGAGTTGGCCAGAACTCTGAAAAGGTTTGGACTGGATGGCTACAGGGGAATCAGCCTAGCAAACTGGATGTGTTTGGCCAAATGGGAGAGTGATTATAACACACGTGCTACAAACTACAATCCTGGAGACCAAAGCACTGATTATGGGATATTTCAGATCAATAGCCACTATTGGTGTAACAAT------GGCAGA---ACCCCAGGAGCAGTTAATGCCTGTCATATATCC------------TGCAATGCTTTGCTGCAAGATGACATCACTGAAGCTGTGGCCTGTGCAAAG---------------

---AGGGTT---------GTCCGCGATCCA---CAAGGCATTAGGGCATGGGTGGCATGGAAAGCTCATTGTCAAAACAGAGAT---GTCAGTCAGTATGTTCAA------GGTTGTGGAGTA---------------------------------------------------------

---

>Squirrel_Lyz

------------------------------------------------------------------------------------------ATGAAGGCT---------------------------------------------------TTTCTGATTCTGGGGCTT---CTT---CTC

---CTTTCTGTCACA---------GTCCAGGGCAAGGTCTATGGAAGGTGTGAGTTGGCCAGAACTCTAAAAAGACTTGGAATGGATGGCTACCATGGAATCAGCTTGGCAAACTGGGTGTGTCTGGCCAAATGGGAGAGTAGTTACAACACCAGAGCTACAAACTACAACCCTGGAGAC

CAAAGTACTGACTATGGGATATTCCAGATCAATAGCCACTACTGGTGCAATGAC------GGCAAA---ACCCCAGGAGCAGTTAATGCCTGTGGTATATCC------------TGCAATGCTCTGTTGCAAGATGACATCTCTCAAGCCGTAACTTGTGCAAAA---------------

---AGGGTT---------GTCCGTGATCCA---CAAGGCATTAGAGCATGGGTGGCATGGAGAAACCATTGTCAAAACCGTGAC---CTCACTCAGTACATTCAG------GGGTGTGGAGTG---------------------------------------------------------

---

>Hyrax_LyzB

------------------------------------------------------------------------------------------ATGAAGGCT---------------------------------------------------CTCCTTATCCTGGGGCTT---CTC---TCC

---CTTTCCCTCACT---------GCCCAGGGCAAGGTCTATGAAAGATGCGAGCTGGCCCGAACTCTGAAACGACAGGGAATGGCTGGCTACAGGGGAGTCAGCCTGGCAAACTGGGTGTGTTTGGCCAAGTGGGAGAGTAGTTACAACACCAAAGCTACAAACTACAATCCTGGAGAC

AGAAGCACCGATTACGGGATTTTTCAGATCAATAGCCGCTGGTGGTGTAATGAC------GGCAAA---ACCCCAGGAGCAGTCAACGCCTGTGGCATACCT------------TGCAGTGTTTTGCTGCAAGATGATATCACTCAAGCTATAACTTGTGCGAAG---------------

---AGGGTT---------GTCAGAGATCCA---CAAGGCATTAGAGCATGGGTGGCATGGAGAAACCATTGTCAGAACCGAGAT---CTCACTCCATACATTCAA------GGTTGTGGAGTT---------------------------------------------------------

---

>Kangaroo_rat_LyzB

------------------------------------------------------------------------------------------ATGAAGGCT---------------------------------------------------CTCCTTATTCTGGGCCTT---TTT---CTA

---CTTTCTAATACT---------GTCCAGGGCAAGATTTATGGAAGATGTGAGTTGGCCAGAGCTCTAAAGAAACTTGGAATGGATGGCTACAAGGGAATCAGCCTGGCAAACTGGATGTGTTTGGCCAAATGGGAGAGCAGTTATAACACAAAAGCTACAAACTACAATCCTGGAGAC

AAAAGCACTGATTATGGGATATTTCAGATCAATAGCCGCTATTGGTGCAATAAT------GGCAAG---ACCCCAAGAGCAGTTAATGCCTGTCACATATCC------------TGCAATGCCTTGTTGCAAGATAACATCTCTCAAGCTGTAGCTTGTGCAAAG---------------

---AGGGTT---------GTCAGAGATCCA---CAAGGCATTAGAGCATGGATGGCCTGGAGATCGCATTGCCAAAACCGAAAT---CTCTCTGAGTATGTTCGG------GGCTGTGGTGTG---------------------------------------------------------

---

>Rabbit_LyzA

------------------------------------------------------------------------------------------ATGAAGGCA---------------------------------------------------CTCCTGATCCTGGGGCTT---CTC---CTC

---CTTTCCACCACT---------GTCCAGGGCAAGATCTATGAAAGGTGTGAGTTGGCCAGAACTCTGAAAAAACTTGGATTGGATGGCTACAAGGGAGTCAGCTTGGCGAACTGGATGTGTTTGACCAAATGGGAGAGCAGTTATAACACGCAAGCTACAAACTACAACCCTGGAGAC

AAAAGCACCGATTATGGAATATTTCAGATCAATAGCCGCTACTGGTGTAACGAT------GGCAAA---ACCCCACGAGCAGTTAACGCCTGCCATATACCC------------TGCAGTGATTTATTGAAAGATGACATCACTCAAGCTGTAGCTTGTGCAAAG---------------

---AGGGTC---------GTCAGCGATCCA---CAAGGCATCAGAGCATGGGTGGCGTGGAGAAATCATTGTCAAAGCCAAGAT---CTCACTTCGTATATTCAG------GGCTGTGGAGTG---------------------------------------------------------

---

>Rabbit_LyzB

------------------------------------------------------------------------------------------ATGAAGGCA---------------------------------------------------CTCCTGATCCTGGGGCTT---CTC---CTC

---CTTTCCACCACT---------GTCCAGGGCAAGATCTATGAAAGGTGTGAGTTGGCCAGAACTCTGAAAAAACTTGGATTGGATGGCTACAAGGGAGTCAGCTTGGCGAACTGGATGTGTTTGACCAAATGGGAGAGCGGTTATAACACGCAAGCTACAAACTACAACCCTGGAGAC

AAAAGCACCGATTATGGAATATTTCAGATCAATAGCCGCTACTGGTGTAACGAT------GGCAAA---ACCCCACGAGCAGTTAACGCCTGCCATATACCC------------TGCAGTGATTTATTGAAAGATGACATCACTCAAGCTGTAGCTTGTGCAAAG---------------

---AGGGTC---------GTCAGCGATCCA---CAAGGCATCAGAGCATGGGTGGCGTGGAGAAATCATTGTCAAACC---GAT---CTCACTTCGTATATTCAG------GGCTGTGGAGTG---------------------------------------------------------

---

>Treeshrew_Lyz

------------------------------------------------------------------------------------------ATGAAGGCC---------------------------------------------------CTCCTTGTTCTGGGGCTT---CTG---CTC

---CTTTCCGTTACT---------GTGCAGGGCAAGATCTATGAAAGGTGTGAGTTGGCCCGAACTATGAAAAGATTTGGATTGGATGGCTTTGCGGGAATCAGCCTGGCCAATTGGATGTGTTTGGCCAAATGGGAGAGTGATTATGACACACGAGCTACAAACTACAATCCTGGAGAC

CGAAGCACTGATTATGGGATATTCCAGATCAACAGCCGGTACTGGTGTAACGAT------GGCAAA---ACCCCGAGAGCAGTGAACGCCTGTGGCATATCC------------TGCAATGCTTTGCTACAAGATGACATCTCTCAAGCTGCAGCGTGTGCCAAG---------------

---AGGGTT---------GTCAAGGATCCA---CAAGGCGTTAGAGCGTGGGTGGCATGGAGGAACCGTTGTCAAAACCAAGAT---GTCTCCCAGTATGTGAGG------GGTTGTGGCGTG---------------------------------------------------------

---

>Elephant_LyzA

------------------------------------------------------------------------------------------ATGAAGGCT---------------------------------------------------CTCCTTATTCTGGGCCTT---CTC---TTC

---CTTTCTGGCACT---------GCCCAGGGCAAGGTCTTTGAAAGGTGCGAGTTGGCCAGAACTCTGAAACGGTATGGAATGGATGGCTTCAGGGGGATCAGCCTGGCAAACTGGATGTGTTTGACCAAATGGGAGAGTGATTACAACACCCAAGCTACAAACTACAATCCTGGAGAC

AAAAGCACTGATTACGGGATTTTTCAGATCAATAGCCACTACTGGTGTAATGAT------GGCAAA---ACCCCAAGAGCAGTTAACGCCTGTGGCATATCC------------TGCTACGCTTTGCTGCAAGATGATATCACTCAAGCTGTAGCTTGTGCGAAA---------------

---AGGGTT---------GTCAGTGATCCA---CAAGGCATTAGAGCATGGGTGGCATGGAGAAACCATTGTCAGAACCACGAT---GTCACTCAGTACATTCAA------GGCTGTGGAGTC---------------------------------------------------------

---

>Dolphin_Lyz

------------------------------------------------------------------------------------------ATGAAGGCT---------------------------------------------------GTCCTTATTCTGGGGCTT---CTC---CTC

---CTTTCTGTCGCT---------GTCCAGGGCAAGACCTTTGAGAGGTGTGAGCTTGCCAGAACTCTGAAAAGACTTGGATTGGATGGCTATAGGGGAGTCAGCCTGGCAAACTGGATGTGTTTGGCCAAATGGGAAAGCAATTATAACACACAAGCTACAAACTACAATCCTGGAAGC

CGAAGCACTGACTATGGGATATTTCAAATCAATAGCCGCTACTGGTGTAATGAT------GGCAAA---ACCCCAAGAGCACATAACGCCTGTGGCATACCC------------TGCAGTGCGTTGCTGAAAGATGACATCACTGATGCTGTAAGATGTGCAAAG---------------

---AGGGTT---------GTCAGGGATCCA---CAAGGCATTAGAGCATGGGTGGCATGGAGAAGTCACTGTCAAAACCAAGAT---CTCAGGACATATGTTCAG------GGTTGCGGAGTG---------------------------------------------------------

---

>Guinea_pig_LyzI

------------------------------------------------------------------------------------------ATGAAGGCT---------------------------------------------------TTCCTTATTCTGGGGCTG---CTC---CTC

---CTTTCTGTCCCC---------ATCCATGGGAAGATCTTTGAAAGGTGTGAGCTTGCCAGAGCTCTGAAGAGATATGGACTGGATGGCTACCGGGGAATCAGCCTGGCAAACTGGGTGTGCTTAGCCAGATGGGAGAGTAGCTATAACACACGAGCTACAAACTACAATCCTGGAGAC

AGAAGCACCGATTATGGGATATTCCAGATCAATAGCCGCTACTGGTGCAATGAT------GGCAAA---ACCCCGAGAGCTGTTAATGGCTGTGGTATATCC------------TGCAACGCTTTGCTGCAAGATGACATCACTCAGGCTGTGAACTGTGCCAAG---------------

---AGGGTT---------GTGAGAGATGCA---CAAGGCATCAGAGCATGGGTGGCGTGGAGGAATCGGTGTCAAAACCATGAT---CTCACCGAGTATGTGCGC------GGCTGTGGAGTT---------------------------------------------------------

---

>Elephant_LyzB

------------------------------------------------------------------------------------------ATGAAGGCT---------------------------------------------------CTCCTTATTCTGGGGCTT---CTC---TTC

---CTTTGTGGCACT---------GTCCACGGTAAGGTCTTTAAAAGGTGCGAGTTGGCCCGAACTCTGAAGCGACATGGACTGGATGGCTACAGGGGAATCAGCCTGGCAAACTGGGTGTGTTTGGCCAAACATGAGAGTAATTACAAGACCCGAACTACAAACTATAATCCTGGAGAC

AAAAGCACTGATTACGGGATTTTTCAGATCAATAGCCGCTACTGGTGTAATGAT------GGCAAA---ACCCCAAACGCAGTTAATGCCTGTGGCATATCT------------TGCTACGATTTACTGCAAGGTGATATCACTCAAGCTGTAGCTTGTGCAAAG---------------

---AGAGTT---------GTCAGGGATCCA---CAAGGCATTAAAGCATGGGTAGCATGGAGAAAACATTGTCAGAACCGTGAT---CTCACTCAGTATGTTCGA------GGCTGTGGGGTT---------------------------------------------------------

---

>Elephant_LyzC

------------------------------------------------------------------------------------------ATGAGGGCT---------------------------------------------------CTCCTTATTCTGGGGCTT---CTC---TTC

---CTTTCTGGCACT---------GTCCATGGCAAGGTCTTTGAAAGGTGCGAGTTGGCCCGAACTCTGAAGCGACATGGACTGGGTGGCTACAGAGGAATCAGCCTGGCAAACTGGGTGTGTTTGGCCAAACATGAGAGTAATTACAACACCCGAGCCACAAACTATAATCCTCCAGAC

AAAAGCACTGATTATGGGATTTTTCAGATCAATAGCCGCTACTGGTGTAATGAT------GGCAAA---ACCCCAAGAGCAGTTAATGCCTGTGGCATATCT------------TGCAAAGCTTTGCTGCAAGATGATATCACTCAAGCTATAGCTTGTGCGAAG---------------

---AGAGTC---------GTCAGGGATCCA---CAAGGCATTAAAGCATGGGTGGCGTGGAGAAACCATTGTCAGAACCACGAT---CTCACTCAGTACGTTCGA------GGCTGTGGGGTT---------------------------------------------------------

---

>Cow_LyzA_Milk

------------------------------------------------------------------------------------------ATGAAGGCT---------------------------------------------------CTCCTTATTGTGGGGCTT---CTC---CTC

---CTTTCTGTTGCT---------GTCCAGGGCAAGAAATTTCAGAGGTGTGAGCTTGCCAGAACTCTGAAGAAACTTGGATTGGATGGCTATCGAGGAGTCAGCCTGGCAAACTGGGTGTGTTTGGCCAGATGGGAAAGCAATTACAACACACGTGCTACAAACTACAATCGTGGAGAC

AAAAGCACTGATTATGGGATATTTCAAATCAATAGCCGCTGGTGGTGCAATGAT------GGCAAA---ACCCCAAAAGCAGTTAACGCCTGTCGTATACCC------------TGCAGCGCTTTGCTGAAAGATGACATCACTCAAGCTGTAGCATGTGCAAAG---------------

---AGGGTT---------GTCAGAGATCCA---CAAGGCATTAAAGCATGGGTGGCATGGAGAAACAAGTGTCAAAACCGAGAT---CTCAGGAGTTATGTTCAG------GGTTGCAGAGTA---------------------------------------------------------

---

>Horse_Lyz

------------------------------------------------------------------------------------------ATGAAGGTT---------------------------------------------------CTCCTGACTCTGGGGCTT---CTC---CTG

---CTGTCTGTCACT---------GTCCAGGGCAAGGTCTTTGAAAGATGTGAGCTGGCCAGAACGCTGAAAAGACTTGGACTGGACGGCTTTAGGGGAGTCAGCCTGCCAAACTGGGTCTGTTTGGCCAGATGGGAAAGTAATTACAACACACGAGCTACAAACTACAATCCTGGAAGT

CAAAGCACTGATTATGGGATATTTCAGATCAACAGCCGCTACTGGTGCAATGAT------GGCAAA---ACCCCACGAGCAGTGAATGCCTGTCGGATTCCC------------TGCAGCGCTCTGCTGCAAGATGACATCACTCAAGCCGTGGCGTGTGCAAAG---------------

---CGGGTG---------GTCAGTGACCCA---CAGGGCATCCGAGCATGGGTGGCGTGGAGAAATCGTTGTCAGAACCACGAC---CTCACTCAGTATGTCAAG------GGTTGCAGAGTG---------------------------------------------------------

---

>Dog_Lyz

------------------------------------------------------------------------------------------ATGAAGACT---------------------------------------------------CTCCTTTTCCTTGGGCTC---CTC---CTC

---CTTTCCATCACC---------GTCCAGGGCAAGATCTTTGAAAGGTGTGAGCTGGCCAGAACTCTGAAAAACCTTGGGCTGGCTGGCTATAAGGGCGTCAGCCTGGCAAACTGGGTGTGTTTGGCCAAGTGGGAAAGTAATTATAATACAAGGGCTACGAACTACAATCCCGGAAGC

AAAAGCACTGATTATGGGATATTTCAAATCAATAGCCGCTACTGGTGTAATGAT------GGCAAA---ACGCCCAGAGCAGTGAACGCCTGTCACATATCC------------TGCAGCGCTTTGCTGCAAGATGACATCACTCAAGCCGTAGCATGTGCAAAG---------------

---AGGGTT---------GTCAGTGATCCA---AATGGCATTCGAGCATGGGTGGCATGGAGAGCACACTGTGAAAACCGAGAT---GTCTCTCAGTATGTTCGG------AATTGTGGAGTG---------------------------------------------------------

---

>Guinea_pig_LyzC

------------------------------------------------------------------------------------------ATGAAGGCT---------------------------------------------------CTCCTCCTTCTGGGGCTT---CTC---GTC

---CTTTCTGTGACT---------GTCCAGGGCAAGGTCTATAAGAGGTGTGAGTTGGCCAGAACTTTAAAGCGTCTTGGAATGGACGGCTACCGTGGGGTCAGCCTGGCAAACTGGATGTGTTTGATCAAATGGGAGAGTAATTACAATACAAGAGTTAAAAACTACAATCCTGAAAGC

AAAAGCACTGATTATGGGATATTCCAGATCAATAGTCGATACTGGTGTAACGAT------GGAAAA---ACCCCAGGAGCAGTGAATGGCTGTGGCATATCC------------TGCAACGTTTTGCTACAAAATGACATCACTAAAGCGGTGGCTTGTGCAAAG---------------

---AGGGTG---------GTCAGGGATCCA---CAAGGCATCAGAGCATGGGTGGCATGGAGAAACCGTTGTCAAAATCAAGAT---GTTTCTCTGTATGTTAGG------GGCTGTAGAGTA---------------------------------------------------------

---

>Shrew_Lyz

------------------------------------------------------------------------------------------ATGAAGGCT---------------------------------------------------CTCCTCATTCTGGGGCTT---GTC---CTC

---CTGTGTGTCACT---------GTCCAGGGCAAGGTCTTTGAACGATGTGAACTGGCCAGGACTCTGAAAAGACTCGGAATGGATGGCTACCGGGGAGTCAGCCTGGCAAACTGGATGTGTTTGACCAAGTGGGAAAGTGATTACAACACTCGAGCCACAAACTTCAACCCTGGAAGC

GGGAGCACTGACTATGGGATATTTCAGATCAACAGTCGCTATTGGTGTAACGAT------GGCAAG---ACCCCTGGGGCCAAGAACGCCTGCGGCATCTCC------------TGCAGCGTTTTGCTGAAAGATGACATCACTCAAGCTGTAGCATGTGCAAAG---------------

---AGAGTC---------GTCAGGGACCCA---CAAGGCATTCGAGCCTGGGTGGCATGGAGAAACCACTGTCAGAACAGAGAT---GTGTCTCAGTATGTGCGG------GGTTGTAGAGTC---------------------------------------------------------

---

>Guinea_pig_LyzE

------------------------------------------------------------------------------------------ATGAAGGCT---------------------------------------------------CTCCTCCTTCTGGGGCTT---CTC---GTC

---CTGTCTGTGACT---------GTCCAGGGCAAGGTCTATGAGAGGTGTGAGTTGGCTAGAACTTTAAAGCGTCTTGGAATGGACGGCTACCATGGAGTCAGCCTGGCAAACTGGATGTGTTTGATCAAATGGGAGAGTGATTATAATACAAAAGCTACAAACTACAACCCTGGAGAT

GAAAGCACTGATTATGGGATATTCCAGATCAATAGTCGATACTGGTGCAACAAT------GGCAAA---ACCCCAGATGCAGTTGATGCCTGTCATATATCC------------TGCAGTGTCTTGTTGGAAGATAACATCGCCCAAGCTGTGGCTTGTGCAAAG---------------

---AGGGTG---------GTCAGGGATCCA---CAAGGCATCAGAGCATGGATGGGATGGAGAAATCACTGTGAAAATCGAGAT---GTTTCTCAGTATGTTAGC------GGCTGTGGAGTA---------------------------------------------------------

---

>Fox_bat_Lyz

------------------------------------------------------------------------------------------ATGAAGGCT---------------------------------------------------CTCCTTATTCTGGGGCTT---CTC---CTC

---CTTTCTGTAACC---------ATCCAGGGCAAGGTCTATGAAAGGTGCGAGTTTGCCAGAACTCTGAAAAGACATGGAATGGATGGCTTCAAGGGAGTGAGCCTGGCAAACTGGGTGTGTTTGGCCAAATGGGAAAGTAGTTACAACACACGAGCTACCAACTACAATCCTGGAGAC

AAAAGCACTGATTACGGAATATTTCAGATCAATAGCCACTACTGGTGTAATGAC------GGCAAA---ACTCCACGAGCAGTTAATGGCTGTCATGTATCC------------TGCAAGGCTTTGATGAATGATGACATTACTCAAGCCATAGCATGTGCAAAG---------------

---ACGGTG---------GTCAGTGATAGA---CAAGGCATCAGAGCATGGGTGGCATGGAGAAATCATTGTCAAAACAGAGAT---CTCACTCAGTATACCCAA------GGTTGTGGAGTG---------------------------------------------------------

---

>Cow_LyzL_Kidney

------------------------------------------------------------------------------------------ATGAAGGCT---------------------------------------------------CTCCTCATTCTGGGGCTT---CTC---CTC

---TTTTCGGTCGCT---------GTCCAAGGCAAGGTCTTTGAGAGATGTGAGCTTGCCAGAAGTCTGAAAAGATTTGGAATGGATAACTTTAGGGGAATCAGCCTGGCAAACTGGATGTGTTTGGCCAGATGGGAAAGCAATTACAACACACAAGCTACAAACTACAATGCTGGAGAC

CAAAGCACTGATTATGGGATATTTCAAATCAATAGCCACTGGTGGTGCAATGAT------GGCAAA---ACCCCAGGAGCAGTTAACGCCTGTCATTTACCC------------TGCGGTGCTTTGCTGCAAGATGACATCACTCAAGCTGTAGCATGTGCAAAG---------------

---AGGGTT---------GTCAGTGATCCA---CAAGGCATTAGAGCATGGGTGGCATGGAGAAGTCATTGTCAAAACCAAGAT---CTCACGAGTTACATTCAG------GGTTGTGGAGTG---------------------------------------------------------

---

>Little_brown_bat_Lyz

------------------------------------------------------------------------------------------ATGAAGGCT---------------------------------------------------CTCCTGACTCTGGGGCTT---CTC---CTC

---CTCTCTGTCGCC---------GTCCGGGCCAAGGTCTATGAGAGGTGTGACCTGGCCAGAACCTTGAAAAGACTTGGAATGGATGGCTACAGTGGAGTGAGCCTGGCAAACTGGATGTGTTTGGCCAAATGGGAAAGTAGTTACAACACCCGAGCTACAAACAACAATCCTGGAGAC

AAAAGCACGGATTATGGGATATTCCAGATCAATAGCCGCTACTGGTGTAATGAT------GGCAAA---ACCCCAGGAGCAGTTAATGCCTGTGGTATACCC------------TGCAACGATTTGCTTCAAGATGACATCACTAAAGCTGCAACCTGTGCCAAG---------------

---AGGGTT---------GTCAAGGATCCA---CGAGGCATCAAAGCATGGGTGGCCTGGAGAAATCACTGTCAAAACAAAGAT---GTCACTCAGTATACCCGG------GGCTGTGGAGTG---------------------------------------------------------

---

>Tenrec_LyzA

------------------------------------------------------------------------------------------ATGAAGGCG---------------------------------------------------CTCCTTATTCTGGGGTTT---CTC---TTC

---CTTTTGGTCACC---------ATCCAGGGAAAGGTCTATGAAAGATGTGAACTAGCCAGAAGGATGAAACAACTTGGAATGGATGGCTACGAGAGAATTAGCTTGGCAAACTGGATGTGCTTGATCAAATGGGAGAGTGGTTATAATACCCGAGTTACAAACTACAATCGTGGTGAC

AAAAGCACAGACTATGGAATTTTTCAGATCAATAGCCGCTACTGGTGCAATGAT------GGCAAA---ACCCCAAGAGCAGTTAATGCCTGCCGCATATCC------------TGCAACGTTTTGCTACAAGATGACATCACTCAAGCTGTAGCATGTGCGAAG---------------

---AGGGTT---------GTCCGTGATCCA---CAGGGCATTAGAGCATGGGTGGCTTGGAGAAACCGTTGTCAGAACCGGGAT---CTCACTGAGTATGTACGA------GGCTGTGGCGTC---------------------------------------------------------

---

>Mouse_LyzM

------------------------------------------------------------------------------------------ATGAAGGCT---------------------------------------------------CTCCTGACTCTGGGACTC---CTC---CTG

---CTTTCTGTCACT---------GCCCAGGCCAAGGTCTACAATCGTTGTGAGTTGGCCAGAATTCTGAAAAGGAATGGAATGGATGGCTACCGTGGTGTCAAGCTGGCTGACTGGGTGTGTTTAGCTCAGCATGAGAGCAATTATAACACACGAGCTACAAACTACAACCGTGGAGAC

CGAAGCACCGACTATGGGATATTTCAGATCAATAGCCGATACTGGTGTAATGAT------GGCAAA---ACCCCAAGATCTAAGAATGCCTGTGGGATCAAT------------TGCAGTGCTCTGCTGCAGGATGACATCACTGCAGCCATACAATGTGCAAAG---------------

---AGGGTG---------GTGAGAGATCCC---CAAGGCATTCGAGCATGGGTGGCATGGCGAACACAATGTCAAAACCGAGAT---CTGTCCCAGTATATTCGG------AACTGCGGAGTC---------------------------------------------------------

---

>Mouse_LyzP

------------------------------------------------------------------------------------------ATGAAGACT---------------------------------------------------CTCCTGACTCTGGGACTC---CTC---CTG

---CTTTCTGTCACT---------GCTCAGGCCAAGGTCTATGAACGTTGTGAGTTTGCCAGAACTCTGAAAAGGAATGGAATGGCTGGCTACTATGGAGTCAGCCTGGCCGACTGGGTGTGTTTAGCTCAGCACGAGAGCAATTATAACACACGAGCTACAAACTACAACCGTGGAGAC

CAAAGCACTGACTATGGGATATTTCAGATCAATAGCCGATACTGGTGTAATGAT------GGCAAA---ACCCCAAGAGCTGTGAATGCCTGTGGGATCAAT------------TGCAGTGCTCTGCTGCAGGATGACATCACTGCAGCCATACAATGTGCAAAG---------------

---AGGGTG---------GTGAGAGATCCC---CAAGGCATTCGAGCATGGGTGGCATGGCGAGCACACTGTCAAAACCGAGAT---CTGTCCCAGTATATTCGG------AACTGCGGAGTC---------------------------------------------------------

---

>Rat_Lyz

------------------------------------------------------------------------------------------ATGAAGGCT---------------------------------------------------CTCCTAGTTCTGGGGTTC---CTC---CTG

---CTCTCTGCCTCT---------GTCCAGGCCAAGACCTATGAACGCTGTGAGTTCGCCAGAACTCTGAAAAGGAATGGGATGTCTGGCTACTATGGAGTCAGCCTGGCAGACTGGGTGTGTTTAGCTCAGCATGAGAGCAATTATAACACACAAGCCAGAAACTACAACCCTGGAGAC

CAAAGCACCGACTATGGGATATTTCAGATCAATAGCCGATACTGGTGTAATGAC------GGCAAA---ACCCCAAGAGCAAAGAACGCCTGTGGGATACCC------------TGCAGCGCTCTGCTGCAGGATGACATCACTCAAGCCATACAATGTGCGAAG---------------

---AGAGTT---------GTGAGGGATCCC---CAAGGCATTCGAGCATGGGTGGCATGGCAAAGACACTGTAAAAACCGAGAT---CTATCCGGGTATATTCGG------AACTGTGGAGTC---------------------------------------------------------

---

>Guinea_pig_LyzB

------------------------------------------------------------------------------------------ATGAAGGCT---------------------------------------------------CTCCTCCTTCTGGGGCTT---CTC---ATC

---CTTTCTGTGACT---------GTCCAGGGCAAGGTCTTTAAAAGATGTCAATTGGCCAGAACTTTAAAGCGTCTTGGAATGGACGGCTACCATGGGATCAGCCTGGCAAACTGGACGTGTTTGGTCAGATGGGAGAGTAAATATAATACAAAAGCTACAAAGTACAATCCTGGAAAC

AAAGGCACTAATTATGGAATATTCCAGATCAATAGTCGATCCTGGTGTGAGGAT------GGAAAA---ACCCCAGGAGCAGTGAATGGCTGTGGCATATCC------------TGCAACGTTTTGTTGCAAAATGACATCACTAAAGCGGTGGCTTGTGCAAAG---------------

---AAGGTG---------GTCAAGGATCCA---AAAGGCATCAAAGAATGGGAGGCATGGAAAAACCATTGTCAAAAGCAAGAT---GTTTCTCAGTATCTTAGG------GGCTGTCAAGTG---------------------------------------------------------

---

>Pig_Lyz

------------------------------------------------------------------------------------------ATGAAGACT---------------------------------------------------CTCCTCGTTCTTGCGCTT---CTC---CTC

---CTTTCTGTCTCT---------GTACAGGCCAAGGTCTATGATCGGTGCGAGTTCGCCAGAATTCTGAAAAAGTCTGGAATGGACGGCTATAGGGGAGTCAGCCTGGCGAACTGGGTATGTTTGGCCAAGTGGGAAAGTAATTTTAACACAAAAGCTACAAACTACAATCCTGGAAGC

CAAAGCACTGATTATGGAATATTTCAAATTAATAGCCGATACTGGTGTAATGAC------GGCAAG---ACACCCAAAGCAGTTAATGCCTGTCACATATCC------------TGCAAAGTTTTGCTGGACGATGACCTCAGTCAAGATATAGAATGTGCAAAG---------------

---AGGGTT---------GTCAGAGATCCA---CAAGGCATTAAAGCATGGGTGGCATGGAAAGCTCATTGTCAGAACAAAGAT---GTCTCGCAGTACATTCGG------GGTTGCAAACTG---------------------------------------------------------

---

>Alpaca_LyzA

------------------------------------------------------------------------------------------ATGAAGGCT---------------------------------------------------CTCCTTGTGCTGGGGCTC---GTC---CTC

---CTCGCTGTGGCT---------GTGCAGGGCAAGGTCTGGGAGAGATGCACGCTGGCCCGAAAGCTGAAGGAACTCGGAATGGACGGCTACAGGGGAGTCAGCCTGGCCAACTGGATGTGTTTGGCCAAATGGGAAAGTAGTTATAACACAAAAGCTACAAACTACAACCCCCCCAGC

AAAAGCACCGATTACGGGATATTCCAGATCAACAGCCGCTACTGGTGTGACGAT------GGCAAA---ACTCCAAAAGCAGTGAACGGCTGTGGCATCAAA------------TGTAACGTTTTGCTGCAAGATGACATCACCAAAGCCATACAATGTGCAAAG---------------

---AGGGTT---------GTCAGGGACCCA---CAAGGCGTTAGAGCATGGGTGGCATGGAAAAACCACTGTGAGGGCCACGAT---GTGGAGCAGTATATTGAG------GGCTGTGATCTG---------------------------------------------------------

---

>Tarsir_Lyz

------------------------------------------------------------------------------------------ATGAAGGCT---------------------------------------------------CTCCTAATCCTGGGGCTT---GTC---CTT

---CTTTCTGTCACA---------GTCCAGGGCAAGAAATGGGAAAGGTGTGCGCTGGCCAGAGAACTAAAAGCAAAAGGAATGGATAACTACGCAGGAGTCAGCCTAGCGGATTGGATGTGTTTGTCCAAATGGGAGAGTGATTATAACACACGAGCCACAAACTACAACCCTGGAGAC

AAAAGCACTGATTATGGGATATTTCAGATCAATAGCCACTACTGGTGTGAGGAT------GGCAAA---ACTCCAAAATCAGTGAATGCCTGTGGAATACCC------------TGCAGTGCTTTGCTGCAAGATGACATCACTCAGGCCGTGACTTGTGCAAAG---------------

---AGGGTT---------GTCAGAGATCCA---CAAGGCATTAAAGCATGGGTGGGATGGAAACGTCATTGTCAAAACAAAGAT---CTCAGAAGCTACATTCGG------GGTTGTGGAGTG---------------------------------------------------------

---

>Cow_LyzB

------------------------------------------------------------------------------------------ATGAAGGCT---------------------------------------------------CTCCTTATTCTGGGGCTT---CTG---CTC

---CTTTCTGTTGCT---------GTCCAGGGCAAGACATTTGAGAGGTGTGAGCTTGCCAGAACTCTGAAGAATCTTGGATTGGCTGGCTACAAGGGAGTCAGCCTGGCAAACTGGATGTGTTTGGCCAAAGGAGAAAGCGGTTATAACACACAAGCTAAAAACTATAGTCCTGGATTC

AAAAGCACTGATTATGGGATATTTCAGATCAACAGCAAATGGTGGTGTAATGAT------GGCAAA---ACCCCAAAAGCAGTTAACGGCTGTGGTGTATCC------------TGCAGTGCTTTGCTGAAAGATGACATCACTCAAGCTGTAGCATGTGCAAAG---------------

---AAGATT---------GTCAGTCAG------CTAGGCCTTACAGCATGGGTGGCATGGAAAAACAAGTGTCAAAACCGAGAC---CTCACGAGTTATGTTCAG------GGTTGCAGAGTA---------------------------------------------------------

---

>Cow_LyzC_Trachea1

------------------------------------------------------------------------------------------ATGAAGGCT---------------------------------------------------CTCCTTATTCTGGGGCTT---CTG---CTC

---CTTTCTGTTGCT---------GTCCAGGGCAAGACATTTAAGAGGTGTGAGCTTGCCAGAACTCTGAAGAATCTTGGATTGGCTGGCTACAAGGGAGTCAGCCTGGCAAACTGGATGTGTTTGGCCAAAGGAGAAAGCAATTATAACACACAAGCTAAAAACTATAATCCTGGAAGC

AAAAGCACTGATTATGGGATATTTCAGATCAACAGCAAATGGTGGTGTAATGAT------GGCAAA---ACTCCAAAAGCAGTTAACGGCTGTGGTGTATCC------------TGCAGTGCTTTGCTGAAAGATGACATCACTCAAGCTGTAGCATGTGCAAAG---------------

---AAGATT---------GTCAGTCAG------CAAGGCATTACAGCATGGGTGGCATGGAAAAACAAGTGTCGAAACCGAGAT---CTCACAAGTTATGTTAAG------GGTTGCGGAGTG---------------------------------------------------------

---

>Cow_LyzD_Trachea2

------------------------------------------------------------------------------------------ATGAAGGCT---------------------------------------------------CTCCTTATTCTGGGGCTT---CTG---CTC

---CTTTCTGTTGCT---------GTCCAGGGCAAGACATTTAAGAGGTGTGAGCTTGCCAAAACTCTGAAGAATCTTGGATTGGCTGGCTACAAGGGAGTCAGCCTGGCAAACTGGATGTGTTTGGCCAAAGGAGAAAGCAATTATAACACACAAGCTAAAAACTATAATCCTGGAAGC

AAAAGCACTGATTATGGGATATTTCAGATCAACAGCAAATGGTGGTGTAATGAT------GGCAAA---ACCCCAAAAGCAGTTAACGGCTGTGGTGTATCC------------TGCAGTGCTTTGCTGAAAGATGACATCACTCAAGCTGTAGCATGTGCAAAG---------------

---AAGATT---------GTCAGTCAG------CAAGGCATTACAGCATGGGTGGCATGGAAAAACAAGTGTCGAAACCGAGAT---CTCACAAGTTATGTTAAG------GGTTGCGGAGTG---------------------------------------------------------

---

>Cow_LyzF

------------------------------------------------------------------------------------------ATGAAGGCT---------------------------------------------------CTCCTTATTCTGGGGCTT---CTG---CTC

---CTTTCTGTTGCT---------GTCCAGGGCAAGACATTTAAGAGGTGTGAGCTTGCCAGAACTCTGAAGAATCTTGGATTGGCTGGCTACAAGGGAGTCAGCCTGGCAGACTGGATGTGTTTGGCCAAAGGAGAAAGCAGTTATAACACACAAGCTAAAAACTTTAATCGTGGAAGC

CAAAGCACTGATTATGGGATATTTCAGATCAACAGCAAATGGTGGTGTAATGAT------GGCAAA---ACCCCAAACGCAGTTAACGGCTGTGGTGTATCC------------TGCAGTGCTTTGCTGAAAGATGACATCACTCAAGCTGTAGCATGTGCAAAG---------------

---AAGATT---------GTCAGTCAG------CAAGGCATTACAGCATGGGTGGCATGGAAAAACAAGTGTCGAAACCGAGAT---CTCACAAGTTATGTTAAG------GGTTGCGGAGTG---------------------------------------------------------

---

>CowG_Intestine

------------------------------------------------------------------------------------------ATGAAGGCT---------------------------------------------------GTCCTTATTCTGGGGCTT---CTC---CTC

---CTTTCTGTCACT---------GTCCAGGGCAAGAAATTTGAGAAGTGTGAGCTTGCCAGAACTCTGAGAAGATATGGACTGGATGGCTATAAGGGAGTCAGCCTGGCAAATTGGATGTGTTTGACCTATGGAGAAAGCCGTTATAACACACGAGTTACAAACTACAATCCTGGAAGC

AAAAGCACTGATTATGGGATATTTCAGATCAACAGCAAATGGTGGTGTAATGAT------GGCAAA---ACCCCAAAAGCAGTTAACGGCTGTGGTGTATCC------------TGCAGTGCTATGCTGAAAGATGACATCACTCAAGCTGTAGCATGTGCAAAG---------------

---ACGATT---------GTCAGTAGG------CAAGGCATTACAGCATGGGTGGCGTGGAAAAACAAGTGTCGAAACCGAGAT---GTCAGCAGTTATATTCGG------GGTTGCAAACTG---------------------------------------------------------

---

>Cow_LyzH_Stomach2

------------------------------------------------------------------------------------------ATGAAGGCT---------------------------------------------------CTCGTTATTCTGGGGTTT---CTC---TTC

---CTTTCTGTCGCT---------GTCCAAGGCAAGGTCTTTGAGAGATGTGAGCTTGCCAGAACTCTGAAGAAACTTGGACTGGACGGCTATAAGGGAGTCAGCCTGGCAAACTGGTTGTGTTTGACCAAATGGGAAAGCAGTTATAACACAAAAGCTACAAACTACAATCCTAGCAGT

GAAAGCACTGATTATGGGATATTTCAGATCAACAGCAAATGGTGGTGTAATGAT------GGCAAA---ACCCCTAATGCAGTTGACGGCTGTCATGTATCC------------TGCAGCGAATTAATGGAAAATGACATCGCTAAAGCTGTAGCGTGTGCAAAG---------------

---CATATT---------GTCAGTGAG------CAAGGCATTACAGCCTGGGTGGCATGGAAAAGTCATTGTCGAGACCATGAC---GTCAGCAGTTACGTTGAG------GGTTGCACCCTG---------------------------------------------------------

---

>Cow_LyzJ_Stomach3

------------------------------------------------------------------------------------------ATGAAGGCT---------------------------------------------------CTCATTATTCTGGGGTTT---CTC---TTC

---CTTTCTGTCGCT---------GTCCAAGGCAAGGTCTTTGAGAGATGTGAGCTTGCCAGAACTCTGAAGAAACTTGGACTGGATGGCTATAAGGGAGTCAGCCTGGCAAACTGGTTGTGTTTGACCAAATGGGAAAGCAGTTATAACACAAAAGCTACAAACTACAATCCTAGCAGT

GAAAGCACTGATTATGGGATATTTCAGATCAACAGCAAATGGTGGTGTAATGAT------GGCAAA---ACCCCCAACGCAGTTGACGGCTGTCATGTATCC------------TGCAGCGAATTAATGGAAAATGACATCGCCAAAGCGGTAGCATGTGCAAAG---------------

---CATATT---------GTCAGTGAG------CAAGGCATTACAGCATGGGTGGCATGGAAAAGTCACTGTCGAGACCATGAC---GTCAGCAGTTATGTTCAG------GGTTGCACCCTG---------------------------------------------------------

---

>Cow_LyzI_Stomach1

------------------------------------------------------------------------------------------ATGAAGGCT---------------------------------------------------CTCATTATTCTGGGGTTT---CTC---TTC

---CTTTCTGTTGCT---------GTCCAGGGCAAGGTCTTTGAGAGATGTGAGCTTGCCAGAACTCTGAAGAAACTTGGATTGGACGGCTATAAGGGAGTCAGTCTGGCAAACTGGCTGTGTTTGACCAAATGGGAAAGCAGTTATAACACAAAAGCTACAAACTACAATCCTGGCAGT

GAAAGCACTGATTATGGGATATTTCAGATCAACAGCAAATGGTGGTGTAATGAT------GGCAAA---ACCCCCAACGCAGTTGACGGCTGTCATGTATCC------------TGCAGCGAATTAATGGAAAATGACATCGCGAAAGCTGTAGCGTGTGCCAAG---------------

---CAGATT---------GTCAGTGAG------CAAGGCATTACAGCATGGGTGGCATGGAAAAGTCACTGTCGAGACCATGAC---GTCAGCAGTTATGTTGAG------GGTTGCACGCTG---------------------------------------------------------

---

>Cow_LyzK

------------------------------------------------------------------------------------------ATGAAGGCT---------------------------------------------------CTCCTTATTCTGGGGCTT---CTC---CTC

---CTTTCTGTCCCT---------GTCCAGGGCAAGGTCTTTGAGAGATGTGAGCTTGCCAAAACTCTGAAAAGACTTGGAATGGATGGCTTTAGGGGAGTCAGTCTGGCAAATTGGTTGTGTTTGACCAAATGGGAAAGCAGTTATAACACAAAAGCTACAAACTACAATCCTAGCAAT

GAAAGCACTGATTATGGGATATATCAGATCAACAGCAAATGGTGGTGT---------------AAA---ACCCCCAAAGCAGTTGACGGCTGTCCTGTGTCC------------CACAGC---------------------ACTAAAGCTGTAGCATGTGCAAAG---------------

---AAGATT---------GTCAGCGAG------CAAGGCATTACAGCATGGGTGGCATGGAAAAGTCATTGTCGAGACCATGAC---GTCAGCAGTTATGTTGAG------GGTTGCACCCTG---------------------------------------------------------

---

>Armadillo_LyzB

------------------------------------------------------------------------------------------GTGAAGACT---------------------------------------------------CTCCTTATTCTGGGGCTT---CTC---TTC

---CTTTCTGCCACT---------ATCCAGGGCAAGAAGTTTGAAAGATGTGAA---GCTAGAACTCTGAAAAGACTTGGACTGGGCGGTGTCAAGGGAATCAGCCTGGCAAACTGGATGTGTTTGGCCAGATGGGAGAGTGATTATGATACACAAGCTATAAACTTCAATCCTGGAGAC

AAAAGTACCGATTATGGGATTTTTCAGATCAATAGCCGCTACTGGTGTAATGAT------GGCAAA---ACTCCAGGTGCAGTTAACGGCTGTGGTATATCC------------TGCAATGTTTTGTTACAAGATGACATCACACAAGCTGTGAAGTGTGCAGAG---------------

---AGGGTT---------GTCAGCGATCCA---CAAGGCATCAGAGCATGGGTGGCA---AAAAAACATTGTCAGAACCAAGAT---CTCACTCAGTACATTCAA------GGTTGTGGAGTT---------------------------------------------------------

---

>Opossum_LyzA

------------------------------------------------------------------------------------------ATGAAGGTC---------------------------------------------------CTGATTCTTTTGGGTCTT---GTC---TTC

---CTCCCCATGCTA---------GCTCATGGCAAGGTCTACGAAAGATGTGAATTGGCAAGAGTCCTGAAGCAAAATGGGATGGATGGCTTCGGAGGTAACAGCCTGGCCGACTGGGTGTGCTTGGCAAAATGGGAAAGTGATTATAACACAAAAGCCACAAATTACAACCCTGGAGAT

CAAAGCACCGATTATGGGATATTTCAGATCAACAGTCACTATTGGTGCAATGAT------GGCAAG---ACTCCTGACGCTAAGAATGTGTGCGGCATCTCA------------TGTAGAGATCTTTTAACAGATAATATCAGCAAAGCTATAACCTGTGCAAAG---------------

---AGGATC---------GTCCAAGATTTC---CAGGGCATTCGGGCATGGGTGGCATGGAGAAACCATTGCGAAGGAAGGGAT---GTTTCCTCTTATATCCGA------GGTTGCGGTCTT---------------------------------------------------------

---

>Opossum_LyzC

------------------------------------------------------------------------------------------ATGAAGGTC---------------------------------------------------CTGATTCTTTTGGGTCTT---GTC---TTC

---CTCCCCATGCTA---------GCTCATGGCAAGGTCTACGAAAGATGTGAATTGGCAAGAGTCCTGAAGCGAAATGGGTTGCATGGCTTCAGAAGTAACAGCGTGGCCGACTGGGTGTGCTTGGCAAAATGGGAAAGTGATTATAACACAAAAGCCACAAATTACAACCCTGGAGAT

CAAAGCACCGATTATGGGATATTTCAGATCAACAGTCACTATTGGTGCAATGAT------GGCAAG---ACTCCTGATGCAAAGAATGTGTGCGGCATCTCA------------TGTAGAGATCTTTTAACAGATAATATCAGCAAAGCTATAACCTGTGCAAAG---------------

---AGGGTT---------GTCCGTGATCCC---AGTGGCATTCGGGCATGGGTGGCATGGAGAAACCATTGCGAAGGAAGGGAT---GTTTCCTCTTATATCCGA------GGTTGCAGTCTT---------------------------------------------------------

---

>Opossum_LyzB

------------------------------------------------------------------------------------------ATGAAGGTC---------------------------------------------------CTGATTCTTTTGGGTCTT---GTC---TTC

---CTCCCCATGCTA---------GCTCATGGCAAGGTCTACAAAAGATGTGAATTGGCAAGAGTCCTGAAGCAAAATGGGATGGATGGCTACAGAGGTAACAGCGTGGACAACTGGGTGTGCTTGGCAAAATGGGAAAGTGATTTTGACACAGAAGCCACAAATTACAACCCTGGAGAT

CAAAGCACCAATTATGGAATATTTCAGATCAACAGTCACTATTGGTGCAATGAT------GGCAAG---ACTCCTGACGCTAAGAATGTATGCGGCATCTCA------------TGTAGAGATCTTTTAACAGATAATATCACCCAAGCTGTAAACTGTGCAAAG---------------

---AGGGTT---------GTCCATGATCCC---AGTGGCATTCTGGCATGGTATGCATGGAGAAACCATTGTCAAGGAAGGGAT---GTTTCCTCTTATATCCAA------GATTGCCGTCTG---------------------------------------------------------

---

>Opossum_LyzD

------------------------------------------------------------------------------------------ATGAAGGTC---------------------------------------------------CTGATTCTTTTGGGTCTT---GTC---TTC

---CTCCCCATGCTA---------GCTCATGGCAAGGTCTACGAAAGATGTGAATTGGCAAGAATCCTGAAGCAAAATGGGATGGATGGCTACAGAGGTATCAGCCTGGCCAACTGGGTGTGCTTGGCAAAATGGGAAAGCAATTATAACACACGAGTCACAAACTACAACTCTAGAGAT

CGAAGCACAGATTATGGAATATTTCAGATCAACAGTCGCTACTGGTGCAATGAT------GGGAAG---ACCCCTAGAGCTGTGAATGCGTGTGGCATCTCA------------TGTAGAGATCTTTTAACAGATAATATCAGCAAAGCTATAACCTGTGCAAAG---------------

---AGGGTT---------GTCCGTGATCCC---AGTGGCATTCGGGCATGGGTGGCATGGAGAAACCGTTGCCAAGGAAAGAAT---CTTTCCTCTTATATCCAA------GGTTGCCGTCTT---------------------------------------------------------

---

>Platypus_Lyz

------------------------------------------------------------------------------------------ATGAAGCCT---------------------------------------------------CTCTTTGTGCTGGGACTC---CTC---GTC

---CTTCCCTTCCTT---------GCCCATGGCAAGGTTTTCGAAAGGTGCGAACTCGCGAGGCTTCTGAAGAGAATGGGAATGGATGGCTACAGGGGAGTGTCTCTGCCAAACTGGGTCTGTTTGGCCAACTGGGAGAGCAGCTTCAACACGAATGCAAAAAACTACAACCCTGGCGAC

GGAAGCACCGATTATGGGATTTTCCAGATCAACAGCCGCTACTGGTGCAACGAT------GGCAAG---ACCCCGAGAGCCGTGAACGCCTGCGGCATCTCT------------TGTAACGAATTGTTGAAAGATGATATTACCCAAGCAGTCCGATGTGCCAAG---------------

---AGAGTG---------GTGGAGGATCCC---CAAGGCATCAGGGCATGGGTTGCATGGAGAAACAGATGCCAAGGACAGGAT---GTGTCCATCTGGACCCGT------GGCTGTGGAGTG---------------------------------------------------------

---

>Guinea_pig_LyzH

------------------------------------------------------------------------------------------ATGAAGGCT---------------------------------------------------CTGCTTCTTCTGGGCCTT---GTC---CTC

---TTGTGCGGGACC---------GTCAAGGGAAAGATCTACAAAAGATGTGAGCTGGCCAAAGCTCTGAAAGAAAAAGGAATGGCTGGCTACCATGGAATCAGCCTGGCAAACTGGATGTGCTTGGCCAAGGCGGAGAGTAGTTACAACACACGAGTTACAAACTACAATCCTGGAGAC

AAAAGCACTGACTATGGGATATTCCAGATTAACAGCCACTATTGGTGCAACGAT------GGCAAA---ACCCCCAATGCTGTTAATGGTTGCCACGTATCC------------TGCAAAGATTTGATGCATGATGACATCTCTAAGTCTGTGGCTTGTGCGAAG---------------

---AGGGTT---------GTGAAGGACCCA---CAAGGCATTAGAGCATGGATGGGATGGAGAAAAGAGTGTCAAAACAAAGAT---CTCTCGTCCTACGTTAAA------GGCTGTGGAGTG---------------------------------------------------------

---

>Guinea_pig_LyzG

---------------------------------------------------------------------------------------------------------------------------------------------------------------------GTC---CTT---CTC

---TTGTCTGGGACC---------ATCTATGGAAAGGTCTATGAAAGACGTGATTTGGCCAAAACCCTGAAAGCAGCAGGGATGGATGGTTATCAAGGAATTAACTTGGCAAACTGGATGTGCTTGGCCAAGGCGGAGAGTAGTTACAACACACGAGTTACAAACTACAATCCTGGAGAC

AAAAGCACTGACTATGGGATATTCCAGATTAACAGCCACTATTGGTGCAACGAT------GGCAAA---ACCCCCAATGCTGTTAATGGTTGCCACGTATCC------------TGCAAAGCTTTGATGCAAGATGACATCTCTCAGTCTGTAGTTTGTGCAAAA---------------

---AATATT---------GTGCTGGATCCA---CAAGCTAAATATGCAATGATGGGATGGAGAAAGCATTGTCAAAACAAAGAT---GTCAAGCCATACACTCAT------GACTGTGGAGTA---------------------------------------------------------

---

>Sloth_LyzB

------------------------------------------------------------------------------------------ATGAAAGCT---------------------------------------------------CTCCTCGTTCTGGGGCTT---CTC---CTC

---CTTTCTGCCTCT---------GTCCAGGGCAAGACGTTTGAAAGGTGTGCATTG---CAAACTCTGAAAAACCTTGGATTGGAT------TACGGCATCAGC---GCAAACTGGATGTGTTTGACCAAATGGGAGAGTGGTTATAACACAAAAGCTACAAACTACAATTCCGGACCC

CAAAGCACCGATTATGGGATTTTTCAGATCAATAGCCGCTACTGGTGTAATGAT------GGCAAA---ACCCCAAATGCAGTTAACTCCTGTGGTATATCC------------TGCAGCGTTTTGCTGCAAGATGACATCACACAAGCTGTGAAGTGTGCAAAG---------------

---AAGATT---------GTCAGTGAT------CAAGGAATTACAGCATGGGTGGCATGGAAAAGCCATTGTCAGGGCAAAGAT---GTCTCTCAGTATGTTCAA------GGTTGTGGAGTT---------------------------------------------------------

---

>Armadillo_LyzC

------------------------------------------------------------------------------------------ATGAAGGCT---------------------------------------------------CTCCTTATTCTGGGACTT---CTC---CTC

---CTTTCTGCCAGT---------GTGCAGGGCAATCAGATGGAAAGGTGTAAATTGGCCAAATCCTTAAAACACTACGGAATGGACAAATTCAAAGGAATCAGCCTAGCACAATGGATGTGTGTGATCAAATGGGAGAGCAATTATAGGACTGATGCTATAAACCACAATACA---GAC

AAGAGCACCGATTATGGGCTTTTTCAGATCAATAGTCGCTACTGGTGTCATGAT------GGCAAA---ACCCCAAAAGCATTTAACGGCTGTGGTATATCC------------TGCAAAGTTTTGCTAAAAGATGACATCACAGAAGCTGTGAAGTGTGCAAAG---------------

---AAGATT---------GTCAGTACA------CAAGGCATTAGAGCATGGGTGGCGTGGAAAAAACATTGTCAGAACAAAGAC---GTCTCGGAGTATGTTAAA------GGCTGTGGAGTT---------------------------------------------------------

---

>Sloth_LyzA

------------------------------------------------------------------------------------------ATGAAGGCT---------------------------------------------------GTCCTTGTTCTGGGGCTT---CTC---CTC

---CTTCCT---------------GTCCAGGGCAAGGTT---GAAAGGTGTAAATTGGCCAGAACTCTGAAAAGACTTAGAATGGCTGGATTCAGGGGAATCAGACTGCCAAAGTGGGTGTGTTTGGTCCAATGGGAGAGTGATTATAACACAAAAGCTACAAACTTCAATCCTGGAGAC

AAAAGCACCGATTACGGAATTTTTCAGATCAATAGCCACTACTGGTGTAATGAT------GGCAAA---ACCCCAGGAGCAGTTAACACCTGCGGTATACCC------------TGCAGCGTTTTGCTGCAAGATGACCTCACACCAGCTGTGAAGTGTGCAAAG---------------

---AAGATT---------GTCAGTTCA------CAAGGCATTAGCGCATGGGTGGCATGGAAAACCCACTGTCAGAACAAAGAT---GTCTCTCAGTATGTTCGA------GGTTGTGGAGTT---------------------------------------------------------

---

>Wallaby_LyzC

------------------------------------------------------------------------------------------ATGAAGGTT---------------------------------------------------CTCTTTCTCCTGGGGTTC---ATC---TTC

---TTCCCCATGACA---------GTACATGGTGTGACGATGAACAGATGTGCATTTGCCAGAAGAATAAAGGAGTTGGGCTTGGATGGGTACCGGGGTGTCAGCCTGGCTAACTGGGTGTGCTTGGCCCAGTGGGAGAGCAATTTTAACACCAAAGCTAAAAACTACAACCCTGGAGAT

CAAAGCACTGATTATGGGATATTTCAGATCAACAGTCACTACTGGTGTAATGAT------GGCAAG---ACTCCAAGGGCAACAAATGGGTGCAAAGTCCGG------------TGTTCAGAACTTGAGGAAGACAACCTTGTTAAAGCAGTACAGTGTGCAAAG---------------

---CAGATT---------GTGCGTCAA------CAAGGCATTGGTGCATGGGTGGCCTGGCGAAACCACTGTCAAGGACACGAT---GTTTCCAAGTATCTTGAT------GGTTGCCATCTG---------------------------------------------------------

---

>Wallaby_LyzD

------------------------------------------------------------------------------------------ATGAAGTTT---------------------------------------------------CTCTTTCTCCTGGGGTTC---CTC---TTC

---TTCCCCATGACA---------GTACATGGTGTGAGGATGAGCAGATGTGCATTTGCCAGAAGAATAAAGGAGTTGGGCTTGAGTGGGTACCATGGTGTCAGCCTGGCTAACTGGGTGTGCTTGGCACAGTGGGAGAGCGATTTTAACACCGCAGCTACAAACTACAATTCTTGGGAT

CGAAGCACTGATTATGGGATATTTCAGATCAACAGTCACTACTGGTGTGATGAT------GACAAG---ACCCCACATGCAACAAATGGGTGTGGAGTCAGG------------TGTTCACAACTTGAGGAAACCAACCTTGTTAAAGCAGTAGAGTGTGCAAAG---------------

---CAGATT---------GTGCGTCAA------CAAGGCATTAGTGCATGGGTGGGCTGGAAAAACCACTGTGAAGGACACAAT---CTTTCAGGGTATCTTGAA------GGTTGCAGTCTG---------------------------------------------------------

---

>Mouse_LyzC

------------------------------------------------------------------------------------------ATGAAGGCT------------------------------------------CCCCTAACTCTCCTTACTCTAGGGCTC---CTC---CTG

---CTCTCCATCACC---------ATCCAGGGCAAGGTCTACGACCGATGTTCTTTGGCCAGAACTCTACAAAGTCTTGGTCTAGCTGGTTTCCAAGGAATCACGCTGGCAAACTGGGTCTGTTTGGCCAAATGGGAGAGTAATTTTAACACAAACACTACAAGATTCAACCCTGAAGAC

CAAAGCACCAGCTATGGGATCTTTCAGATCAACAGCAGATTCTGGTGTAATGAC------GGCAAA---ACCCCAGGATCAAGGAACTTTTGTCGGATATCC------------TGCAAAGCTTTGCTGAAGAGCAACATCTGGTCTGCCGTAGTTTGTGCAAAG---------------

---AGGATC---------GTGAAGGATCCA---CAAGGAATTTATTCATGGGCGGGATGGATAAAACACTGTAAAAACAAAAAC---CTCAAAGAGTACATTCGG------GGCTGCCATCTG---------------------------------------------------------

---

>Rat_LyzC

------------------------------------------------------------------------------------------ATGAAGGCT------------------------------------------CTCCCAACTCTCCTCACTCTGGGGCTC---CTC---CTG

---CTTTCCATCACA---------GTCCAGGGCAAGGTCCTCAACCGATGTTTGCTAGCCAGAACTCTACAAAGATTTGGACTAGGTGGCTTCAAAGGAATCAGCCTGGCGAATTGGATCTGTTTGGCTAAGTCGGTGAGTGGTTATGACACAAAAGCTATAAAATACAACCATGAAGAC

CGAAGCACCAACTATGGGATATTTCAGATTAGTAGCCGATACTGGTGTAATGAC------AGCAAA---ACCCCAGGATCAAAGAACTTTTGTCGGGTGTCC------------TGCAAAGCTTTGCTGAAGAATAACATCAAGGCTAGTGTAACTTGTGCGAAG---------------

---AGGATC---------GTGAAGGATCCA---CGAGGAATTACTACATGGTATGCATGGAGGAAAAACTGTGAGCACAAAAAC---CTCTTTCAGTACGTTCAG------GGCTGCAGTCTA---------------------------------------------------------

---

>Chicken_Lyz

------------------------------------------------------------------------------------------ATGAGGTCT---------------------------------------------------TTGCTAATCTTGGTGCTT---TGC---TTC

---CTGCCCCTGGCT---------GCTCTGGGGAAAGTCTTTGGACGATGTGAGCTGGCAGCGGCTATGAAGCGTCACGGACTTGATAACTATCGGGGATACAGCCTGGGAAACTGGGTGTGTGCTGCAAAATTCGAGAGTAACTTCAACACCCAGGCTACAAACCGTAACACC---GAT

GGGAGTACCGACTACGGAATCCTACAGATCAACAGCCGCTGGTGGTGCAACGAT------GGCAGG---ACCCCAGGCTCCAGGAACCTGTGCAACATCCCG------------TGCTCAGCCCTGCTGAGCTCAGACATAACAGCGAGCGTGAACTGCGCGAAG---------------

---AAGATC---------GTCAGCGATGGA---AACGGCATGAACGCGTGGGTCGCCTGGCGCAACCGCTGCAAGGGCACCGAC---GTCCAGGCGTGGATCAGA------GGCTGCCGGCTG---------------------------------------------------------

---

>Turkey_Lyz

------------------------------------------------------------------------------------------ATGAGGTCT---------------------------------------------------TTGCTAATCTTGGTGCTT---TGC---TTC

---CTGCCCCTGGCT---------GCTCTGGGGAAAGTCTATGGACGATGTGAGCTGGCAGCAGCTATGAAGCGTCTGGGACTTGATAACTATCGGGGATACAGCCTGGGAAACTGGGTGTGTGCCGCAAAATTCGAGAGTAACTTCAACACACATGCTACAAACCGTAACACC---GAT

GGGAGTACCGACTACGGAATCCTACAGATCAACAGCCGCTGGTGGTGCAATGAT------GGCAGG---ACCCCAGGCTCCAAGAACCTGTGCAATATCCCA------------TGTTCAGCACTGTTGAGCTCAGACATAACAGCGAGCGTGAACTGCGCGAAG---------------

---AAGATC---------GCCAGCGGTGGA---AACGGCATGAACGCGTGGGTCGCCTGGCGCAACCGCTGCAAGGGCACCGAC---GTCCACGCGTGGATCAGA------GGCTGCCGGCTG---------------------------------------------------------

---

>Duck_Lyz

------------------------------------------------------------------------------------------ATGAAGGCT---------------------------------------------------TTGCTGACCCTCGTCTTT---TGC---CTC

---CTACCCCTGGCT---------GCTCAGGGGAAAGTCTACAGCAGGTGTGAGCTGGCAGCAGCTATGAAGCGACTCGGACTGGACAACTACCGGGGATACAGCCTGGGAAACTGGGTGTGTGCAGCAAATTACGAGAGCGGCTTTAACACGCAGGCTACAAACCGTAACACT---GAT

GGAAGCACCGACTACGGAATTTTACAGATCAACAGCCGCTGGTGGTGCGATGAT------GGCAAG---ACCCCAAGGTCCAAGAACGCGTGCGGTATCCCA------------TGCTCAGTACTACTGAGATCAGACATAACAGAAGCTGTGAGGTGTGCAAAG---------------

---AGGATT---------GTCAGCGATGGA---AATGGCATGAACGCATGGGTCGCGTGGCGCAACCGGTGCAGAGGCACAGAT---GTCTCCAAGTGGATCCGA------GGCTGCAGGCTA---------------------------------------------------------

---

>Fugu_Lyz

---------------------------------------------------------------------------------ATGATTTGTCTAACTTTG------------------------------------------------------------ATTGTGTTT---CTG---CTT

---CTTTTGGCTTTG---------GCCAACGCTAAAGTCTTCCAGCGCTGTGAATGGGCTCGAGTGCTGAAGGCACGCGGGATGGACGGCTATCGTGGAATTAGCCTGGCTGACTGGGTCTGTCTGTCCAAGTGGGAGTCACAATACAACACCAATGCGATCAACCACAACACC---GAT

GGTTCCACTGACTATGGCATCTTTCAGATCAATAGCCGCTGGTGGTGCAACGAT------GATCGC---ATCCCG---ACGCGCAATGCATGCAACATCAAG------------TGTAGTGCACTTCAGACTGATGATGTCACCGTGGCGATCAATTGTGCAAAA---------------

---CGTGTT---------GTCAGTGACCCT---CAAGGCATCCGGGCCTGGGTTGCCTGGAATCGACACTGCCAAAACCGTGAT---CTGAGTGCCTACATTGCA------GGATGTGGTCTT---------------------------------------------------------

---

>Tetraodon_Lyz

ATGAGGCACAAAGTTCACAGCAACGGAGGAGCAGCGTTAGCGGCGCCAGGACGCCTAGCGTCCGTCAGAAGGCTAGCAGCAGCAGCAGCGATGAGGACT------------------------------------------------------------CTGGTGTTT---CTG---CTC

---CTGTTGGCTGTG---------GCCAGCGCTAAGGTCTACCAGCGCTGTGAGCTGGCCCGAGTGCTGAAGAGCCAGGGGATGGATGGCTACCGAGGGATCAGCCTAGCCAACTGGGTCTGTCTGTCCAAGTGGGAGTCCGAGTACAATACCAACGCGATCAACCACAACACC---GAT

GGTTCCACCGACTACGGCATCTTTCAGATCAACAGCCGCTGGTGGTGCAATAAT------GATGTG---ACCCCG---ACAAGCAACGGGTGCAACATCAAG------------TGCAGGGCGCTCCTGACTGATGACATCAGCGTGGCCATCGCCTGTGCCAAG---------------

---CGTGTG---------GTCAGAGATCCT---CAAGGCATCAGGGCCTGGGTTGCGTGGAGAAACCGGTGCCAAGGACGCGAT---CTGAGCGGCTACGTTGCA------GGATGTGGCCTT---------------------------------------------------------

---

>Stickleback_Lyz

------------------------------------------------------------------------------------------ATGAGGGCT------------------------------------------------------------GTGGTGTTT---CTG---CTG

---TTGGTGGCCGCG---------GCCGGTGCGAAGGTCTACGAGCGATGCGAATGGGCCCGCGTGCTGAAGGCTAATGGGATGGACGGATACGGAGGCTACGGCCTGGCCGACTGGGTCTGTCTGAGCTACTCGGAGTCCGGTTACAGCACCACCGCCACAAACTTCAACACC---GAC

GGATCAACTGATTACGGCATCTTCCAGATCAACAGCCGCTGGTGGTGTGAAGAC------GGGCGG---ACCCCCCGCTCAAAGAACGCATGTTCCATCTCG------------TGCAGCGCACTTGTTTCTGATGATGTCAGAGTGGCAATCAATTGTGCCAAA---------------

---CGCGTC---------GTCAGGGATCCC---AACGGCATGAGAGCCTGGGTGGGCTGGCGCAATCACTGCGAGCACCGTGAC---GTGAGCTCCTATCTGGCA------GGATGTCGTCTT---------------------------------------------------------

---

>Medaka_Lyz

------------------------------------------------------------------------------------------ATGAAGAGC------------------------------------------------------------CTTGTGTTT---TTG---CTG

---TTGGTGGCCGGT---------GCCAGTGCCAAAGTGTTTGAGCGCTGTCAGTGGGCCCGCCTGCTGAAGGCTCAGGGAATGGATGGGTATCGCGGTGTGAGCCTGGCCAACTGGGTCTGCCTGACACAACATGAGTCTAGATTCAACACCAATGCCATCAACCACAACAGG---GAC

GGGTCAACGGATTATGGCATCTTCCAGATCAACAGTTATTGGTGGTGTGACGAT------GGAAAA---ACCGGA---AGAGTCAACGGGTGCAAAATTCCA------------TGCAGCGCGCTTCTGTCTGACTCGGTGGGCACAGCCATCGCCTGTGCCAAA---------------

---CGCATC---------GTTCAGGATCCA---AGTGGCATAGCTGCCTGGGTGGCTTGGCGTGATCACTGCAGAGGGAGAGAC---GTGAGTTCCTACATCCAG------GGATGTGGATTC---------------------------------------------------------

---

>Rat_LyzB

------------------------------------------------------------------------------------------ATGAAGGCT---------------------------------------------------CTCCTAGTTCTGGGGTTC---CTC---CTG

---CTCTCTGCCTCT---------GTCCAGGCCAAGGTCTTTAAACACTGTGAGTTGGCCAGAATTCTGAGAAGTTCTGCACTAGCAGGCTATCGTGGAGTCAGCCTGGAAAACTGGATGTGTATGGCTCAGCATGAGAGTAATTTTGATACAGAAGCTATAAACTACAACTCTACTGAC

CAAAGCACCGACTATGGGATATTTCAGATCAATAGCCGATACTGGTGTAATGAT------GGCAAA---ACCCCAAGAGCAGTGAACGCCTGTGGGATACCC------------TGCAGCGGTAAGACAAGGCGGGTTACAACCCAGGCCCACAGTCATGTCATG---------------

---ATTGCA---------GTATTCTGTCCC---CAGGGTTTGAGTGTG---GTGGCATGGCAAAGACACTGTCAAAACCGAGAT---CTATCCGGGTATATTCGG------AACTGCGGAGTC---------------------------------------------------------

---

>Xenopus_LyzA

------------------------------------------------------------------------------------------ATGAACTCT------------------------------------------------GCAGTGCTTTTCTGGGGGGGG---ATT---TTT

---ATTTTTACAGTC---------ACAGATGGAAAAGTATTCGAAAGATGTGAACTTGCTGGAATTATGAAGAAGATGGGACTTGATGGGTATCGAGGATACAGCCTGCCTAACTGGGTGTGTACAGCTTTTTTTGAGAGCTCATTTTATACAGACCGTACAAATTTCAACAGAGGAGAT

AACAGCACTGATTATGGCATTCTCCAAATAAACAGTCGTTGGTGGTGTAATGAC------AACAAG---ACTCCAGGAAGTCACAATGCTTGCAACATTAAT------------TGCAGAGCTCTCTTGAGTGATGACATCACCCAGTCTGTGATATGTGCAAAA---------------

---CGCGTT---------GTAAGAGACCCA---CAGGGCATGGAAGCATGGGTTGGCTGGAGAAATCATTGCAAAGGAAGAGAC---CTTTCTCAGTGGATTAAA------GACTGCAAACTTGATATTCTTCCG---------------------------------------------

---

>Dog_Lysc1

------------------------------------------------------------------------------------------ATGAGGTCT---------------------------------------------------ATTCTGGTCATCACCATC---CTCAGCTAC

---TTCTTTGTAGCA---------GATGAGGCCAAAATCTTCTCCAAGTGTGAGCTGGCCCGCAAGCTGAAGAGCATGGGAATGGATGGCTTCCATGGCTACAGCCTGGCAAACTGGGTCTGCATGGCTGAGTATGAGAGTAACTTCAACACCCAGGCCTTTAATGGGAGAAATTCCAAT

GGCAGTAGTGACTATGGAATCTTCCAGCTGAACAGCAAGTGGTGGTGCAAAAGC------AACTCT---CACTCCTCGGCA---AATGCCTGCAACATAATG------------TGCAGCAAGTTCCTGGATGACAACATCGATGATGATATCGCCTGTGCCAAG---------------

---AGGGTT---------GTGAAAGATCCT---AATGGGATGTCTGCCTGGGTGGCCTGGGTTAAACACTGCAAAGGCAAAGAT---TTGTCCAAATACCTGGCC------AGCTGTAACCTG---------------------------------------------------------

---

>Horse_Lysc1

------------------------------------------------------------------------------------------ATGAGGTCC---------------------------------------------------ACTCTCATCATCTCCCTC---CTCAGCTGC

---TTCTTTGCAGTT---------TACGAGGCCAAAGTCTTCTCCAAGTGTGAGCTGGCCCACAAGCTAAAGGCCCAGGAAATGGATGGTTTCGGTGGCTACAGCCTGGCAAACTGGGTCTGCATGGCTGAGTATGAGAGTAACTTCAACACCCGGGCCTTTAATGGAAAAAATGCCAAT

GGCAGTAGTGACTATGGGCTCTTCCAGCTGAACAACAAGTGGTGGTGCAAAGAT------AACAAG---CGTTCTTCATCA---AATGCCTGCAACATAATG------------TGCAGCAAACTTTTGGATGAGAACATCGATGATGACATCAGCTGTGCCAAG---------------

---AGGGTT---------GTGAGAGATCCT---AAAGGGATGTCTGCCTGGAAGGCCTGGGTAAAACACTGCAAGGACAAGGAT---TTGTCCGAATACCTGGCT------AGCTGTAACCTG---------------------------------------------------------

---

>Shrew_Lysc1A

------------------------------------------------------------------------------------------ATGAGGTCC---------------------------------------------------ATCCTGGTCATCTCTCTC---CTCAGTTGC

---TTCTTTGCGGCC---------TACGAGGCCAAAATCTTCCCCAGATGTGAGCTAGCCAAGAAGCTGAAGGCCCAGGGGCTGGATGGCTACCAGGGCTACAGCCTGGCAAACTGGGTCTGCTTGGCTGATCATGAGAGTAGCTACAATACTAAAGCCTTCAATGGGAAAAATTCTGAT

GGCAGCAGTGACTATGGGATCTTCCAGCTGAACAACAGGTGGTGGTGCACAGAC------AGCAAG---TATCCCTCTGCG---CACGGCTGCAACATGGCA------------TGCAGTAAATTTTTGGATGACAACATTGATGATGACATCGCCTGTGCCAAG---------------

---AGAGTT---------GTGAAAGATCCC---AATGGGATAAGTGCCTGGGTTGGCTGGGTTAAACACTGCAAAGGAAAAGAT---TTGTCTAAGTACTTGGCT------GGCTGCAATCTG---------------------------------------------------------

---

>Shrew_Lysc1B

------------------------------------------------------------------------------------------ATGAGGTCC---------------------------------------------------ATGCTGATTCTCTTTCTC---TTCAGCTAC

---TTCTTTGCAAGT---------TATGATGCCAAAATCTTCTCTGAATGTGAGCTAGCCAAAAAGCTGAAGGCCCATGGAATGGATAACTTCCATGGTTACAGCCTGGCAAACTGGGTCTGCATGGCTAAGCACGAAAGTGATTTAAATACCAAGGCCCTCAATGACAAAAATTCTGAT

GGCACCATTGACTATGGGATATTCCAGGTGAACAACAAATGGTGGTGCAAGGAC------AACAAA---CGTCAGTCAAAA---AATGCCTGCAACATAGCA------------TGCAGCAAACTCTTGGATGACAACATTGATGATGACATCACTTGTATCAAA---------------

---AGAATA---------GTGAAAGATCCT---AACAAGATGAATGCCTGGTATGCCTGGGTCAAACACTGCAAAGGCAAGGAT---TTATCCAAGTACTTGGCT------GGCTGTAAGCTA---------------------------------------------------------

---

>Lizard_LyzA

---------------------------------------------------------------------------------------------ATGAAA---------------------------------------------------GTGTTGGCCTTCACCCTC---TTTTGCCTC

---TTCATTGCTGTC---------AATGAAGCCAAAGTATATGAGAGATGCGAATTGGCTAGGAAACTGAAAAACTCTCGGTTGGATGTTTCATCTGGCTACAGCATTGCCGACTGGGTCTGCTTGGCTTACTATGAGAGCAGATTCAACAGCAGGGCCGTGGGGCCACCCAACCGGAAT

GGAAGTCGTGACTATGGGATTTTTCAGATAAACAGCCGCTGGTGGTGCTCCAAT------GGAAAA---GGCACAACTGCC---AATGGCTGCAGAAGCTCC------------TGCAGTGCTTTCACAGATGATGACATCACAAATGATATTGCATGCGCTAAG---------------

---AGAATT---------GTTAAAGATCCC---AACGGGATAAGAGCCTGGGTCGCTTGGGTGAAATACTGCCAAGGAAAAAAC---CTCACCCGGTGGACACAA------GGCTGCAGACTC---------------------------------------------------------

---

>Lizard_LyzD

---------------------------------------------------------------------------------------------ATGAAA---------------------------------------------------GTGTTGGCCTTCGTCCTC---CTCTGCCTC

---TTCATTGCTGTC---------AGTGAAGCCAAAGTGTATGAGAAATGCGAACTGGCAAAGATCCTGAAAATCTCTAAGATGGATGTCTCATCTGGCTACAGCCTTGACAATTGGGTCTGCTTGGCTTACCATGAGAGCAGATTCGACAGTAAGGCCGTGGGGCCACCCAACTGGGAT

GGAAGTCGTGACTATGGGATTTTTCAGATAAACAGCCGCTGGTGGTGCTCCAAC------GGAGAG---GGCACAACTGCC---AATGGCTGCAAAACCTCC------------TGCAGTGCTTTCACAACTGATGACATCACAGATGATATTACCTGTGCTAAG---------------

---AGGATT---------GTTCGTGATCCC---AACGGAATACGTGCCTGGGTTGCTTGGGTGAACCACTGCGAAGGAAGAGAC---CTCTCCAGCTGGACTAAA------GACTGCAGTCTC---------------------------------------------------------

---

>Xenopus_LyzB

------------------------------------------------------------------------------------------ATGAAGAGT---------------------------------------------------CTCCTCTGCGCCATTCTC---TTTGCC---

---TTCCTCGCGGGC---------ACCGATGCCAAGGTCTTCGCCAAATGTGACCTGGCCAAGGTGCTGAAAGCCGGGGGCCTGGACGGTTACTATGGCTACAGCCTGGCTAACTGGATGTGCCTGTCGTACTACGAGAGCCGCTACACCACCAACGCCATGTACGACAACGGCTGG---

---AGCCGGGATTATGGGGTCTTCCAGATCAACAGCTACTGGTGGTGCAACGAC------GGCAAG---ACCTCCGGAGCGGTGGCAGCCTGTGGCATCAGC------------TGTAGCAATTTAATGAATGATGACATCACAGATGACATCACATGTGCCAAG---------------

---AGAGTC---------GTGCGAGACCCC---AATGGCATGGGGGCCTGGGTTGCATGGAATAATTACTGCAAGAACAAAGAC---GTCAGCAGTTTTGTAAGT------GGCTGTGTTCTG---------------------------------------------------------

---

>Lizard_LyzL

------------------------------------------------------------------------------------------ATGGCTCCT------------------------------------------------TTGCTCCAGACCCTCCTCCTC---CTCGCCTGC

---TTTGTGGAAGCA---------ACCCAAGCCAAAATTTTTGATCGCTGTCAGCTGGCCCATGTGCTGAAGGACAACGGGTTGGATGCATTCGAGGGGATTAGCCTGGCTGACTGGATCTGTATGGCGTTTTTTGAGTCTGGCTTTGACACTGAGGCCATTGATTGGCACAAT---GAT

GGCACTAAAGACTACGGTATCTTCCACATCAACAGCGGCTGGTGGTGCAAAGAT------GAATCTCTTGACAGTTCATCAGAGAACCTTTGCTCCATGAAT------------TGTAAAGATTTATCAGATGATGACATTACAGATGACATCAACTGTGCCAAG---------------

---AGGATC---------GTGCAAGATCCC---CAAAGCATGGGGGCTTGGAATCAGTGGAAAATGCATTGTAAAGACCAAGAC---CTGTTCCGCTGGGTGAAG------GGTTGTCAGCTG---------------------------------------------------------

---

>Duck_Calzm

---------------------------------------------------------------------------------------ATGACAAAGTCA---------------------------------------------------ATTCTCTTCTTTGGCTTTCTTCTTGTCTTC

---CTTGGCCTGACTCTGCCAGGCACCCAGGGAAGAATTATTCCCCGATGTGAGCTGGTGAAGATCCTCCGCAACCATGGCTTTAAGGGCTTCGTGGGCAAAACTGTAGCTGACTGGGTCTGCCTGGTGCAACATCAGAGCAATTACAGCACCCACGCT------TTTCGTGATGAGGGT

AAGAGCCGGGACTACGGAATCTTTCAGATCAACAGCAAGTACTGGTGTGATGAT------GGCAAA---ACCCACGGAACTTCAAATACCTGTCGCATCAAC------------TGCTCGAAATTTCTAGATGATAACATTGAAGATGATATTCGGTGTGCCAAG---------------

---ATAATT---------GCTAAAGAGGCT---AATGGCCTCTCACGCTGGGTTGCCTGGAAAAAACACTGCAAAGGCAAAAAA---CTGGATTCCTACGTCAAG------GGGTGC---------------------------------------------------------------

---

>ZebraFinch_Calzm

---------------------------------------------------------------------------------------ATGAGAAAGTCA---------------------------------------------------ATGCTCTTCCTTGGCTTT---CTTGTTTTC

---CTTGGCCTGGCTCTGCCAGGCACCCAGGGAAAAATAATTCCCAGATGTGAGATGGTGAAGATTCTACGTCAGAATGGCTTTCAGGGCTTCGAGGGCACAACTGTTGCTGACTGGATGTGCCTGGTGAAACATGAGAGTGATTATAACACGAAAGCG------TACAATGACAATGGT

CCAAGCAGGGACTACGGCATCTTCCAGATCAACAGCAAGTACTGGTGCAACGAT------GGCAAG---ACCTCTGGATCCAAGAATGCCTGCAAAATCAGT------------TGCTCAAAACTGCAAGATGATAATCTTGGGGATGATATTCAGTGTGCCAAG---------------

---AAGATT---------GCCCGCGAGGCT---CATGGCCTCAGTCCCTGGTATGGCTGGAAAAACCATTGCCGGGGCAAAGAC---CTGAGTTCCTTTGTCAGG------GGCTGC---------------------------------------------------------------

---

>Human_Lyzl1

------------------------------------------------------------------------------------------ATGAAGGCT---------------------------------------------------GCGGGCATTCTGACCCTC---ATTGGCTGC

---CTGGTCACAGGC---------GCCGAGTCCAAAATCTACACTCGTTGCAAACTGGCAAAAATATTCTCGAGGGCTGGCCTGGACAATTACTGGGGCTTCAGCCTTGGAAACTGGATCTGCATGGCATATTATGAGAGCGGCTACAACACCACAGCCCAGACGGTCCTGGAT---GAC

GGCAGCATCGACTATGGCATCTTCCAGATCAACAGCTTCGCGTGGTGCAGACGC------GGAAAG------CTGAAGGAGAACAACCACTGCCATGTCGCC------------TGCTCAGCCTTGATCACTGATGACCTCACAGATGCAATTATCTGTGCCAGG---------------

---AAAATT---------GTTAAAGAGACA---CAAGGAATGAACTATTGGCAAGGCTGGAAGAAACATTGTGAGGGCAGAGAC---CTGTCCGAGTGGAAAAAA------GGCTGTGAGGTTTCC------------------------------------------------------

---

>Chimpanzee_Lyzl1

------------------------------------------------------------------------------------------ATGAAGGCT---------------------------------------------------GCGGGCATTCTGACCCTC---ATTGGCTGC

---CTGGTCACAGGC---------GCCGAGTCCAAAATCTACACTCGCTGCAAACTGGCAAAAATATTCTCAAGGGCTGGCCTGGACAATTACTGGGGCTTTAGCCTTGGAAACTGGATCTGCATGGCGTATTATGAGAGCGGCTACAACACCACGGCCCAGACGGTCCTGGAT---GAC

GGCAGCATCGACTACGGCATCTTCCAGATCAACAGCTTTGCTTGGTGCAGACGC------GGAAAG------CTGAAGGAGAACAACCACTGCCATGTCGCC------------TGCTCAGCCTTGATCACTGATGACCTCACAGATGCAATTATCTGTGCCAGG---------------

---AAAATT---------GTTAAAGAGACA---CAAGGAATGAACTATTGGCAAGGCTGGAAGAAACATTGTGAGGGCAGAGAC---CTGTCCGAGTGGAAAAAA------GGCTGTGAGGTTTCC------------------------------------------------------

---

>Chimpanzee_Lyzl2

------------------------------------------------------------------------------------------ATGAAGGCT---------------------------------------------------GCGGGCATTCTGACCCTC---ATTGGCTGC

---CTGGTCACAGGC---------GCCGAGTCCAAAATCTACACTCGCTGCAAACTGGCAAAAATATTCTCGAGGGCTGGCCTGGACAATTACTGGGGCTTCAGCCTTGGAAACTGGATCTGCATGGCGTATTATGAGAGCGGCTACAACACCACGGCCCAGACGGTCCTGGAT---GAC

GGCAGCATCGACTACGGCATCTTCCAGATCAACAGCTTTGCGTGGTGCAGACAT------GGAAAG------CTGAAGGAGAACAACCACTGCCACGTCGCC------------TGCTCAGCCTTGATCACTGATGACCTCACAGATGCGATTATCTGTGCCAAG---------------

---AAAATT---------GTTAAAGAGACA---CAAGGAATGAACTATTGGCAAGGCTGGAAGAAACACTGTGAGGGGAGAGAC---CTGTCCGAGTGGAAAAAA------GGCTGTGAGGTTTCC------------------------------------------------------

---

>Human_Lyzl2

------------------------------------------------------------------------------------------ATGAAGGCT---------------------------------------------------GCGGGCATTCTGACCCTC---ATTGGCTGC

---CTGGTCACAGGC---------GCCGAGTCCAAAATCTACACTCGTTGCAAACTGGCAAAAATATTCTCGAGGGCTGGCCTGGACAATTACTGGGGCTTCAGCCTTGGAAACTGGATCTGCATGGCGTATTATGAGAGCGGCTACAACACCACAGCCCAGACGGTCCTGGAT---GAC

GGCAGCATCGACTACGGCATCTTCCAGATCAACAGCTTCGCGTGGTGCAGACGC------GGAAAG------CTGAAGGAGAACAACCACTGCCACGTCGCC------------TGCTCAGCCTTGGTCACTGATGACCTCACAGATGCGATTATCTGTGCCAAG---------------

---AAAATT---------GTTAAAGAGACA---CAAGGAATGAACTATTGGCAAGGCTGGAAGAAACACTGTGAGGGGAGAGAC---CTGTCCGACTGGAAAAAA------GACTGTGAGGTTTCC------------------------------------------------------

---

>Gorilla_Lyzl1

------------------------------------------------------------------------------------------ATGAAGGCT---------------------------------------------------GCGGGCATTCTGACCCTC---ATTGGCTGC

---CTGGTCATAGGC---------GCCGAGTCCAAAATCTACACTCGCTGCAAACTGGCAAAAATATTCTCGAGGGCTGGCCTGGACAATTACTGGGGCTTCAGCCTCGGAAACTGGATCTGCATGGCGTATTATGAGAGCGGCTACAACACCACGGCCCAGACGGTCCTGGAT---GAC

GGCAGCATCGACTATGGCATCTTCCAGATCAACAGCTTCGCGTGGTGCAGACGC------GGAAAG------CTGAAGGAGAACAACCACTGCCACGTCGCC------------TGCTCAGCCTTGATCACTGATGACCTCACAGATGCGATTCTCTGTGCCAAG---------------

---AAAATT---------GTTAAAGAGACA---CAAGGAATGAACTATTGGCAAGGCTGGAAGAAACACTGTGAGGGGAGAGAC---CTGTCCGACTGGAAAAAA------GACTGTGAGGTTTCC------------------------------------------------------

---

>Orangutan_Lyzl2

------------------------------------------------------------------------------------------ATGAAGGCT---------------------------------------------------GTGGGCATTCTGACCCTC---ATTGGCTGC

---CTGGTCACAGGC---------GCCGAGTCCAAAATCTACACTCGCTGCAAACTGGCAAAAATATTCTCGAGGGCTGGCCTGGACAATTACTGGGGCTTCAGCCTTGGAAACTGGATCTGCATGGCATATTATGAGAGCGGCTACAACACCACGGCCCAGACGGTCCTGGAT---GAC

GGCAGCATTGACTATGGCATCTTCCAGATCAACAGCTTCGTGTGGTGCAGACGC------GGAAAG------CTGAAGGAGAACAACCACTGCCACGTCGCC------------TGCTCAGCCTTGATCACTGATGACCTCACAGATGCGATTATCTGTGCCAAG---------------

---AAAATT---------GTTAAAGAGACA---CAAGGAATGAACTATTGGCAAGGCTGGAAGAAACACTGTGAGGGGAGAGAC---CTGTCCGACTGGAAAAAA------AGCTGTGAGGTTTCC------------------------------------------------------

---

>Macaque_Lyzl1

------------------------------------------------------------------------------------------ATGAAGGCT---------------------------------------------------GCGGGTATTCTGACCCTG---ATTGGCTGC

---CTGGTCACGGGC---------GCGGAGTCCAAAATCTACACGCGCTGCAAACTGGCAAAAATATTCTCGAGGGCTGGCCTGGACAACTACTGGGGCTTCAGCCTTGGAAACTGGATCTGCATGGCATATTACGAGAGTGGCTACAACACCACGGCCCAGAGGGTCCTGGAT---GAC

GGCAGCATCGACTACGGCATCTTCCAGATCAACAGCTTCACGTGGTGCAGACAT------GGAAAG------CTGCAGGAGAACAACCACTGCCACGTCGCT------------TGCTCAGCCTTGATCACTGATGACCTCACAGATGCGATTATCTGTGCCAGG---------------

---AAAATT---------GTTAAAGAGACA---CAGGGAATGAACTATTGGCAAGGCTGGAAGAAACACTGTGAGGGCAGAGAC---CTGTCTGACTGGAAAAAA------GGCTGTGAGGTTTCC------------------------------------------------------

---

>Baboon_Lyzl1

------------------------------------------------------------------------------------------ATGAAGGCT---------------------------------------------------GCGGGTATTCTGACCCTC---ATTGGCTGC

---CTGGTCACAGGC---------GCTGAGTCCAAAATCTACACGCGCTGCAAACTGGCAAAAATATTCTCGAGGGCTGGCCTGGACAACTACTGGGGCTTCAGCCTTGGAAACTGGATCTGCATGGCGTATTACGAGAGCGGCTACAACACCACTGCCCAGAGGGTCCTGGAT---GAC

GGCAGCATCGACTACGGCATCTTCCAGATCAACAGCTTCACGTGGTGCAGACAT------GGAAAG------CTGCAGGAGAACAACCACTGCCACGTCGCT------------TGCTCAGCCTTGATCACTGATGACCTCACAGATGCAATTATCTGTGCCAGG---------------

---AAAATT---------GTTAAAGAGACA---CAGGGAATGAACTATTGGCAAGGCTGGAAGAAACACTGTGAGGGCAGAGAC---CTGTCTGATTGGAAAAAA------GGCTGTGAGGTTTCC------------------------------------------------------

---

>Macaque_Lyzl2

------------------------------------------------------------------------------------------ATGAAGGCT---------------------------------------------------GCGGGTATTCTGACCCTG---ATTGGCTGC

---CTGGTCACGGGC---------GCGGAGTCCAAAATCTACACGCGCTGCAAACTGGCAAAAATATTCTCGAGGGCTGGCCTGGACAACTACTGGGGCTTCAGCCTTGGAAACTGGATCTGCATGGCATATTACGAGAGCGGCTACAACACCACCGCCCAGAGGGTCCTGGAT---GAT

GGCAGCATCGACTACGGCATCTTCCAGATCAACAGCTTCACGTGGTGCAGACAT------GGAAAG------CTGCAGGAGAACAACCACTGCCACGTCGCT------------TGCTCAGCCTTGATCACTGATGACCTCACAGATGCGATTATCTGTGCCAGG---------------

---AAAATT---------GTTAAAGAGACA---CGAGGAATGAACTATTGGCAAGGGTGGAAGAAACACTGTGAGGGGAGAGAC---CTGTCCGACTGGAAAAAA------GGCTGTGAGGTTTCC------------------------------------------------------

---

>Orangutan_Lyzl1

------------------------------------------------------------------------------------------ATGAAGGCT---------------------------------------------------GTGGGCATTCTGACCCTC---ATTGGCTGC

---CTGGTCACAGGC---------GCCGAGTCCAAAATCTACACTCGTTGCAAACTGGCAAAAATATTCTCGAGGGCTGGCCTGGACAATTACTGGGGCTTCAGCCTTGGAAACTGGATCTGCATGGCGTATTATGAGAGCGGCTACAACACCACGGCCCAGACGGTCCTAGAT---GAT

GGCAGCATTGACTACGGCATCTTCCAGATCAACAGCTTCGCGTGGTGCAGACGC------GGAAAG------CTGAAGGAGAACAACTACTGCCACGTCGCC------------TGCTCAGCCTTGATCACTGATGACCTCACAGATGCGATTATCTGTGCCAGG---------------

---AAAATT---------GTTAAAGAGACA---CAAGGAATGAACTATTGGCAAGGCTGGAAGAAACACTGTGAAGGCAGAGAC---CTGTCCGAGTGGAAAAAA------GGCTGTGAGGTTGTTTCC---------------------------------------------------

---

>Marmoset_Lyzl1

------------------------------------------------------------------------------------------ATGAAGGCT---------------------------------------------------GTGGGCCTTTTGACCCTG---ACTGGCTGC

---TTGGTCACAGGC---------GCCGAGTCCAAAATCTACACTTGCTACAAACTGGCAAAAATATTCTCGAGGGCTGGCCTGGACAATTATGGGGGCTTCAGCCTTGGAAACTGGATCTGCATGGTATATTACGAGAGCGGCTACAACACCACGGCCCAGACGGTCCTGGAT---GAT

GGCAGCATCGACTACGGCATCTTCCAGATCAACAGCTTCACCTGGTGCAGACAA------GGAAAG------CTGCAGGAGTGGAACCACTGCCACGTCGCC------------TGCTCAGCCTTGATCACTGATGACCTCACAGATGCGATTATCTGTGCCAGG---------------

---AAAATT---------GTTAAAGAGACA---CAAGGAATGAACTATTGGCAAGGCTGGAAGAAACACTGCGAGGGCAGAGAC---CTGTCCAAGTGGAAAAAA------GGCTGTGAGGTTTCC------------------------------------------------------

---

>Marmoset_Lyzl2

------------------------------------------------------------------------------------------ATGAAGGCT---------------------------------------------------GTGGGCCTTTTGACCCTG---ATTGGCTGC

---TTGGTCACAGGC---------GCCGAGTCCAAAATCTACACTCGCTGCAAACTGGCAAAAATATTCTCGAGGGCTGGCCTGGACAATTATGGGGGCTTCAGCCTTGGAAACTGGATCTGCATGGCGTATTACGAGAGCGGCTACAACACCACGGCCCAGACGGTCCTGGAT---GAT

GGCAGCATCGACTACGGCATCTTCCAGATCAACAGCTTCACCTGGTGCAGACAA------GGAAAG------CTGCAGGAGCGGAACCACTGCCACGTCGCC------------TGCTCAGCCTTGATCACTGATGACCTCACAGATGCGATTATCTGTGCCAGG---------------

---AAAATT---------GTTAAAGAGACA---CAAGGAATGAACTATTGGCAAGGCTGGAAAAAACACTGCGAGGGCAGAGAC---CTGTCCAAGTGGAAAAAA------GGCTGTGAGGTTTCC------------------------------------------------------

---

>Mouse_lemur_Lyzl2

------------------------------------------------------------------------------------------ATGAAGGCT---------------------------------------------------GCAGGGGTCCTGGCCCTG---ATCGGCTGT

---CTGGTCACAAGC---------ACGGAGTCCAAGGTCTACACGCGCTGCAAACTGGCAAAAATATTTTCAAGGGCTGGCCTGGACAATTACGCGGGCTTTAGCCTTGGAAACTGGATCTGCATGGCGTACTACGAGAGCGGCTACAACACCACCGCGCAGACCACGCTGGAC---GAC

GGCAGTGTCGACTACGGCATTTTTCAGATAAACAGTTTCACGTGGTGCAGATAC------GAAAAG------GTGCAAGAGAAGAACCACTGTCACGTTGCC------------TGCTCAGCCTTGATCACGGACGACCTCACCGATGCGATTATTTGTGCCAAG---------------

---AAAATC---------ATTAAAGAGACG---CAAGGGATGAACTATTGGCAGCGCTGGAAGAAGCACTGTGAGGGCAGAGAC---CTGTCCGAGTGGAAGAAA------GGGTGTGAGGTCTCG------------------------------------------------------

---

>Pika_Lyzl2

------------------------------------------------------------------------------------------ATGAAGGCT---------------------------------------------------GTGGCTGTTCTGGCCCTG---GCCTGCTGC

---CTGGCCACCATC---------ATCGAATCCAAAGTCTACACTCGTTGCAAACTGGCCAAGATATTTTCAAGGGCTGGCCTGGACAATTACGAGGGCTTTAGCCTTGGAAACTGGATCTGTATGGCATACTACGAAAGCCACTACAACACCACGGCCCAGACCGTCCTGGAA---GAC

GGAAGCATCGACTATGGCATCTTCCAGATCAACAGTTACACGTGGTGCCGACGT------GGGAAG------ACCCAGGAGAAGAACCTCTGCCATGTCGCC------------TGCTCAGCCTTGCTCACGGATGATCTCACCGATGCCATCATCTGTGCCAAG---------------

---AGGATC---------ATCAAAGACACC---CAGGGCATGAACTATTGGCAAAGCTGGAAGAAGCACTGTGAGGGCAGGGAA---CTGTCCGAGTGGAAGAAA------GGGTGCGAGGTTTCC------------------------------------------------------

---

>Rabbit_Lyzl2

------------------------------------------------------------------------------------------ATGAGGGCT---------------------------------------------------GCTGCCATCCTGGCCCTG---GTTGGCTGC

---CTGGCCACCGTC---------ACTGAATCCAAAGTCTACACTCGCTGTAAACTGGCAAAAATATTTTCGAGGGCCGGCCTGGACAATTACGAGGGCTTTAGCCTTGGAAACTGGATCTGCATGGCGTACTACGAAAGCCACTACAACACCACGGCTCAGACCGTCCTGGAG---GAT

GGGAGCATTGACTACGGCATTTTCCAGATAAACAGTTTCACGTGGTGCAGACGT------GCGAAG------ACGCAGGAGAAGAACCATTGCCACGTTGCC------------TGCTCAGCCTTGCTCACAGACGACCTCACGGATGCGATCATCTGTGCCAAG---------------

---AAAATC---------GTGAAAGATACC---CAGGGGATGAACTACTGGCAAGGCTGGAAGAAGCACTGTGAGGGCAAGGAG---TTGTCTGAGTGGAAGAAA------GGGTGTGAGGTTTCC------------------------------------------------------

---

>Pig_Lyzl2

------------------------------------------------------------------------------------------ATGAAGGCT---------------------------------------------------ACTGGAATATTGGCCCTG---ATGGGCTGC

---CTGATCACAGTC---------ATTGAGCCTAAAATCTACACTCGCTGTAAACTGGCAAAAATATTTTCGAGGGCTGGCCTGGATAATTACCGGGGCTTTAGCCTTGGAAACTGGATCTGCATGGCCTACTATGAGAGCCACTACAACACCACGGCTCAGACCAACCTGGAG---GAT

GGCAGCACAGACTATGGCATTTTTCAGATCAACAGTTACACATGGTGCAGACGT------GAGAAG------CTGCAAGAGAAAAACCACTGCCACGTAGCC------------TGCTCAGCTTTGATCACCGACGACCTCACGGATGCAATTATCTGTGCCAAG---------------

---AAAATT---------GTGAAAGAGACA---GAGGGAATGAACTATTGGCAAGGCTGGAAGAACCATTGTGAGGGCAAAGAC---CTGTCTGAGTGGAAAAAG------GGATGCGAGGTTTCC------------------------------------------------------

---

>Cat_Lyzl2

------------------------------------------------------------------------------------------ATGAAGGCT---------------------------------------------------GCCAGCATCTTGGCCCTG---ATTGGCTGC

---CTGGTCACCGTC---------ACTGAGTCCAAAGTCTACACTCGCTGTAAACTGGCAAAAATATTTTCAAGGGCTGGCCTGGACAATTACCAGGGTTTTAGCCTTGGAAACTGGATCTGCATGGCATACTATGAGAGCCACTACAATACGACAGCGCAGACCCAGCTGGAG---GAT

GGAAGCACTGACTATGGCATTTTCCAGATAAACAGCTTCACGTGGTGCAGACAT------GCTAAG------CTACAGGAGAAGAACCACTGTCACGTCGCC------------TGCTCAGCCTTGCTCACTGACGACCTCACAGATGCGATTATCTGTGCCAAG---------------

---AAAATT---------GCTAAAGAGACA---GAAGGGATGAACTATTGGCAAGGCTGGAAGAAACACTGCGAGGGCAAGGAC---CTGTCTGAATGGAAAAAG------GGATGTGAGGTCTCC------------------------------------------------------

---

>Horse_Lyzl2

------------------------------------------------------------------------------------------ATGAAGGCT---------------------------------------------------GCCGGCATCTTGGCCCTG---ATTGGCTGC

---CTGGTCGCAGTC---------ACCGAGCCCAAAGTCTACACTCGCTGTAAACTGGCAAAAATATTTTCGAGGGCTGGCCTGGACAATTACCGGGGCTTTAGCCTTGGAAACTGGATCTGCATGGCATACTACGAGAGCCACTACAACACAACGGCTCAGACTGTCCTGGAG---GAT

GGGAGCACTGACTATGGCATTTTTCAGATAAACAGTTTCACGTGGTGCAGAAGT------GCGAAG------CTGCAAGACAAGAACCACTGTCATGTTGCC------------TGCTCAGCCTTGCTCACTGATGACCTCACAGATGCGATTATCTGTGCCAAG---------------

---AAAATT---------GTTAAAGAGACA---GAAGGGATGAACTACTGGCAAGGCTGGAAGAAACACTGTGAAGGCAAAGAC---TTGTCTGAGTGGAAAAAG------GGATGTGAAGTTTCG------------------------------------------------------

---

>Dog_Lyzl2

------------------------------------------------------------------------------------------ATGAAGGCT---------------------------------------------------GCCGGCATCTTGGCCCTG---ATTGGCTGT

---CTGATCATGGTC---------ACTGAGCCCAAAATCTACACTCGCTGTAAACTGGCAAAAATATTTTTGAGGGCTGGCCTGGACAATTACCAGGGTTTTAGCCTTGGAAACTGGATCTGCATGGCATACTATGAGAGCCGCTACAATACAACAGCTGAGACCCAGCTGGAG---GAT

GGAAGCATTGACTATGGCATTTTTCAGATAAATAGTTTCACGTGGTGCAGACGT------GCTAAG------CTACAAGAGAAGAACCACTGTCACGTTGCC------------TGCTCAGCCTTGATCACAGATGATCTCACAGATGCAATTCTATGTGCCAAG---------------

---AAAATT---------GCTAAAGAGACA---GAAGGGATGAACTACTGGCAAGGCTGGAAGAAACACTGTGAGGGCAAGGAC---CTGTCTGAGTGGAAAAAG------GGATGTGAGGTTTCA------------------------------------------------------

---

>Treeshrew_Lyzl2

------------------------------------------------------------------------------------------ATGAAGGTT---------------------------------------------------GCTGCCGTCTTGACCCTG---CTTGGGTTC

---CTGGTCACAGCC---------ACGGAGTCCAAAGTCTACACTCGTTGTAAACTGGCAAAAATATTTTCGAGAGCTGGCCTGGACAATTACGGAGGCTTTAGCCTTGGAAACTGGATCTGCATGGCATACTACGAGAGCCACTACAACACCACGGCTCAGACAATCCTGGAG---GAC

GGGAGCGTCGACTATGGCATATTTCAGATAAACAGTTTCACGTGGTGCAGACAT------GCGAAG------CTGCAGGAGAAGAACCACTGTCATGTGGCC------------TGCTCAGCCTTGGTCACTGATGACCTCACGGATGCCATTATCTGTGCCAAG---------------

---AAAATT---------GTCAAGGAGACA---GAGGGGATGAACTACTGGCAAGGCTGGAAGAAGAACTGTGAAGGCAAAGAC---GTGTCCGAGTGGAAAAAA------GGATGTGAGGTTTCA------------------------------------------------------

---

>Cow_Lyzl2

------------------------------------------------------------------------------------------ATGAAGGCT---------------------------------------------------GCTGGTATTTTGGCCCTG---ATGGGCTGC

---CTGGTCACAGTC---------GTTGAGCCCAAAGTCTACACTCGATGTAAACTGGCAAAAATATTTTCGAGGGCCAGCCTGGACAATTACCGAGGCTTTAGCCTTGGAAACTGGATCTGCATGGCCTACTATGAGAGCCACTACAACACCACGGCTCAGACCCAACTGGAG---GAC

GGAAGCACCGACTATGGCATCTTTCAGATAAACAGCGACACGTGGTGCAGAAGT------ACAAAG------CTACAAGAGAAAAACCGCTGTCACGTGGCC------------TGCTCAGCCTTGATGACTGATGACCTCACGGATGCAATTATCTGTGCCAAG---------------

---AAAATT---------GTTAAAGAAACG---GATGGCATGAACTACTGGCAAGGCTGGAAGAAGAACTGTGAGGGCAGAGAC---CTGTCTGAGTGGAAAAAG------GGATGTGAGGTTTCA------------------------------------------------------

---

>Elephant_Lyzl2

------------------------------------------------------------------------------------------ATGAAGGCT---------------------------------------------------GTCAGCATCCTGGCCCTG---ATAGGCTGT

---GTGGCCACGGTC---------GTCGAGTCCAAAGTCTACACTCGCTGTAAACTGGCAAAAATATTTTCGAGGGCTGGCCTGGACAATTACCGGGGTTTTAGCCTTGGAAACTGGATCTGCCTGGCGTACTATGAGAGTCACTATAACACCACGGCCCAGAATGTACTGGAT---GAC

GGTAGCATCGACTATGGCATTTTTCAGATAAACAGTTACACGTGGTGCAGGAGC------GGGACA------CTACAGCAGAAGAACCATTGTCATGTTGCC------------TGCTCAGCCTTGATCACTGACGACCTCACAGATGCAATTATCTGTGCCAAG---------------

---AAAATT---------GTGAAAGAAACA---GAAGGGATGAACTATTGGCAAGGCTGGAGGAAATACTGTAAGGGCAAAGAC---CTGACCGAATGGAAGAAG------GGATGTGAGGTTTCA------------------------------------------------------

---

>Tenrec_Lyzl2

------------------------------------------------------------------------------------------ATGAAGGCT---------------------------------------------------ATCGGCATCCTGGCCCTG---ATGGGCTGT

---GTGGCCACGGTC---------ATCGAGGCCAAAATCTACACTCGCTGTAAACTGGCTAAAATATTTTCGAGAGCCGGCCCGGACAATTACCAAGGTATCAGCCTTGGAAACTGGATCTGCCTGGCATATTATGAGAGTCACTACAACACCACAGCCCAGACAGTCCTAGAG---GAT

GGAAGTATCGACTACGGCATCTTCCAGATAAACAGCTTTACCTGGTGCCGAAGT------GGCAAA------CTGCAGCAGAAGAACCACTGCCATGTCGCC------------TGCTCAGCCTTGATCACCGACGACCTCACAGATGCAATTATCTGTGCCAAG---------------

---AAAATC---------GTGAAAGAGACC---GAGGGGATGAACTACTGGCAAGGCTGGAAGCAATACTGTGAAGGCAAAGAC---CTGTCCGAATGGAAAAAG------GGATGTGAGGTTTCA------------------------------------------------------

---

>Squirrel_Lyzl2

------------------------------------------------------------------------------------------ATGAAGGCT---------------------------------------------------GCTGGCATCTTGTCCCTG---ATTGGCTGT

---CTGGTCACAGTC---------ACAGACTCCAAAATCTACACTCGCTGCAAACTGGCAAAAGTATTTGCCAGGGCCGGTCTGGACAATTACCAGGGCTTTAGCCTGGGAAACTGGTTCTGCATGGCATACTATGAGAGCGGCTACAACACCACAGGTCAGACGGGCGTGGAT---GTA

CACAGTATTGACTATGGCATTTTTCAGATAAATAGCTTCACGTGGGGCAGAGAT------GCGGAG------CTGCAAGAGAAGAACCACTGTCATGTTGCC------------TGCTCAGCCTTAATCACTGATGATCTCACAGATGCAATTATCTGTGCCAAG---------------

---AAAATT---------GCTAAAGAGACT---CAAGGGATGGACTATTGGCAAGGCTGGAAGAAACACTGTGAGGGCAGAGAA---CTATCTGAGTGGAAAAAA------GGATGTGAAGTTTCC------------------------------------------------------

---

>Armadillo_Lyzl2

------------------------------------------------------------------------------------------NNNNNNNCT---------------------------------------------------GTCGGCATCCTGGCGCTG---ATTGGCTGC

---TTC---ACGGTC---------CTTGAGTCCAAAGTCTACACTCGCTGTAAACTGGCAAAAATATTTTCGAGGGCCAACCTGGACAATTACTGGGGTTACAGCCTTGGAAACTGGATCTGCATGGCCTACTACGAGAGCCACTACAACACTTCAGCCCAGAAGGTGCTGGAA---GAC

GGAAGCATCGACTACGGCATCTTTCAGATAAACAGCTTCACCTGGTGCCGAAGT------AAGAAG------CTGCAGGAGAAGAACCACTGCCATGTAGCC------------TGCTCAGCCTTGATCACTGATGACCTCACAGATGCGATTATCTGCGCCAAG---------------

---AAAATC---------GTTAAAGAAACG---GACGGGATGAACTACTGGCAAGGCTGGAAG---CACTGTGAGGGCAGAGAA---CTGTCTGAG---AAAAAG------GGATGTGAGGTTTCA------------------------------------------------------

---

>Guinea_pig_Lyzl2

------------------------------------------------------------------------------------------ATGAAGGCC---------------------------------------------------ACTGGGGTCCTGCTCCTG---ATTGGCTTG

---CTGGTCACAGAC---------ATAGAGTCCAGAGTCTACACTCGCTGTAAACTGGCAAAGATATTCTCAAGGGCTGGCCTGGATAATTACGCAGGCTTCGTCCTTGGCAACTGGATCTGCATGGCGTATTATGAGAGCCGCTACAACACCTCGGCTCAGACTGTCCTGGAT---GAC

GGCAGCACTGACTATGGCATTTTCCAGATCAACAGCTTCACGTGGTGCAGAGAC------AGCAGT------TTCCAG---AAGAACCATTGCCACGTCGCC------------TGCTCAGCCTTGCTCTCAGATGACCTCACAGACGCAATTATCTGTGCCAAG---------------

---AAAATT---------GTTAAAGAGACA---CAAGGGATGAACTACTGGCAAGGCTGGAAGAAACACTGTGAAGGTCAAGAT---CTGTCTGAGTGGAAAAAA------GGGTGTGATGTTTTA------------------------------------------------------

---

>Mouse_Lyzl2

------------------------------------------------------------------------------------------ATGAAGTCT---------------------------------------------------GTTGGTGTCTTCGCCCTA---ATTATCAGC

---TTCAGCATAGTC---------GCAGAATCCAAAATCTACACTCGATGTAAACTGGCAAAAATCTTTGCAAAGGCCGGCCTGGACAATTACGGGGGATTTGCCCTTGGAAACTGGCTCTGCATGGCATACTATGAGAGCCACTACAACACTACAGCCGAGAATGTCCTGGAG---GAT

GGAAGCACTGACTATGGCATTTTCCAGATAAACAGTTTCACATGGTGCAGAAAT------GCAAGA------AAGCATCAGAAGAATCACTGCCATGTTGCC------------TGCTCAGCCTTGATCACTGATGACCTCACAGATGCCATTCTCTGTGCCAAG---------------

---AAAATT---------GTTAAAGAGACA---CAAGGGATGAACTATTGGCAGGGCTGGAAGAAGAACTGTGAGAACAAAGAC---ATGTCAGAGTGGAAGAGA------GGATGTGAGGTTTCC------------------------------------------------------

---

>Rat_Lyzl2

------------------------------------------------------------------------------------------ATGAAGGCT---------------------------------------------------GTCGGTGTCTTCGCCCTA---ATTATGAGC

---CTCGGCATAGTA---------GCAGAATCCAAAGTCTACACTCGATGTAAACTGGCAAAAGTCTTTGTGAAGGCCGGCCTGGACAATTACGGGGGATTTACCCTTGGAAACTGGCTCTGCATGGCATACTATGAGAGTCACTACAACACTTCAGCAGAGACTGTCCTGGAG---GAC

GGGAGCACTGACTATGGCATTTTCCAGATAAACAGCTTCACGTGGTGCAGAAAT------GGAAAA------AAACATCAGAAGAATCACTGCCATGTTGCC------------TGCTCAGCACTGACCACTGATGACCTCACAGATGCCATTCTCTGTGCCAAG---------------

---AAAATT---------GTTAAGGAGACA---CAAGGGATGAACTATTGGCAGGGCTGGAAGAAGAACTGTGAGAGCAAAGAC---TCGTCTGATTGGAAAAGA------GGATGCGATGTTTCC------------------------------------------------------

---

>Platypus_Lyzl2

------------------------------------------------------------------------------------------ATGAAGGCC---------------------------------------------------CTCGGCCTCCTGCTTCTG---ATTGACTGC

---CTGATCACATTC---------AGTCAAGCTAAAATCTATTCCCGCTGCAAACTGGCACGGAGATTGGAGCACAGTGGAATGGACAAATTTAGGGGCTACAGCCTTGAAAACTGGCTCTGTGTGGCATATTACGAGAGTGGTTTTGACACTGAAGCTCAGAACACTTTGGAA---GAC

GGCAGCACTGCTTACGGCATCTTCCAGATCGACAGCCGGATATGGTGCAAAAAC------GATGAG------GAGCCATCCAAGAACCGCTGTCACATTTCC------------TGCTCTGATTTATTAAATGATGATATAAGTGACGACATAATGTGTGCTAAG---------------

---AAAATT---------GTGAAGGAGACT---GAGAGCATGGATTACTGGGACAGCTGGAAGATTCACTGTGAAGACAAGGAC---CTCTCTGAGTGGATAGAA------GGCTGCAATGACAAC------------------------------------------------------

---

>Human_Spaca3

------------------------------------------------------------------------------------------------------------------------------------------------ATGTTGTTGGCCCTGGTCTGTCTG---CTCAGCTGC

---CTGCTACCCTCC---------AGTGAGGCCAAGCTCTACGGTCGTTGTGAACTGGCCAGAGTGCTACATGACTTCGGGCTGGACGGATACCGGGGATACAGCCTGGCTGACTGGGTCTGCCTTGCTTATTTCACAAGCGGTTTCAACGCAGCTGCTTTGGACTACGAGGCT---GAT

GGGAGCACCAACAACGGGATCTTCCAGATCAACAGCCGGAGGTGGTGCAGCAAC------CTCACC------CCGAACGTCCCCAACGTGTGCCGGATGTAC------------TGCTCAGATTTGTTGAATCCTAATCTCAAGGATACCGTTATCTGTGCCATG---------------

---AAGATA---------ACCCAAGAGCCT---CAGGGTCTGGGTTACTGGGAGGCCTGGAGGCATCACTGCCAGGGAAAAGAC---CTCACTGAATGGGTGGAT------GGCTGTGACTTC---------------------------------------------------------

---

>Gorilla_Spaca3

------------------------------------------------------------------------------------------------------------------------------------------------ATGTTGTTGGCCCTGGTCTGTCTG---CTCAGCTGC

---CTGCTACCCTCC---------AGTGAGGCCAAGCTCTACGGTCGNTGTGAACTGGCCAGAGTGCTACATGACTTCGGGCTGGACGGATACCGGGGATACAGCCTGGCCGACTGGGTCTGCCTTGCTTATTTCACAAGCGGTTTCAATGCAGCTGCTTTGGACTACGAGGCT---GAT

GGGAGCACCAACAACGGGATCTTCCAGATCAACAGCCGGAGGTGGNGCAGCAAC------CTCACC------CCGAACGTCCCCAACGTGTGCCGGATGTAC------------TGCTCAGATTTGTTGAATCCTAATCTCAAGGATACCGTTATCTGTGCCATG---------------

---AAGATA---------ACCCAAGAGCCT---CAGGGTCTGGGTTACTGGGAGGCCTGGAGGCATCACTGCCAGGGAAAAGAC---CTCACTGAATGGGTGGAT------GGCTGTGACTTC---------------------------------------------------------

---

>Macaque_Spaca3

------------------------------------------------------------------------------------------------------------------------------------------------ATGTTGTTGGCCCTGGTCTCTCTG---CTCAGCTGC

---CTGCTACCCTCC---------AGTGAGGCCAAGGTCTACAGTCGCTGTGAACTGGCCAGAGTGCTACAGGACTTCGGGCTGGACGGATACCGGGGATACAGCCTGGCTGACTGGGTCTGCCTTGCTTATTTCACAAGCGGTTTCAACACAGCTGCTGTGGACCACGAGGCT---GAC

GGGAGCACCAACAACGGGATCTTCCAGATCAGCAGCCGGAGGTGGTGCAGAAAC------CTCACT------CCCAACGCCCCCAACATGTGCGGGATGTAC------------TGCTCAGATTTGTTGAATCCTAATCTCAAGGATACCGTTATCTGTGCCATG---------------

---AAGATC---------ACCCAAGAGCCT---CAGGGTCTGGGTTACTGGGAGGCCTGGAGGCATCACTGCCAGGGCAAAGAC---CTCACTGACTGGGTGGAT------GGCTGTGACTTC---------------------------------------------------------

---

>Marmoset_Spaca3

------------------------------------------------------------------------------------------------------------------------------------------------ATGTTGTTGGCCCTGGTCTCTCTG---CTCAGCTGC

---CTGTTCCCCTCC---------AGTGACGCCAAGGTCTACGGTCGCTGTGAACTGGCCAGAGTGCTACAGGACTTCGGGCTGGACGGATACCGGGGATACAGCCTGGCTGACTGGGTCTGCCTTGCTTATTTCGCAAGCGGTTTCAACACAGCTGCTGTGGACCACGAGGCT---GAT

GGAAGCACCAACAACGGCATCTTCCAGATCAGCAGCCGGAGGTGGTGCAGAAAC------CTCACC------CCACACGTCCCCAACATGTGCCAGATGTAC------------TGCTCAGATTTGTTGAATCCTAATCTCAAGGATACCGTCATCTGTGCCATG---------------

---AAGATT---------ACCCAAGAGCCT---CAGGGTCTGGGTTACTGGGAGGCCTGGAGGCATCACTGCCAGGGCAAAGAC---CTCACGGACTGGGTGGAT------GGCTGTGACTTC---------------------------------------------------------

---

>Kangaroo_rat_Spaca3

------------------------------------------------------------------------------------------ATGGAAACC---AGGAGCCCAGCTCCCAGAAAGCAGCTGTGCCCCTCTGGGATTGCAGTGCTGGCTTTCCTGTCTCTC---CTTAGCTGC

---CTACTCCCCTCC---------AGTGAGGCCAAGGTCTACAGTCGCTGTGAGCTGGCCAGAGTGCTCCAGAACTTTGGTCTGGATGGCTTTCGGGGATACGACTTGGCTGACTGGGTCTGCCTTGCTTATTTCACAAGTGGTTTCAACACGGCTGCTGTGGATCATGAAGCT---GAT

GGCAGCACCAACAATGGCCTCTTCCAGATCAGCAGCCGGAGGTGGTGCAAAAAC------CTCACC------CCAAATGCCATCAACATCTGCCGGATGTAC------------TGCACTGACTTGTTGAACCCTAATCTCAAGGACACTGTTATCTGTGCCATG---------------

---AAGATA---------GCCCAAGAGCCT---CAAGGCCTAGGCTATTGGGAGGCCTGGAGGCATCACTGCCAGGGCAAGGAT---CTTCGAGACTGGGTTGAT------GGCTGCGACTTCATG------------------------------------------------------

---

>Dog_Spaca3

------------------------------------------------------------------------------------------ATGGAAGCC---AGGAGCTGGGCTCCCAGAGGGCATCTGTGCCCACCGGGCAGCATGTTGCTGGCCTTTGCCTCTCTG---CTCGGCTGC

---CTGCTCACCTCC---------AGCCAAGCCAGGGTCTACAGTCGCTGCGAGCTGGCCAAAGTGCTCCAGGATTTCGGCATGGAGGGATACCGGGGATACACCCTGGCTGACTGGGTCTGTCTTGCATATTTCACAAGTGGCTTCAACACAGCTGCTGTGGACCACGAGGCT---GAT

GGAAGTACTAACAACGGCATTTTCCAGATCAACAGCCGGAAGTGGTGCAAAAAT------CTCAAC------ACAGAGGTCCCCAATGTGTGCCAGATGTAC------------TGCTCTGACTTGTTGAATCCTAACCTTAAGGATACTGTTATCTGTGCCATG---------------

---AAGATC---------ACTCAACAGCCC---CAAGGTCTGGCCAGCTGGGAGGCCTGGAGGCGTCACTGCCAGGGCAAGGAC---CTCACAGACTGGGTGGAT------GGCTGTGACTTG---------------------------------------------------------

---

>Cat_Spaca3

------------------------------------------------------------------------------------------ATGGAAGCC---AGGAGCTGGGCCCCCAGAAGGCAACTGTGCCCGCCGGGCATCATGCTGCTGGCCTTTGCTACTCTG---CTCAGCTGC

---CTGCTCACCTCT---------GGCCAGGCCAAGGTCTACAGTCGCTGTGAGCTGGCCAGAGCCCTCCAGGATTTCGGCATGGAGGGATACCGGGGATACAGCATGGCTGACTGGGTCTGTCTTGCTTACTTCACAAGTGGCTTCAATACAGCTGCTGTGGACCATGAAGCC---AAT

GGGAGCACCAACAATGGCATCTTCCAGATCAGCAACCGGAAGTGGTGCAAAAAT------CTCAGC------ACAGATGTCCCCAACTGGTGCCAGATGTAC------------TGCTCCGACTTGCTGAATCCTAACCTTAAGGACACTGTCATCTGTGCCATG---------------

---AAGATA---------GCTCAACAGCCC---CAGGGGCTGGCCAGCTGGGAGGCCTGGAGGCGTCACTGCCAGGGCAAGGAC---CTCAAGGACTGGGTGGAT------GGCTGTGACTTG---------------------------------------------------------

---

>Fox_bat_Spaca3

------------------------------------------------------------------------------------------ATGGAAGCC---AGGAGCTGGGCTCCCAGAAGGCAGCCGTGCCCACCCGGGATCGGACTGCTGGCCTTGGCCTCTCTG---CTCAGCTGC

---CTGCTCACCTCC---------AGCCAGGCCAAGGTCTACAGTCGCTGTGAGCTGGCCAGAGTGCTCACGGATTTCGGCCTCGAGGGATATCGGGGCTACAGCCTGACTGACTGGATCTGTCTTGCTTATTTCACAAGTGGTTTCAACACAGCTGCTGTAGACCACGAAGCT---GAT

GGGAGCACCAACAATGGCATCTTCCAGATCAGCAGCCGGAAGTGGTGCAAAAAT------TTCACC------CCAAATGTCCCCAACCAGTGCCAGATGTAC------------TGCTCCGACTTGTTGAATCCTAACCTCAAGGATACTGTTATCTGTGCCATG---------------

---AAGATA---------ATTCAAGAGCCC---CAGGGTCTGAGCAGCTGGGAGGCCTGGAGGCATCACTGCCAGGGCAAGGAC---CTCAGAGACTGGGTGGAT------GGCTGTGACTTC---------------------------------------------------------

---

>Cow_Spaca3

------------------------------------------------------------------------------------------ATGGAAGCC---GGGAGCTGGGCTCCCAGAAGGTGGCCCCGCCCGCCCGGGATCGTGCTGCTGGCCTTGGCTTCTGTC---CTCAGCAGC

---CTGCTCTCCTCC---------GGCCAGGCCAGGGTCTACAGCCGCTGCGAGCTGGCCAGGGTGCTTCAGGATTTTGGCTTGGAGGGATACCGGGGCTACAGCTTGGCTGACTGGATCTGTCTTGCTTACTTCGCAAGCGGCTTCAACACAGGTGCCGTGGACCATGAAGCC---GAT

GGCAGTACCAACAGCGGCATCTTCCAGATCAACAGCCGAAAGTGGTGCAAAAAC------CTCAAC------CCTAATGTCCCAAACTTGTGCCAGATGTAC------------TGCTCCGACTTATTGAATCCCAACCTCAAGGATACTGTTATCTGTGCCATG---------------

---AAGATA---------ACTCAAGAACCC---CAGGGTCTGGGCAGCTGGGAGGCATGGAGGCATCACTGCCAGGGCAAAGAC---CTCAGTGACTGGGTGGAT------GGCTGTGAGCTG---------------------------------------------------------

---

>Dolphin_Spaca3

------------------------------------------------------------------------------------------ATGGAAGGT---GGGAGCTGGGCTCCCAGAAGGTGGCCGTGCCTGCCTGGGATGACGTCACTGGCCTTGGCGTCTCTG---CTCAGCTGC

---CTGCTCACCTCC---------GGCCAGGCCGAGGTCTACAGTCGCTGTGAGCTGGCCAGAATGCTGCAGGATTTCGGCCTGGATGGCTTCCGGGGATACAGCCTGGCGGACTGGGTCTGTCTTGCTTACTTCGCAAGTGGCTTCAACACAGCTGCCGTGGACCACGAAGCC---GAT

GGAAGTACCAACAATGGCATCTTCCAGATCAACAGCCGGAAGTGGTGCAAAAAT------CTCAAC------CCCAAGGTCCCAAACATGTGCCAGATGTAC------------TGCTCCGACTTGTTGAATCCTAACCTCAAGGATACTGTTATCTGTGCCATG---------------

---AAGATA---------GTTCAGGATCCC---CAGGGTCTGGGCACCTGGGAGGTCTGGAGGCATCACTGCCAGGGCGAGGAC---CTCAGTGACTGGGTGGAT------GGCTGTGAATTG---------------------------------------------------------

---

>Pig_Spaca3

------------------------------------------------------------------------------------------ATGGAAGCC---CGGAGCCGGGCTCCCAGGAGGCGGCTGTGCCCACCCGGGATCGTGTTGTTGACCTTGGCCTCTCTG---CTCAGCTGT

---CTGCTCACCTCC---------GGCCAGGCCAAGGTCTACAGTCGCTGCGAGCTGGCCAGACTGCTCCAGGATGTAGGCTTGGATGGATTCCGAGGATACAGCCTGGCTAACTGGGTCTGTCTGGCTTACTTCGCAAGTGGCTTCAACACAGCTGCTGTGGACCACGAAGCA---GAT

GGAAGCACCAACAGTGGCATCTTCCAGATCAACAGCCGGAAGTGGTGCAGAAAT------CTCAAC------CCCAGTGTCCTCAACTTGTGCCAGATGTAC------------TGCTCTGACTTGTTGAATCCTAACCTCAAGGATACTATTATCTGTGCCATG---------------

---AAGATA---------GCTCAAGATCCC---AAGGGGCTGGGGACCTGGGAGGCCTGGAGGCACCACTGCCAGGGCAAGGAC---CTCACTGACTGGGTCGAT------GGCTGTGACTTA---------------------------------------------------------

---

>Horse_Spaca3

------------------------------------------------------------------------------------------ATGGATGCC---AGGGGCTGGCCTCCCGGAAGGTGGCTGTGCCCGCCCGGGATCGTGTTGCTGGCCTTCGCCTCTCTG---CTCAGCTGC

---CTGCTCCCCTCC---------GGCCAGGCCAAGATCTACAGTCGCTGTGAGCTGACCAGAACGTTACGGAATTTCGGCCTCGAGGGCTACCGGGGCTACAGCCTGGCTGACTGGGTCTGTCTTGCTTACTACACAAGTGGCTTCAATACAGCTGCCGTGGACCATGAAGCT---GAT

GGAAGCACCAACAATGGCATCTTCCAGGTCAACAGCCGGAAGTGGTGCCAAAAT------CTCGAC------CCAGAGGCCCCCAACCTGTGCCAGATGTAC------------TGTTCCGACTTGTTGAATCCTAACCTCAAGAATGCTGTTATCTGTGCCATG---------------

---AAGATA---------ACTCAAGAGCCC---CGGGGCATGGGCAGCTGGGAGGCCTGGAGGCATCACTGCCAGGGCAAGGAC---CTCAGAGACTGGGTGGAT------GGATGTGACTTC---------------------------------------------------------

---

>Rabbit_Spaca3

------------------------------------------------------------------------------------------ATGGACGCC---AGGAGCTGGGCAGCCAGAAGGTGGCTGTGCCCACCTGGGATCGCGATGCTGGCCCTCCTCTCTCTG---CTTGGCTGC

---CAGCTCCCCTCC---------AGCGAGGCCAAGGTCTACAGCCGCTGCGAGCTGGCCAAAGTGCTGCACGACTTCGGTCTTAATGGATTCCGGGGCTACGGCCTGTCTGACTGGATCTGCCTTGCTTACTTCACCAGTGGCTTCAACACAGCCGCTGTGGACCATGAGGCT---GAT

GGAAGCACCAACAACGGCATCTTCCAGATCAACAGCCGCAGGTGGTGCAAAGAC------CTCTCC------AAGAAGGCCCTCAACTTGTGCCGGTTATAC------------TGCTCTGATTTGCTGAACCCAAACCTCAAAGACACTGTTGTTTGTGCCATG---------------

---AAGATA---------GCCCAAGAGCCC---CTAGGTCTGGGCTACTGGGAGGCCTGGAGGCATCACTGCCAGGGCAAGGAC---CTCAGCGACTGGGTGGAC------GGCTGTGAGTTC---------------------------------------------------------

---

>Guinea_pig_Spaca3

------------------------------------------------------------------------------------------ATGGAAACCAGAAGGAGCTGGGCTCTCAGAAGTCGACTGTGCTCACCTGTGATGACACTGCTGGTCTTTGCCTTTCTG---CTCAGCTGT

---CTTCTCAACTCC---------AACGAGGCCAAGGTCTACAGTCGCTGTGAGCTGGCCAGAGTGCTTCGAGACTTCGGCCTGGATGGCTATCGGGGATATAGCCTGGCTGACTGGGTCTGCCTTGCTTACTTCACAAGTGGCTTCAATACGGGTGCTGTGGACCATGAGGCA---GAT

GGAAGCACCAACAACGGCATTTTCCAGATCAACAGCCGGAGGTGGTGCAAAAAT------CTCACC------CCGAACTCCCACAACCAGTGCCGGGTGTAC------------TGCTCTGACTTGCTGAGCCCTGACCTCAAGGATACCGTTGTCTGTGCCATG---------------

---AAGATT---------GCTCAAGAGCCC---CAGGGTCTGGGCTACTGGGAGACCTGGAGGCGTCACTGCCAGGGCAAGGAC---CTGAGCGACTGGGTGGAT------GGCTGTGACTTC---------------------------------------------------------

---

>Elephant_Spaca3

------------------------------------------------------------------------------------------ATGAAAGCC---AGGAACTGGGCTCCCAGAAGGTGGCTGTGCCCGAATGGGAGCATGATGCTCGCCTTCGTCTCTCTG---CTCAGTTGC

---TTGCTGGCCACC---------AGTGAGGCCAAAGTCTACAGCCGCTGTGAGCTGGCCCGATTGCTACAGGAATTCGGTCTGGATGGATACCGTGGATACAGCCTGGCTGACTGGATCTGCCTTGCTTACTTCACAAGTGGCTTTAACACAGCTGCTGTGGACCATGAGGCA---GAT

GGAAGCACCAACAATGGCATCTTCCAGATCAGCAGCCGGAAGTGGTGCAACAAC------TTCGAT------TCGAAGGCCCCCAACCTGTGCCGGATGTAC------------TGTACTGACTTGTTGAAGCCTAACCTCAGGGAGACAGTTATCTGTGCAATG---------------

---AAGATA---------GCCCAAGGGCCC---CGTGGTCTGGCCACCTGGGAGACCTGGAGGCATCACTGCCAAGGCAAAGAC---CTCAGCGACTGGGTGGAT------GGCTGTGAC------------------------------------------------------------

---

>Hyrax_Spaca3

------------------------------------------------------------------------------------------ATGGAAGCC---AGGAACTGGGCTCCCAGAAGGTGGCTGCGCCCGACTGGGAGCATGATGCTGGCATTCGTCTCTCTG---CTCAGTTGC

---TTGCCGGCCGCC---------AGCGAGGCCAAGGTCTACAGCCGCTGTGAGCTGGCCAGGTTGCTACAGGAATTTGGTCTGGATGGATACCGTGGATACAGCCTGGCTGACTGGATCTGCCTTGCTTACTTCACAAGTGGCTTCAACACAGCTGCTGTGGACCACGAGGCA---GAC

GGAAGCACCAACAACGGCATCTTCCAGATCAACAGCCGCAAGTGGTGCAACAAC------TTCGAT------CTGAAGGCTCCCAACCTGTGCCGGATGTAC------------TGCACTGATTTGTTGAATTCTAACCTCAAGGATACAGTTATCTGTGCCATG---------------

---AAGATA---------ACCCAAGGGCCC---CAGGGTCTGGCTGCCTGGGAGGCCTGGAGGCATCACTGCCAAGGCGAGGAC---CTCAGCGACTGGGTGGAT------GGCTGTGACTTG---------------------------------------------------------

---

>Mouse_Spaca3

------------------------------------------------------------------------------------------ATGGAAGCT---AGGAGCCGGGCTCCCAGAAGACAGCTGTGCCCGCCTGGGATCACTTGGCTGGCCCTGGCCTATCTG---CTCAGCTGC

---CTGCTTGCCTCC---------AGCAAGGCCAAGGTCTTCAGTCGCTGTGAGCTGGCCAAAGAGATGCATGACTTCGGTCTGGATGGCTACCGGGGTTATAACCTGGCTGACTGGGTCTGCCTTGCTTACTACACAAGTGGCTTCAACACAAATGCTGTGGATCATGAAGCT---GAT

GGAAGCACCAACAATGGCATCTTCCAGATCAGCAGCCGGAGGTGGTGCAGAACC------CTCGCC------TCGAATGGCCCCAATCTTTGCAGGATATAC------------TGCACTGATTTGTTGAACAATGATCTCAAAGATTCTATCGTCTGTGCCATG---------------

---AAGATA---------GTTCAAGAACCC---CTGGGTCTGGGCTATTGGGAAGCCTGGAGGCACCACTGCCAGGGCAGGGAC---CTCAGTGACTGGGTGGAT------GGCTGTGACTTC---------------------------------------------------------

---

>Rat_Spaca3

------------------------------------------------------------------------------------------ATGGAAGCT---AGGAGCCGGGCTCCCAGAAGACAGCCGTGCCCGCCTGGGATCACTTGGCTGGCCCTGGCCTATCTG---CTCAGCTGC

---CTGCTTGCCTCC---------AGCAAGGCCAAGGTCTTCAGTCGCTGTGAGCTGGCCAAAGTGCTGCATGACTTCGGTCTGGAAGGCTACAGGGGATATAACCTGGCTGACTGGATCTGCCTTGCGTACTACACAAGCGGCTTCAATACGGATGCTGTGGATCACGAAGCT---GAC

GGAAGCACCAACAACGGCATCTTCCAGATCAGCAGCCGGAAGTGGTGCAAAAAC------CTCGCC------CCGAACGGCCCCAATCTTTGCAGGATATAC------------TGCACGGATTTGTTGAGCAATGATCTCAAAGATTCTGTCGCCTGTGTCATG---------------

---AAGATA---------GCGCAAGAGCCC---CAGGGCCTGGGCTACTGGGAATCCTGGAAGCACCATTGTCAGGGCAGGGAC---CTCAGTGACTGGGTGGAT------GGCTGTGACTTC---------------------------------------------------------

---

>Lizard_LyzI

------------------------------------------------------------------------------------------ATGAGGTCT---------------------------------------------------CTCTGGTCACTCGTGCTC---CTTGCCTGC

---CTCCTGGTCACA---------GGACAAGGAGAGTACCTCTCACGATGTGAGGTGGCCCAGCAGCTCCAGCAATTGGGGATGGATGGCTATGCTGGCTACAGCCTGGCCAATTGGGTCTGCACGGCATTCCACGAGAGCAGCTTCAACACTCAGGCCATGCACTATGACAGT---GAT

GGTAGCATTGACTTCGGCATCTTCCAGATCAACAGTCGCTACTGGTGCCAGTAT------GGCAAC------GAGAAAAGCTCCAACGCCTGCGGGATTCAA------------TGCTCTGAGCTGCTGACAAACAACCTCGCTGTAGACGCGGCTTGTGCTAAG---------------

---ATTGTG---------GTCTCAAATTCGTGGAATGGAATGGGTGCCTGGGTGGCATGGAGGTTGCATTGCCAAGGCCAAGAC---CTTTCTAGTTATGTTGAA------GGATGTGGTGTG---------------------------------------------------------

---

>Platypus_Lyzl6

------------------------------------------------------------------------------------------------------------------------------------------------AGGACCTTGGTGGTCCTCCTGTTG---GCCAGCAGC

---CTCTTGGGGGAG---------GCCCAGAGTGAGATCCTTGCCCGCTGTGAGCTGGCCATGCAGCTTCAAGAAGGAGGCCTGGGGGGGTTTAAGGGCTACAGCCTCGCTGACTGGATATGCCTGGCCTACCAGGAGAGCAAGTTTGACACGATGCTGATCAGCACCAACTCA---GAT

GGCAGCACGGACTATGGCCTCTTCCAGATCAACAGCCACGTCTGGTGCTCTGAC------CACCTC------AGCCATTCGCAGAACCGCTGCAAGGTCTCCTGCATCGGTACCTGTTGGGAGATACTGAAT---AACATCGTTGCCCACATCCTCTGTGCCAAG---------------

---AAGATT---------GTGTCTGAGCAC---TCTGGGCTCAAATACTGGTCTTCCTGGACGGAGCACTGTGAGGGCCGGGAC---CTCTCCCACTGGGTGGCC------GGATGTGACCTG---------------------------------------------------------

---

>Human_Spaca5

------------------------------------------------------------------------------------------ATGAAGGCC---------------------------------------------TGGGGCACTGTGGTAGTGACCTTG---GCCACGCTG

---ATGGTTGTCACT---------GTGGATGCCAAGATCTATGAACGCTGCGAGCTGGCGGCAAGACTGGAGAGAGCAGGGCTGAACGGCTACAAGGGCTACGGCGTTGGAGACTGGCTGTGCATGGCTCATTATGAGAGTGGCTTTGACACCGCCTTCGTGGACCACAATCCT---GAT

GGCAGCAGTGAATATGGCATTTTCCAACTGAATTCTGCCTGGTGGTGTGACAAT------GGCATT------ACACCCACCAAGAACCTCTGCCACATGGAT------------TGTCATGACCTGCTCAATCGCCATATTCTGGATGACATCAGGTGTGCCAAG---------------

---CAGATT---------GTGTCCTCACAG---AATGGGCTTTCTGCCTGGACTTCTTGGAGGCTACACTGTTCTGGCCATGAT---TTATCTGAATGGCTCAAG------GGGTGTGATATGCATGTGAAAATTGATCCA---AAAATTCATCCA------------------------

---

>Human_Spaca5B

------------------------------------------------------------------------------------------ATGAAGGCC---------------------------------------------TGGGGCACTGTGGTAGTGACCTTG---GCCACGCTG

---ATGGTTGTCACT---------GTGGATGCCAAGATCTATGAACGCTGCGAGCTGGCGGCAAGACTGGAGAGAGCAGGGCTGAACGGCTACAAGGGCTACGGCGTTGGAGACTGGCTGTGCATGGCTCATTATGAGAGTGGCTTTGACACCGCCTTCGTGGACCACAATCCT---GAT

GGCAGCAGTGAATATGGCATTTTCCAACTGAATTCTGCCTGGTGGTGTGACAAT------GGCATT------ACACCCACCAAGAACCTCTGCCACATGGAT------------TGTCATGACCTGCTCAATCGCCATATTCTGGATGACATCAGGTGTGCCAAG---------------

---CAGATT---------GTGTCCTCACAG---AATGGGCTTTCTGCCTGGACTTCTTGGAGGCTACACTGTTCTGGCCATGAT---TTATCTGAATGGCTCAAG------GGGTGTGATATGCATGTGAAAATTGATCCA---AAAATTCATCCA------------------------

---

>Chimpanzee_Spaca5

------------------------------------------------------------------------------------------ATGAAGGCC---------------------------------------------TGGGGCACTGTGGTAGTGACCTTG---GCCACGCTG

---ATGGTTGTCACT---------GTGGATGCCAAGATCTATGAACGCTGCGAGCTGGCGGCAAGACTGGAGAGAGCAGGGCTGAACGGCTACAAGGGCTATGGCGTTGGAGACTGGCTGTGCATGGCTCATTATGAGAGTGGCTTTGACACCGCCTTCGTGGACCACAATCCT---GAT

GGCAGCAGTGAATATGGCATTTTCCAACTGAATTCTGCCTGGTGGTGTGACAAT------GGCATT------ACACCCACCAAGAACCTCTGCCACATGGAT------------TGTCATGACCTGCTCAATCGCCATATTCTGGATGACATCAGGTGTGCCAAG---------------

---CAGATT---------GTGTCCTCACAG---AATGGGCTTTCTGCCTGGACTTCTTGGAGGCTACACTGTTCTGGCCATGAT---TTATCTGAATGGCTCAAG------GGGTGTGATATGCATGTGAAAATTGATCCA---AAAATTCATCCA------------------------

---

>Gorilla_Spaca5

------------------------------------------------------------------------------------------ATGAAGGCC---------------------------------------------TGGGGCACTGTGGTAGTGACCTTG---GCCACGCTG

---ATGGTTGTCACT---------GTGGATGCCAAGATCTATGAACGCTGCGAGCTGGCGGCAAGACTGGAGAGAGCAGGGCTGAACGGCTACAAGGGCTACGGCGTTGGAGACTGGCTGTGCATGGCTCATTATGAGAGTGGCTTTGACACCGCCTTCGTGGACCACAATCCT---GAT

GGCAGCAGTGAATATGGCATTTTCCAACTGAATTCTGCCTGGTGGTGTGACAAT------GGCATT------ACACCCACCAAGAACCTCTGCCATATGGAT------------TGTCATGACCTGCTCAATCGCCATATTCTGGATGACATCAGGTGTGCTAAG---------------

---CAGATT---------GTGTCCTCACAG---AATGGGCTGTCTGCCTGGACTTCTTGGAGGCTACACTGTTCTGGCCATGAT---TTATCTGAATGGCTCAAG------GGGTGTGATATGCATGTGAAAATTGATCCA---AAAATTCATCCA------------------------

---

>Orangutan_Spaca5

------------------------------------------------------------------------------------------ATGAAGGCC---------------------------------------------TGGGGCACTGTGGTAGTGACCTTG---GCCACGCTG

---ATGGTTGTCACT---------GTGGATGCCAAGATCTATGAACGCTGCGAGCTGGCAGCAAGACTGGAGAGAGCAGGGCTGAACGGCTACAAGGGCTACGGCGTTGGAGACTGGCTGTGCATGGCTCATTATGAGAGTGGCTTTGACACCGCCTTCGTGGACCACAATCCT---GAT

GGCAGCAGTGAATATGGCATTTTCCAACTGAATTCTGCCTGGTGGTGTGACAAT------GGCATT------ACACCCACCAAGAACCTCTGCCACATGGAT------------TGTCATGACCTGCTCAATCGCCATATTCTGGATGACATCATGTGTGCCAAG---------------

---CAGATT---------GTGTCCTCACAG---AATGGGCTGTCTGCCTGGACTTCTTGGAGGCTACACTGTTCTGGCCATGAT---TTATCTGAATGGCTCAAG------GGGTGTGATATGCACGTGAAAATTGATCCA---AAAATTCATCCA------------------------

---

>Macaque_Spaca5

------------------------------------------------------------------------------------------ATGCAGGCC---------------------------------------------TGGGGCACTGTGGTAGTGACCTTG---GCCACACTG

---ATGGTTGTCACT---------GTGGATGCCAAGATCTATGAACGCTGCGAGCTGGCGGCAAGACTGGAGAGAGCAGGGCTGAACGGCTACAAGGGCTACGGCATTGGAGACTGGCTGTGCATGGCTCATTATGAGAGTGGCTTTGACACCGCCTTCGTGGACCACAATCCT---GAT

GGCAGCAGTGAATATGGCATTTTCCAACTGAATTCTGCCTGGTGGTGTGACAAC------GGCATT------ACACCTACCAAGAACCTCTGCCACATGGAT------------TGTCATGACCTGCTCAATCGCCATATTCTGGATGACATCATGTGTGCCAAG---------------

---CAGATT---------GTGTCCTCACAG---AATGGGCTGTCTGCCTGGACTTCTTGGAGGCGGCACTGTTCTGGCCATGAT---TTATCTGAATGGCTCAAG------GGGTGTGATATGAACGTGAAAATTGATCCG---AAAATTCATCCA------------------------

---

>Baboon_Spaca5

------------------------------------------------------------------------------------------ATGCAGGCC---------------------------------------------TGGGGCACTGTGGTAGTGACCTTG---GCCACACTG

---ATGGTTGTCACT---------GTGGATGCCAAGATCTATGAACGCTGCGAGCTGGCGGCAAGACTGGAGAGAGCAGGGCTGAACGGCTACAAGGGCTACGGCATTGGAGACTGGCTGTGCATGGCTCATTATGAGAGTGGCTTTGACACCGCCTTCGTGGACCACAATCCT---GAT

GGCAGCAGTGAATATGGCATTTTCCAACTGAATTCTGCCTGGTGGTGTGACAAT------GGCATT------ACACCCACCAAGAACCTCTGCCACATGGAT------------TGTCATGACCTGCTCAATCGCCATATTCTGGATGACATCATGTGTGCCAAG---------------

---CAGATT---------GTGTCCTCACAG---AATGGGCTGTCTGCCTGGACTTCTTGGAGGCGGCACTGTTCTGGCCATGAT---TTATCTGAATGGCTCAAG------GGGTGTGATATGAACGTGAAAATTGATCCG---AAAATTCATCCA------------------------

---

>Marmoset_Spaca5

------------------------------------------------------------------------------------------ATGCAGGCC---------------------------------------------TGGGGCACTGTGGTAGTGACCTTG---GCCATACTG

---ATGGTTGTCACT---------GTGGATGCCAAGATTTATGAACGCTGCGAGCTGGCTGCAAGACTGGAGAGGGCAGGGCTGAACGGCTACAAAGGCTACAGTGTTGGAGACTGGCTGTGCATGGCTCACCATGAGAGTGGTTTTGACACCACCTTCGTGGACCACAATCCT---GAT

GGCAGCAGTGAATATGGCATTTTCCAACTGAATTCTGCCTGGTGGTGTGACAAT------GGCATT------ACACCCACCAAGAACCTCTGCCACATGGAT------------TGTCATGACCTGCTCAATCGCCATATCCTAGATGACATCATGTGTGCCAAG---------------

---AAGATC---------GTGTCCTCACAG---AACGGGCTGTCTGCCTGGACTTCTTGGAGGCGGCACTGTTCTGGCCATGAT---TTATCTCAATGGCTCAAG------GGGTGTGCTATGCAAGTGAAAACTGACCCA---AAAAATTATCCA------------------------

---

>Mouse_lemur_Spaca5

------------------------------------------------------------------------------------------ATGCAGGCC---------------------------------------------TGGGGCACTGTGGTAGTGACCTTG---GCCGCGCTG

---ATGGTTGCCACT---------GTGGATGCCAAGATCTATGAACGCTGCGAGCTGGCAACCAAGCTGGAGAAGGCAGGCCTTAATGGCTACAAGGGCTACAGCGTTGGAGACTGGCTGTGCATGGCTCACTATGAGAGTGGCTTTGACACCTCCTTCGTAGACCACAATCCT---GAT

GGCAGCAGCGAATATGGCATTTTCCAGCTGAACTCTGCCTGGTGGTGTGACAAT------GGTGTC------ACACCCACCCAGAACCTTTGTCACATGGAT------------TGTCGTGACCTGCTCAATCGCCATATTCTGGATGACATCTTGTGTGCCAAG---------------

---CGGGTT---------GTGTCGTCACAT---AACAGTATGACTGCCTGGGATTCTTGGAGCCGGCACTGTTCTGGCCATGAT---TTATCTGAATGGCTCAAA------GGGTGTGATGTGTATGTGAAAACTGACCCG---AAAATTAATTCA------------------------

---

>Treeshrew_Spaca5

------------------------------------------------------------------------------------------ATGCAGTCC---------------------------------------------TGGGGCACTGTGGTAGTGACCTTG---GCCATAGTG

---ATGGTTGCCACT---------GTGGATGCCAAGATCTACGAGCGCTGTGAGCTGGCAAGAAAGCTGGAGAAAGCAGGCCTCAATGGCTACAGGGGCTACACCATTGGAGACTGGCTATGCATGGCACACTATGAGAGTGGCTTTGATACCTCCTTCGTGAATCACAATCCT---GAC

GGCAGCAGTGAATATGGCATTTTCCAGCTGAACTCTGCCTGGTGGTGTGATAAT------GGTGTC------ACACCTTCCCAGAATCTCTGCCACATGGAT------------TGTCGTGACCTGCTCAACCGCCATATTCTGGATGACATCTTGTGTGCTAAG---------------

---CGGATT---------GTGTCCTCACAT---AACAGTATGACTGCCTGGAACTCGTGGACCAAGCACTGTTCTGGCCATGAT---TTAAGTGGATGGATCAAG------GGGTGTGATATGAATGTGAAAACTGACCCAAAGAAAATTAATTCA------------------------

---

>Rabbit_Spaca5

------------------------------------------------------------------------------------------ATGCAGGCC---------------------------------------------TGGAGCTTTGTGGTGTTAACCTTG---GCCACACTG

---ATGATTTCTACT---------GTAGATGCCAAGATCTATGAACGCTGTGAGCTGGGAATGAAGCTGCAGAAAGCAGGCCTCGATGGCTACAGGGGCTACAGCATTGGAGACTGGCTGTGCATGGCACACTATGAGAGTGGTTATGACACCTCTTTCGTGGACCACAATCCT---GAT

GGTAGCAGTGAATATGGGATTTTCCAGCTGAATTCTGCCTGGTGGTGTAACAAC------GGCATC------ACACCCACCGAGAACCTCTGCCACATGGAA------------TGTCGTGATCTACTCAACCGCCACATTCTAGATGACATCTTGTGTGCCAAG---------------

---CGAGTT---------GTATCCACACAG---AACAGTATGAGTGCCTGGGATTCCTGGACCCAGCACTGTTCTGGCCATGAT---TTATCTGAATGGATCAAG------GGGTGTGATGTGCATTTAAAGACTGACTCAAGGAAAATGCATCCA------------------------

---

>Dog_Spaca5

------------------------------------------------------------------------------------------ATGCAGGCC---------------------------------------------TGGGTCACCATGGTGGTGACCCTG---GCCACGCTG

---ACAGTTGCCCCT---------GTGGATGCCAAAATCTATGAACGCTGTGAACTGGCAATGAAGCTGGAGAAGGCGGGCCTCAACGGCTTCAAGGGCTACAGCATTGAAGACTGGCTGTGCATGGCACACTATGAGAGTGGCTTTGACACCTCCTTCGTGGACCACAATCCC---GAC

GGCAGCAGTGAATATGGCATTTTCCAGCTGAACTCCGCCTGGTGGTGTAACAAT------GGTGTT------ACACCCACCCAGAACCTCTGTCACATGGAG------------TGTCGTGACCTGCTCAACCGCCATATTCTGGATGATATCTTGTGTGCCAGG---------------

---CAGGTG---------GCATCATCAAAG---AATGGTATGACTGCCTGGGATTCTTGGACCCGGCACTGTTCTGGCCATTAT---TTATCTGAATGGCTCAAG------GGATGCAATATGCATGCCAAATCTGACTCAAAGAAAATTAATTCA------------------------

---

>Elephant_Spaca5

------------------------------------------------------------------------------------------ATGGAGGCT---------------------------------------------TGGGGCACTGTAGTGGCAATCCTG---GCCACACTG

---ATGGTTGCCACT---------GTGGATGCCAAAATCTATGGACGCTGTGAGCTGGCAATGAAGCTGGACAAGGCGGGCCTCAATGGCTTCAAGGGCTACACCATTGGAGACTGGATGTGCATGGCACACTATGAGAGTGGCTTTGACACCTCCTTCGTGGACCACAATCCC---GAC

GGTAGCAGTGAATACGGCATTTTCCAGCTGAACTCTGCTTGGTGGTGTGACAAT------GGCATT------ACACCTACCTGGAACCTCTGCCACATGGAT------------TGCCGTGACCTGCTCAACCGCCACCTTCTAGATGACATCCTGTGTGCCAAG---------------

---CAGGTC---------GTGTCCTCAAAA---AAGGGTATGACTGCCTGGGATTCTTGGACCAAGCACTGTTATGGCCATGAT---TTATCTGAATGGCTCAAG------GGGTGTAATATGCGCGTGAAAATTGACTCAAAGAAAATGAATTCA------------------------

---

>Tenrec_Spaca5

------------------------------------------------------------------------------------------ATGAAGGTC---------------------------------------------TGGGGTGCTGTGGTGGCAATCTTG---GCCACACTG

---ATGGTTGTCACT---------TTGGACAGCAAGATCTATAGACGCTGTGAGCTGGCAAGGAAGCTGGACAAGGCGGGCCTCAACGGCTACAAGGGCTACACCATTGGAGACTGGTTATGCCTGGCACACTATGAGAGTGGCTTTGACACCTCCTTCGTGGACCACAATCCT---GAC

GGTAGCAGCGAATATGGCATTTTCCAGCTGAACTCTGCTTGGTGGTGTAACAAT------GCCGTT------ACACCCACCTGGAACCTCTGCCACATGGAT------------TGCCATGACCTACTCAACCGCCATCTTCTGGATGACATCTTGTGTGCTAAG---------------

---AAAGTC---------GTGTCCTCAAAA---AGGGGCATGACTTCCTGGGATTCTTGGACCGAGCACTGTGATGGCCACGAT---TTATCTCAATGGCTCAAG------GGGTGTAAAATAAGTGTGAGAATTGACTCA---AAAACAAATTCA------------------------

---

>Pig_Spaca5

------------------------------------------------------------------------------------------ATGAAAGCC---------------------------------------------TGGGGCTCTGTGGTGGTGACCCTC---ACCATGCTA

---ATGGCTGCCACT---------GTGGATGCCAAGATTTATGAACGCTGTGAGCTGGCAACGAAGCTGGAGAAGGCGGGCCTCGATGGCTTCAAGGGCTACACCACTGGAGACTGGCTGTGCATGGCGCACTATGAGAGTGGCTTTGACACCTCCTTTGTGAACCACAATCCT---GAT

GGCAGCAGTGAATATGGCATTTTCCAACTGAACTCCGCCTGGTGGTGTGACAAT------GGTGTT------ACACCCACCCAGAACCTCTGTCACATGGAG------------TGTCATGAACTGCTCAACCGCCATATTCTTGATGACATCATGTGTGCCCAG---------------

---CAGGTA---------GTGTCCTCACAG---GAAGGTATGAATGCCTGGGATTCTTGGAGCCAGCACTGTTCTGGCCATGAC---TTATCTGAATGGCTCAAG------GGGTGTCATTTGAATGTAAAACCTGACCCCAAAAGAATTCATTCA------------------------

---

>Mouse_Spaca5

------------------------------------------------------------------------------------------ATGAAGGTC---------------------------------------------TGCAGCATTGTGGTAGTGATCTTG---GCCGTATTG

---TTGATTGCCAAA---------TTGGATGCCAAGATTTATGAACGCTGTGAGCTGGCAAAGAAGCTGGAGGAGGCTGGCCTCGATGGCTTCAAAGGCTATACTGTTGGAGACTGGCTGTGTGTGGCACACTATGAGAGTGGCTTTGACACCTCTTTTGTGGACCACAATCCA---GAT

GGCAGCAGTGAATATGGCATTTTCCAGCTGAACTCTGCCTGGTGGTGTAACAAT------GGCATC------ACACCCACTCAGAACCTCTGCAACATCGAT------------TGTAATGACCTGCTCAACCGCCATATTCTGGATGATATCATATGTGCCAAG---------------

---AGGGTT---------GCATCCTCACAT---AAGAGTATGAAGGCCTGGGATTCCTGGACCCAGCACTGTGCCGGTCATGAT---TTATCAGAATGGCTAAAG------GGGTGTTCTGTGCGTCTGAAAACTGACTCAAGCTATAATAACTGG------------------------

---

>Rat_Spaca5

------------------------------------------------------------------------------------------ATGCAGTTC---------------------------------------------TGCAGTACTGTGGTGGTGATCTTG---GCCACACTG

---TTGCTTGCCACA---------GTGGATGCCAAGATTTATGAACGCTGTGAGCTGGCAAGGAAGCTGGAGAAAGCTGGCCTCAATGGCTTCAAAGGCTACACTGTTGGAGACTGGCTGTGTGTGGCACACTATGAAAGTGGTTTTGACACCTCTTTTGTGGACCACAATCCA---GAT

GGCAGCAGTGAATATGGCATTTTCCAGTTGAACTCTGCTTGGTGGTGTAATAAT------GGCATC------ACACCCACTCAGAACCTCTGCCACATGGAT------------TGTAATGACCTACTCAACCGCCATATTCTGGATGATATCATGTGTGCCAAG---------------

---AGGGTT---------GTATCCTCACAT---AAGAGTATGAAGGCCTGGGATTCCTGGACCCGGCACTGTGCTGGTCATGAT---TTATCTGAATGGCTCAAG------GGGTGTGATGTGCCTCTGAAAGCTGACTCAAACTATGATGACTGG------------------------

---

>Cow_Spaca5

------------------------------------------------------------------------------------------ATGCAGGTC---------------------------------------------TCAGGCACTATTGTGGTGATCCTG------------

---ATGGCTGCCAAC---------GTGGAGGCCAAGATCTACGAACGCTGTGACCTGGCAAAGAAGCTGGAAGCAGCAGGCCTCAACGGCTTCAAGGGCTATACCATTGGAGACTGGCTGTGCATGGCACACTATGAGAGTGGCTTTGACACTTCCTTCGTGAACCACAATCCC---GAT

GGCAGCAGTGAATATGGCATTTTTCAGCTGAATTCCGCCTGGTGGTGTTACAAT------GGTGTT------ACACCCAGCGAGAACCTCTGCCACATGGAT------------TGTCATGAACTGCTCAACCGCCATATTCTGGATGACATTATGTGTGCCAAG---------------

---AAGGTA---------GTGTCCTCAGAG---AGTGGCATGAGTGCTTGGGATTCTTGGAACCAACACTGTTATGGCTATGAT---TTATCTGAATGGCTCAGG------GGATGTCATATGAATGCAAAACCTAACAAAAAAATTATTTCA---------------------------

---

>Guinea_pig_Spaca5

------------------------------------------------------------------------------------------ATGCAGTTC---------------------------------------------TCAAGCACAGTGATAATGATCATA---------CTA

---ATGGCTGCCAAC---------GTGGACGGCAAGATTTATGAACGCTGTGAGCTAGCAAAGAAGCTAGAGAAAGCAGGCCTCAACAACTATAAAGGCTACAGCATTGGAGACTGGTTGTGCATGGCACACTACGAGAGTGGCTTTGACACCTCCTTTGTGGACCACAATCCT---GAT

GGCAGCAGTGAGTATGGCATTTTCCAGCTGAACTCCGCCTGGTGGTGTGACAAT------GGCATC------ACACCCACCAAGAACCTTTGCCACATGGAC------------TGTAATGATCTGCTCAACCGCCATATTCTGGATGACATCATATGTGTCAAG---------------

---CAGGTT---------GTATCCTCAAAG---AACGGTATGAATGCCTGGGATTCATGGAGCCGGCATTGTTTTGACCATGAT---TTATCTGAATGGCTCAAG------GGATGTGATATGAAAGTAAAAACCCAACTTAGAGAAAATTATTCA------------------------

---

>Shrew_Spaca5

------------------------------------------------------------------------------------------ATGCAGGCT---------------------------------------------TGGGTCTCTGCTTTGGTGACCCTA---ACCATGATG

---ATGGTTCTGACT---------ACAGATACCAAGATCTATGATCGCTGTGAACTGGCACAAAAGCTGGCGAAAGCAGGCCTCAACAATTTCATGGGCTACAGCATTGGAGACTGGCTGTGCATGGCACACTACGAAAGTGGATTTGATACCTCTTTCTTGAATCACAATGTT---GAT

GGCAGCAGTGAATATGGCATTTTCCAGCTGAATTCTGCATGGTGGTGTGAAAAT------GGCATT------ACACCTACACATAACCTCTGTCACATAAGT------------TGTTATGATCTACGTAATCGCCATATTAAAGATGATATCTTGTGTGCAAAG---------------

---AAGATT---------GTGAAGTTACAT---AATGGGATGAGTGCCTGGGATACTTGGACACACCACTGTTCTGGTCATGAT---TTATCTCAATGGACCAAG------GGGTGTCATTTATACTTAAAACCTCACTATAGGAAAAATGACTGG------------------------

---

>Human_Lyzl4

------------------------------------------------------------------------------------------ATGAAGGCA---------------------------------------------------TCCGTGGTTCTCTCCCTC---CTTGGCTAC

---CTGGTGGTTCCA---------AGTGGTGCTTACATCTTGGGGCGTTGCACAGTGGCTAAGAAACTCCACGATGGAGGCCTGGATTATTTTGAGGGCTATAGCCTTGAGAACTGGGTGTGCCTGGCCTACTTCGAGAGCAAGTTCAACCCCATGGCCATCTACGAGAACACACGTGAG

GGCTACACTGGCTTTGGCCTCTTTCAGATGCGTGGCAGTGACTGGTGTGGCGAC---------------------CATGGCAGGAACCGCTGCCATATGTCA------------TGTTCCGCTTTACTGAATCCTAATTTAGAGAAGACAATTAAATGTGCCAAG---------------

---ACCATT---------GTAAAAGGAAAA---GAAGGGATGGGAGCATGGCCCACCTGGTCCCGGTACTGCCAGTACTCCGATACCCTGGCACGGTGGCTGGAT------GGTTGCAAGCTG---------------------------------------------------------

---

>Gorilla_Lyzl4

------------------------------------------------------------------------------------------ATGAAGGCA---------------------------------------------------TCCGTGGTTCTCTCCCTC---CTTGGCTAC

---CTGGTGGTTCCA---------AGTGGTGCTTACATCTTGGGGCGTTGCACAGTGGCTAAGAAGCTCCACGATGGAGGCCTGGATTATTTTGAGGGCTATAGCCTTGAGAACTGGGTGTGCCTGGCCTACTTCGAGAGCAAGTTCAACCCCATGGCCATCTACGAGAACACACGTGAG

GGCTACACTGGCTTCGGCCTCTTTCAGATGCGTGGCAGTGACTGGTGTGGCGAC---------------------CATGGCAGGAACCGCTGCCATATGTCA------------TGTTCCGCTTTACTGAATCCTAATTTAGAGAAGACAATTAAATGTGCCAAG---------------

---ACCATT---------GTAAAAGGAAAA---GAAGGGATGGGAGCATGGCCCACCTGGTCCCGGTACTGCCAGTACTCCGACACCCTGGCGCGGTGGCTGGAT------GGTTGCAAGCTG---------------------------------------------------------

---

>Chimp_Lyzl4

------------------------------------------------------------------------------------------ATGAAGGCA---------------------------------------------------TCTGTGGTTCTCTCCCTC---CTTGGCTAC

---CTGGTGGTTCCA---------AGTGGTGCTTACATCTTGGGGCGTTGCACAGTGGCTAAGAAACTCCACGATGGAGGCCTGGATTATTTTGAGGGCTATAGCCTTGAGAACTGGGTGTGCCTGGCCTACTTCGAGAGCAAGTTCAACCCCATGGCCATCTACGAGAACACACGTGAT

GGCTACACTGGCTTCGGCCTCTTTCAGATGCGTGGCAGTGACTGGTGTGGCGAC---------------------CATGGCAGGAACCGCTGCCATATGTCA------------TGTTCCGCTTTACTGAATCCTAATTTAGAGAAGACAATTAAATGTGCCAAG---------------

---ACCATT---------GTAAAAGGAAAA---GAAGGGATGGGAGCATGGCCCACCTGGTCCCGGTACTGCCAGTACTCCGATACCCTGGCACGGTGGCTGGAT------GGTTGCAAGCTG---------------------------------------------------------

---

>Macaque_Lyzl4

------------------------------------------------------------------------------------------ATGAAGGCA---------------------------------------------------TCCGTGGTTCTCTCCCTC---CTTGGCTAC

---CTGGTGGTTCCA---------AGTGGTGCTTACATCTTGGGGCGTTGCACAGTGGCTAAGAAACTCCACGATGGAGGCCTGGATTATTTTGAGGGCTATAGTCTTGAGAACTGGGTGTGCCTAGCCTACTTCGAGAGCAAGTTCAACCCCATGGCCATCTACGAGAACACACAAGAT

GGCTACATTGGCTTTGGCCTCTTTCAGATTCGTGGCAGTGACTGGTGTGGTGAC---------------------CATGGCAGGAACCGCTGCCACATGTCA------------TGTTCTGCTTTACTGAATCCTAATTTAGAGAAGACAATTAAATGTGCCAAG---------------

---ACCATT---------GTAAAAGGAAAA---CAAGGGATGGGAGCATGGCCCACCTGGTCCCGGAACTGCCAGTACTCCGACACCCTGGCGCGGTGGCTGGAT------GGTTGCAAGCTG---------------------------------------------------------

---

>Marmoset_Lyzl4

------------------------------------------------------------------------------------------ATGAAGGCA---------------------------------------------------GGCCTGGTTCTCTCCCTC---CTTGGCTGC

---CTGGTGGTTCCA---------ACTGGGGCTTACATCCTGGGGCGCTGCACAGTGGCCAAGAAGCTCCGTGATGGAGGCCTGGATTACTTCGAGGGCTATAGCCTTGAGAACTGGGTGTGCCTGGCCTACTTCGAGAGCAAGTTCAACCCCACGGCCGTCTACGAGAACACACGTGAT

GGCTACACTGGCTTCGGCCTCTTTCAGATTCGTGGCAGTGACTGGTGTGGCGAC---------------------CGTGGCAGGAACCGCTGCCACATGTCA------------TGTTCTGCTTTACTGAACCCTAATTTAAAGAAGACAATTGAATGTGCCAAG---------------

---ACCATC---------GTGAAAGGAAAG---AAAGGGATGGGAGCATGGCCCACCTGGTCCCGGTACTGCCGGTACTCTGACACCCTGGCGCGGTGGCTGGAC------GGTTGCAGGCTG---------------------------------------------------------

---

>Tarsir_Lyzl4

------------------------------------------------------------------------------------------ATGAAGGCA---------------------------------------------------TCTGTCCTTCTCTCCCTC---ATCAGCTGC

---CTGGTAATTCCG---------AGTGGTGCTCACATCTTGGGGCGTTGCACGGTGGCAAAAAAGCTCCTCAAAGGAGGCCTGGATTACTTTGAAGGCTACAGCCTTGCAAACTGGGTGTGCCTGGCTTACTTTGAGAGCAAGTTCAATCCCACAGCTGTCTACGAGAACACACAAGAT

GGCTCCACTGGCTTTGGCCTCTTTCAGATCCGCAACAGTGACTGGTGT---GAC---------------------CAAGGAAGGAACCGCTGCCACATGTCA------------TGTTCTGAGTTACTGAATCCGAATTTAAAGAAGACAATTGAATGTGCCAAG---------------

---ATAATT---------GTAAGAGGAAAA---GAAGGGATGGGAGCATGGCCCACCTGGTCCCAGTACTGCCAGTTCTCCGACACTCTGGCACAGTGGCTGGAT------GGTTGCAAGCTA---------------------------------------------------------

---

>Cow_Lyzl4

------------------------------------------------------------------------------------------ATGAAGGCG---------------------------------------------------TCTGTGGTGCTGTCTCTC---ATTGGGTAC

---CTGGTGGTTCCA---------AGTGACACTGCAGTTCTGGGGCGCTGCGTGGTGGCTAAGAAGCTCCATGAAGGAGGCCTGAGTGATTTTGAGGGCTACAGCCTTGAAAACTGGGTGTGCCTGGCTTATTTTGAGAGCAAGTTCAACCCTATGGCTGTCTATGAAAACTCGCGCAGT

GACTTCATTGGCTACGGCCTCTTTCAGATCCGCAACCATGACTGGTGT---GAC---------------------CACGGCAGGAACCGCTGCCATATGTCT------------TGCTCTGCTTTACTGAATCCAGATTTAAAGAAGACAATCGAATGTGCCAAG---------------

---ACAATT---------GTAAAAGGAAAA---AGAGGGATGGGAGCATGGCCCTCTTGGACTCTCAACTGCCAGCACTCTGACACTCTGGCACGGTGGCTGGAC------GGATGCAAGCTG---------------------------------------------------------

---

>Pig_Lyzl4

------------------------------------------------------------------------------------------ATGAAGGCG---------------------------------------------------TCTGTGGTGCTCTCCCTC---ATTGGGTAC

---TTGGTGGTTCCA---------AGTGGTGCTGCTGTCTTGGGGCGCTGTGTCGTGGCCAAGAAGCTCCACGAAGGAGGCCTGAGTGACTATGAGGGATATAGCCTTGAAAACTGGGTGTGCCTGGCTTATTTTGAGAGCAAGTTCAACCCCACGGCCGTCTATGATAACCTTCGTGGT

GACTACACCGGCTATGGCCTCTTTCAGATCCGCAATCATGACTGGTGT---GAC---------------------CATGGCAAGAACCGCTGCCATGTGTCC------------TGCTCTGCTTTACTGAATCCGAATTTAAAGGAGACAATTAAGTGTGCTAAG---------------

---AAAATT---------GTACAAGGAAAA---AGAGGGATGGGAGCATGGCCTTCCTGGACCCTGAACTGCCAGAACTCTGACACTCTGGCGCGGTGGTTGGAC------GGGTGCAAGCTG---------------------------------------------------------

---

>Elephant_Lyzl4

------------------------------------------------------------------------------------------ATGAAAGCA---------------------------------------------------TCTGTGGTTCTCTCCCTC---CTCAGTTAC

---CTGGTGGTGCCA---------AGTGACGCTCGGGTCTTAGGGCGCTGCAGGGTGGCTAAGAAGCTCCAGGAAGGAGGCCTGAGTGACTTTGATGGCTACAGCCTTGAAAACTGGGTGTGCCTGGCTTACTTTGAGAGCAAGTTCAACCCCTCAGCTGTCTATGACAACTTGCGTGGT

GACTACACTGGCTATGGCCTCTTCCAGATCCGGAACAATGACTGGTGT---GAC---------------------CACGGCAAGAACCGCTGCCACGTGTCA------------TGCTCTGCTCTACTGAATCCAGATTTAAAGAAGACAATTGAATGTGCCAAG---------------

---AAAATT---------GTACAAGGAAAA---GATGGGATGGGAGCATGGCCTTCCTGGACCCTGAACTGCCAGAACTCCGACACGCTGGCACGGTGGTTGGAT------GGATGCATTCTG---------------------------------------------------------

---

>Horse_Lyzl4

------------------------------------------------------------------------------------------ATGAAGGCT---------------------------------------------------TTCGTGGTTCTGTCCCTC---ATTGGGTAC

---CTGGTGGTTCCA---------AGTGGCGCTGCTGTCTTGGGGCGCTGTGTGGTGGCTAAGAAGCTCCATGAAGGAGGCCTGAGTAATTTTGAGGGCTACAGCCTTGAAAACTGGGTGTGCCTGGCTTACTTTGAGAGCAAGTTCAACCCCATGGCTGTCTATGAGAACACGCGTGAT

GGCTACACCGGCTTCGGCCTCTTTCAGATCCGCAACAATGTCTGGTGC---GAC---------------------AGAGGCAAGAACCGCTGCCACGTGTCC------------TGCTCCGCTTTACTGAATCCAAATTTACAGAAGACAATTGAATGTGCCAAG---------------

---AAAATT---------GTAAAGGAAAAA---GAAGGGATGAGAGCATGGCCCTCCTGGTCCCTGAACTGTCAGAACTCGGACACTCTGGCACGGTGGTTGGAC------GGATGCAAGCTG---------------------------------------------------------

---

>Rabbit_Lyzl4

------------------------------------------------------------------------------------------ATGAAAGCA---------------------------------------------------TCTGTGGTGTTCTCCCTT---CTTGGCTTC

---TTGGTAGCGCCA---------AGTGCTGCTTACGTTTTGGGCCGTTGCACAGTGGCTAAGAAACTCCGAGAAGGAGGCCTGGATTATTTTGAGGGCTACAGCCTTGAAAACTGGGTGTGTCTGGCTTATTTTGAGAGCAAGTTCAACCCCACGGCTGTCTATGAAAACGCACCTGAT

GGCTACGCAGGCTTCGGCCTCTTTCAGATCCGCAACGATGGCTGGTGT---GAC---------------------CACGGCAAGAACCTCTGCCGCATGTCA------------TGCTCTGAGATGCTGAATCCAAATTTAAAGAAGACAATTGAATGTGTCAAG---------------

---ATAATT---------GTAAAAGGAAAA---GAAGGGATGGGAGCATGGCCCACCTGGTCCCAGAACTGCCAGCTCTCCGACACTCTGGGGCGCTGGCTCGAC------GGGTGCAAGATG---------------------------------------------------------

---

>Dolphin_Lyzl4

------------------------------------------------------------------------------------------ATGAAGGCG---------------------------------------------------TCCATGGTGCCCTGCCTC---ATTGGGTAC

---CTGGTGGTTCCA---------AGTGGCGCTGCTGTCTTGGGGCGCGGTGTGGTGGCTAAAAATCTCCAC---GGAGGCCTGAGTGATTTTGAGGGCTATAGCCTCGAAAACTGGGTGTGCCTGACTTATTTTGAGAGCAAGTTCAACCCCACTGCTGCCTACGACAGCTTGCGCGGT

GACTACACCGGCTACGGCTTCTTTCAGATCTGCAACCGTGACCGGTGT---GAT---------------------CACGGCAAGAACTGCTGCCACGTGTCC------------TGCTCTGCTTCACTGAATCCAAATTTAAAGGAGACAATTGAATGTGCCAAG---------------

---AAAATT---------GTAAAAGGAAAA---AGAGGAATGGGAGCGTGGACCTCCTGGACCCTCAACTGCCAGGACTCTGACACTCTGGAGCGGTGGTTGGAC------GGATGCAAGCTG---------------------------------------------------------

---

>Mouse_lemur_Lyzl4

------------------------------------------------------------------------------------------ATGAAGGCG---------------------------------------------------TCTGTGGTTCTCTCCCTC---ATTGGCTGC

---CTGGTGTTTCCA---------AGCAGTGCTCGCATCATAGGGCGTTGCACAGTGGCTAAGAAGCTCCACCAAGGAGGCCTGACTTATTATGAGGGCTACAGCCTTGAAAACTGGGTGTGCCTGGCTTATTTTGAGAGCAAGTTCAACCCCACAGCCGTGTACGACAACGCACCTGGA

GGCTACACTGGCTACGGCCTCTTTCAGATCCGAAGCAATGACTGGTGT---GGC---------------------CATGGCAAGAGCCGCTGCCATACGGAA------------TGTTCCGCTTTACTGAACCAGAATTTGCAGAAGACAATTGAATGTGTCAAG---------------

---ATAATT---------GTAAAAGGGAAA---GATGGGATGGGATCATGGCCCACCTGGTCCCGGTTCTGCCAGTACTCCGACACCCTGGCGCGCTGGCTAGAC------GGTTGCAAGCTG---------------------------------------------------------

---

>Mouse_Lyzl4

------------------------------------------------------------------------------------------ATGCAGCTG---------------------------------------------------TACCTGGTGCTTCTCCTC---ATCAGCTAC

---TTGCTGACTCCG---------ATCGGTGCTTCCATCTTGGGGCGCTGCACAGTGGCTAAGATGCTCTATGATGGGGGCTTGAATTATTTTGAGGGCTACAGCCTTGAAAACTGGGTGTGCCTGGCTTATTTTGAGAGCAAGTTCAACCCCTCAGCTGTCTATGAGGACCCACAAGAT

GGCTCCACCGGTTTCGGCCTCTTTCAGATTCGTGACAATGAATGGTGC---GGC---------------------CACGGCAAGAACCTCTGCAGTGTGTCC------------TGCACTGCTTTACTGAATCCAAACTTAAAGGATACGATCCAGTGTGCCAAG---------------

---AAAATC---------GTGAAAGGAAAA---CATGGGATGGGAGCGTGGCCCATCTGGTCCAAGAACTGCCAGCTGTCTGACGTTCTGGACCGGTGGCTGGAC------GGCTGTGATCTG---------------------------------------------------------

---

>Rat_Lyzl4

------------------------------------------------------------------------------------------ATGCAGCTG---------------------------------------------------TACCTGGTGCTTCTCCTC---ATCAGCTAC

---TTGCTGACTCCG---------ATTGGTGCCTCCATTTTGGGGCGCTGCGTGGTGGCTAAGAAACTCTATGACGGGGGCTTAAATTATTTTGAGGGCTACAGCCTTGAAAACTGGGTGTGCCTGGCTTATTTTGAGAGCAAGTTCAACCCCTCGGCTGTCTATGAGAACTCACGAGAT

GGCTCCACCGGCTTCGGCCTCTTTCAGATCCGCGACAATGAATGGTGT---GAC---------------------CATGGCAAGAACCTCTGCAGTGTGTCC------------TGCACGGCTTTACTGAATCCCAACTTAAAGGATACAATCGAGTGTGCCAAG---------------

---AAAATC---------GTGAAAGGAAAA---CAAGGGATGGGAGCATGGCCCGTCTGGTCCAGGAACTGCCAGCTGTCGGACATTCTGGATCGGTGGCTGGAT------GGCTGTGAGCTG---------------------------------------------------------

---

>Guinea_pig_Lyzl4

------------------------------------------------------------------------------------------ATGCGGGCG---------------------------------------------------CTGGTGCTGCTCTTCCTC---CTGGGCTGC

---CTCGTGGTCCCC---------TGCGGCCCCCACATCCTAGGTCGCTGTGAGGTGGCTCGGAGGCTCCATGAAGGGGGCCTGAACTACTTCGAGGGTTACAGCCTCGCCAACTGGGTGTGCCTGGCTTATTTCGAGAGCAAGTTCAACCCCTCAGCAGTGTATGAGAACCGGCGGGAC

GGCTCCCTGGGCTACGGCCTCTTCCAGATCCACGACCGCAAGTGGTGT---GGG---------------------CCAGGCAGGAACCTCTGCAATGTGGAC------------TGCTCAGCTTTGCTGAATTCGAATTTAAAGGACACAATCAAATGTGTCAAG---------------

---AAAATC---------GTGCAAGGAAAA---AAAGGGCTGGGAGCATGGCTCAGCTGGTCCCTGCACTGCCATTACGCCGACACCCTGCCACGGTGGCTGGAC------GGCTGCAAGCTG---------------------------------------------------------

---

>Platypus_Lyzl4

------------------------------------------------------------------------------------------ATGAAGATC---------------------------------------------------TTCATGACTCTGTCCTTG---ATAAACTGC

---CTGATTGTGTCT---------AATGTAGCTAAGTTGTTGAGCCGCTGTGAGGTAGCCAATAAACTAAGCGATGAAGGTCTAGATGGCTATGATGGTTACAGCCTTGAAAACTGGGTCTGCCTGGCTTTCTTTGCGAGCAAGTTCAATACCACTGCGGAACACAAAGAA---GAAGAT

GGGAGCACTAGTTACGGAATCTTCCAAATCAACAGCAAAGAATGGTGCACCAAC------CACGAA------GAACACAGCAGGAACCGCTGTCACCATCTC------------TGCAGCGACTTCCAGAGTAGCGACTTGAGCAACAGCATTGAATGTGCCAAG---------------

---AAAATC---------ATTAAAGAAAAG---GAAGGAATGGGACATTGGTACGTCTGGAAAGAGAACTGCCAGAATAGTCACGGCCTGAAAAGATGGCTGTAC------CGCTGTGTAGAGATGAGC---------------------------------------------------

---

>Human_Lyzl6

------------------------------------------------------------------------------------------ATGACAAAG---------------------------------------------------GCGCTACTCATCTATTTG---GTCAGCAGC

---TTTCTTGCCCTA---------AATCAGGCCAGCCTCATCAGTCGCTGTGACTTGGCCCAGGTGCTGCAGCTGGAGGACTTGGATGGGTTTGAGGGTTACTCCCTGAGTGACTGGCTGTGCCTGGCTTTTGTGGAAAGCAAGTTCAACATATCAAAGATAAATGAAAATGCA---GAT

GGAAGCTTTGACTATGGCCTCTTCCAGATCAACAGCCACTACTGGTGCAACGAT------TATAAG------AGTTACTCGGAAAACCTTTGCCACGTAGAC------------TGTCAAGATCTGCTGAATCCCAACCTTCTTGCAGGCATCCACTGCGCAAAA---------------

---AGGATT---------GTGTCCGGAGCA---CGGGGGATGAACAACTGGGTAGAATGGAGGTTGCACTGTTCAGGCCGGCCA---CTCTTCTACTGGCTGACA------GGATGCCGCCTGAGA------------------------------------------------------

---

>Chimpanzee_Lyzl6

------------------------------------------------------------------------------------------ATGACAAAG---------------------------------------------------GCGCTACTCATCTATTTG---GTCAGCAGC

---TTTCTTGCCCTA---------AATCAGGCCAGCCTCATCAGTCGCTGTGACTTGGCCCAGGTGCTGCAGCTGGAGGACTTGGATGGGTTTGAGGGTTACTCCCTGAGTGACTGGCTGTGCCTGGCTTTTGTGGAAAGCAAGTTCAACATATCAAAGATAAATGAAAATGCA---GAT

GGAAGCTTTGACTATGGCCTCTTCCAGATCAACAGCCACTACTGGTGCAACGAT------TATAAG------AGTTACTCGGAAAACCTTTGCCACGTAGAC------------TGTCAAGATCTGCTGAATCCCAACCTTCTTGCAGGCATCCACTGCGCAAAA---------------

---AGGATT---------GTGTCCGGAGCA---CGGGGGATGAACAACTGGGTAGAATGGAGGTTGCACTGTTCAGGCCAGCCA---CTCTCCTACTGGCTGACA------GGATGCCGCCTGAGA------------------------------------------------------

---

>Gorilla_Lyzl6

------------------------------------------------------------------------------------------ATGACAAAG---------------------------------------------------GCGCTACTCATCTATTTG---GTCAGCAAC

---TTTCTTGCCCTA---------AATCAGGCCAGCCTCATCAGTCGCTGTGACTTGGCCCAGGTGCTGCAGCTGGAGGACTTGGATGGGTTTGAGGGTTACTCCCTGAGTGACTGGCTGTGCCTGGCTTTTGTGGAAAGCAAGTTCAACATATCAAAGATAAATGAAAATGCA---GAT

GGAAGCTTTGACTATGGCCTCTTCCAGATCAACAGCCACTACTGGTGCAACGAT------TATAAG------AGTTACTCGGAAAACCTTTGCCACGTAGAC------------TGTCAAGATCTGCTGAATCCCAACCTTCTTGCAGGTATCCACTGCGCAAAA---------------

---AGGATT---------GTGTCCGGAGCA---CGGGGGATGAACAACTGGGTAGAATGGAGGTTGCACTGTTCAGGCCAGCCA---CTCTCCTACTGGCTGACA------GGATGCNGCCTGAGA------------------------------------------------------

---

>Orangutan_Lyzl6

------------------------------------------------------------------------------------------ATGACAAAG---------------------------------------------------GTGCTACTCATCTACTTG---GTCAGCAGC

---TTTCTTGCCCTA---------AATCATGCCAGCCTCATCAGTCGCTGTGACTTGGCCCAGGTGCTGCAGCTGGAGGACTTGGATGGGTTTGAGGGTTACTCCCTGAGTGACTGGCTGTGCCTGGCTTTTGTGGAAAGCAAGTTCAACATATCAAAGATAAATGAAAATGCA---GAT

GGAAGTTTTGACTATGGCCTCTTCCAGATCAACAGCCACTACTGGTGCAACGAT------TATAAG------AGTTACTCGGAAAACCTTTGCCACGTAGAC------------TGTCAAGATCTGCTGAATCCCAACCTTCTTGCAGGCATCCACTGCGCAAAA---------------

---AGGATT---------GTGTCCGGAGCA---CGGGGGATGAACAACTGGGTAGAATGGAGGTTGCACTGTTCAGGCCGGCCA---CTCTCCTACTGGCTGACA------GGATGCCGCCTGAGA------------------------------------------------------

---

>Macaque_Lyzl6

------------------------------------------------------------------------------------------ATGACAAAG---------------------------------------------------GTGCTATTCATCTACTTG---GTCAGCAGC

---TTTCTTGCCCTA---------AATCAGGCCAGCCTCATCAGTCGCTGTGACTTGGCCCAGGTGCTGCAGCTGGAGGACTTGGATGGGTTTGAGGGTTACTCCCTGAGTGACTGGCTGTGCCTGGCTTTTGTGGAAAGCAAATTCAACATATCAAAGATAAATGAAAACGCA---GAT

GGAAGCTTTGACTATGGCCTCTTCCAGATCAACGGCCACTACTGGTGCAACGAT------TATAGG------AGTCACTCGGAAAACCTTTGCCAGGTGGAC------------TGTCAAGATCTGCTCAATCCCAACCTTCTCGCAGGCATCCACTGTGCAAAA---------------

---AGGATT---------GTGTCCGGAGCA---CGGGGGATGAACAACTGGGTAGAATGGAGGTTGCACTGTTCAGGCCGGCCA---CTCTCCTACTGGCTGACA------GGATGCCGACTGGGA------------------------------------------------------

---

>Baboon_Lyzl6

------------------------------------------------------------------------------------------ATGACAAAG---------------------------------------------------GTGCTACTCATCTACTTG---GTCAGCAGC

---TTTCTTGCCCTA---------AATCAGGCCAGCCTCATCAGTCGCTGTGACTTGGCCCAGGTGCTGCAGCTGGAGGACTTGGATGGGTTTGAGGGTTACTCCCTGAGTGACTGGCTGTGCCTGGCTTTTGTGGAAAGCAAATTCAACATATCAAAGATAAATGAAAACGCA---GAT

GGAAGCTTTGACTATGGCCTCTTCCAGATCAACGGCCACTACTGGTGCAACGAT------TATAGG------AGTCACTCGGAAAACCTTTGCCAGGTGGAC------------TGTCAAGATCTGCTCAATCCCAACCTTCTCGCAGGCATCCACTGTGCAAAA---------------

---AGGATT---------GTATCCGGAGCA---CGGGGGATGAACAACTGGGTAGAATGGAGGTTGCACTGTTCAGGCCGGCCA---CTCTCCTACTGGCTGACA------GGATGCCGACTG---------------------------------------------------------

---

>Marmoset_Lyzl6

------------------------------------------------------------------------------------------ATGACAAAG---------------------------------------------------TTGTTACTCATCTACTTG---GTCAGCAGC

---TTCCTTGCCCTA---------AATCAGGCCGGCCTCATCAGTCGCTGTGACTTGGCCCAGGTGCTGCAGCAGGAGGACCTGGACGGGTTTGAGGGCTACTCCCTGAGTCACTGGCTGTGCCTGGCTTTTGTGGAAAGCAAGTTCAACATATCAAAGATAAATGAAAATGCA---GAT

GGAAGCTTTGACTATGGCCTCTTCCAGATCAACAGCCACTACTGGTGCAATGAT------CACAAG------AGTCACTCAGAAAACCTTTGCCACGTGGAG------------TGTCAAGATCTGCTGAATCCCAACCTTCTCGCAGGCATCCATTGTGCAAAA---------------

---AGGATT---------GTATCTGGAGCA---GGGGGGATGAACAACTGGGCAAAATGGAGGTTATACTGTTCAGGCCGGCCC---CTCTCCTACTGGATGACA------GGATGCCACCTGGGA------------------------------------------------------

---

>Mouse_lemur_Lyzl6

------------------------------------------------------------------------------------------ATGACGAGG---------------------------------------------------GAGCTACTCATCCCTTTG---GTCAGCTGC

---CTCCTTGTCGTA---------AACGAGGCCAGCCTCATCCATCGCTGTGACTTGGCCAGGGTGCTGTACCAGCAAGACTTGAATGGGTTTGAGGGCTACTTCCTGAGTGACTGGCTGTGCCTGGCTTTTATAGAAAGCAACTTCAACATATCAAAGGTAAATGAAAATGCA---GAT

GGCAGCTTTGACTATGGCCTCTTCCAGATCAACAGCCACTACTGGTGCAACGAT------TACAAG------AGTCACTCGGAAAACCTTTGCCATGTGGAC------------TGTCAAGATCTGCTGAACCCCAACCTTCTCTCAGGCATTAACTGCGCAAAA---------------

---ATGATT---------GTGTCCAGATCC---GGGGGGATGAACAAATGGGTAGAATGGAGGTTGCACTGCGCAGGCCGGCCA---CTCTCCTACTGGTTGACA------GGATGCCACCTG---------------------------------------------------------

---

>Guinea_pig_Lyzl6

------------------------------------------------------------------------------------------ATGGCCAGG---------------------------------------------------GCGCTGCTCATTTCCTTG---GCCAGCTGC

---CTGCTTGTCGTA---------AACCAAGCCTTCATTGTTAGACGCTGCGACTTGGCCAAGGTTCTGCACGAGGAGCAGCTGGATGGGTTTGAGGGGTACTCCCTGAGTGACTGGTTGTGCCTGGCTTTTGTGGAAAGCAACTTCAACATATCAAAGGTAAATGAAAATGCA---GAT

GGCAGCTTTGACTACGGAATCTTCCAGATCAACAGTCACTACTGGTGCAATGAT------TACCAG------AGTCACTCGGAAAACTATTGCCACGTGGAC------------TGCCAAGATTTACTGAACCCCAATCTTCTCTCATCCATCAGCTGTGCAAAG---------------

---ATCATT---------GTGTCTGCATCA---GGGGGGATGAGGAACTGGGTGAGATGGAAGCTGCACTGTGCAGACCGGCCA---CACACCTACTGGATGACA------GGGTGTCATCTGGGA------------------------------------------------------

---

>Cow_Lyzl6

------------------------------------------------------------------------------------------ATGACAAGC---------------------------------------------------CCACTGCTCATCTCGTTG---GCCAGCTGC

---CTCGTTGCTGTG---------AATCAAGCCAGCCTCATTGGCCGCTGTGACTTGGCCAAGGTGCTGCACCAGGAGGACTTGGATGGGTTTGAGGGCTACTCCCTGACTGACTGGCTGTGCCTGGCTTTCGTAGAGAGTGATTTCAACATAACAAAGGTAAATGAAAACACA---GAT

GGCAGCTTTGACTATGGCATCTTCCAGATCAACAGCCACTACTGGTGCAACGAT------TACCAG------AGTCACACGGAAAACAATTGCCAAGTGGAC------------TGTCAAGAACTGCTGAGCCCCAATCTTCTCGCAATTATCAACTGTGCAAAA---------------

---AAGATT---------GTGTCCGGAGCT---GGGGGCATGAAGAACTGGGTAAAATGGAGGTTGCACTGTGCTGGCCGGCCC---CTCTCCTACTGGATGACA------GGCTGTCACCTGGCA------------------------------------------------------

---

>Pig_Lyzl6

------------------------------------------------------------------------------------------ATGACCCGC---------------------------------------------------CCGCTGCTCATCTCCTTG---GTCAGCTGC

---CTGGTTGCTGTC---------AGTCAAGCCAGCCTCATCAACCGCTGTGACCTGGCCAAGGTGCTGCACCAGGAGGACCTGGATGGCTTTGAGGGCTACTCCCTGAGCGATTGGCTGTGCCTGGCTTTCGTGGAAAGTGAATTCAATATAACAAAGGTAAATGAAAATACA---GAC

GGCAGCTTTGACTATGGCATCTTCCAGATCAACAGCCACTACTGGTGCAACGAT------TACCGG------AGTCACACGGAGAACATTTGCCAAGTAGAA------------TGTGAAGAACTGCTGAGCCCCAATCTTCTCTCAATCATCAACTGTGCAAAA---------------

---AAGATT---------GTGTCTGGAGCA---GGAGGGATGAAGAACTGGGTAAAGTGGAGGTTGCACTGTGCAGGCCGGCCA---CTCTCCTACTGGATGACA------GGGTGCCACTTGGGA------------------------------------------------------

---

>Horse_Lyzl6

------------------------------------------------------------------------------------------ATGGCGAGG---------------------------------------------------GCGCTGCTCCTCTCCCTG---GTCAGCTGC

---CTCGCTGCCGTG---------AGTCAGGCCAGCCTCATCAGACGCTGTGACTTGGCCAAGGTGCTGCACGAGGAGGACTTGGATGGGTTTGAGGGCTACTCCCTGAGTGACTGGCTGTGCCTGGCTTTTGTGCAAAGCAACTTCAACATATCAAAGGTAAATGAAAATACA---GAT

GGCAGCTTTGACTATGGCATCTTCCAGATCAACAGCCACTACTGGTGCAACGAT------CACAAG------AGTCATTCAGAAAACCTTTGCGACGTGGAC------------TGTCAAGAGTTGCTGAGCCCCAATCTGCTCTCGACCATCAACTGTGCAAAA---------------

---AAGATC---------GTGTCCAGTGCA---GGGGGGATGAAGAACTGGGTAGGATGGAGGTTGCACTGTTCGGGCCGGCCG---CTCTCCTACTGGATGACA------GGGTGCCACCTGGGG------------------------------------------------------

---

>Alpaca_Lyzl6A

------------------------------------------------------------------------------------------ATGCCCAGC---------------------------------------------------CTGCTGCTCGTCTCCTTG---GCCAGCTGC

---CTGCTGGCCATA---------AAGCAGGCCAGCCCCATCAGCCGCTGTGACTTGGCCAAGGTGCTGCGCGAGGAGGACTTGGATGGGTTTGAGGGCTACTCCCTGAGTGACTGGCTGTGCCTGGCTTTCGTGGAGAGTGCCTTCAACATAACAAAGGTAGATGAAAATACC---GAT

GGCAGCTTTGACTATGGCATCTTCCAGATCAACAGCCACTACTGGTGCAACGAT------TACCAG------AGTCACACGGAAAACATTTGCCACATGGAC------------TGTCAAGAACTGCTGAGCCCCAACCTTCTCTCAATCATCAACTGTGCAAAA---------------

---AAGATT---------GTGTCCGGAGCA---GGGGGCATGAAGAACTGGGTAAAATGGAGGCTGCACTGTGCGGGCCGGCCA---CTCTCCTACTGGATGACA------GGATGCCACCTGGGA------------------------------------------------------

---

>Alpaca_Lyzl6B

------------------------------------------------------------------------------------------ATGCCCAGC---------------------------------------------------CTGCTACTCATCTCCTTG---GTCAGCTGC

---CTGGTGGCCATA---------AGTCAGGCCAGACCCATCAGCCGCTGTGACTTGGCCAAAGTGCCACACAAGGAGGACTTGGATGGGTCTGAGGGCCACTCTCTGAGTGACTGGCTGAGCCTGGCTTTTGTGGAAAGTGACTTCAACATAGCAAAGGTAAATGAAAATACA---TAT

GGCAGCTTTGACTACGGCATCTTCCAGATCAACAGCCACTACTGGTGCAACGAT------TACCAG------AGTCACACGGAAAACATTTGCCACGTGGAC------------TGTCAAGAACTGCTGAGCCCTAACCTTCTCTCAATCATCAACTGTGCAAAA---------------

---AAGATT---------GTGTCCGGAGCA---GGGGGCATGAAGAACTGGGTAAAATGCAGGCTGCACTGTGCAGGCCGGCCA---CTCTCCTGCTGGATGACA------GGGTGCCACATGGGA------------------------------------------------------

---

>Dog_Lyzl6A

------------------------------------------------------------------------------------------ATGACAAGG---------------------------------------------------GTGCTGTTCATGTCCTTG---GTCAGCTTC

---CTCATTGCCATA---------AATCAGGCCAGCCTCATCAATCGCTGCGACTTGGCCAGGGTGCTGCGCAAGGAGGACTTGGATGGGTTTGAGGGCTACTCCTTGAATGACTGGCTATGTCTGGCTTTTGTGGAAAGCAGATTCAACATATCAAAGGTAAATGAAAACGCA---GAC

GGCAGCTTCGACTATGGCATCTTCCAGATCAACAGCCATTACTGGTGCAACGAT------TACCGG------AGTCACTCAGAAAACATTTGCCACGCGGAC------------TGTCAAGACATACTGAGCCCCAATCTTCTCTCAGCCATCAGCTGTACAAAA---------------

---AGGATT---------GTGTCTGGAGCA---GGGGGTATGAAGAACTGGGTAGCATGGAGGTTGCACTGTGCAGGCCGGCCA---CTCTCCTACTGGATGACA------GGATGTTTCTCGGGA------------------------------------------------------

---

>Cat_Lyzl6B

------------------------------------------------------------------------------------------ATGATGAGG---------------------------------------------------ACGCTACTGATGTCCTTG---GTCAGCTGC

---CTCGTTGCCATA---------AATGAGGCCAAGCTCATCAATCGCTGTGACTTGGCCAGAGTGCTGCACAAGGAGGATCTGGATGGGTTTGAGGGCTACTCCCTGAGTGACTGGCTGTGCCTGGCTTTTGTGGAGAGCAACTTCAACCTGTCAAAGGTAAATGAAAATGCA---GAC

GGCAGCTTCGACTATGGCATCTTCCAGATCAACAGCCACTACTGGTGCAACGAT------TACAAG------AGTCACTCGGAAAACATTTGCCACGAGGAC------------TGCAAAGAACTGCTGAGCCCCGATCTTCTCTCAACCATCAACTGTGTGAAA---------------

---AAGATG---------GTGTCTGGAGCA---GGGGGAATGAAGAACTGGGTGGCATGGAGGTTGCACTGTGCGGGCCGGCCA---CTCTCCTACTGGATGACG------GGGTGTCACAAGGAA------------------------------------------------------

---

>Little_brown_bat_Lyzl6

------------------------------------------------------------------------------------------ATGACAAGG---------------------------------------------------GCATTATTCATCTCCTTG---TTCAGCTGC

---CTTGTTGCCATA---------AACCAGGCCAGCCTCATCAACCGCTGTGACTTGGCCAAGCTGCTGCACCAGGAAGATTTGGATGGGTTTGAGGGCTACTCCGTGAGTGACTGGCTGTGCCTAGCTTTTGTGGAAAGCCATTTCAATATGTCAAAGGTAAATGAATATGCA---GAT

GGAAGCTCTGACTATGGCATCTTCCAGATCAACAGCCACTACTGGTGCAACGAT------TACCAG------AGTCACTCGGAAAACACTTGCCACGTGGCA------------TGTCAAGAACTGCTGAGCCCCAATCTTCTCGCAACCATCCACTGTGCAAAA---------------

---AAGATT---------GTGTCCAGAGCA---GGGGGGATGAAGAACTGGGCAGAATGGTCGATACACTGTGCCGGCCGGCCA---CTCTCCTACTGGGTAACA------GGATGTCACATGGGA------------------------------------------------------

---

>Hedgehog_Lyzl6

------------------------------------------------------------------------------------------ATGATGAAG---------------------------------------------------CCAATACTCATCTGCTTG---GTCAGCTGC

---CTCCTCACCATA---------GTCCAGGCAATATTCATCCATCGCTGTGATTTGGCCAAGATCCTGCATGAGGAGGATTTGGATGGGTTTGAGGGATACTCCCTGAGTGACTGGCTCTGCCTGGCTGCTTTGGAGAGTAATTTCAACATATCAAAGGTCAATGAAAATACA---GAC

GGCAGCTTTGACTATGGCATCTTCCAGATCAACAGCCACTACTGGTGCAACGAT------TACAAG------AGTCACTCAGAAAATATTTGCCGTGTGGAC------------TGTAGAGATTTACTGAGCTTGGATCTTCTCCCAACCATCAACTGCATCAAA---------------

---AAAATT---------GTGTCAGGAGCA---GGAGGGATGAAGAACTGGGTAAACTGGAGGTTACACTGTTCAGGCCGGCCA---CTCTCCTACTGGACTACA------GGATGCCACCTAGGA------------------------------------------------------

---

>Mouse_Lyzl6

------------------------------------------------------------------------------------------ATGTTGAAG---------------------------------------------------GCACTGTTCATCTGTGTG---GCAAGCTGC

---CTTCTGGTAGTG---------AATGATGGCAACATCATCCATCGCTGTAGTTTGGCCAAGATTCTGTATGAAGAGGACTTGGATGGGTTTGAGGGCTACTCCCTGCCTGACTGGCTGTGCTTGGCTTTTGTGGAAAGCAACTTCAACATATCAAAGGTGAACGAGAACGTT---GAT

GGTAGCTTCGACTATGGCATCTTCCAGATCAACAGTCGTTACTGGTGCAACGAC------TACCAG------AGTCATTCGGAGAACTTCTGCCATGTCGAC------------TGTCAAGAACTACTGAGTCCCAACCTCATTTCAACCATTCACTGTGCAAAA---------------

---AAGATT---------GTGTCTGGACCA---GGAGGGATGAAGAACTGGGTAGAATGGAAATTGCACTGTTTAGGACGACCA---CTCTCCTACTGGATGACG------GGATGCCATCTGGGA------------------------------------------------------

---

>Rat_Lyzl6

------------------------------------------------------------------------------------------ATGATGAGG---------------------------------------------------GTGCTGTTTATCTGTGTG---GTGAGCTGC

---CTTCTGGTCGTG---------AATGATGGCAGCATCATCAATCGCTGTACCTTGGCCAGGATTCTGTATCAAGAGGACTTGGATGGGTTTGAGGGCTACTCCCTGCCTCACTGGCTGTGCTTGGCTTTTGTGGAGAGCAAATTCAACATATCAAAGGTGACTGAGAATGCA---GAT

GGTACCTTTGACTATGGAATCTTCCAGATCAATAGCCGTTACTGGTGCAATGAC------TACCAG------AGTCATTCAGAGAACTTCTGCCGCCTGGAC------------TGTGAAGAACTACTGAATCCCAACCTCATTCCATCCATCCACTGTGCAAAA---------------

---ATGATT---------GTGTCTGGATCT---GGAGGGATGAAGAACTGGGTAGATTGGAGGTTGCACTGTTTAGGACGGCCA---CTCTCCTACTGGCTGACG------GGATGCCGTCTGGTA------------------------------------------------------

---

>Rabbit_Lyzl6

------------------------------------------------------------------------------------------ATGGCGGGG---------------------------------------------------CCGCTACTCCTCTCCTGG---GCCAGCTGC

---CTCCTGGTGGTG---------AACCAGGCCTTCATCATCCACCGCTGTGACCTGGCCAAGGTGCTGCACGAGCAGGAGCTGGAGGGCTTTGAGGGCTACTCCCTGAGCGACTGGCTGTGCCTGGCTTTTGTGGAAAGCAAGTTCAACACTTCAAAGGTGAACGAAAATGCA---GAT

GGCAGCTCTGACTACGGCATCTTCCAGATCAACAGCCACTTCTGGTGCAATGAT------TATCAC------AGCCACACAGAAAACTTTTGCCACGAGGAC------------TGCCAAGACATGCTGAGCCCCAACCTGCTCTCCTCCATCAGCTGCGCAAAG---------------

---AAGATT---------GTGTCCGGGTCA---AGGGGCATGAAGTACTGGGTAGAATGGAGGCTGCACTGCGCAGGCCGGCCA---CTCTCCTACTGGATGACA------GGATGCCGCCTG---------------------------------------------------------

---

>Shrew_Lyzl6

------------------------------------------------------------------------------------ATGACAGTGGTGAGG---------------------------------------------------ACACTCCTCGTCTGTACA---GTCAGCTGC

---CTTGAGGCTACC---------CATCACGCCCACATGATTAGCCGCTGTGACTTGGCCAAGCTGCTGAAGGAGGAGGACTTGGACGGGTTTGAGGGCTACTCCCTGAGTGACTGGATTTGCCTGGCTTTTGTGGAAAGCTACTTCAACGTATCAAAGGTAAATGAAAACGTA---GAT

GGCAGCTACGACTATGGCATCTTCCAGATCAACAGCCACCTCTGGTGCAACGAT------TACCTC------AGCCACTCAGAAAACATTTGCCACGTGGAA------------TGCAAAGACCTGGTCGTGCCTGATCTTCTCGCATCCGTCAACCGTGCCAAA---------------

---AAGATT---------CTGTCCAGAGCA---GGAGGGATGAGGAACTGGGTGAGGTGGAAGCTGCACTGTGAGGGC---CCA---CTCTCTTCCTGGTTGACC---------TGTCATGTGGAA------------------------------------------------------

---

>Xenopus_LyzD

------------------------------------------------------------------------------------------ATGAAGATC---------------------------------------------------TGTTTGATGATCGTAGTT---------GCT

---GCTGCTGTTGCT---------GGTAACAGCTGGGCATTGGATAGATGCTCAGTAGTGAGAGCCATTCGGAGAGGTGGGCTTGCTGGCATAAAAGGTTATTCCTTGGGGGACTTTGTCTGCTTGGCTTATCATCGCAGTCGATACCATACCTCCCTGCAT---------------AGA

AGCCCAACAAGATATGGCATATTCCAGATCAACAGCTACTGGTGGTGCGACCAT------GGAAAA---ACCCCAGGACGCAAGAATGTGTGTCGAATTCCA------------TGCAGAAATTTGTTAAATACAAACATCGCAGATGATGTGAAGTGCGTAAAA---------------

---AGAATT---------GTGAGTGATCCC---AATGGTCTGGCAGCTTGGGAACCCTGGAAAAAATATTGTAGAGGAAAGAAC---CTCAGCTCCTATGTCAGA------GGATGT---------------------------------------------------------------

---

>Xenopus_LyzE

------------------------------------------------------------------------------------------ATGAAGATC---------------------------------------------------TTTGTTTTGCTTATGATC---------ACT

---GCAGCGTTTGCT---------GCTCACAGCTGGGCATTAGATAGGTGCAGTGTGGTGAGAGCGATTAGAAATGGGGGAGTTATTGGCATCAAAGGATATACACTGGGGGACTATGTGTGCTTAGCTTACCAAGCCAGCCGCTACGACACTTCCCTCAAC---------------AGA

AGCCCAACAGAGTATGGTATCTTCCAGATCAACAGCTACTGGTGGTGCGACGAT------GGCAGA---ACCGTAGGGCGCAAAAACCTCTGTGGAATGTCT------------TGTAGAAGTTTACTGAACAGTAACATTGGCGATGATGTGAGGTGCCTTAGA---------------

---AGGATT---------GTGAGAGATCCC---AATGGACTCGATGCTTGGAGTGTTTGGACCAGATACTGTAAAGGAAGAGAC---CTAAGCTCATACGCCAGT------TGGTGC---------------------------------------------------------------

---

>Wallaby_Spaca3

------------------------------------------------------------------------------------------ATGGAGACC------------------AGGGCCTGGGTGTATCTGCCAAGGAACATTATCCTTGCAATTCTCTCCCTG---ATTAGCTGC

---TTTTTTGCTCCC---------AGTGAAGCCAAAATCTACAGTCGATGTAAAACTGTCCATCTGTTTCAACAAAACGGATTTGATGGCTATCGGGGACATCCCCTAGCTGACTGGGTCTTCTTGGTTTACTCTGCCAGTGGCTTCAATACAGCAGCCTTGGACTATGAAACT---GAT

AGGAGCACCAACAATGGTATCTTTCAGATCAACAGCTGTGAGTGGTGTAATGAC------TACCAC------ACTCCGGGGGTCAACAAATGCAAAATTCAC------------TGCACTGACTTGCTGAACAAGGAT---------GATATTGCCTATGCCATG---------------

---AGGATT---------GTGAGGCAGCCC---CACGGTCTAGCAAGCTTGGAGACCTGGAGGAACAACTGCCAAGGCAAAGAT---CTC------TGGATTGAG------GGTTGTTTCTCT---------------------------------------------------------

---

>Platypus_Spaca3

------------------------------------------------------------------------------CAAGGCTCTGAGCTCAGGGCC---------------------CTGGGCCTGGTGCTGACTCCCTGGAGTGTGGTGCCCGTGCTGCTCCTCTGGGCCGGTTGC

---CTGTTTGCCACC---------AGTGAGGCCAAGATCTATAGTCGCTGCGAGCTGGCCCGCACCCTGCAGGAAGCCGGGCTGGGAGGCTACCGAGGCTACCAAGTGGCTGACTGGGTGTGCTTAGCATACTACGAGAGCGGCTTCGACTCTGGCCTGGAGGACTACGAGATC---GAC

GGAAGCACCAACAATGGCATCTTTCAGATCAACAGCCGCCTGTGGTGCCTGGGT------TACCAG------GACCCTGGTGCCAACCGGTGCCACCTGCAC------------TGCAGTGATAAAATA---------------------------TGCAACAGGGAGGGGACAATGAGT

GTAAGGGTATGGATAGGTGTGAGTGTCCCC---AATGGCTTAGGTGACAGGTCTGCCTGGAAGACCAACTGCGAGGGCAAGGAT---CTGGCGTTTTGGGTGGAA------GGTTGTGAGTTG---------------------------------------------------------

---

>Platypus_Lyzl8

------------------------------------------------------------------------------------------ATGAAGCAC------------------------------------------------------------CTGGTTCTG---CTGTGCATC

---CTGCTGGTCGCC---------TCTCAGGCCCGGTTTATTCAGAAAGGCGAGCTGTGCCAGAAGCTGAAAGCCCAGGGCATGAATGGCTACCTAGGCATCACTCTGCCCAATTGGGTCTGTACTGCATATTATGAAAGTAGTTATAATACTCAAGCCATCAACCACAATAGA---GAC

GGCAGCACCGACTACGGGATCTACCAGATCAACAGCCGTTACTGGTGTCAGGAT------GGTAAA---ACACCTGGAAGCAAGAACATTTGCAAGATCGCC------------TGTTCTATTCGACTGAATGAAACCCTGCAGTGGGTTTGTCTGACACTGCAT---------------

---CATCTG---------TATACAAGTGCT---GAGGGGCTGAGGGTGGGGGTGGCCTGGAAGAATCACTGTCGTAATCGCGAT---CTGTCACAGTTC---------------------------------------------------------------------------------

---

>Zebrafish_Lyz

------------------------------------------------------------------------------------------ATGAGG------------------------------------------------------CTGGCAGTGGTGCTTTTGTGTCTGGCGTGG

---ATGTCCTCG------------TGTGAAAGCAAGACACTGGGACGCTGTGATGTTTACAAGATCTTCAAGAATGAAGGGCTTGATGGATTTGAGGGATTCTCCATTGGCAACTATGTGTGCACTGCCTACTGGGAAAGCAGGTTTAAGACCCACCGAGTGCGTTCAGCT------GAT

ACGGGGAAGGACTACGGGATCTTCCAGATTAACAGCTTCAAGTGGTGTGATGAC------GGGACC------CCTGGTGGGAAGAATTTGTGCAAAGTGGCC------------TGTTCAGACTTGCTTAACGATGACCTGAAAGCTTCAGTTGAGTGTGCAAAG---------------

---CTGATT---------GTGAAAATG------GACGGGCTGAAATCATGGGAAACCTGGGATTCTTACTGTAACGGTCGTAAG---ATGTCCCGCTGGGTAAAA------GGCTGTGAGCAGCGCAAACAAAGCCTCCGAGCC------------------------------------

---

>Platypus_Lalba

------------------------------------------------------------------------------------------ATGAAGCGT------------------------------------------------CTGCTT------------CTG---CTGTGCGTC

---CTGCTGGTCGCC---------TCTCGGGCCAGGATCTTCCAGATCTGCGAGCTGTCCCGAGTACTGAAAGAAAACGGCCTCGGCGGCTTCCACGGCGTCTCTCTGGAGGAATGGCTCTGCGTAATCTTTCACGAGAGCGGTTACGACAGCCAGGCCCTCAACTATTAC------AAC

GGCAGCAGCAGCCATGGGCTCTTCCAGATCAATCAACCGTACTGGTGCGACGACGAAGACTCCGAATCCACCGAGCCCTCCGTGAACGCCTGCCAGATACCT------------TGCAGCAAGCTTCTGGATGATGACATCCTTGACGACATCGAGTGCGCCAAG---------------

---AAGATT---------GTGAAGGAACCC---AAAGGGATCACAGCCTGGGAAGCCTGGCAGCCCTTCTGC---AACGACGAT---CTCGATCAGTGG---AAG------TGT------------------------------------------------------------------

---

>Lizard_LyzE

------------------------------------------------------------------------------------------ATGAGGGTG------------------------------------------------CTAGTTTTTACTCTTGCCCTC---CTTAGCTCT

---GTGCTTGTGGCA---------AGTGAAGCAAAAATAATCCAGAGGTGTCACCTAGCAAAGGAACTTAATGATTTGGGGCTGAGTCAGTACCTTGGTTTCACCCTCGGTGACTGGCTTTGTACAGTCTTTCATGAAAGCGGATTTAACACTGATCCTCCAGTGAGCCCA------ACA

AGACGCAAAGCCTATGGGCTATTCAGGATTAACAATTCAGACTGGTGCTCTGAT------------GGGGTTCAGCCCTCAAAGAATCTCTGCAACATTTCC------------TGTTCTAAGCTAATTGATGATGACATCAAGGATGACATTCTGTGTGCCAAG---------------

---AAAATT---------ATAAAGCAGAAT---GGGCACTTGAAGGCCTGGCCACGCTGGGCTAAAAAGTGTCGTGGAAAAGAT---CTTTCGGTTTATGTGAAA------TGCTGTAATTTT---------------------------------------------------------

---

>Lizard_LyzK

------------------------------------------------------------------------------------------ATGCGG------------------------------------------------------CTTTCCGTCCTCTCCCAC---TTCTGCCTG

---CTTCTTGTAGCA---------AACAAAGCCAAGCCGTTATACAAATGCAACGTAGCCGAGGCCTTGAAGGCGGAAGGGATGGATCGATACGGAGGCTATTCCCTGGGCGACTGGCTATGTGTGATCCATATCACAAGCGGTTTTGACCCCGAGTACGAGGACGATGGCTTT---GCT

GGCCAAAACCATTATGGCATTTTCCAGATTGACAGCCACAATTGGTGCTATGAG------------GATTGTGTCAGTTCAAAGAAGAAGTGCAACGTACCT------------TGCTCAATGTTCTTAGACAAAGACCTCAAGGATGATATTGCATGCGCAAAA---------------

---AAAATT---------GTCTCCTCGTAC---GACGATATTGATTACTGGAAAGTATGGAGAGATTACTGTTATAGAAAGAAC---AATACGAAATGGTTGCAT------AAATGTAAACTG---------------------------------------------------------

---

>Human_Lalba

------------------------------------------------------------------------------------------ATGAGGTTC---------------------------------------------------TTTGTCCCTCTGTTCCTG---GTGGGCATC

---CTGTTCCCTGCC---------ATCCTGGCCAAGCAATTCACAAAATGTGAGCTGTCCCAGCTGCTGAAAGAC------ATAGATGGTTATGGAGGCATCGCTTTGCCTGAATTGATCTGTACCATGTTTCACACCAGTGGTTATGACACACAAGCCATAGTTGAAAAC------AAT

GAAAGCACGGAATATGGACTCTTCCAGATCAGTAATAAGCTTTGGTGCAAGAGC------AGCCAG---GTCCCTCAGTCAAGGAACATCTGTGACATCTCC------------TGTGACAAGTTCCTGGATGATGACATTACTGATGACATAATGTGTGCCAAG---------------

---AAGATC---------CTGGATATT------AAAGGAATTGACTACTGGTTGGCCCATAAAGCCCTCTGCACTGAGAAG------CTGGAACAGTGGCTT------------TGTGAGAAGTTG------------------------------------------------------

---

>Gorilla_Lalba

------------------------------------------------------------------------------------------ATGAGGTTC---------------------------------------------------TTTGTCCCTCTGTTCCTG---GTGGGCATC

---CTGTTCCCTGCC---------ATCCTGGCCAAGCAATTTACAAAATGTGAGCTGTCCCAGCTGCTGAAAGAC------ATAGATGGTTATGGAGGCATCGCTTTGCCTGAATTGATCTGTACCATGTTTCACACCAGTGGTTATGACACACAAGCCATAGTTGAAAAC------AAT

GAAAGCACGGAATATGGACTCTTCCAGATCAGTAATAAGCTTTGGTGCAAGAGC------AGCCAG---GTCCCTCAGTCAAGGAACATCTGTGACATCTCC------------TGTGACAAGTTCCTGGATGATGACATTACTGATGACATAATGTGTGCCAAG---------------

---AAGATC---------CTGGATATT------AAAGGAATTGACTACTGGTTGGCCCATAAAGCCCTCTGCACTGAGAAG------CTGGAACAGTGGCTT------------TGTGAGAAGTTG------------------------------------------------------

---

>Orangutan_Lalba

------------------------------------------------------------------------------------------ATGAGGTTT---------------------------------------------------TTTGTCCCTCTGTTCCTG---GTGGGCATC

---CTGTTCCCTGCC---------ATCCAGGCCAAGCAATTTACAAAATGTGAGCTGTCCCAGCTGCTGAAAGAC------ATAGATGGTTATGGAGGCATCGCTTTGCCTGAATTGATCTGTACCGTGTTTCACACCAGTGGTTATGACACACAAGCCATAGCTGAAAAC------AAT

GAAAGCATGGAATATGGACTCTTCCAGATCAGTAATAAGCTTTGGTGCAAGAGC------AGCCAG---GTTCCTCAGTCAAGGAACATCTGTGACATCTCC------------TGTGACAAGTTCCTGGATGATGACATTACTGACGACATAATGTGTGCCAAG---------------

---AAGATC---------CTGGATATT------AAAGGAATTGACTACTGGTTGGCCCATAAAGCCCTCTGCACTGAGAAG------CTGGAACAGTGGCTT------------TGTGAGAAGTTG------------------------------------------------------

---

>Macaque_Lalba

------------------------------------------------------------------------------------------ATGAGGTCA---------------------------------------------------TTTGTCCCTCTGTTCCTG---GTGGGCATC

---CTGTTCCCTGCC---------ATCCCGGCCAAGCAATTTACAAAATGTGAGCTGTCCCAGCTGCTGAAAGAC------ATAGATGGTTATGGAGGCATCGCTTTGCCTGAATTCATCTGTACCATGTTTCACACCAGTGGTTATGACACACAAGCCATAGTTGAAAGC------AAT

GGAAGCACAGAATATGGACTCTTCCAGATCAGTAATAAGCTTTGGTGCAAGAGC------AGCCAG---GTCCCTCAGTCAAGGAATATCTGCGACATCTCC------------TGTGACAAGTTCCTGGACGATGACATTACTGATGACATAATGTGTGCCAAG---------------

---AAGATC---------CTGGATATT------AAAGGAATTGACTACTGGTTGGCCCATAAAGCCCTCTGCACTGAGAAG------CTGGAACAGTGGCTT------------TGTGAGAAGTTG------------------------------------------------------

---

>Baboon_Lalba

------------------------------------------------------------------------------------------ATGAGGTCG---------------------------------------------------TTTGTCCCTCTGTTCCTG---GTAGGCATC

---CTGTTCCCTGCC---------ATCCCGGCCAAGCAATTTACAAAATGTGAGCTGTCCCAGCTGCTGAAAGAC------ATAGATGGTTATGGAGGCATCGCTTTGCCTGAATTCATCTGTACCATGTTTCACACCAGTGGTTATGACACACAAGCCATAGTGGAAAGC------AAT

GGAAGCACAGAATATGGACTCTTCCAGATCAGTAATAAACTTTGGTGCAAGAGC------AGCCAG---GTCCCTCAGTCAAGGAATATCTGCGACATCTCC------------TGTGACAAGTTCCTGGACGATGACATTACTGATGACATAATGTGTGCCAAG---------------

---AAGATC---------CTGGATATT------AAAGGAATTGACTACTGGTTGGCCCATAAAGCCCTCTGCACTGAGAAG------CTGGAACAGTGGCTT------------TGTGAGAAGTTG------------------------------------------------------

---

>Marmoset_Lalba

------------------------------------------------------------------------------------------ATGATGTCC---------------------------------------------------TTTGTCCCTCTGCTCCTG---GTGGGCATC

---CTGTTCCCTGCC---------ATCCCTGCCAAGCAATTTACAAAATGTGAGCTGTCCCAGGAGCTGAAAGCC------ATGGATGGTTATAGAGGCATCTCTTTGCCTGAATTCATCTGTACCATGTTTCACACCAGTGGTTATGATACACGAGCCATAGCTGAAAAC------AAG

GGCAGCACGGAATATGGACTCTTCCAGATCAGTAATAAGCATTGGTGCAAGAGC------AACCAG---ATCCCTCAGTCAAGGAATATCTGTGACATCTCC------------TGTGACAAGTTCCTGGATGATGACATTACTGATGACATAATGTGTGCCAAG---------------

---AAGATC---------CTGGACATT------AAAGGAATTGACTACTGGTTGGCCCATAAAGCCCTCTGCACTGATAAC------CTGGAACAGTGGCTT------------TGTGAGAAGTTG------------------------------------------------------

---

>Pig_Lalba

------------------------------------------------------------------------------------------ATGATGTCC---------------------------------------------------TTTGTCTCTCTCCTCGTG---GTGGGGATT

---CTCTTTCCTGCC---------ATCCAGGCCAAGCAATTTACAAAATGTGAGCTGTCCCAGGTGCTGAAAGAC------ATGGATGGCTATGGAGACATCACTTTGCCTGAATGGATCTGTACCATATTTCATATCAGTGGCTATGACACAAAAACCATTGTGCATGAC------AAT

GGCAGCACAGAATATGGACTCTTCCAGATCAATAATAAACTCTGGTGCAGAGAC------AACCAG------ATCCAGTCAAAGAACATCTGTGGCATCTCC------------TGTGACAAATTCCTGGATGATGACCTTACTGATGACATGATGTGTGCCAAG---------------

---AAGATC---------CTGGATAAT------GAAGGGATTGACTACTGGTTGGCCCATAAAGCACTCTGTTCAGAAAAA------CTGGATCAGTGGCTC------------TGTGAGAAGATG------------------------------------------------------

---

>Little_brown_bat_Lalba

------------------------------------------------------------------------------------------ATGATGTCC---------------------------------------------------TTCTTGTCTCTGCTCCTG---GTGGGCATT

---CTGTTCCCTGCC---------CTTGAGGCCAAGCAATTTACAAAATGTGAGCTGTCCCAGGTGCTGAAAGAC------ATGGATGGCTATGGAGGCGTCACTCTACCTGAATGGATCTGTAACATATTTCATGTCAGTGGTTATGACACACAAACCATGGTCAATAAC------AAT

GGCAAGACAGAATATGGACTCTTCCAGATCAATAACAAACTTTGGTGCAGGGAC------AATCAG------ATACAGTCAAGGAACATCTGTGACATCTCC------------TGTGATAAATTCTTGGATGATGACCTTACTGATGACATGATATGTGCCAAG---------------

---AAGATC---------CTGGATAGT------GAAGGAATTGACTACTGGTTGCCCCATAAGCCACTCTGCTCCGAGAAG------CTGGAACAGTGGCTC------------TGCGAGAAGTTG------------------------------------------------------

---

>Dog_Lalba

------------------------------------------------------------------------------------------ATGATGTCC---------------------------------------------------TTTGTCTCTCTGCTCCTG---GTTAGCATC

---TTGTTCCCTGCC---------ATCCAGGCAAAGCAATTTACAAAATGTGAGCTGTCCCAGGTGCTGAAAGAC------ATGGATGGCTTTGGAGGCATTGCTTTGCCTGAATGGATCTGTACTATATTTCATACCAGTGGTTATGATACACAAACCATAGTCAATAAC------AAT

GGTGGCACAGACTATGGACTTTTCCAGATCAGCAATAAATTTTGGTGCAAGGAC------GACCAG---AACCTTCAGTCAAGGAACATCTGTGACATCTCC------------TGTGACAAGTTCCTGGATGATGACCTTACTGATGACATGATTTGTGCCAAG---------------

---AAGATC---------CTGGATAAG------GAAGGAATTGACTACTGGTTGGCCCATAAACCACTCTGCTCTGAGAAG------CTGGAGCAGTGGCGC------------TGTGAGAAGTTG------------------------------------------------------

---

>Cat_Lalba

------------------------------------------------------------------------------------------ATGATGTCC---------------------------------------------------TTTGTCTCTCTGCTCCTG---ATTGGCATC

---ATGTTCCCTGCC---------ATCCAGGGGAAGCAATTTACAAAATGTGAGCTGTCCCAGGTGCTGAAAGAC------ATGGATGGCTATGGAGGCATTGCTTTGTCTGAATGGATCTGTACCATATTTCATACTAGTGGTTATGATACACAAACCATAGTCAATAAC------AAT

GGCAGCACAGAATATGGACTCTTCCAGATCAACAATAAATTTTGGTGCAGGGAC------AACCAG---ATCCTTCAGTCAAGGAACATCTGTGACATCTCC------------TGTGACAAGTTCCTGGATGATGACCTTACTGATGACATGATTTGTGCCAAG---------------

---AAGATC---------CTGGATAAG------GAAGGAATTGACTACTGGTTGGCCCATAAACCACTCTGCTCTGAGAAA------CTGGAACAGTGGCAC------------TGTGAGATGTTG------------------------------------------------------

---

>Elephant_Lalba

------------------------------------------------------------------------------------------ATGATGTCC---------------------------------------------------TTCGTCCCTCTGCTCCTG---GTAGGCATA

---CTGTTCCCTGCC---------ATCCAGGCCAAACAATTTACAAAATGTGAGCTGTCCCAGGTCCTGAAAGAC------ATAGACGGCTATGCAGGCATCACTTTGCCAGAATTTACCTGTACCATATTTCATATCAGTGGTTATGATACACAAACCATAGTTAATAAC------AAT

GGCAGCACAGAATATGGACTCTTCCAGATCAGTAATAAATACTGGTGCAGGGAC------CACCAG---ATCCCTCAGTCAAGGAACATCTGTGACATCTCC------------TGTGACAAGTTCCTAGATGATGACCTTACTGATGACATGATGTGTGCCAAG---------------

---AAGATC---------CTGGATAGC------AAAGGAATTGACTACTGGTTGGCCCATAAACCTCTCTGCTCTGAGAAG------CTGGAACAGTGGCTC------------TGTGAGAAGTTG------------------------------------------------------

---

>Horse_Lalba

------------------------------------------------------------------------------------------ATGATGTCC---------------------------------------------------TTCGCCTCACTGCTCCTG---GTGGGCATC

---CTGTTCTCTGCC---------ACCCAGGCCAAGCAATTTACAAAATGTGAGCTGTCCCAGGTGCTGAAATCC------ATGGATGGCTATAAAGGCGTCACTTTGCCTGAATGGATCTGTACCATATTTCATAGCAGTGGTTATGACACACAAACCATAGTTAAAAAT------AAC

GGCAAAACAGAATATGGACTCTTCCAGATCAATAATAAAATGTGGTGCAGGGAC------AACCAG---ATCCTCCCATCAAGGAACATCTGTGGCATCTCC------------TGTAACAAGTTCCTGGATGATGACCTTACTGATGACGTGATGTGTGCCAAG---------------

---AAGATC---------CTGGATAGT------GAAGGAATTGACTACTGGTTGGCTCATAAGCCCCTCTGCTCTGAGAAG------CTGGAACAGTGGCTC------------TGCGAGGAGTTG------------------------------------------------------

---

>Fox_bat_Lalba

------------------------------------------------------------------------------------------ATGATG---------------------------------------------------------CTCTCTCTGCTTCTG---GTGGGCATT

---CTGTTGCCCGCC---------ACTCAGGCCAAGCAATTTACCAAATGTGAGCTGTCCCAGGTGCTGAAAGAT------ATGGATGGCTATGGAGGCATCACCTTACAAGAATGGATCTGTACCATATTTCATAGCAGTGGTTATGACACAAAAACCATAATCAACAAC------AAT

GGAAAGAGAGAGTATGGACTCTTCCAGATCAATAACAAACTTTGGTGTAGGGAC------AATCGG---AAACTTCAGTCAAGAAACATCTGTGGCATCTCT------------TGTGATAAGTTCTTGGATGATGACCTTACTGATGACGTGATGTGTGCCAAG---------------

---AAGATC---------TTGGATAGT------GAAGGAATCGACTATTGGTTGGCCCATAAACCACTCTGCTCTGAGAAG------CTGGAACAGTGGCGC------------TGTGAGAAGTTG------------------------------------------------------

---

>Hyrax_Lalba

------------------------------------------------------------------------------------------ATGATGTCC---------------------------------------------------TTCATCCCTCTGCTTCTG---GTAGGCATA

---CTGATCCCTGCC---------ATCCAGGCCAGACAATTGACAAAATGTGAAGTGTCCCAGGTACTGAAAGAC------ATGAATGGCTATGCAGGCATCATCATGTCAGAATTAATTTGTACTGTATTTCATAGCAGTGCTTTTGATACACAAGCCATAGTTAATAAC------AGT

GACAGCACAGAATATGGACTCTTCCAGATAAGTAATAAACTTTGGTGCAGAGAC------CACCAG---ATTCCTCAGTCAAGGAACATCTGTGGCATCTCC------------TGTGACAAGTTCGTGGATGATGACATTACTGATGACATAATGTGTGCCAAG---------------

---AAGATC---------CTTGATAGT------GAAGGAATTGACTACTGGCCAGCCCATAGACCTCTCTGTTCTGAGAAG------CTGGAACAGTGGCAG------------TGCGAGAAACCC------------------------------------------------------

---

>Cow_Lalba

------------------------------------------------------------------------------------------ATGATGTCC---------------------------------------------------TTTGTCTCTCTGCTCCTG---GTAGGCATC

---CTATTCCATGCC---------ACCCAGGCTGAACAGTTAACAAAATGTGAGGTGTTCCGGGAGCTGAAAGAC------TTGAAGGGCTACGGAGGTGTCAGTTTGCCTGAATGGGTCTGTACCACGTTTCATACCAGTGGTTATGACACACAAGCCATAGTACAAAAC------AAT

GACAGCACAGAATATGGACTCTTCCAGATAAATAATAAAATTTGGTGCAAAGAC------GACCAG---AACCCTCACTCAAGCAACATCTGTAACATCTCC------------TGTGACAAGTTCCTGGATGATGATCTTACTGATGACATTATGTGTGTCAAG---------------

---AAGATT---------CTGGATAAA------GTAGGAATTAACTACTGGTTGGCCCATAAAGCACTCTGTTCTGAGAAG------CTGGATCAGTGGCTC------------TGTGAGAAGTTG------------------------------------------------------

---

>Dolphin_Lalba

---------------------------------------------------------------------------------------------ATGTCC---------------------------------------------------TTTGTCTCTCTGCTCTTG---GTGGGCATC

---CTGTTCCATGCC---------GTCCAGGCCGATCAATTAACAAAATGTGAGCTGTTCCAGAGGCTG---GAC------CTGCATGGCTATGGAGGCGTCACTTTGCCTGAATGGGTCTGTACCGTATTTCATACTAGTGGGTGTGACACACAAACCATAGTAAATAAC------AAT

GACAGCACAGAATATGGACTTTTCCAGATCAATAATAAAATTTGGTGCAGAGAC------AACCAG---ATCCCTCACTCAAGGGACATCTGTGGCATCTCC------------TGTGACAAGTTCCTGGATGATGACCTTACTGATGACATTATGTGTGTCAAG---------------

---AAGATT---------CTGGATAAT------GTA---ATTAACTACTGGTTGGCCCATAAAGCACTCTGTTTTGAGAAG------CTGGATCAGTGGCGC------------TGCGAGAAGTGG------------------------------------------------------

---

>Shrew_Lalba

------------------------------------------------------------------------------------------ATGATGTAC---------------------------------------------------TTTGTCTCTCTGCTCCTG---GTGGGCATC

---CTGTTCCCTGCA---------ATCCAAGCCAAGCAATTTACAAAATGTGAGCTGTACCGGGAGCTGTCAGAC------CTGAGTGACTAT---GATTTCACTCTACAAGAACTGATCTGTGTTATATTTCACATCAGTGGCTATGACTCACGGGTTGTACTTAATAAT------AAT

GGCAGCACAGAATATGGACTCTTCCAGATCAGTAATAAAAACTGGTGTCGTGAT------AACAAG---TTCCCTGAGTCAGAGAACATATGCAACATTTCT------------TGTGAGAAGTTCCTCGATGATGACCTGACTGATGATGTGATGTGTGTAAAG---------------

---AAGATC---------CTTGATATG------AAAGGAATTGACTACTGGTTGGCTCATAAGGCACTCTGTTCTGAGAAG------CTGGACCAGTGGAGC------------TGCAAGAAATTG------------------------------------------------------

---

>Armadillo_Lalba

------------------------------------------------------------------------------------------ATGATGTCT---------------------------------------------------TACCTCCCTTTGCTTCTG---GTGGGCATC

---CTGTTCCCTGTC---------ATTCAGGCCAGCCAATTGACAAAATGTGAACTGCCCCAGAAGCTGAAAGAC------ATAGATGGCTATCGAAACGTCACTTTGCCGGAATGGATCTGTACCACATTTCATATCAGTGGTTTTGACACACAAACCATAGTCAATAAC------AAT

GACAGCATAGAATATGGACTCTTCCAGATCAGTAATAAATGTTGGTGCAGGGAC------GACCAG---AACCCTCTGTCAAAGAACATCTGTAACACCACC------------TGTAACAAGTTCCTGGATGATGACCTTACTGATGACATCATGTGTGTCAAG---------------

---AAGATC---------CTGGATATT------GAAGGAATTGGCTACTGGTCAGCCCATAAAGCACTTTGCTCTGACAAG------CTGGAACAGTGGCTC------------TGCAAGACGTCG------------------------------------------------------

---

>Pika_LalbaA

------------------------------------------------------------------------------------------ATGATGTCC---------------------------------------------------CTGGTCCTTCTGCTCCTA---GTGAGCAGC

---GTCTTCTCCGCC---------ATCCAGGCGACACAACTGACTAAGTGTGAGCTGTCCGCGAAGCTGAAAGAC------TTGGATGGCTATGGAAATATCCCGATGTCTGAGTGGATATGTACCTTGTTTCATACCAGTGGTCTTGACACACAGATTACAGTCAACAAC------AAT

GACAGCACAGAATATGGGCTCTTCCAGATCAGTGACAAACTTTGGTGCAAGAGC------AAACGG---AACCCTCGGTCGAGGAACATCTGTGACATCGCT------------TGTGAAAAGTTCTTGGATGATGACCTTATTGATGACATAATGTGTGCCAAG---------------

---AAGATT---------CTGGATAAA------GAAGGCATTGACCACTGGCTGGCACATAAACCACTCTGCTCTGAGAAC------CTGGAACAGTGGGTC------------TGTAAGAAGTTG------------------------------------------------------

---

>Rabbit_Lalba

------------------------------------------------------------------------------------------ATGATGCCT---------------------------------------------------CTTGTCCCTCTGCTCCTG---GTGAGCATC

---GTCTTCCCCGGC---------ATCCAGGCTACACAACTGACGAGGTGTGAGCTGACTGAGAAGCTGAAGGAG------CTGGATGGATACAGAGATATCTCTATGTCTGAGTGGATATGTACCTTATTTCATACCAGCGGCCTTGACACAAAGATTACAGTCAATAAC------AAT

GGCAGCACAGAATATGGAATCTTCCAGATCAGCGATAAGCTGTGGTGTGTGAGC------AAACAG---AACCCTCAGTCGAAGAACATCTGTGACACCCCC------------TGTGAAAATTTCTTGGATGATAACCTTACTGATGATGTAAAGTGTGCCATG---------------

---AAGATC---------CTGGATAAA------GAAGGGATTGACCACTGGTTGGCACATAAACCACTCTGCTCTGAGAAC------CTGGAACAGTGGGTC------------TGTAAGAAG---------------------------------------------------------

---

>Guinea_pig_Lalba

------------------------------------------------------------------------------------------ATGATGTCC---------------------------------------------------TTTTTCCCTCTGTTGCTG---GTGGGCATC

---CTGTTTCCTGCC---------GTGCAGGCCAAGCAACTTACCAAATGTGCGCTGTCTCATGAGTTGAACGAC------CTTGCCGGCTACCGAGACATCACTTTGCCTGAATGGCTCTGTATCATATTTCATATCAGTGGTTATGACACACAAGCCATAGTGAAAAAT------AGT

GACCACAAAGAGTACGGACTTTTCCAGATAAATGATAAAGATTTCTGTGAGAGC------AGCACG---ACTGTTCAATCAAGGAACATTTGTGACATTTCC------------TGCGACAAGCTCCTGGATGATGACCTTACTGATGACATAATGTGTGTCAAG---------------

---AAGATC---------CTGGATATC------AAAGGAATTGACTACTGGTTGGCCCACAAACCACTGTGCTCTGACAAG------CTGGAGCAGTGGTAC------------TGCGAGGCACAG------------------------------------------------------

---

>Mouse_Lalba

------------------------------------------------------------------------------------------ATGATGCAT---------------------------------------------------TTCGTTCCTTTGTTCCTG---GTGTGTATT

TTGTCGTTGCCTGCC---------TTTCAAGCCACAGAGCTTACAAAATGCAAGGTGTCCCATGCCATTAAAGAC------ATAGATGGCTATCAAGGCATCTCTTTGCTTGAATGGGCCTGTGTTTTATTTCATACCAGTGGCTACGACACACAAGCTGTTGTCAACGAC------AAC

GGCAGCACAGAGTACGGACTCTTCCAGATCAGTGACAGATTTTGGTGTAAAAGT------AGTGAG---TTCCCCGAGTCGGAGAACATCTGTGGCATCTCC------------TGTGACAAGTTATTGGATGACGAGTTGGATGATGACATAGCGTGTGCCAAG---------------

---AAGATC---------CTGGCTATC------AAAGGAATCGACTACTGGAAAGCCTACAAGCCCATGTGCTCTGAGAAG------CTTGAACAGTGGCGT------------TGTGAGAAGCCC------------------------------------------------------

---

>Rat_Lalba

------------------------------------------------------------------------------------------ATGATGCGT---------------------------------------------------TTTGTTCCTCTGTTCCTG---GCGTGTATT

---TCGCTGCCTGCC---------TTTCAAGCCACAGAGTTTACAAAATGTGAGGTGTCCCACGCCATTGAAGAC------ATGGATGGCTATCAAGGCATCAGCTTGCTTGAATGGACCTGTGTTTTATTCCACACCAGTGGCTATGACTCACAAGCTATGGTCAAGAAC------AAT

GGCAGCACAGAGTACGGACTCTTCCAGATCAGTAACAGAAATTGGTGCAAGAGT------AGCGAG---TTCCCCAAGTCAGAGAACATCTGTGACATCTCC------------TGTGACAAGTTCCTGGATGATGAGCTTGCGGATGACATAGTATGTGCCAAG---------------

---AAGATC---------GTGGCTATC------AAAGGGATCGACTACTGGAAGGCTCACAAGCCCATGTGCTCTGAGAAG------CTGGAACAGTGGCGC------------TGTGAGAAGCCGGGAGCTCCTGCCCTTGTTGTCCCTGCCCTGAATTCAGAAACACCTGTTCCC---

---

>Tenrec_Lalba

---------------------------------------------------------------------------------------------ATGGCT---------------------------------------------------TTCTTTCCTCTACTCCTG---GTGGGCATA

---CTGCTTTCTGCT---------CTCCAGGCCACACAAGTAACAAAATATGAACTATGCCAGATGCTGAAAGAG------CTGGATGGCTATCGATCCATCACTTTGTCAGAATGGATCTGTATCATATTTCATATGAGTGGTTGTGATACACAAACCATAATTAATTAC------AAT

GGCAGCACAACATACGGACTCTTCCAGATCAGCAATAAATTTTGGTGCAGGGAC------GACCAG---TTCCCTCAGTCATGGGACATCTGTGACATCGCC------------TGTGACAACTTCCTGAATGATGACTTATCTGATGACATACAGTGTGTCAAG---------------

---AAGATT---------CTGGACAGT------GAAGGGATTGGCTACTGGCCAGCCTATAAACCTCTCTGCTCTGACAAC------TTGGAACAGTGGCTC------------TGCAAGTCA---------------------------------------------------------

---

>Opossum_Lalba

------------------------------------------------------------------------------------------------------------------------------------------------------ATGAAGTCTCTGCTCCTA---CTCAGCATC

---ATACTCTCAGCC---------ACCCAGGCTAGAGAATTAACGAAATGTGAGCTGATCCAGGATCTAAAGAATCATGGCATGGACAAATATGAAGACTTCCATTTGAATGAAATGATCTGCGTCACATTCCATAGCAGTGGCTTTAACACCCAAATTAAAGTCAGTAAC------AAT

GGCAACACAGAATACGGAATATTTCAGATCAGCAACAATGGTTGGTGTGCCGAA------AAGCAG---GAAGATGTGGCCAAAAGTACTTGTGGCATTCTT------------TGCTCCAAACTCCTGGATGATGACATTAATGATGACATCGTGTGTGCCAAG---------------

---AAGATC---------ATAGAGCAACCC---AAAGGAATTGACTACTGGAAAGCCCATAAAACATTCTGCCTTGAGAAC------CTAGACCAATGGAGG------------TGCTTGAAT---------------------------------------------------------

---

>Wallaby_Lalba

------------------------------------------------------------------------------------------ATGATGTCT---------------------------------------------------CTGCTCTCTTTGCTCCTG---CTTGGCATT

---GCGCTCCCAGCC---------ACCCAGGCCATAGATTACAGGAAATGTCAAGCGTCGCAGATATTGAAAGAACATGGCATGGACAAAGTT------ATCCCTCTGCCTGAATTGGTCTGTACCATGTTCCATATCAGTGGCCTTAGCACCCAAGCTGAGGTCAATAAC------CAC

AGCAACAAAGAATACGGAATATTCCAGATCAGCAATAATGGCTGGTGTGCTGAA------AAGCAG---GAAGATGTAGCAAACAGTGTCTGTGGCATTCTT------------TGCTCTAAGTTCCTTGATGATGACATTACTGATGACATTGAGTGTGCCAAG---------------

---AAGATC---------CTTCAGCTACCT---GAAGGCCTTGGCTACTGGAAGGCCCATGAAACTTTTTGCATTGAGGAC------CTGGACCAGTGGCGC------------TGC---------------------------------------------------------------

---

>Lizard_LyzO

------------------------------------------------------------------------------------------ATGGCTGCT---------------------------------------------------TGGCTTCCTCGGCTCCTCTGCCTTTGGCTC

---CTTTCAGGGGCC---------TCACTAGCCGTGACGTTTCATGAATGTCCGCTGGCCAAGATTTTCCTTGAGGAAGGGCTGGACGGATACGCAGGCATCTCTTTGGAACAGTGGATGTGTATAGCATTTCACTCAAGTTCGTACAATACGGATCACACCAACAAGTATGGA------

------------TTTGGATTGTTTCATTTCGACAGACAAGTTTGGTGCAAAACG------GGCGAG------GAGGACTCACTGAACCTCTGCAACATATGC------------TGTTCAAAGTTGGCAGATGCTGACATCCACGATGACATTAAGTGCGTCAAA---------------

---AAAATT---------GTGCGCTCACCA---GATAAACTAACCCTATTCTGGGCATGGAACTCGGTGTGCAGGTCTGACTCT---ATAGGGCATTGGGCTGGAAAAGTGAACTGCAAAAGGCTCAATCGTCCATCGAAGAATATATTACGAAAGAAAGGCAGTGCTGTTCTTGATTTC

TTC

>Amphyoxus_LyzA

---------------------------------------------------------------------------------------------ATGTTT---------------------------------------------------CTGGCTGTGGTGCTGTTG---ATGGGGGTG

---GTGTGCACG------------GCGCATGCCAAGACCTACGAGAAGTGTGAACTGGCGAGAGAGCTGGTGTCACGTGGTCTGACTACACGATCT------CAGGCCGGCGAATGGATCTGTCTGGTCCAGCACGAGAGTAGCTTCAGGACCGGTGCGTTGGGCGGCCCCAACGGGGAT

GGTTCCTACGATCACGAGTTGTTCCAGATCAACGACTACTACTGGTGC------GACGACGGT------------GGACCACATAACGACTGTGGTGTCTCC------------TGCTCAGCCCTCCGTGACAACAACATCGCTGATGACGTACGATGTGCCAAG---------------

---CTGATC---------TACCAGCGC------CATGGCTTCAATGCCTGGTATGGCTGGATCAACCACTGCCAGGGTCACAAC---AACGCAAACCTGGTCACC------AGCTGCTGG------------------------------------------------------------

---

>Amphioxus_LyzB

---------------------------------------------------------------------------------------------ATGTTC---------------------------------------------------CTGGCTGTGGTACTAGTG---ATGGGGCTT

---GTGTGCTCG------------GCGCATGCCAAGACCTACGAGCGCTGTGAACTGGCGAGAGAGCTGGTGTCCCGCGGGCTGACTTCGCGATCC------CAGGCCGGCGAATGGATCTGTCTGGTTCAGCACGAGAGTTCCTTCAGGACTGGAGCCAGAGGCGGCCCCAACTGGGAC

GGATCCTACGATCACGGGCTGTTCCAGATCAACGATCACTACTGGTGC------GACAACGGT------------GGACCACACAACGACTGTGGCGTCTCC------------TGCTCCAACCTCCGTGACAACAACATCGCTGATGACGTCAGGTGTGCCAAG---------------

---CTGATC---------TACCAGCGT------CACGGCTTCTCTGCCTGGTATGGCTGGCAAAACAACTGCCAGGGC---AGC---ACCTCCAGCTACGTCAGC------GGCTGTTTC------------------------------------------------------------

---

>Bombyx_Lyz

---------------------------------------------------------------------------------------------ATGCAG---------------------------------------------------AAGTTAATTATTTTCGCT---TTGGTTGTC

---CTCTGCGTTGGT---------TCTGAAGCCAAAACGTTCACGAGATGCGGTCTCGTGCATGAGCTGAGGAAGCATGGCTTC---GAAGAAAAT------CTCATGAGGAACTGGGTATGTCTGGTCGAGCACGAGAGCAGCCGTGACACGTCCAAG---ACGAACACGAACCGTAAC

GGCTCGAAGGACTACGGATTGTTCCAGATCAACGACCGGTACTGGTGC------AGCAAAGGCGCC---------AGTCCGGGCAAAGACTGCAACGTTAAG------------TGCTCCGACCTCCTGACTGACGACATTACTAAGGCAGCGAAATGCGCTAAG---------------

---AAAATT---------TACAAACGT------CACCGCTTCGATGCCTGGTACGGTTGGAAGAACCACTGCCAGGGC---TCT---CTGCCTGAT---ATTAGC------AGCTGC---------------------------------------------------------------

---

>Anopheles_Lyz

---------------------------------------------------------------------------------------ATGAAAGTGTTT---------------------------------------------------TCCACAGTTTTGCTCGCC---ATCGTCGCG

---TGTTGTGCCGTA---------GCCGAAGCTAAAACGTTCGGCAAATGTGAACTGGCAAAGGCGCTGGCCAACAATGGTATC---GCGAAAGCA------TCGTTGCCGGATTGGGTCTGTCTGGTGCAGAACGAGAGTGCGTTCAGCACATCGGCG---ACAAACAAGAACAAGAAC

GGTTCGACCGATTACGGCATCTTCCAGATCAACAACAAGTACTGGTGC------GATTCGGGCTAC---------GGT---TCAAACGACTGCAAGATCGCG------------TGCAAAAATTTACTAAACGACGATATTACGGATGACATCAAGTGTGCCAAG---------------

---CTGATC---------CACAAACGT------CATGGATTTAACGCCTGGTACGGCTGGAAGAACCACTGCAATGGCAAGAAG---CTGCCGAAT---GTTAGC------TCCTGTTTT------------------------------------------------------------

---

>Drosophila_LyzB

------------------------------------------------------------------------------------------ATGAAGGCT---------------------------------------------------TTCATCGTTCTGGTTGCC---CTGGCTCTG

---GCCGCTCCTGCT---------CTT---GGTCGCACCATGGACCGTTGCTCCCTGGCCCGGGAGATGTCCAACCTGGGCGTT---CCTCGTGAC------CAATTGGCTCGTTGGGCCTGCATTGCCGAGCACGAGTCCTCCTACCGCACCGGAGTGGTTGGTCCCGAGAACTACAAC

GGCTCCAACGACTACGGAATCTTCCAGATCAACGACTACTACTGGTGCGCTCCTCCCAGCGGTCGC---------TTCTCCTACAATGAGTGCGGGTTGAGC------------TGCAATGCCCTCTTGACCGACGACATCACCCACTCCGTCCGTTGTGCCCAG---------------

---AAGGTC---------CTCAGCCAG------CAGGGATGGTCCGCCTGGTCCACCTGG---CACTACTGCAGCGGA---TGG---TTGCCGTCC---ATCGAT------GACTGCTTC------------------------------------------------------------

---

>Drosophila_LyzS

------------------------------------------------------------------------------------------ATGAAGGCT---------------------------------------------------TTCTTTGCTCTGGTGCTC---CTGGCCATT

---GCCGCTCCTGCC---------CTGGCAGGTCGCACCCTCGACCGCTGCTCGCTGGCCCGCGAGATGGCCGACTTGGGAGTT---CCCCGCGAC------CAGCTGGACAAGTGGACCTGCATTGCCCAGCACGAGAGTGACTACCGCACCTGGGTGGTGGGACCTGCCAACTCCGAT

GGCTCCAACGACTACGGAATCTTCCAGATCAACGATCTGTACTGGTGCCAG---GCCGACGGACGC---------TTCTCCTACAACGAGTGCGGCTTGAGC------------TGCAATGCCCTCTTGACCGACGACATCACCAACTCTGTCCGTTGCGCCCAG---------------

---AAGGTC---------CTCAGCCAG------CAGGGATGGTCCGCCTGGGCCGTCTGG---CATTACTGCAGCGGA---TGG---TTGCCGTCC---ATCGAT------GAGTGCTTC------------------------------------------------------------

---

>Drosophila_LyzP

------------------------------------------------------------------------------------------ATGAAAGCT---------------------------------------------------TTTCTTGTGATTTGTGCT---CTGACTCTC

---ACAGCAGTCGCT---------ACCCAGGCCCGAACGATGGATAGGTGTTCCCTAGCCAGGGAGATGTCCAAACTGGGCGTT---CCCCGGGAC------CAGCTGGCCAAGTGGACCTGCATCGCCCAGCACGAGAGTTCCTTCCGCACCGGCGTCGTTGGACCGGCCAACTCCAAC

GGATCCAATGACTACGGCATCTTTCAGATCAACAACAAGTACTGGTGCAAGCCGGCCGACGGACGC---------TTCTCCTACAACGAGTGCGGCTTGAGC------------TGCAATGCCCTCCTAACCGACGACATCACCAATTCCGTGAAGTGCGCCCGG---------------

---AAGATC---------CAGCGCCAG------CAGGGATGGACTGCTTGGTCCACATGG---AAGTACTGCAGCGGA---TCC---CTGCCCTCG---ATCAAC------AGTTGCTTC------------------------------------------------------------

---

>Drosophila_LyzX

------------------------------------------------------------------------------------------ATGAGAGCC---------------------------------------------------CTCCTAGGAATCTGTGTC---CTGGCACTA

---GTCACTCCAGCT---------GTCCTGGGTCGCACCATGGATCGCTGCTCACTGGCGCGCGAGATGGCCAATATGGGTGTT---TCTCGTGAC------CAGTTGTCCAAGTGGGCGTGCATTGCAGAGCACGAGAGCTCCTACCGCACCGGAGTGGTGGGGCCTCCCAACACCGAT

GGATCCAACGACTATGGCATTTTCCAGATCAACGACATGTACTGGTGCCAGCCGTCCAGTGGAAAG---------TTCTCCCACAATGGCTGCGATGTGAGT------------TGCAACGCTCTCTTGACCGATGACATCAAAAGTTCCGTAAGATGTGCCCTA---------------

---AAGGTC---------CTGGGTCAA------CAGGGCTGGTCAGCCTGGTCCACCTGG---CACTACTGCAGTGGA---TAC---CTGCCCCCG---ATCGAT------GATTGCTTTGTT---------------------------------------------------------

---

>Lizard_LyzM

------------------------------------------------------------------------ATGCCGATTTTGAAACTCATGGCTCCT---------------------------------------------------TTTTTAATCTTCCTGCTC---ATTGGGGCT

------------------------GGTGAAACCAAGATCATTTCGCGATGTGCCCTGGCTAATCTCATTGCCGATTTAGTCCCGGACGGGTATGCGCGATACACTATCAATGACTGGATCTGTCTCGCCCACTCTGCGAGCTATTTCGATTCAATGAGAATATCAAGACAGGAAGAGGAC

AGTGCCCGGAGATATGGGATATTCCAGTTCAGTAACATGGAACATTGCTCCGAC------GGATGC------CGCAGCTCCCTGAACATATGCCGCACGCAA------------TGCACAAACTTCATAAATGATGACTTGAGGGACGATATCCGGTGTGTGATG---------------

---AAAATA---------ATCAGAGCTGAC---ACTGGGTTCGACGAATGGAGAGTATATGGGGTGAACTGCAAAGGAAAAGAC---CTAGCGAGGTGGATCCAG------GGCTGCACAATCGTGAAACCATGGGACAAGTGGCGC---------------------------------

---

>Lizard_LyzN

------------------------------------------------------------------------ATGCTGATTTTGAAACTCATGGCTCCT---------------------------------------------------TTTTTAATCTTCCTGCTC---ATTGGGGCT

------------------------GGTGAAACCAAGATCATTTCGCGATGTGCCCTGGCTAATCTCATTGCCGATTTAGTCCCGGACGGGTATGCGCGATACACTATCAATGACTGGATCTGTCTCGCCCACTCTGCGAGCTATTTCGATTCAATGAAAATATCAAAACAGGAAGAGGAC

AGTGCCCGGAGATATGGGATATTCCAGTTCAGTAACATGGAACATTGCTCCGAC------GGATGC------CGCAGCTCCCTGAACATATGCCGCACGCAA------------TGCACAAACTTCATAAATGATGACTTGAGGGATGATATCCGGTGTGTGATG---------------

---AAAATA---------ATCAGAGCTGAC---ACTGGGTTCGACGAATGGAGAGTATATGGCGTGAACTGCAAAGGAAAAAAC---CTAGCGAGGTGGATCCAG------GGCTGCACAATCGTGAAACCGTGGGACAAGTGGCGC---------------------------------

---

**Supplementary Figure 17. Alignment of lysozyme-like gene coding sequences.** Lysozyme-like gene coding sequences from diverse vertebrate species (see Additional file 1: Table S1) and select outgroups (see Additional file 18: Table S2) were aligned using the MAFFT algorithm [56] as implemented on the Guidance web server [58,59]. The outputted alignment in fasta format is provided.
